# Redundant and Singular Regulatory Elements Underlie the Rapidly Evolving Pigmentation of *Drosophila*

**DOI:** 10.1093/molbev/msaf213

**Published:** 2025-09-04

**Authors:** Logan A Brubaker, Hayley Long, Allison Pavlus, Melissa E Williams, Devon M Seibert, Ashley V Williams, Marc S Halfon, Mark Rebeiz, Thomas M Williams

**Affiliations:** Department of Biology, University of Dayton, Dayton, OH 45469, USA; Department of Biology, University of Dayton, Dayton, OH 45469, USA; Department of Biology, University of Dayton, Dayton, OH 45469, USA; Department of Biology, University of Dayton, Dayton, OH 45469, USA; Department of Biology, University of Dayton, Dayton, OH 45469, USA; Department of Biology, University of Dayton, Dayton, OH 45469, USA; Program in Genetics, Genomics, and Bioinformatics, University at Buffalo-State University of New York, Buffalo, NY, USA; Department of Biochemistry, University at Buffalo-State University of New York, Buffalo, NY, USA; Department of Biological Sciences, University of Pittsburgh, Pittsburgh, PA 15260, USA; Department of Biology, University of Dayton, Dayton, OH 45469, USA

**Keywords:** *Drosophila*, *cis*-regulatory element, enhancer, pigmentation, redundancy, modularity, gene regulatory network, transcription factor

## Abstract

A major hurdle in understanding the molecular changes responsible for metazoan diversity is the characterization of *cis*-regulatory elements (CREs) for gene regulatory networks (GRNs). CRE changes are suspected to be commonplace in trait evolution, since such changes circumvent the deleterious effects of pleiotropy. A growing list of genes, though, is known to be regulated by redundant CREs. Such redundant CRE architectures complicate the characterization of GRN evolution, as they compound the effort to characterize each locus, and raise the questions of how and whether genes with redundant architectures evolve expression. Here, we used the evolution of sexually dimorphic abdomen pigmentation of *Drosophila* (*D.*) *melanogaster* as a model to study the function and evolution of CREs. Numerous sequences were evaluated that were previously predicted as potential abdomen CREs. Most of these predictions were validated, including two, four, and ten that, respectively, reside in the *homothorax*, *grainy head*, and *Eip74EF* transcription factor loci. The *homothorax* CREs were found to be partially redundant for this gene's pigmentation function, and pupal-stage Homothorax expression and the CRE activities were conserved among *Drosophila* species with the derived dimorphic and ancestral monomorphic phenotypes. Similarly, the *Eip74EF* CREs were conserved in the monomorphic *D. willistoni*. Thus, this gene's extensive CRE spatiotemporal redundancy has been conserved for over 30 million years, predating the dimorphic trait. Pigmentation evolution has been connected elsewhere to changes in nonredundant CREs. When these traits evolve, GRN changes may be biased towards the genes with singular nonredundant CREs, while the expression of redundantly regulated genes remains conserved.

## Introduction

The evolution of animal gene regulatory networks (GRNs) and their resulting gene expression patterns underlie much of the phenotypic variation that exists within and between species (Carroll 2008). *Cis*-regulatory elements (CREs) are genomic sites at which the upstream *trans*-regulators of GRNs connect to their phenotype-manifesting downstream target genes, so-called realizators (Hueber et al. 2007), to direct spatial and temporal patterns of gene expression. For many realizator genes and *trans*-regulatory loci, single CREs have been found that drive a modular (unique) pattern of their regulated gene's expression (Prud’homme et al. 2007). Conversely, a growing list of genes, often encoding transcription factors, has been shown to be regulated by multiple, seemingly functionally redundant, CREs with overlapping spatiotemporal activities (Wunderlich et al. 2015; Waymack et al. 2020; Kvon et al. 2021; Vitale et al. 2021; McDonald and Reed 2024). Thus, a contemporary question of broad importance is to determine whether redundant or singular CRE regulatory architectures are the exception or the norm for animal gene expression regulation and its evolution.

**Fig. 1.**
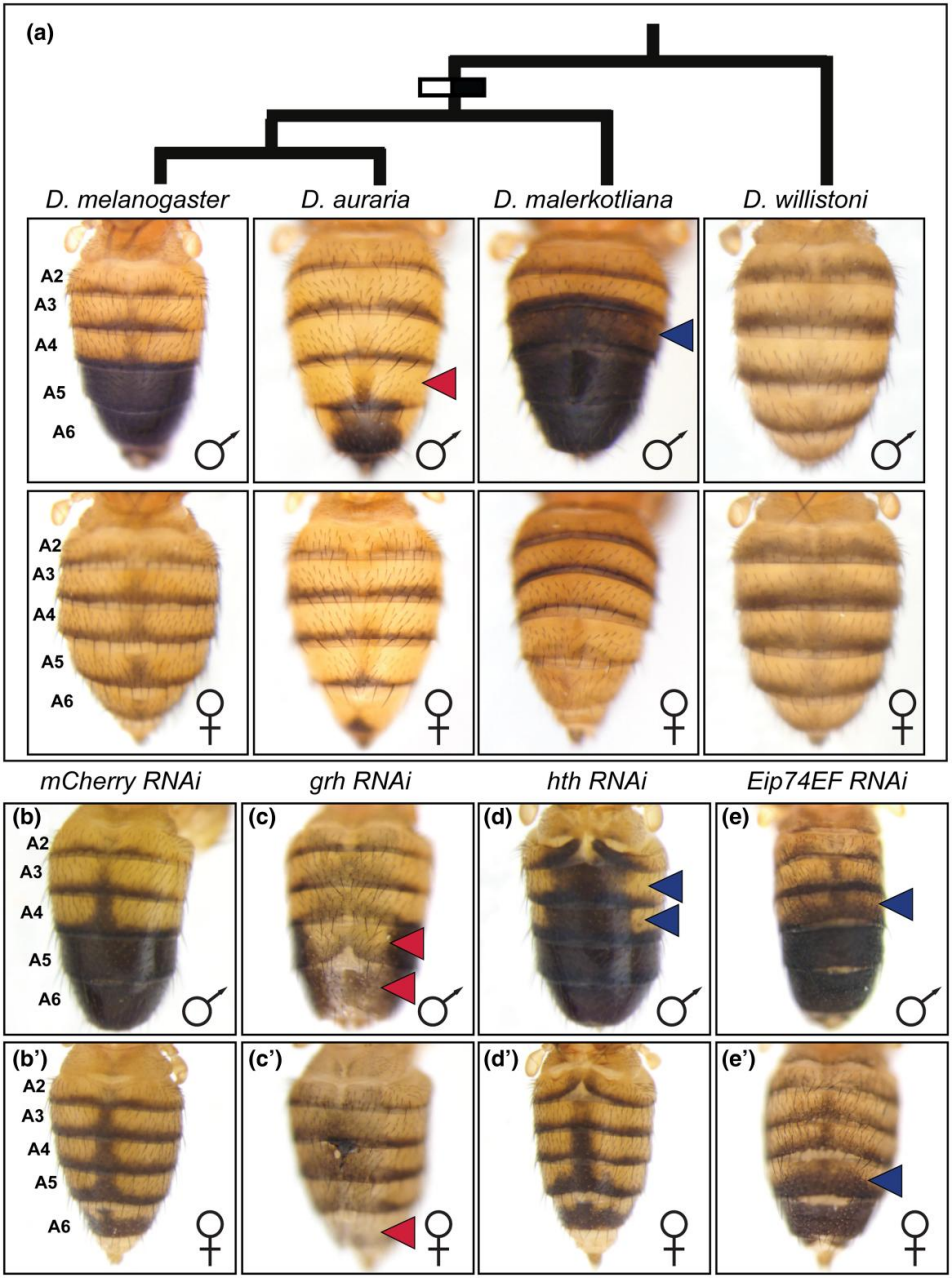
Abdomen pigmentation phenotypes and key transcription factor genes regulating pigmentation in *D. melanogaster*. a) *Drosophila* species studied here and their phylogenetic relationship. Black/White bar indicates the origin of male-specific melanic pigmentation. Number of melanic tergites was reduced for *D. auraria* and increased for *D. malerkotliana*. RNAi phenotypes in *D. melanogaster* b) to e) males and b′) to e′) females. b) and b′) RNAi for the negative control *mCherry* gene resulted in a wild-type pigmentation phenotype. c) and c′) Whereas RNAi for *grh* caused a loss of melanic pigmentation, and d) and d′) *hth* and e) and e′) *Eip74EF* resulted in ectopic pigmentation. Red and blue arrowheads, respectively, indicate abdomen tergites with reduced or ectopic melanic pigmentation. UAS-site-regulated RNAi transgenes were activated in the midline region under the control of the *pnr-GAL4* chromosome for *mCherry*, *grh*, and *hth*. *Eip74EF* RNAi was due to leaky expression of the UAS-transgene genomic insertion site.

The pigmentation patterns of *Drosophila* (*D.*) genus fruit flies are quite diverse, including a derived sexually dimorphic pattern on the posterior abdomens of the model organism species *D. melanogaster* (Fig. 1a) (Kopp et al. 2000; Jeong et al. 2006; Hughes et al. 2020). The A5 and A6 abdomen segments of males of this species are covered by fully melanic tergite plates on the dorsal surface, whereas melanic pigmentation is more limited to posterior stripes for conspecific females. These melanic stripes are similar to what is seen on the anterior A2 to A4 segments of both males and females. This dimorphic pattern of pigmentation evolved from an ancestral monomorphic state after the *melanogaster* lineage diverged from the *D. willistoni* lineage (Hughes et al. 2020). Subsequently, this derived dimorphism diversified, yielding species with more (*D. malerkotliana*) or fewer (*D. auraria*) melanic tergites in males (Fig. 1a).

The GRN and CREs for this derived trait have received considerable attention (Rogers et al. 2014; Rebeiz and Williams 2017). This includes the CREs controlling the expression of the realizator genes that encode pigment metabolic proteins (supplementary fig. S1, Supplementary Material online). *ebony* is one of the realizator genes, and its expression regulation is the most complex (Rebeiz et al. 2009), including modular enhancer and silencer-type CREs (Akiyama et al. 2022; Méndez-González et al. 2023). The other characterized pigmentation realizator genes, *yellow*, *tan*, and *Ddc*, appear to be regulated in the abdomen by singular nonredundant CREs (Jeong et al. 2006; Camino et al. 2015; Grover et al. 2018). Regarding the upstream *trans*-regulators in this GRN (supplementary fig. S2, Supplementary Material online), the tandem duplicate *bab1* and *bab2* genes were shown to be regulated by singular nonredundant CREs known as the *dimorphic element* and *anterior element* (Williams et al. 2008). The former CRE activates *bab* expression exclusively in the female A5 and A6 segments (Rogers et al. 2013), and the latter activates expression in the anterior segments of males and females (Williams et al. 2008). Collectively, these studies shape an impression that the pigmentation GRN genes are largely governed by CREs with modular functions. Dozens of additional *trans*-regulatory loci are known for this GRN (Rogers et al. 2014), though their expression-regulating CREs have gone largely uncharacterized.

Recently, the known pigmentation GRN CREs were used as a training set for the SCRMshaw CRE prediction tool to find other sequences genome-wide that may similarly function as pigmentation CREs (Weinstein et al. 2023). It was shown that over two-thirds of the predicted sequences functioned as CREs that direct pupal abdomen expression of a *nuclear-localized enhanced Green Fluorescent Protein* (*neGFP*) reporter gene in transgenic *D. melanogaster*. Among the newly identified CREs were two that function as redundant enhancers for *trithorax* (*trx*), three CREs in the *silverback* (*sbb*) gene, and four in the *Hormone receptor 4* (*Hr4*) gene that demonstrated spatiotemporal redundant regulatory activities in the abdominal epidermis. *trx* encodes a key part of a chromatin-regulating complex, and *sbb* and *Hr4* encode transcription factor proteins. This multiplicity of CREs suggested that redundant enhancers may be common for *trans*-regulatory loci in this GRN.

In this study, we explored the SCRMshaw CRE predictions for the *D. melanogaster* abdomen tergite pigmentation trait, focusing on *trans*-regulatory genes. Twenty-nine CRE predictions (referred to as *S3.1* to *S3.29* to distinguish this third (*S3*) set of tested SCRMshaw predictions from the first (*S1*) and second (*S2*) sets that were previously investigated [Weinstein et al. 2023]) were evaluated in *neGFP* reporter transgenes assays, with 22 showing activity in the pupal abdomen epidermis from which tergite pigmentation develops. Among the positives were four, two, and ten CREs that, respectively, reside in the *grainy head* (*grh*), *homothorax* (*hth*), and *Eip74EF* loci that encode transcription factor proteins. The two *hth* and ten *Eip74EF* CREs were shown to be older than the dimorphic pigmentation trait, indicating that these spatiotemporally redundant CRE architectures have persisted for more than 30 million years.

## Results

### Identification of Novel CREs for Eight *Trans*-regulators, Including Multiple CREs for *grh* and *hth*

Our previous sampling of the SCRMshaw abdomen pigmentation GRN dataset (Weinstein et al. 2023) left untested many predicted CREs (pCREs) residing in or near several *trans*-regulatory loci. This included four pCREs in each of the *grh* and *hth* loci, two transcription factor genes whose reduced expression, respectively, results in lost and ectopic melanic pigmentation (compare Fig. 1c to d and 1c′ to d′ to the negative control Fig. 1b and b′), which is consistently observed among replicate specimens (supplementary fig. S3, Supplementary Material online). We selected an initial 19 pCREs (Table 1), which included the four for both *grh* and *hth*, and tested each for enhancer activity in the abdomens of *D. melanogaster* pupae through *neGFP* reporter transgene assays. We found that 12 of the 19, or just over 63%, drove *neGFP* expression in the abdomen epidermis during late pupal development, when pigmentation patterning is well underway (supplementary fig. S4, Supplementary Material online). This set of 19 highlighted potential redundant CREs for select transcription factor genes in the pigmentation GRN. This included the *bab* locus, in which the *S3.2*, *S3.3*, and *S3.4* sequences reside, and the neighboring *trio* gene, in which the *S3.17* sequence is found within the second intron (supplementary fig. S5a, Supplementary Material online). However, none of these predictions drove compelling expression in the dorsal abdomen epidermis (supplementary figs. S5b to e and S6). Thus, the dimorphic expression of the *bab1* and *bab2* genes, to the best of our knowledge, appears to be under the control of the two previously identified nonredundant CREs: *dimorphic element* and *anterior element* CREs (supplementary figs. S4 and S5a, Supplementary Material online) (Williams et al. 2008).

**Table 1:** Predicted abdominal pigmentation CREs in the *D. melanogaster* genome from the Weinstein et al. SCRMshaw analysis with a second training set of CREs

Coordinates (pCRE)	Proximal gene 1	Proximal gene 2
3R:16830110 to 16830620 (*S3.1*)	*abd-A*	*iab-8*
3L:1108580 to 1109170 (*S3.2*)	*bab1*	*bab2*
3L:1064330 to 1064900 (*S3.3*)	*bab1*	*CG9205*
3L:1122130 to 1122770 (*S3.4*)	*bab2*	*bab1*
3L:5639880 to 5640390 (*S3.5*)	*Blimp-1*	*lin-28*
3R:13011880 to 13012770 (*S3.6*)	*CtBP*	*CG8031*
2R:17813810 to 17814480 (*S3.7*)	*grh*	*CR45270*
2R:17822120 to 17822690 (*S3.8*)	*grh*	*CR45270*
2R:17837680 to 17838600 (*S3.9*)	*grh*	*olf186-F*
2R:17823460 to 17824370 (*S3.10*)	*grh*	*olf186-F*
3R:10616720 to 10617350 (*S3.11*)	*hth*	*CR44018*
3R:10586900 to 10587700 (*S3.12*)	*hth*	*CR44018*
3R:10552660 to 10553180 (*S3.13*)	*hth*	*mir-4944*
3R:10535760 to 10536270 (*S3.14*)	*hth*	*mir-4944*
2L:248400 to 248950 (*S3.15*)	*kis*	*CR44218*
2L:166450 to 167270 (*S3.16*)	*spen*	*CG33635*
3L:1032040 to 1032690 (*S3.17*)	*trio*	*CG9205*
2R:11603050 to 11603930 (*S3.18*)	*tou*	*snoRNA:Psi28S-1180*
X:19774050 to 19774660 (*S3.19*)	*Zld*	*CG12702*

Among the set of 19 pCREs, several were mapped as the only prediction for their associated *trans*-regulatory loci (supplementary fig. S7, Supplementary Material online). One is the *S3.1* CRE that resides just 5′ of the first exon of the *Hox* gene *abdominal-A* (*abd-A*, supplementary fig. S7a, Supplementary Material online). This sequence demonstrated robust monomorphic (supplementary fig. S8, Supplementary Material online) enhancer activity throughout the dorsal epidermis of all abdomen segments, including A1 (supplementary fig. S7h, Supplementary Material online). Since the A1 segment is not a normal domain of *abd-A* expression (Rogers et al. 2014), there must be other sequences that sculpt the domain of *abd-A* expression. Other sequences with pupal abdomen epidermis enhancer activity were the *S3.15*, *S3.16*, *S3.18*, and *S3.19* CREs, which, respectively, reside in the *kismet* (*kis*), *split ends* (*spen*), *toutatis* (*tou*), and *zelda* (*zld*) loci (supplementary fig. S7d to g and S7k to n, Supplementary Material online). Each of these CREs drove monomorphic neGFP expression (supplementary fig. S8, Supplementary Material online). The *S3.18* CRE resides in the large first intron of the *tou* gene, and this element drives reporter expression broadly throughout the abdominal epidermis. This CRE is flanked by the *S2.11* and *S2.12* CREs (supplementary fig. S7f, Supplementary Material online) that were previously shown to possess strikingly similar enhancer activities (Weinstein et al. 2023). Thus, the *tou* locus possesses three spatiotemporally redundant enhancers.

**Fig. 2.**
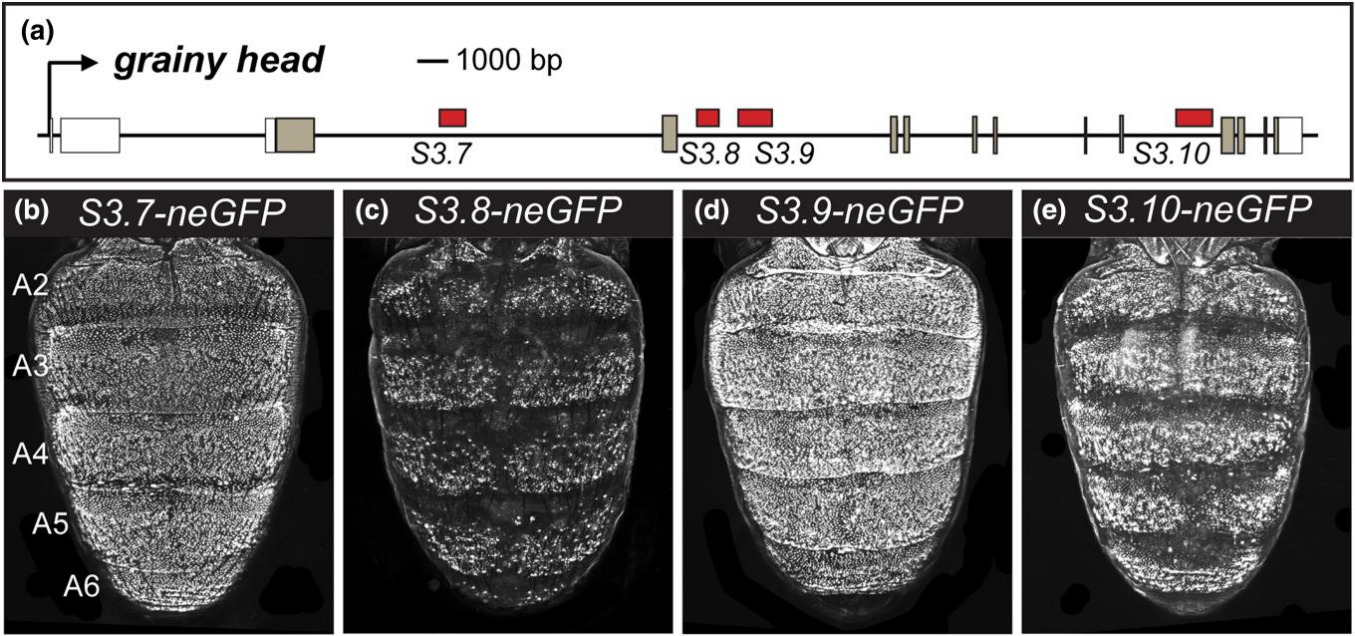
neGFP expressions driven by predicted CREs within the *grh* locus. a) *D. melanogaster grh* locus annotated with the locations of the *S3.7* to *S3.10* CREs. CRE rectangles are colored red to refer to their capability of activating reporter expression in the dorsal abdomen epidermis. b) to e) neGFP reporter expression in the dorsal abdomens of *D. melanogaster* pupae between 80 and 95 hAPF..

**Fig. 3.**
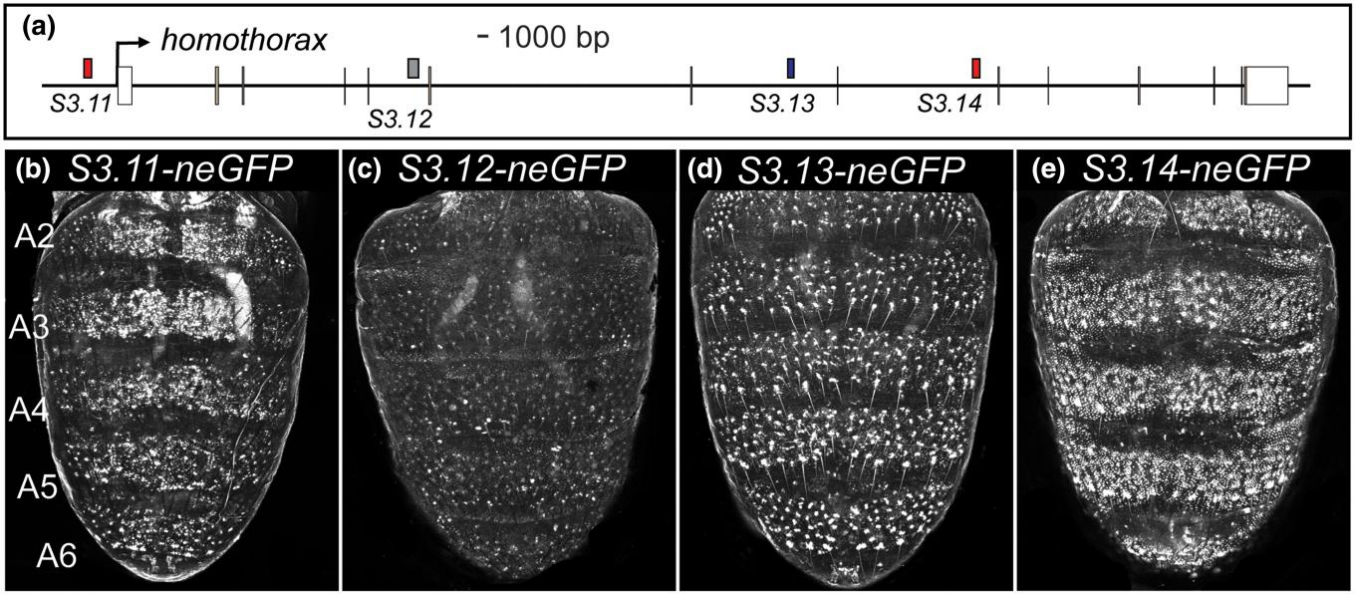
neGFP expressions driven by predicted CREs within the *hth* locus. a) *D. melanogaster hth* locus annotated with the locations of the *S3.11* to *S3.14* CREs. CRE annotations colored in red, blue, and gray, respectively, indicate either abdomen epidermis, other cell type, or no noteworthy neGFP expression. b) to e) neGFP reporter expression in the dorsal abdomens of *D. melanogaster* pupae between 80 and 95 hAPF. b) *S3.11* drives more widespread neGFP expression in the epidermis of the A2 and A3 segments than in the more posterior A4 to A6 segments. c) The *S3.11* sequence shows little to no dorsal abdomen enhancer activity, d) while the *S3.13* sequence acts as a CRE with abdomen bristle regulatory activity. e) *S3.14* CRE drives neGFP expression throughout the dorsal epidermis of the A2 to A6 segments..

While the role of *tou* in the development of the dimorphic pigmentation phenotype remains poorly understood, the roles of *grh* and *hth* are better resolved. *grh* function is necessary for the development of melanic pigmentation (Fig. 1c and c′), and Grh expression occurs throughout the abdomen epidermis, where it binds to and activates *Ddc* expression through binding to the *MEE1* CRE (Rogers et al. 2014; Grover et al. 2018). All four of the *S3.7* to *S3.10* sequences are located in a *grh* intron (Fig. 2a), and were found to possess monomorphic (supplementary fig. S9, Supplementary Material online) dorsal pupal-stage abdomen epidermis enhancer activity in *neGFP* reporter transgene assays (Fig. 2b to e). These four CREs were active during a similar critical time period, when tergite pigmentation is being patterned (supplementary fig. S10, Supplementary Material online). *hth* function is needed to repress melanic pigmentation formation in the A2 to A4 abdomen tergites of *D. melanogaster* males (Fig. 1d), abdomen regions where it acts as a direct repressor of *tan* expression through its binding to a site in the *t_MSE2* CRE (Camino et al. 2015). The expression and regulation of *hth* during tergite pigmentation development had not been previously explored. We tested the *S3.11* to *S3.14* sequences for epidermis enhancer activity in transgenic pupae, and found such activity for the *S3.11* and *S3.14* CREs (Fig. 3b and e). The *S3.11* CRE had monomorphic activity that was more pronounced in the A2 to A4 segments, while the *S3.14* CRE drove monomorphic neGFP expression throughout the dorsal abdomen epidermis (Fig. 3b and supplementary fig. S11, Supplementary Material online). Like the *grh* CREs, the *hth S3.11* and *S3.14* CREs were active during the critical time period, when tergite pigmentation is being patterned (supplementary fig. S12, Supplementary Material online). These results suggest that *grh* and *hth* are transcription factors whose expression during tergite pigmentation development is under the control of CREs with spatiotemporally redundant regulatory activities.

**Fig. 4.**
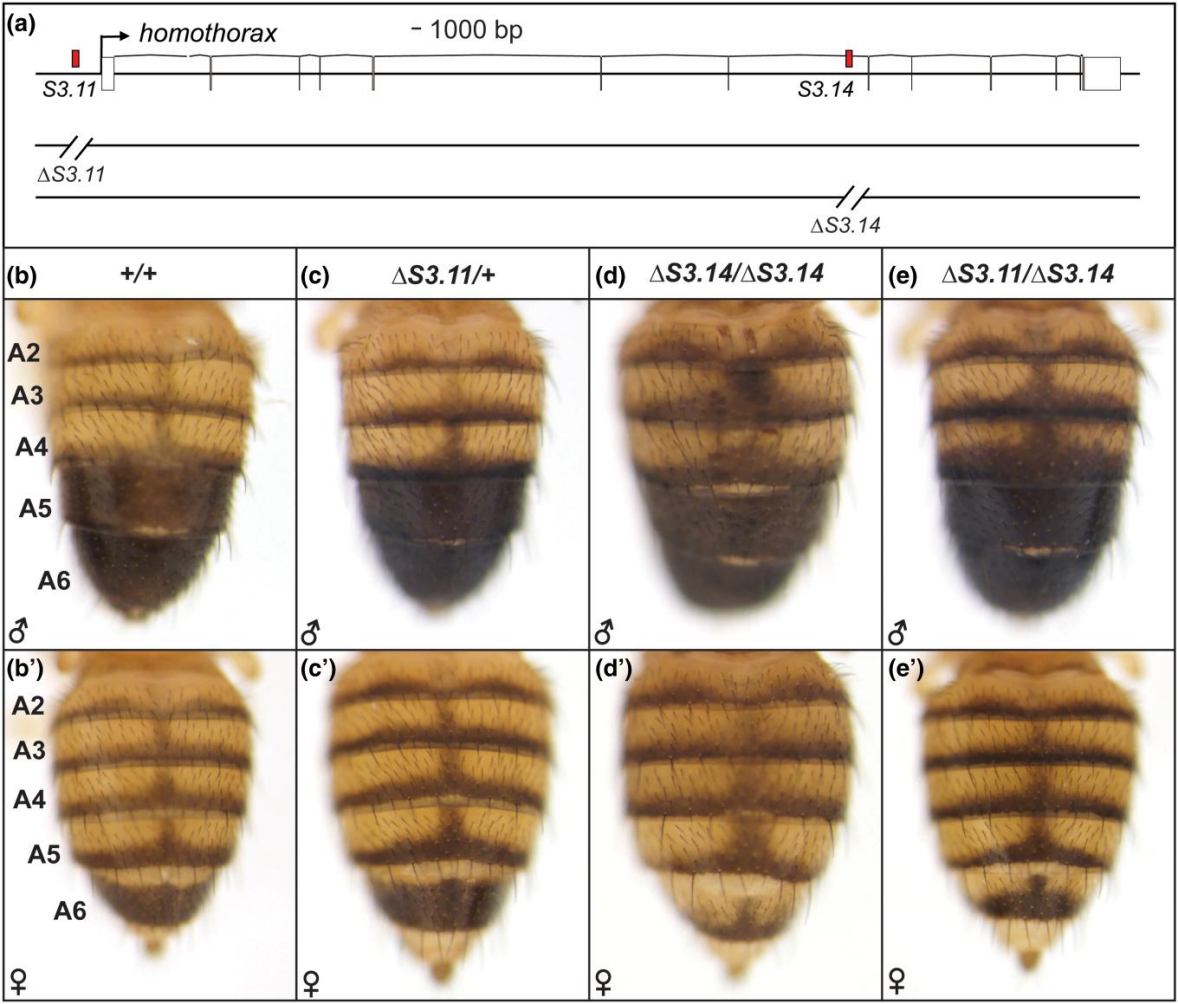
*Homothorax* function and its CREs are necessary for the robustness of the *D. melanogaster* pigmentation pattern. a) To-scale annotated representation of the *hth* locus. Black arrow indicates the site and direction of transcription initiation. Exons are the large rectangles, with those colored white comprising the 5′ and 3′ untranslated regions. The position of the *S3.11* and *S3.14* CREs each lies between a pair of gRNA target sites that were used to create the CRE deletion alleles by CRISPR/Cas9-mediated genome editing. The wild type pattern of b) male and b′) female tergite pigmentation from the *w1118* stock. The *S3.11* deletion is homozygous lethal, c) and c′) hence the pigmentation phenotype shown here for individuals heterozygous for this deletion. d) and d′) Pigmentation phenotypes for individuals homozygous for the *S3.14* deletion. d) Some ectopic melanic pigmentation is seen on the male A4 and A3 tergites of these *S3.14* deletion homozygotes. e) and e′) Individuals *trans*-heterozygous for the *hth S3.11/S3.14* deletions. e) Some ectopic melanic pigmentation is seen on the male A4 and A3 tergites.

Although CREs often regulate the expression of the gene in which they reside (intronic) or are proximal to, this is not always the case. Moreover, when a CRE with a striking patterning activity is found, it remains unknown whether this CRE functions in a nonredundant manner, or whether additional CREs with overlapping activities remain undiscovered. We were interested in whether the *S3.11* and *S3.14* CREs regulate *hth* function in vivo, and whether these two CREs are entirely responsible for *hth*'s pigmentation-suppressing function in the anterior male abdomen. To this end, pairs of Cas9 guide RNAs (gRNAs) were designed that flanked the *S3.11* sequence and, separately, the *S3.14* sequence (supplementary table S1, Supplementary Material online). These gRNAs were used to target double-stranded breaks by the Cas9 enzyme at the sides of the CREs, which was followed by homologous recombination with a *3XP3::GFP* donor cassette with appropriate flanking homology arms. These events resulted in the creation of the *hth^ΔS3.11^* and *hth^ΔS3.14^* CRE deletion alleles (Fig. 4a).

The *hth^ΔS3.11^* allele was homozygous lethal, and *hth^ΔS3.11^*/+ individuals had a pigmentation phenotype that was indistinguishable from those with a wild-type pigmentation genotype (compare Fig. 4b and b′ to c and c′). However, ectopic melanic pigmentation was observed with incomplete penetrance and variable expressivity (supplementary fig. S13, Supplementary Material online) in males with the homozygous *hth^ΔS3.14^*/*hth^ΔS3.14^* (Fig. 4d and d′) and *trans*-heterozygous *hth^ΔS3.11^*/*hth^ΔS3.14^* (Fig. 4e and e′) genotypes. These results confirm that the *S3.11* and *S3.14* CREs are necessary for the pigmentation-repressing function of the *hth* gene. Moreover, the modest pigmentation phenotype for these CRE deletion genotypes compared to the phenotype caused by *hth* RNAi (Fig. 1d) is consistent with a functionally redundant mechanism of *hth* regulation in the pupal abdomen epidermis that may include additional as-of-yet unknown CREs.

### *Homothorax* Expression and Its Regulation Predate the Origin of the Derived Dimorphic Pigmentation of *D. melanogaster*

**Fig. 5.**
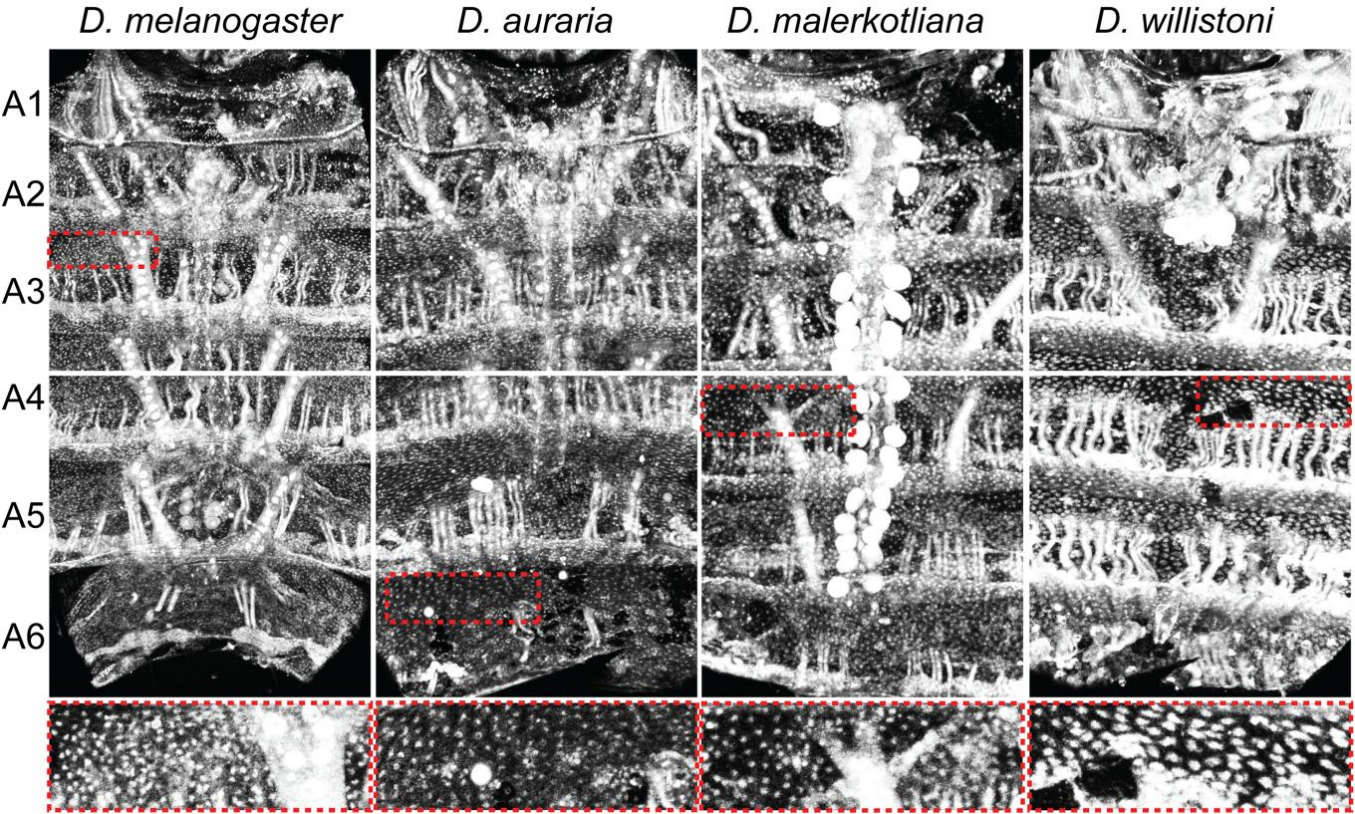
Homothorax expression in the dorsal abdomen epidermis is conserved at the equivalent stage in dimorphic and monomorphic *Drosophila* species. *Drosophila melanogaster*, *D. auraria*, and *D. malerkotliana* develop a derived male-specific pattern of abdomen tergite pigmentation, whereas *D. willistoni* develops the ancestral monomorphic phenotype. Hth expression, using the affinity-purified rabbit anti-Hth antibody, was revealed in the dorsal abdomen sections of specimens at the stage equivalent to 75 hAPF in *D. melanogaster*. The *melanogaster* specimen shown here is a male, and the *auraria*, *malerkotliana*, and *willistoni* specimens shown are female. Representative sections were selected from each specimen, outlined by dashed boxes, and provided as a zoomed-in image. Epidermis nuclei are the small bright dots that are visible between the contiguous vertical muscle nuclei. Large bright centrally located dots in *D. malerkotliana* are the Hth-expressing pericardial nephrocyte cells. In several samples, the heart tube and associated nephrocyte cells were removed without damaging the specimen.

Hth expression has been well-characterized in the *D. melanogaster* embryo, larval imaginal discs, and adult central nervous system, where both broad and spatially restricted patterns have been reported (Kurant et al. 1998; Pai et al. 1998; Azpiazu and Morata 2000; Ando et al. 2011; Hasegawa et al. 2011). Hth expression patterns during pupal development, though, remain largely uncharacterized. The ectopic pigmentation in the anterior male abdomen following *hth* RNAi (Fig. 1d) indicated that Hth must be expressed in the anterior male abdomen during the pupal stage of development, when pigmentation is being patterned. The activities of the *S3.11* and *S3.14* CREs (Fig. 3) suggest that Hth expression might be more widespread in the abdomens for both males and females. To observe Hth expression and its evolution during pigmentation development, an affinity-purified rabbit anti-Hth polyclonal antibody was made. This antibody detects nuclear-localized Hth in the *D. melanogaster* pupal abdomen epidermis and other closely associated cell types like muscle and nephrocytes (supplementary fig. S14, Supplementary Material online). 75 h After Puparium Formation (hAPF) in *D. melanogaster* (pupal development proceeds for nearly 100 h at room temperature) is a late pupal stage when key pigmentation GRN transcription factors and realizator genes are expressed. At this stage and beyond, Hth is expressed and localized to the nucleus of epidermis cells in the dorsal side of all abdomen segments, and expression is monomorphic (Fig. 5 and supplementary fig. S15, Supplementary Material online). Thus, Hth expression appears to match the combined epidermal patterns of reporter gene expression driven by the *S3.11* and *S3.14* CREs.

We were curious whether this pattern of Hth expression was conserved among dimorphic and monomorphic pigmented species. Samples were collected from *D. auraria* and *D. malerkotliana*, species with one fewer and one additional dimorphic pigmented segment, respectively (Fig. 1). Samples were also collected from *D. willistoni*, which has a monomorphic pattern of pigmentation (Fig. 1) and which is used as a surrogate for the monomorphic ancestral state (Salomone et al. 2013; Camino et al. 2015; Grover et al. 2018; Hughes et al. 2023). At the equivalent 75 hAPF developmental stage, Hth expression was observed in a broad, nuclear pattern similar to that seen for *D. melanogaster* (Fig. 5). This conserved pattern of Hth expression can be inferred to predate the origin of the male-specific pattern of tergite pigmentation. However, Hth expression persisted in *D. melanogaster* at 80, 85, and 90 hAPF, though expression was substantially reduced in *D. willistoni* (supplementary fig. S16, Supplementary Material online). These results hint at a scenario in which Hth's presence and function during late pupal development are somewhat derived.

**Fig. 6.**
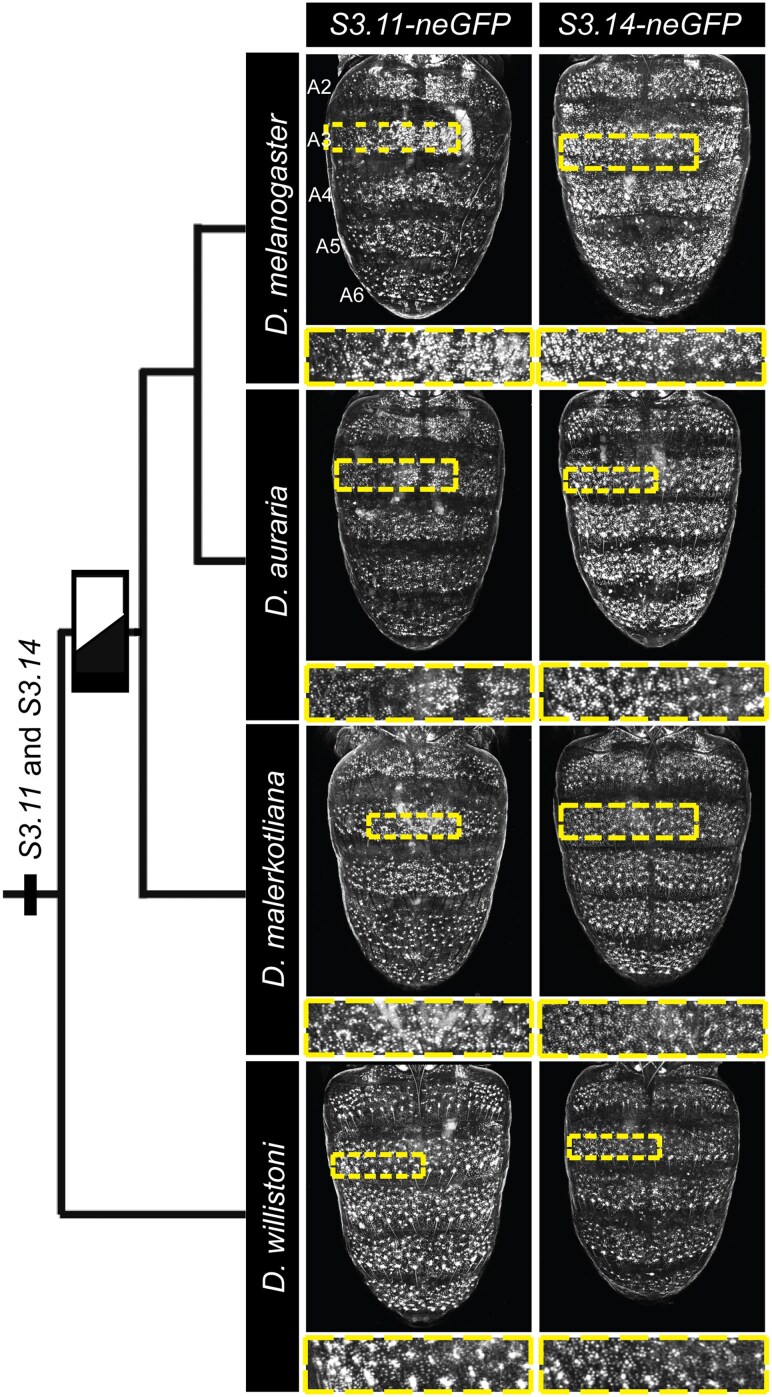
The regulatory activity of the *hth S3.11* and *S3.14* CREs is conserved. neGFP expressions driven by the *D. melanogaster S3.11* and *S3.14* CREs and the orthologous sequences from *D. auraria*, *D. malerkotliana*, and *D. willistoni*. All transgenes are situated in the 51-D site of transgenic *D. melanogaster*, and specimens shown here are at the ∼88 hAPF stage. The black/white bar on the phylogeny indicates the timepoint of origin for the male-specific pattern of tergite pigmentation. Select (dashed rectangles) abdomen regions were zoomed in on and provided below the full abdomen images to provide more detail on the neGFP expressions in epidermal cell nuclei. Based on the similar CRE activities, it can be concluded that the *S3.11* and *S3.14* CREs existed prior to the origin of the dimorphic pigmentation trait..

We wanted to further understand the evolutionary history of Hth expression and its regulation through the evaluation of sequences orthologous to the *D. melanogaster S3.11* and *S3.14* CREs. These sequences were obtained from the genome sequences available for *D. auraria*, *D. malerkotliana*, and *D. willistoni*, cloned adjacent to the *neGFP* reporter transgene, and integrated into the same *att*P site in the *D. melanogaster* genome. At the 85 hAPF stage, the *D. auraria* and *D. malerkotliana S3.11* and *S3.14* orthologous sequences drove similar patterns and levels of neGFP expression compared with the *D. melanogaster* CREs (Fig. 6 and supplementary fig. S17, Supplementary Material online). The *D. willistoni* sequences possessed pupal abdomen epidermis CRE activity, though the neGFP expression appears reduced compared with that driven by the dimorphic species CRE sequences. These results support a scenario in which the *S3.11* and *S3.14* CREs predated the origin of the dimorphic trait, and some functional changes occurred to these CREs in the lineage of the dimorphic species that augmented the activity of the *S3.11* and *S3.14* CREs. Such conservation and divergence are seen in alignments of the orthologous sequences (supplementary fig. S18, Supplementary Material online). The sequences of the *hth* and *grh* loci were compared between species with a derived dimorphic abdomen pigmentation phenotype, and multiple outgroup species with monomorphic pigmentation phenotypes. The observed patterns of sequence conservation support a scenario in which these pupal abdomen CREs existed before the origin of the dimorphic pigmentation trait (supplementary documents S1 and S2, Supplementary Material online and supplementary table S2, Supplementary Material online). However, a functional CRE activity for many of these orthologous sequences remains unconfirmed.

### The Highly Redundant Regulation of the *Eip74EF* Transcription Factor Gene and Its Evolution

**Fig. 7.**
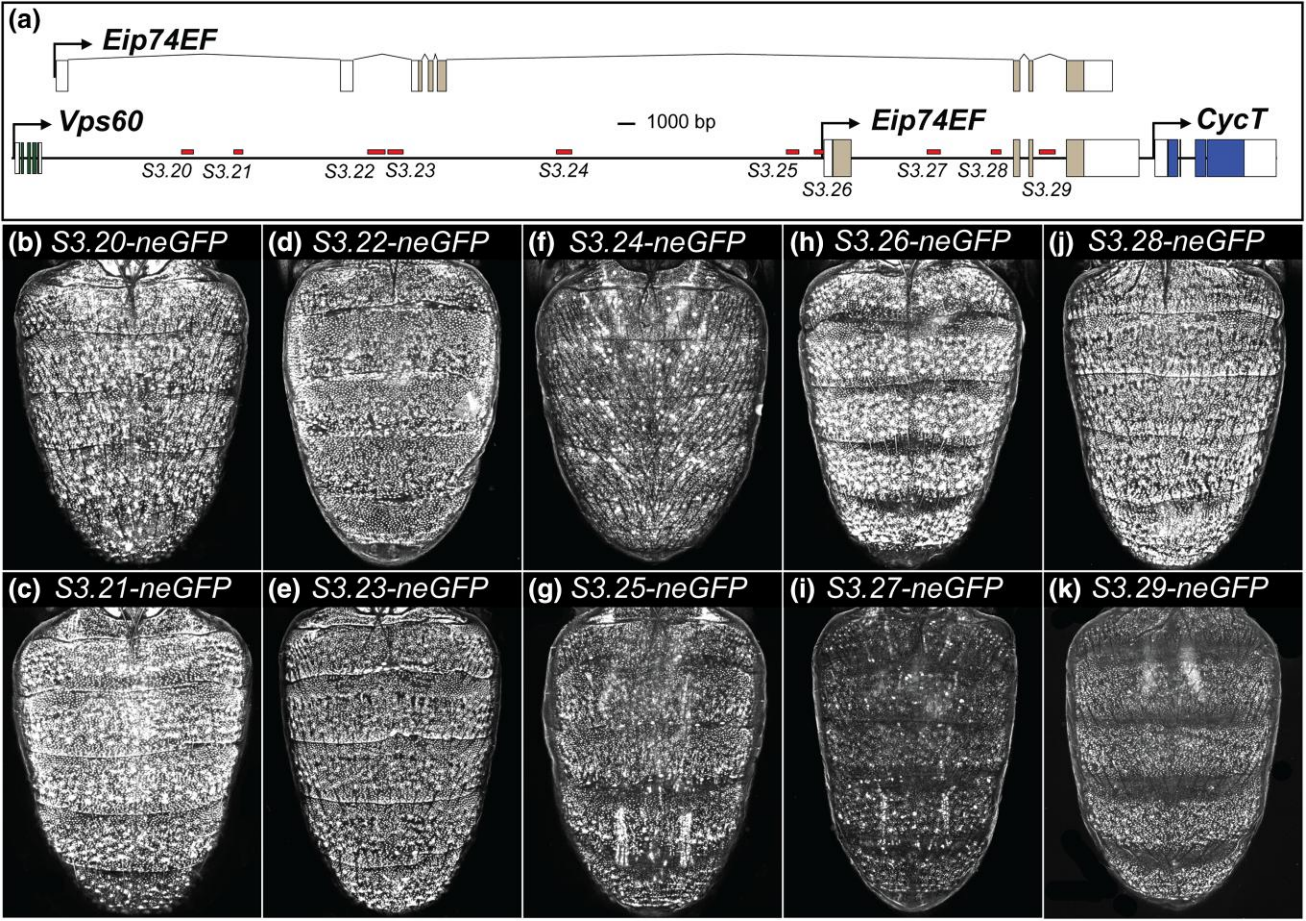
neGFP expressions driven by predicted CREs within the *Eip74EF* locus. a) *D. melanogaster Eip74EF* locus annotated with the locations of the *S3.20* to *S3.29* CREs. The exon and intron locations are shown for the *Eip74EF-RC* (above) and *Eip74EF-RD* (on the black line) transcripts. CRE annotations colored in red indicate abdomen epidermis neGFP expression. b) to k) neGFP reporter expression in the dorsal abdomens of *D. melanogaster* pupae at ∼88 to 95 hAPF.

The SCRMshaw tool predicted 10 unique sequences in the *Eip74EF* locus (named *S3.20* to *S3.29*; Table 2 and Fig. 7a) that might function as pupal abdomen CREs (Weinstein et al. 2023). This large number of potential CREs seemed at odds with the previous findings that *Eip74EF* either played a subtle role in suppressing melanic pigmentation of the female A6 tergite by RNA-interference knockdown (Rogers et al. 2014), or no apparent role in tergite pigmentation patterning in CRISPR/Cas9 conditional knockout progeny (Petrosky et al. 2024). To better resolve whether *Eip74EF* functions in pigmentation patterning and development, we identified several unique guide sequences to target by RNA-interference (supplementary table S3, Supplementary Material online). We selected two combinations (#1 and #6, and #4 and #9) of target sequences that we separately chained together as short hairpin RNAs (shRNAs) (Chang et al. 2014; Roeske et al. 2018) downstream of five UAS binding sites and a minimal *hsp70* promoter in a vector that possesses an *att*B site for genome integration. Though transgenic progeny were obtained by phiC31 integrase insertion into the standard *att*P2 and *att*P40 integration sites (Ni et al. 2008, 2009, 2011), these progeny were sickly and died before reproducing. Transgenic progeny was obtained at the *att*P154 and *att*P88 insertion sites with the #1 and #6 shRNA chain transgene, and these genotypes were viable for culturing. While *pnr-GAL4* activation of this shRNA chain in the dorsal body was lethal, uninduced leaky expression of this chain resulted in consistent ectopic pigmentation in the notum and abdomen tergites of male and female *D. melanogaster* (Fig. 1e and e′, and supplementary figs. S19 and S20, Supplementary Material online). These findings implicate *Eip74EF* as a broad repressor of melanic pigmentation formation.

**Table 2:** Predicted abdominal pigmentation CREs in the *D. melanogaster Eip74EF* locus from the Weinstein et al. SCRMshaw analysis with a second training set of CREs

Coordinates (pCRE)	Proximal gene 1	Proximal gene 2
3L:17590410 to 17591290 (*S3.24*)	*Eip74EF*	*snoRNA:Me28S-A576*
3L:17563370 to 17564290 (*S3.29*)	*Eip74EF*	*snoRNA:Me28S-A576*
3L:17577700 to 17578430 (*S3.25*)	*Eip74EF*	*snoRNA:Me28S-A576*
3L:17566390 to 17566970 (*S3.28*)	*Eip74EF*	*snoRNA:Me28S-A576*
3L:17576340 to 17576890 (*S3.26*)	*Eip74EF*	*snoRNA:Me28S-A576*
3L:17569800 to 17570560 (*S3.27*)	*Eip74EF*	*snoRNA:Me28S-A576*
3L:17600900 to 17601890 (*S3.22*)	*Eip74EF*	*Vps60*
3L:17599880 to 17600740 (*S3.23*)	*Eip74EF*	*Vps60*
3L:17611620 to 17612290 (*S3.20*)	*Eip74EF*	*Vps60*
3L:17608870 to 17609380 (*S3.21*)	*Eip74EF*	*Vps60*

The RNA interference (RNAi) phenotypes indicate that *Eip74EF* is normally expressed in pupal developmental stages and cell types involved in pigmentation patterning. This is consistent with the previous finding that *Eip74EF* average level of expression increased during the later stages of pupal development, though patterns of cell-specific expression remained unexplored (Brown et al. 2014). To better understand the onset and pattern of *Eip74EF* expression, *D. melanogaster* gene trap flies (supplementary figS21a, Supplementary Material online) were crossed to flies with a *UAS-neGFP* transgene. neGFP expression was observed in the dorsal pupal abdomen at ∼90 hAPF in some abdomen muscles and sporadically in other cells (supplementary fig. S21b, b′, c, and c′, Supplementary Material online). Just after eclosion from the pupal case, robust and widespread neGFP expression was observed in the epidermis (supplementary fig. S21d, d′, e, and e′, Supplementary Material online). Thus, *Eip74EF* expression seems to spike in the epidermis cells at eclosion, when it can shape the development of tergite pigmentation.

We were curious whether any of the ten, *S3.20* to *S3.29*, SCRMshaw predicted sequences among the *Eip74EF* locus showed enhancer activity in late-stage pupal abdomens that recapitulate some or all of the expression pattern seen from the gene trap line. Somewhat surprisingly, all ten sequences drove neGFP expression in the dorsal pupal abdomen epidermis (Fig. 7b to k and supplementary fig. S22, Supplementary Material online). The *S3.24* and *S3.25* sequences additionally activated GFP expression in some abdominal muscle cells as well (Fig. 7f and g). Like the *grh* and *hth* CREs, the ten *Eip74EF* CREs were active during the critical time period when tergite pigmentation was being patterned (supplementary figs. S23 and S24, Supplementary Material online). Thus, it seems that *Eip74EF* expression in the abdomen epidermis is under the control of numerous nonmodular CREs that exhibit spatiotemporal redundancy.

This seemingly highly redundant regulation of the *Eip74EF* gene raised several questions about its evolutionary history. One was whether some of these adjacent CREs resulted from tandem duplication events. To inspect for such evidence of duplication, the BLAST search tool was used to identify similar sequences between any two of the ten CREs. For nearly all comparisons, no noteworthy sequence similarities were found (supplementary fig. S25, Supplementary Material online). While two alignable sub-sequences were observed for the *S3.22* and *S3.23* CREs, the similarities were due to low-complexity repeats. Thus, these sequence comparisons do not provide compelling evidence that the redundant CREs originated from local duplication events.

**Fig. 8.**
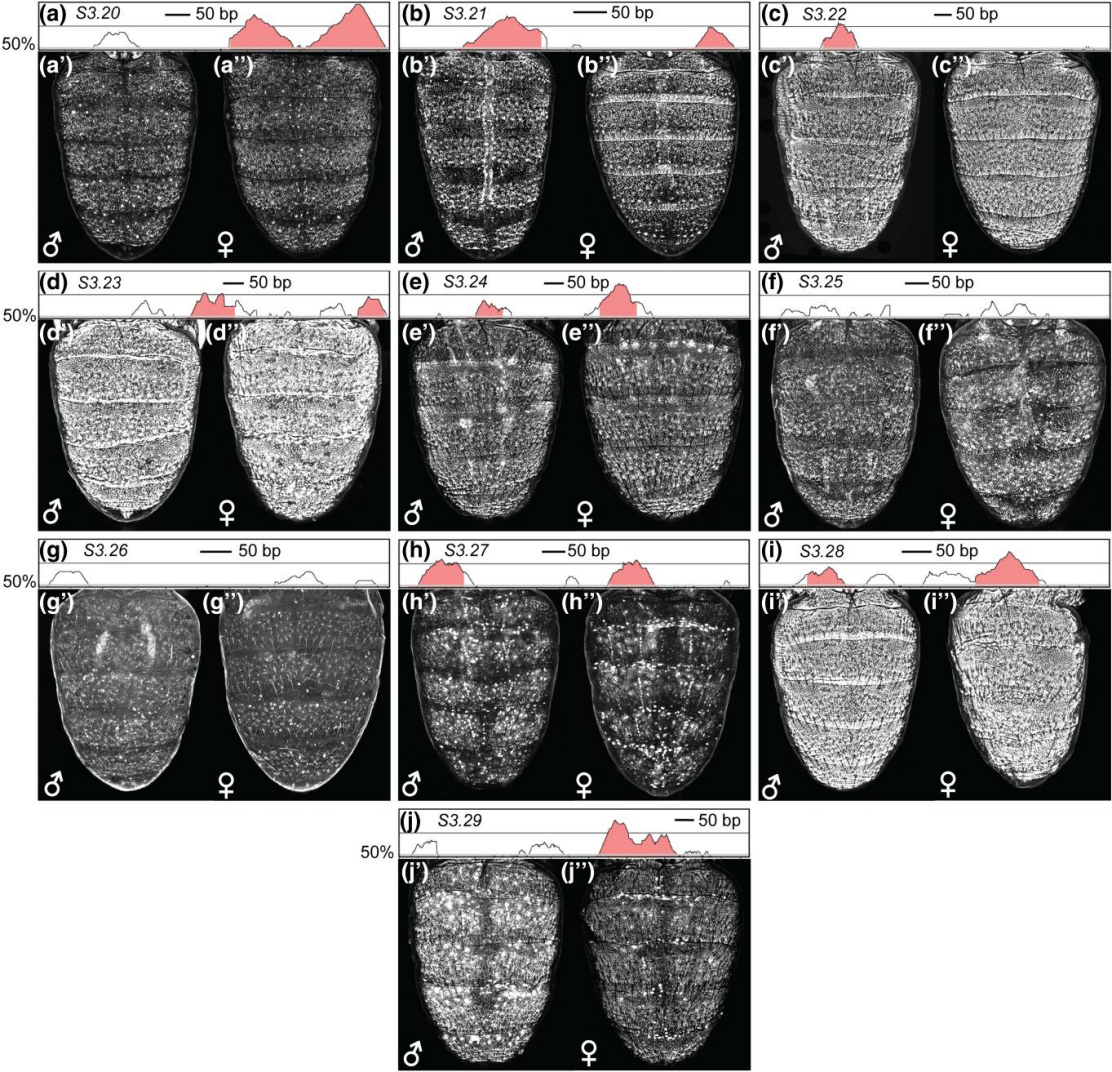
The activities of the *Eip74EF* CREs are conserved in *D. willistoni* despite substantial DNA sequence divergence. a) to j) mVISTA plots of DNA sequence conservation between the *melanogaster* and *willistoni* CRE sequences. Sequence conservation is annotated as peaks exceeding 50% identity to the melanogaster sequence. Conserved noncoding sequences are annotated as Salmon-colored peaks for which sequence identity is 70% or greater for 100 base pairs or more. a′) to j′) neGFP expression driven by an orthologous *willistoni* sequence in ∼88 hAPF stage transgenic *D. melanogaster* males. a′′) to j′′) neGFP expression driven by an orthologous *willistoni* sequence in ∼88 hAPF stage transgenic *D. melanogaster* females.

We wanted to explore the evolutionary history of these ten CREs by testing the orthologous sequences from the *Eip74EF* locus of the distantly related *D. willistoni*. Interestingly, all ten of the *D. willistoni* orthologous sequences drove neGFP expression in the abdomen epidermis (Fig. 8 and supplementary fig. S26, Supplementary Material online). Moreover, sequence conservation is observed between the *melanogaster* CRE sequences and orthologous sequences from a closely related species with the dimorphic pigmentation trait, as well as several outgroup species that exhibit ancestrally monomorphic phenotypes (supplementary document S3, Supplementary Material online and supplementary table S2, Supplementary Material online). These results indicate that all ten of the *Eip74EF* CREs identified in *D. melanogaster* have been conserved for more than 30 million years and predate the origin of the dimorphic pigmentation trait. Though the CRE activity for the orthologous sequences from the other ingroup and outgroup species, besides *melanogaster* and *willistoni*, remains inferred but not demonstrated.

## Discussion

**Fig. 9.**
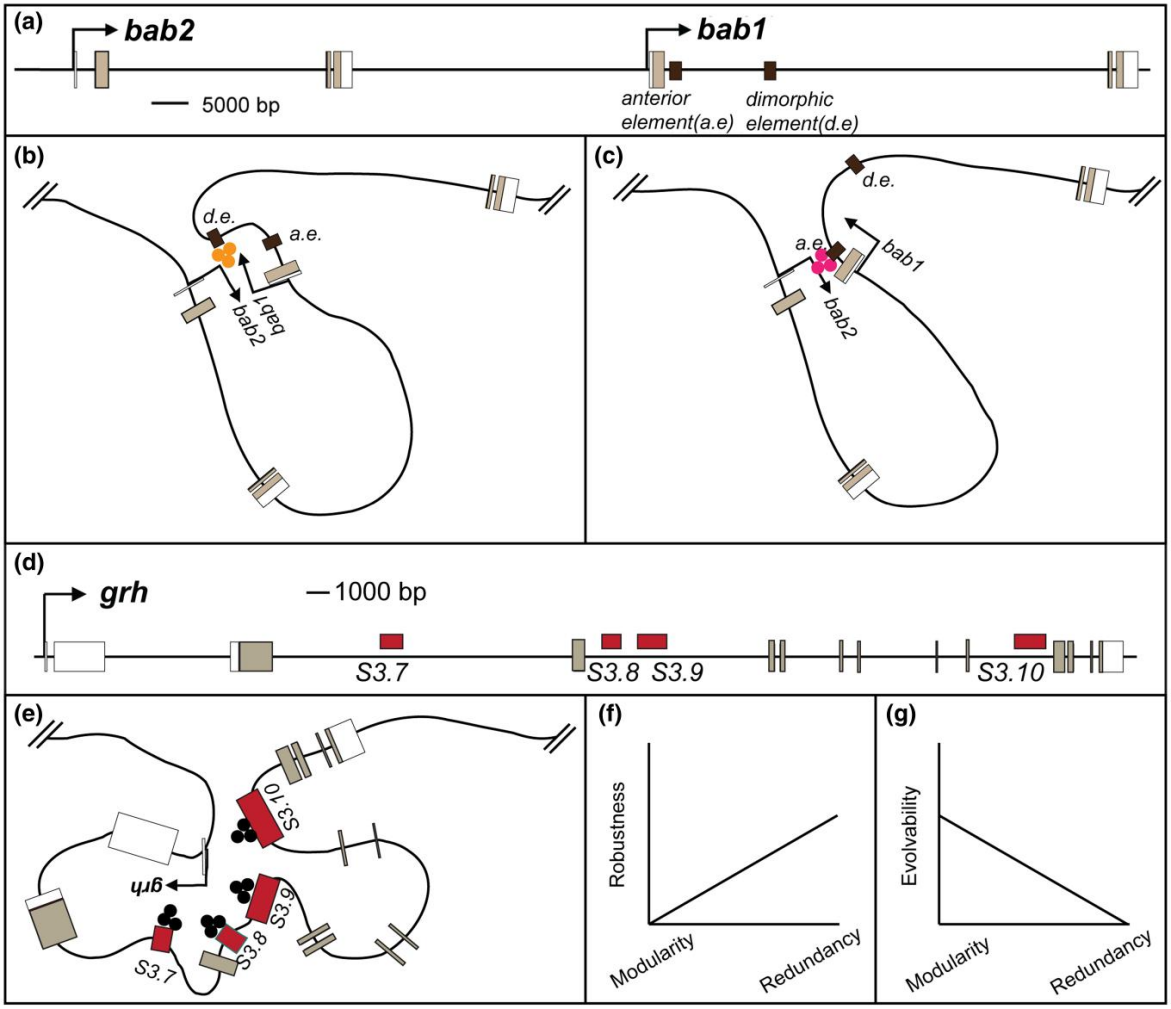
Modular and redundant regulated genes and their evolutionary potential. a) the *bab* locus contains the tandem duplicate *bab1* and *bab2* transcription factor genes that possess the *anterior element* (*a.e.*) and *dimorphic element* (*d.e.*) CREs in the large first intron of *bab1*. b) All evidence to date indicates that *bab* expression in the posterior female abdomen is under the modular activity of the *dimorphic element*. c) Likewise, *bab* expression in the anterior abdomen appears to be under the modular control of the *anterior element*. d) The *grh* locus contains four CREs (*S3.7* to *S3.10*) that each exhibit a similar abdomen enhancer activity. e) Abdomen expression of *grh* appears to be redundantly controlled, perhaps through a hub-type mechanism. f) A relationship may exist where expression patterns that are modularly regulated are less robust than those with redundant CRE control. g) The ability of gene expression to evolve may be inversely related to the degree of CRE redundancy..

One of the biggest challenges in studying GRN biology is the identification of CREs with specific activity, especially in large *trans*-regulatory loci. Finding these CREs is critical for understanding the evolution of the GRN, as the only way to discern *trans*- versus *cis*-regulatory evolution is to identify these CREs and test the orthologous sequences from different lineages. Here, our work shows how the SCRMshaw approach provides rapid inroads into mapping the CREs of a GRN. We successfully found pupal abdomen epidermis CREs from the large *D. melanogaster hth*, *Eip74EF*, and *grh* transcription factor loci. The SCRMshaw dataset (Weinstein et al. 2023) included numerous instances where two or more CRE predictions resided within the same gene. In the cases of *grh*, *hth*, and *Eip74EF*, the predictions were mostly confirmed and revealed CREs with strikingly similar, considered spatiotemporally redundant, activities, including 2, 4, and 10 CREs, respectively, for *hth*, *grh*, and *Eip74EF*. The seemingly redundant CREs for *hth* and *Eip74EF* were shown to be conserved for over 30 million years. Previous studies into the evolution of the derived dimorphic pattern of abdomen pigmentation for *D. melanogaster* revealed genes whose evolved expression patterns occurred through changes in CREs with singular nonredundant activities (Fig. 9a to c). In this study, we identified *trans*-regulatory genes with redundant CREs whose activity has remained deeply conserved (Fig. 9d and e). Our results hint at the interesting possibility that evolutionarily static portions of GRNs may be subject to complex combinations of redundant CREs, while change may be biased to occur in loci with simpler nonredundant architectures (Fig. 9f and g).

## CRE Modularity and Redundancy in the Development and Evolution of Fruit Fly Abdomen Pigmentation

The gene, CRE, and GRN basis for the development and evolution of the male-specific (dimorphic) pattern of *D. melanogaster* abdomen tergite pigmentation has received considerable attention. Two key realizator genes are *yellow* and *tan*, whose individual expression is under the control of a single nonredundant CRE that activates expression in the male A5 and A6 abdomen segments (Camino et al. 2015). The origin of the dimorphic trait and its subsequent loss were shown to include the creation and degradation of the modular *yBE0.6* and *t_MSE* CREs (Jeong et al. 2006, 2008; Camino et al. 2015). A third realizator gene is *Ddc*, and its robust expression in the posterior abdomen segments is under the control of the modular *MEE1* CRE (Grover et al. 2018). The evolution of *Ddc* expression was linked to changes in the *MEE1*, leading to an augmented activity. Thus, modularity appears to be the norm for this trait's realizator gene expression (supplementary fig. S1, Supplementary Material online), and evolution has occurred on multiple occasions through changes in these modular entities.

However, for a fourth realizator gene, *ebony*, the CRE architecture is more complex. *ebony* is expressed in an inverse dimorphic expression pattern, with broad expression that is specifically absent in pigmented male posterior body segments. *ebony's* expression is regulated by two redundant enhancers and three silencers that collaborate to sculpt patterns of *ebony* expression (Rebeiz et al. 2009). In *D. melanogaster* and closely related fruit fly species, abdomen pigmentation evolved through changes in *ebony* expression by the modification of silencers (Johnson et al. 2015; Liu et al. 2019). The *ebony* example highlights how CREs with silencer activities can drive expression changes that override redundant CRE architectures through changes in a single nonredundant element. Currently, silencers are much harder to identify than enhancers (Halfon 2020; Méndez-González et al. 2023). Applying SCRMshaw approaches to silencers may accelerate their identification once sufficient numbers of silencers have been identified.

*Drosophila melanogaster* male-specific pigmentation requires the dimorphic expression of the tandem paralog *bab1* and *bab2* transcription factor genes (Kopp et al. 2000; Roeske et al. 2018). A survey of the entirety of the *bab* locus for CREs identified nonredundant elements whose complementary expression composes the entire *bab* expression domain (Fig. 9) (Williams et al. 2008). Moreover, the evolution of dimorphic Bab expression from the ancestral monomorphic pattern involved changes that altered the activities of these two CREs. Thus, CREs with modular activities extend to the *trans*-regulatory tier of genes for this pigmentation GRN.

Recently, though, it was shown that the *trx*, *Hr4*, and *sbb* pigmentation GRN *trans*-regulatory loci harbor multiple CREs with similar pupal abdomen enhancer activities (Weinstein et al. 2023). In this study, we further characterized the CRE architectures of *trans*-regulatory loci for the *D. melanogaster* pigmentation trait. Among the results was the discovery of a third abdomen enhancer in the *tou* transcription factor locus. The *grh* locus was shown to possess at least four CREs with seemingly redundant enhancer activities. Grh expression in the pupal abdomen was shown to be conserved between *D. melanogaster* and *D. willistoni* (Grover et al. 2018). Indicating that Grh expression remained conserved for over 30 million years, though the extent of its CRE conservation remains to be studied. We showed that the transcription factor Hth has an ancestral pattern of pupal abdomen expression that was conserved during the evolution of the derived dimorphic pigmentation. Likewise, two *hth* CREs were characterized with conserved activities that originated prior to the dimorphic pigmentation trait. Last but not least, the *Eip74EF* transcription factor gene was shown to function as a broad repressor of melanic pigmentation in *D. melanogaster*, including the abdomen. This gene's pattern of expression was shown to switch on at a late stage of pupal development. Impressively, this gene locus harbors at least ten CREs with similar pupal epidermis enhancer activities that have been conserved for more than 30 million years.

This study draws attention to a dichotomy among the genes in the dimorphic pigmentation trait's GRN: those like *bab* (Fig. 9a to c), for which expression is regulated by singular nonredundant CREs, and those like *grh* whose expression is controlled by apparently redundant CREs (Fig. 9d and e). The former class of genes is characterized by expression patterns that are less robust (e.g. female pigmentation is sensitive to Bab expression levels, dimorphic element genotypes, and environmental conditions (Gibert et al. 2007; Rogers et al. 2013; De Castro et al. 2018)), and amenable to expression evolution. The latter class is characterized by more robust patterns of expression that are less amenable to expression evolution (Fig. 9f and g).

## CRE Modularity and Redundancy as Guiderails for GRN and Trait Evolution

A single observation of a phenomenon can be considered an anecdote. It is intriguing to speculate whether the CRE modularity and redundancy paradigm applies more broadly to evo-devo traits. The elaborate polka-dotted pigmentation pattern of the *D. guttifera* wing evolved through the co-opted expression of the Wingless morphogen (Werner et al. 2010). This derived trait required *wingless* to become expressed in novel pupal wing territories, including where the veins interact with the distal wing margin. This expression feature is driven by the *vein-tip* CRE that was co-opted from an ancestral CRE with crossvein enhancer activity (Koshikawa et al. 2015). The numerous pigment spots along the longitudinal wing veins of *D. guttifera* require *wingless* expression that colocalizes with where the campaniform sensilla originate. This novel *guttifera*-specific pattern of wingless expression is driven by the novel *gutCS* CRE (Koshikawa et al. 2015). The *D. guttifera wingless* locus and its neighboring genes were thoroughly screened for CREs with wing activity, and it seems that no redundant CREs were found. While it is possible that redundant CREs may exist at an even greater distance than assessed, the novel *wingless* expression appears to have evolved through CREs that behave in modular manners. Similarly, male-specific wing spots evolved multiple times among *Drosophila*. In the cases of *D. biarmipes* and *D. tristis*, their pigmentation spot evolved in part through a novel expression domain of the gene *yellow* (Prud’homme et al. 2006). For both species, the spot pattern of expression is driven by nonhomologous but similarly functioning modular CREs.

Fruit fly larvae and adults are covered by patterns of nonsensory hairs called trichomes. A master regulator of trichome development is the transcription factor gene *shavenbaby* or *svb*. Trichomes are generally conserved among *Drosophila*, though larval trichomes were independently lost in *D. sechellia* and *D. ezoana* (Frankel et al. 2012). The ancestral pattern of embryonic *svb* expression is driven by at least six CREs with modular and some redundant activities (Frankel et al. 2010; Preger-Ben Noon et al. 2018). While the redundant CRE activities were shown to provide robustness to environmental perturbation (Frankel et al. 2010), changes to the activities of multiple CREs shaped the loss of *svb* expression and larval trichomes in *sechellia* and *ezoana* (McGregor et al. 2007; Frankel et al. 2012). Adult trichomes require *svb*, whose expression is regulated by a shocking number of CREs with seemingly redundant pupal epidermis activities in reporter transgene assays (Sabarís et al. 2024). This robust regulation might explain why the evolution of a naked patch of leg trichomes occurred through changes at other loci while *svb* expression remained conserved (Kittelmann et al. 2018).

Outside of arthropods, several cases of morphological evolution have been documented among vertebrates that result from modifications to CREs. These include cases of polydactyly and the loss of the tetrapod limb among snakes. Both evolved traits stem from mutations to the same modular CRE, called the *ZRS*, which activates the posterior limb bud expression of the *Sonic hedgehog* or *Shh* morphogen gene. In the case of polydactyly, mutations in the *ZRS* result in additional ectopic *Shh* expression in the anterior limb bud (Lettice et al. 2002, 2008; Kvon et al. 2020). For limblessness, the *ZRS* was altogether lost from some snake genomes (Kvon et al. 2016). Thus, we can consider polydactyly and limblessness as examples where the underlying gene expression evolved through changes in a single modularly functioning CRE.

Another well-studied vertebrate morphological trait is the repeated loss of pelvic spines among stickleback fish. A dominant genetic change driving pelvic structure reduction was the repeated deletion of a modular CRE known as *PelA*. This CRE normally drives pelvic expression of the *Pitx1* transcription factor gene (Chan et al. 2010). More recently, it was shown that an ancient, over 400 million years old, and conserved hindlimb CRE exists, called *PelB*, which is additionally inactivated in stickleback fish with more complete losses of their pelvic appendages (Thompson et al. 2018). Thus, *Pitx1* expression in stickleback is driven by two CREs with somewhat modular and redundant functions. While mutations to a single CRE can lead to modest phenotypic change, mutations must involve both CREs to elicit a more complete loss of *Pitx1* expression. Interestingly, the hindlimb expression of *Pitx1* among tetrapods appears to be driven by more numerous CREs than seen for stickleback. Deletions of individual CREs for this gene generally result in subtle effects on *Pitx1* expression and morphology in mice (Kragesteen et al. 2018; Sarro et al. 2018; Thompson et al. 2018). Thus, it seems that tetrapods have a more redundant *Pitx1* CRE architecture, which may favor stability in expression over novelty.

A multiplicity of redundant CREs conferring robustness in gene expression regulation and development has been shown to be widespread in the mouse genome (Osterwalder et al. 2018). While exceptions are commonplace in biology, the body of evo-devo literature described here is consistent with an interpretation where novel expression patterns emerge from modular CRE architectures, while the genes with prolonged conserved expression patterns will be governed by architectures with CRE redundancy.

## The Future of Evo-Devo Studies on Gene Expression Regulation and Its Evolution

It was previously articulated that evolutionarily constrained traits will exhibit robust CRE regulatory architectures, while rapidly evolving traits will have more easily mutable regulatory architectures (McDonald and Reed 2024). This study and others on *Drosophila* abdomen pigmentation show how an evolving GRN can include robustly regulated constrained genes and nonredundantly regulated, more easily mutable genes. While this dichotomy is likely to be common among evolving traits, exceptions certainly exist. CRE redundancy likely impacts the level of expression for the target gene of regulation. Quantitative differences in expression levels can certainly result in quantitative differences in a trait's phenotype. Different modes of interaction have been attributed to redundant enhancers that include both additive and repressive interactions (Kvon et al. 2021). It is possible that more complex interactions, including silencing mechanisms, might provide opportunities for CRE redundancy to contribute to morphological novelty.

Studies of isolated CREs have been extremely useful for identifying cases of CRE evolution in the evo-devo field. As shown in this study, reporter transgene assays can identify sequences that exhibit spatiotemporal redundancy for a gene expression-regulating activity. However, it is important to note that spatiotemporal redundancy does not confirm that two or more sequences are functionally redundant in vivo and confer gene expression and phenotypic robustness. Future studies will need to innovate additional manipulative approaches (such as those involving large transgene clones or massive gene edits) along with comprehensive phenotypic analysis to validate perceived cases of CRE functional redundancy and to explore its propensity for constraint, and to reveal the types of CRE interactions that might allow for expression evolution. This innovation is essential to sufficiently interrogate loci where potential redundant architectures extend to three or more CREs. Such loci likely require deleting two or more CREs in order to expose the expression-buffering capacity and fitness consequences afforded by numerous elements with similar regulatory capabilities.

## Materials and Methods

### Fly Stocks and Genetic Crosses

All fly stocks used in this study were maintained at 22 ˚C and cultured on a sugar food medium that was described previously (Salomone et al. 2013 ). The species stocks used were the Kuala Lumpur *D. melanogaster* ([BGS] 3033.8, formerly 14021-0231.04), Guadeloupe Island *D. willistoni* ([Powell] Gd-H4-1, formerly 14030-0811.24), and Mysore *D. malerkotliana* ([BGS] 3253.5, formerly 14024-0391.00). These stocks were obtained from the San Diego Drosophila Stock Center, which is now located at Cornell University. The *D. auraria* stock was obtained from the lab of Sean B. Carroll when located at the University of Wisconsin. Transgenic *D. melanogaster* were created by Best Gene Inc., and CRE-deletion stocks were created by GenetiVision.

*Drosophila melanogaster* stocks possessing the *UAS-grh* RNAi (BDSC ID#28820), *UAS-hth* RNAi (ID#27655), *UAS-mCherry* (ID#35785), and *pnr-GAL4* (ID#3039) transgenes were obtained from the Bloomington Drosophila Stock Center. The effects of reduced *grh* and *hth* expression on melanic pigmentation development were observed for flies with a *UAS-gene specific RNAi*/*pnr-GAL4* genotype. The *pnr-GAL4* allele has a chromosome in which the *GAL4* gene is inserted in the *pannier* (*pnr*) locus, resulting in GAL4 expression in the dorsal–medial body (Calleja et al. 2000).

## CRE Prediction

The SCRMshaw pupal abdomen CRE prediction was previously described in detail (Weinstein et al. 2023). In brief, a set of 16 CREs with pupal abdomen epidermis regulatory activity were used as a training set to predict additional CREs genome wide. The analysis produced a list of over 500 sequences. In this study, we selected sequences within or adjacent to regulatory genes (transcription factors, coactivators, and corepressors) for validation (Tables 1 and 2).

## Novel neGFP Reporter Transgenes

Select pCREs were tested for their ability to activate the expression of a *neGFP* reporter gene in transgenic *D. melanogaster*. These so-called pCREs were inserted between the *Asc*I and *Sbf*I restriction enzyme sites of the S3aG vector (Rogers and Williams 2011), placing these sequences 5′ of the minimal *hsp70* promoter and the *neGFP* coding sequence. The primer pairs used to PCR-amplify the *D. melanogaster S3.1* to *S3.19* sequences are listed in supplementary table S4, Supplementary Material online.

The *Eip74EF S3.20* to *S3.29* pCRE sequences (supplementary document S4, Supplementary Material online) were synthesized and cloned into the *Asc*I and *Sbf*I sites of the S3aG vector. Orthologous sequences for the *D. melanogaster hth S3.11* and *S3.14* CREs were identified by BLAST analysis of the *D. auraria*, *D. malerkotliana*, and *D. willistoni* genomes. These sequences (supplementary document S5, Supplementary Material online) were synthesized and inserted into the *Asc*I and *Sbf*I sites of the S3aG vector. The orthologous sequences for the *Eip74EF S3.20* to *S3.29* CREs were identified by BLAST analysis of the *D. willistoni* genome. Primer pairs were designed to PCR-amplify each of the ten orthologous regions. The primer pairs are listed in supplementary table S5, Supplementary Material online, and the cloned *D. willistoni* sequences are in supplementary document S6, Supplementary Material online. DNA syntheses and cloning were done by GenScript Biotech. Each reporter transgene was inserted by Best Gene Inc. into the *D. melanogaster* second chromosome 51D *att*P site (Bischof et al. 2007) by ɸC integrase methods (Groth et al. 2004).

## CRE Removal by CRISPR/Cas9 Gene Editing

The design and steps to delete CRE sequences were previously described (Weinstein et al. 2023). For this study, the deletion of the *hth S3.11* and *S3.14* sequences was carried out by GenetiVision Corporation. The target site sequences for the gRNAs that flank the *S3.11* and *S3.14* sequences are provided in supplementary table S1, Supplementary Material online. The identified gRNA target sequences were cloned into an expression vector, and then a donor construct CRIMIC vector containing a *loxP* site-flanked *3xP3-GFP+* selectable marker cassette was made for each targeted CRE. Both donor vectors included approximately 1 kb of gene locus sequence that is immediately outside of the gRNA target sequence. To make the *S3.11* and *S3.14* CRE knockouts, flies from a *w1118* stock containing a *nos-Cas9* transgene were co-injected with a donor vector and its pair of gRNA vectors. CRE knockout flies were identified by GFP expression in the eyes and ocelli. The deletion was confirmed by DNA sequencing, and the deleted sequences that were replaced by the donor construct cassette are provided in supplementary document S7, Supplementary Material online.

## Antibody Production

An affinity-purified polyclonal antibody against *D. melanogaster* Hth-PC isoform was generated by Genscript (https://www.genscript.com/). The antigen used to immunize rabbits had a 6X His-tag followed by the Hth amino acid sequence: HGYHSGAGGHGTPSHVSPVGNHLMGAIPEVHKRDKDAIYEHPLFPLLALIFEKCELATCTPREPGVQGGDVCSSESFNEDIAMFSKQIRSQKPYYTADPEVDSLMVQAIQVLRFHLLELEKVHELCDNFCHRYISCLKGKMPIDLVIDERDTTKPPELGSANGEGRSNADSTSHTDGASTPDVRP. The His-tag was not removed during this procedure. This Hth sequence is present in all annotated Hth isoforms, has no noteworthy homology to any other annotated protein in the *D. melanogaster* proteome, is identical to *D. willistoni* and *D. virilis* Hth orthologs at 183 of 185 amino acids, and was previously used to create a Guinea Pig anti-Hth antibody (Özel et al. 2021).

## Immunohistochemistry

*Drosophila* specimens were dissected from the dorsal abdomens of pupae at various hAPF based upon *D. melanogaster* development at 25 °C. These specimens included the species *D. melanogaster*, *D. auraria*, *D. malerkotliana*, and *D. willistoni*. Other specimens were *D. melanogaster* with *pnr-GAL4/RNA interference transgene* genotypes (*hth* RNAi transgene and *mCherry* RNAi transgene), third chromosome genotypes. Specimens at different timepoints were identified based on the presence and absence of morphological markers (Ashburner et al. 2011). Morphological markers allow specimens to be obtained from *Drosophila* species whose time length of pupal development differs from that of *D. melanogaster*, such as *D. auraria*, *D. malerkotliana*, and *D. willistoni* (Grover et al. 2018; Hughes et al. 2020).

For the immunohistochemistry procedure, male and female specimens were combined in the same tubes to ensure the same conditions were experienced. All specimens were fixed for 35 min in PBST solution (phosphate-buffered saline with 0.3%Triton X-100) with 4% paraformaldehyde (Electron Microscopy Services). Once fixed, specimens were washed twice for 5 min in PBST and then placed in blocking solution (PBST with 1% Bovine Serum Albumin) for 1 h. Specimens were then transferred into PBST with a primary antibody and incubated overnight at 4 °C. The primary antibody used was a 1:100 dilution of the affinity-purified rabbit anti-Hth primary antibody, or an equivalent amount of purified IgG from a preimmunized rabbit. After primary antibody incubation, the specimens were washed four times for 5 min with PBST, and then transferred into blocking solution for an hour incubation. Specimens were then incubated in goat anti-rabbit Alexa Fluor 647 secondary antibody in PBST. The secondary was at a 1:500 dilution. The specimens were incubated for 2 h at room temperature. After secondary incubation, specimens were washed four times for 5 min in PBST and then equilibrated for 10 min  in a Glycerol Mount (50% glycerol, 50% PBST) solution. Specimens were then transferred to the glycerol mount (80% glycerol) before being placed on a glass slide with a coverslip for imaging. The coverslips and slides were separated by a piece of double-sided sticky tape that had a hole cut out in the middle by a razor blade. *Drosophila* specimens were situated in the center hole with the cuticle side adjacent to the coverslip.

## *Eip74EF* Small Interfering RNA Expressing Flies

The protein-coding exon sequences for the *Eip74EF* locus were obtained from the *D. melanogaster* genome using the GenePalette application (Rebeiz and Posakony 2004; Smith et al. 2017). Exons 7 to 9 are present in all five of the annotated transcripts, and were entered into the Designer of Small Interfering RNA (DSIR) algorithm that is accessible at: http://biodev.cea.fr/DSIR/DSIR.php (Vert et al. 2006). Exons 7, 8, and 9 had 5, 1, and 24 rows of output, respectively, which are included in supplementary table S3, Supplementary Material online and sorted by descending DSIR Score. To make sure candidate small interfering RNAs (siR) lack the same seed residues (nucleotides 2 to 8) as those present in known miRNAs, the guide sequences were searched against a miRNA database (http://mirbase.org) (Kozomara et al. 2019). Search results were shown for “Drosophila melanogaster” and the results are included in supplementary table S3, Supplementary Material online. RNAi was found to cause phenotypes in *D. melanogaster* when the guide shares at least 16 to 21 base pairs of contiguous sequence with the target gene (Haley et al. 2010). In order to avoid off-target effects, we prioritized the highest-scoring guide sequences with fewer than 16 contiguous bases matching a heterologous *D. melanogaster* exon. Guide sequence matches were sought by a BLAST search of the *D. melanogaster* genome (http://flybase.org/blast/) with the word size set to 7 (Crosby et al. 2007; Gramates et al. 2022). The genomic position of the BLAST hits was identified using the GBrowse feature, and the results are included in supplementary table S3, Supplementary Material online.

It has been shown that siR chains with multiple guide sequences can cause a greater reduction in a target gene's expression (Haley et al. 2008). Thus, we designed chains of two siRs targeting different *Eip74EF* sequences. These chains included the #1 and #4 sequences, and separately the #6 and #9 sequences (supplementary table S3, Supplementary Material online), and were synthesized with flanking *Kpn*I and *Eco*RI restriction enzyme sites (supplementary document S8, Supplementary Material online). These cassettes were cloned into the *Kpn*I and *Eco*RI sites of a custom-made vector (called pRedEyePig chain) that situates the siR chain sequences 3′ of five UAS sites and an *hsp70* heat-inducible promoter, and 5′ of the small t intron and an SV40 poly-adenylation signal containing sequence (Haley et al. 2008; Roeske et al. 2018). The vector additionally contains an *att*B sequence for site-specific integration by ΦC31 integrase, *3XP3-DsRed* transgene that allows the identification of transgenic flies by red fluorescent eyes and ocelli. Moreover, the entire fluorescent protein and siR chain cassettes are flanked by *piggyBac* inverted terminal repeats, which make this vector suitable for PiggyBac transposase-mediated transgenesis (Hughes et al. 2023). This vector was constructed by GenScript Biotech, and its sequence with the *Eip74EF* #1 and #6 siRs chain is included in supplementary document S9, Supplementary Material online.

Transgene vectors containing the chained guide sequences were site-specifically integrated into the *D. melanogaster att*P2 landing site by standard protocol (Best Gene Inc.). However, the offspring were sickly and did not reproduce. The *Eip74EF* siR chain vector was additionally integrated into the *attP*88 and *attP*154 insertion sites. Though sickly and exhibiting pigmentation phenotypes from leaky expression, these transgenic flies could reproduce. However, heat-shock-induced ectopic expression and *pnr-GAL4* conditional activation were lethal.

## Imaging of *Drosophila* Abdomens

Pigmentation patterns on dorsal abdomens of *Drosophila* were taken by an Olympus SZX16 Zoom Stereoscope with a mounted DP72 digital camera that was controlled by the Olympus CellSens Standard 2.2 software package. Specimens were imaged at magnifications of 50×. Prior to imaging, the legs, wings, and head of flies were removed from adult specimens between 5 and 7 d old. Specimens were then mounted on double-sided tape that was adhered to a conventional glass slide.

Projection images for the neGFP reporter transgene expressions in *D. melanogaster* were taken using an Olympus Fluoview FV 1000 confocal microscope and software. Samples were imaged at pupal developmental stages or a couple of hours after eclosion. For pupal-stage samples, pupae were removed from their puparium at the proper stage and then placed in halocarbon oil on standard glass microscope slides. Adult flies had their heads, legs, and wings removed and then were situated in halocarbon oil on a glass slide. Generic settings for imaging neGFP expression patterns were: laser 30% power (488 laser), HV between 650 and 700, gain set to 1, offset set to 1, Kalman line averaging set to 2, aperture set to 200 microns, and Z-series step size set to 10 microns.

Immunohistochemistry specimen images were taken with the Fluoview FV 1000 confocal microscope and software, with settings of: 20% laser power (647 laser), HV between 550 and 700, gain set to 1, offset set to 1, Kalman line averaging set to 2, aperture set to 200 microns, and Z-series step size set to 2 or 5 microns.

## Comparative Genomics

The mVISTA comparative genomic tool (Frazer et al. 2004) was used to identify conserved noncoding sequences in orthologous sequences of the SCRMshaw-identified *D. melanogaster hth* and *Eip74EF* CREs. For the *hth* S3.11 and S3.14 CREs, FASTA sequences for *D. melanogaster*, *D. auraria*, *D. malerkotliana*, and *D. willistoni* were uploaded to the mVISTA server. The alignments were completed with the Shuffle-LAGAN global pair-wise alignment, using default settings, which can detect rearrangements in aligned sequences. Orthologous *D. willistoni* sequences were similarly aligned with the *D. melanogaster Eip74EF* S3.20 to S3.29 CREs. Similar sequences within the *Eip74EF* S3.20 to S3.29 CREs were sought through the use of the Align Sequences Nucleotide BLAST tool (McGinnis and Madden 2004). Each pair of two sequences was inputted in FASTA format. The program selection was optimized for somewhat similar sequences.

The *grh*, *hth*, and *Eip74EF* orthologous loci were obtained by genome BLAST (McGinnis and Madden 2004) with the *D. melanogaster* sequence as the query and “Somewhat similar sequences (blastn)” as the program selection. The species whose genomes were interrogated were the dimorphic pigmented species *D. malerkotliana*, and the monomorphic outgroup species *D. pseudoobscura*, *D. willistoni*, *D. saltans*, and *D. virilis (*Richards et al. 2005; Drosophila 12 Genomes Consortium 2007; Signor et al. 2013; Prediger et al. 2024*)*. All species except for *D. virilis* are from the *Sophophora* subgenus (O’Grady and Kidwell 2002). *melanogaster* and *malerkotliana* are from the most distantly related lineages of the *melanogaster* species group, whose last common ancestor has been inferred to have possessed the derived dimorphic abdomen pigmentation phenotype (Jeong et al. 2006; Hughes et al. 2020). The other species represent ancestrally monomorphic groups (supplementary fig. S27, Supplementary Material online). Metadata information on the genomes and extracted orthologous sequences are provided in supplementary document S10, Supplementary Material online. Alignments of the loci were made using the mVISTA comparative genomics tool (Frazer et al. 2004). The five species were aligned to the *melanogaster* sequence using the Shuffle-LAGAN alignment program. mVISTA conservation plots were generated for the relevant *grh*, *hth*, and *Eip74EF* CREs (supplementary documents S1 to S3, Supplementary Material online). The length of conserved noncoding sequences and their percent identity were taken from the mVISTA output and compiled in supplementary table S2, Supplementary Material online. Full-locus mVISTA plots were also generated. For these, the position of the *D. melanogaster* CREs is annotated at the top of these alignments as short rightward-pointing gray arrows (supplementary documents S1 to S3, Supplementary Material online). The positions of noncoding and protein-coding exons are indicated as aqua and blue rectangles, respectively. Sequence conservation is annotated as peaks exceeding 50% identity to the *melanogaster* sequence. Conserved noncoding sequences are annotated as salmon-colored peaks for which sequence identity is 70% or greater for 100 base pairs or more.

## Figure Development

To-scale visualizations of the studied *D. melanogaster* gene loci were generated by the GenePalette tool (Smith et al. 2017). Sequence conservation plots for the *Eip74EF* and *hth* CREs were generated with the mVISTA tool (Frazer et al. 2004).

All neGFP and immunohistochemistry expression patterns shown were representative images selected from replicate specimens (*n* ≥ 3). TIFF images were processed through the same sequences of modifications using Adobe Photoshop CS3. Figures were assembled in Adobe Illustrator CS3.

## Supplementary Material

msaf213_Supplementary_Data

## Data Availability

All relevant data are within the paper and its supplementary materials that are available online.

## Supplementary Material

Supplementary material online will be made available at *Molecular Biology and Evolution* online.
