# Supplementary material for: Redundant and Singular Regulatory Elements Underlie the Rapidly Evolving Pigmentation of Drosophila
Source: Mol Biol Evol. 2025 Sep 4;42(9):msaf213. doi: 10.1093/molbev/msaf213 (PMC12449766; doi:10.1093/molbev/msaf213)
Supplement: msaf213_Supplementary_Data [file msaf213_supplementary_data.zip › Brubaker et al. supplement (2025) v4 no line numbers.pdf]

SUPPLEMENTARY MATERIAL

Redundant and Singular Regulatory Elements Underlie the Rapidly Evolving Pigmentation of *Drosophila*

Logan A. Brubaker[1], Hayley Long[1], Allison Pavlus[1], Melissa E. Williams[1], Devon M. Seibert[1], Ashley V. Williams[1], Marc S. Halfon[2,3], Mark Rebeiz[4], and Thomas M. Williams[1,*]

[1]Department of Biology, University of Dayton, 300 College Park, Dayton, OH 45469, USA

[2]Program in Genetics, Genomics, and Bioinformatics, University at Buffalo-State University of New York, Buffalo, NY, USA

[3]Department of Biochemistry, University at Buffalo-State University of New York, Buffalo, NY, USA

[4]Department of Biological Sciences, University of Pittsburgh, Pittsburgh, PA 15260

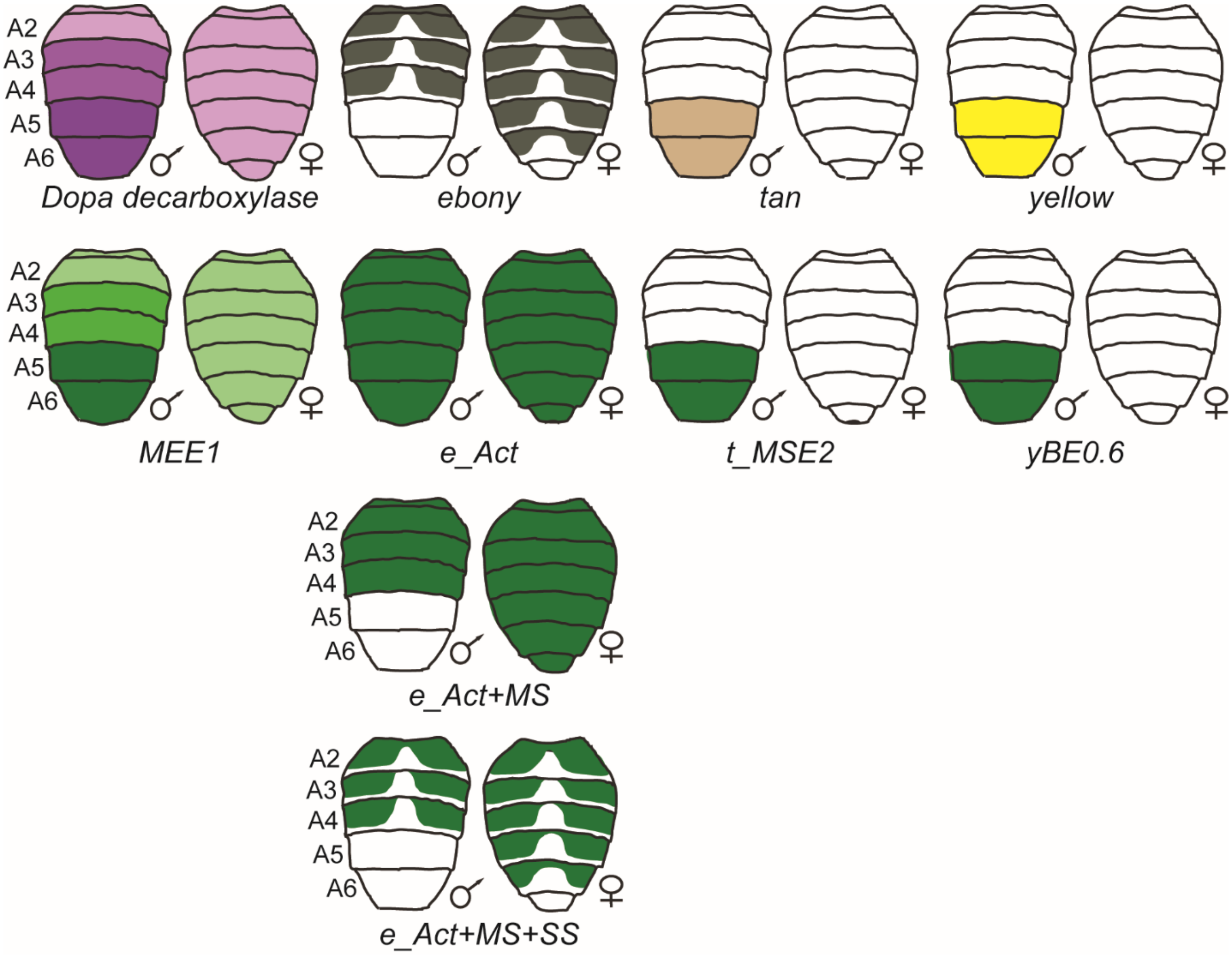

Supplementary Figure S1. Schematic representation of the expression patterns and modular activities of CREs that control pigmentation realizator expression in *D. melanogaster*. Patterns of realizator expression in the dorsal abdomen epidermis during tergite pigmentation and patterning are illustrated at the top for the genes that encode realizator proteins that function in pigmentation metabolism. Below in green depict the activities of CREs in *neGFP* reporter transgene assays from these loci. *Ddc*, *tan*, and *yellow* expression are under the control of singular modular CREs respectively referred to as the *MEE1*, *t_MSE2*, and *yBE0.6*. *ebony* expression is shaped by the modular activities of an enhancer (*e_Act*), a male-specific silencer (*MS*), and a posterior segment stripe silencer (*SS*).

Alt text: Schematic overview of the gene expression patterns and expression activities activated by singular *cis*-regulatory elements for *Drosophila melanogaster* pigmentation genes.

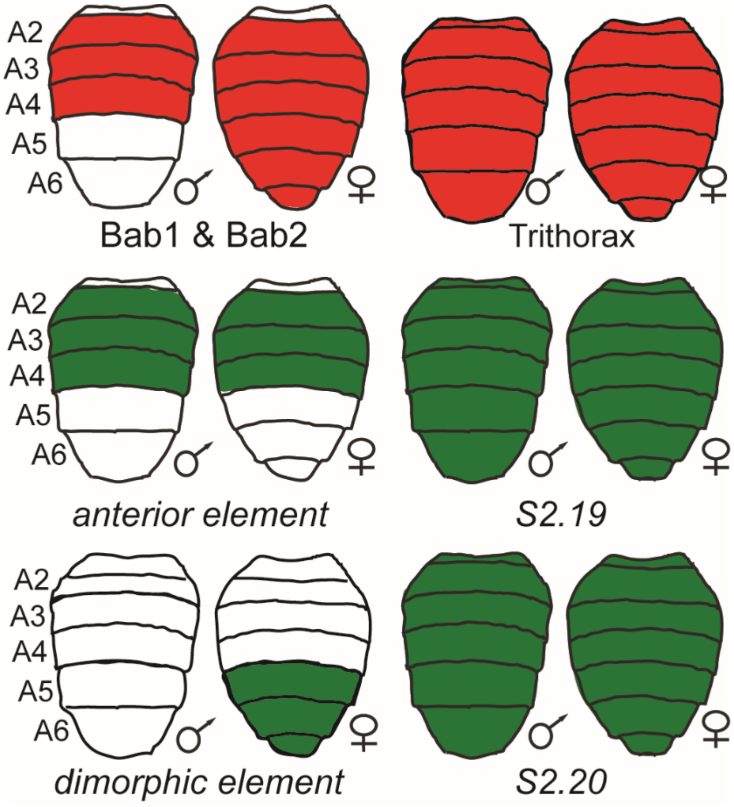

Supplementary Figure S2. Schematic representation of the expression patterns and activities of CREs that control abdomen pigmentation *trans*-regulator expressions in *D. melanogaster*. Patterns of protein expression in the dorsal abdomen epidermis during tergite pigmentation and patterning are illustrated at the top for the *trans*-regulatory proteins who's governing CREs were previously characterized. Below in green depict the activities of CREs in *neGFP* reporter transgene assays from these loci. The paralogous Bab1 and Bab2 proteins are regulated by the *anterior element* in the anterior A2-A4 segments, and the *dimorphic element* which has female specific enhancer activity in the A5 and A6 segments. Trx expression is under the redundant regulation of the CREs known as *S2.19* and *S2.20*.

Alt text: Schematic overview of the gene expression patterns and expression activities activated by *cis*-regulatory elements for *Drosophila melanogaster* regulatory genes that shape abdomen pigmentation.

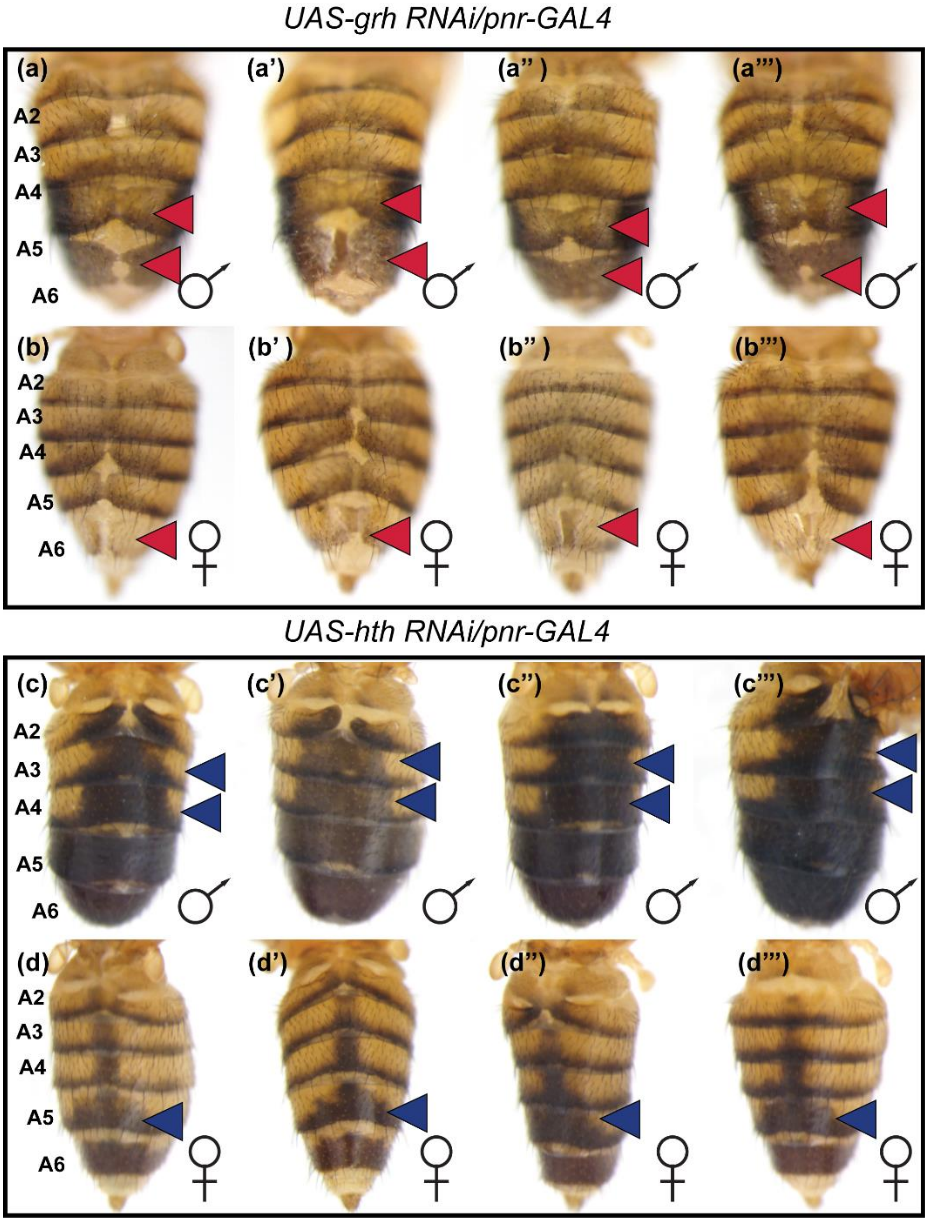

Supplementary Figure S3. RNA-interference for *grh* and *hth* caused consistent aberrations in abdomen tergite pigmentation patterns. The *pnrGAL4* driver activated the expression of an RNAi transgene targeting the (a-a''' and b-b''') *grh* and (c-c''' and d-d''') *hth* genes in the dorsal abdomen midline region. The adult abdomen pigmentation phenotypes are shared for four replicate (a-a''' and c-c''') male and (b-b''' and d-d''') female specimens. *grh* RNAi consistently resulted in reduced melanic pigmentation phenotypes in male and female specimens (red arrowheads). *hth* RNAi resulted in consistent ectopic pigmentation phenotypes in male and female specimens (blue arrowheads).

Alt text: RNA-interference for the *grh* and *hth* genes resulted in consistent aberrations in abdomen tergite pigmentation.

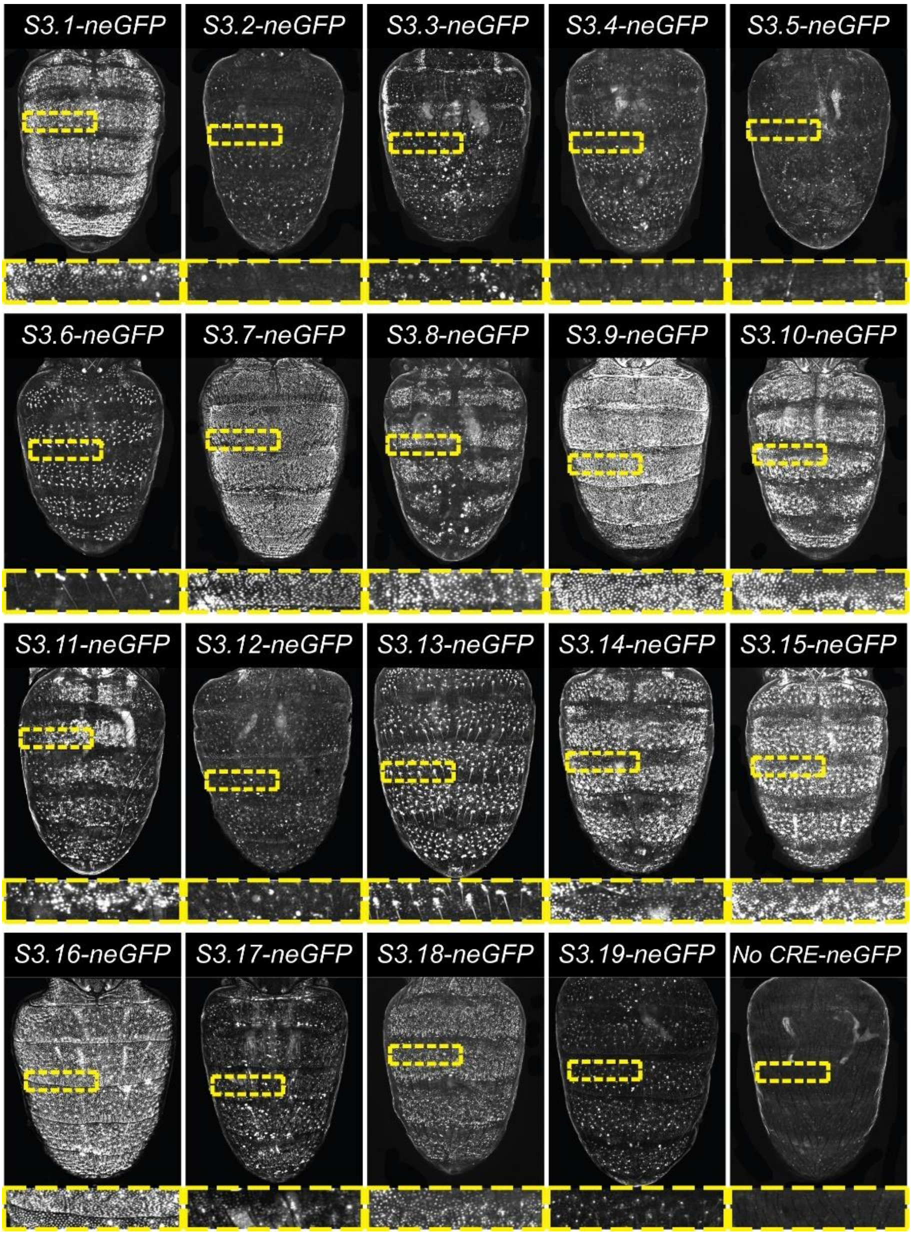

Supplementary Figure S4. neGFP expressions driven by 19 different predicted *cis*-regulatory elements (CREs) that are in or near a gene expression regulating loci. Representative neGFP expressions from reporter gene transgenes in the dorsal abdomens of *D. melanogaster* pupae. The developmental stage of specimens is between 80-95 hours after puparium formation (hAPF). *S3.1*, *S3.6-S3.11*, *S3.14-S3.18* drove neGFP expression in epidermis cells to an extent greater than that observed in control pupae for which the reporter *neGFP* gene did not have an adjacent CRE (No-CRE).

Alt text: Green Fluorescent Protein expression patterns activated in the *Drosophila melanogaster* pupal abdomen by nineteen predicted *cis*-regulatory elements. Each expression includes a regional zoom-in that provides a clearer visualization of the expressing cells.

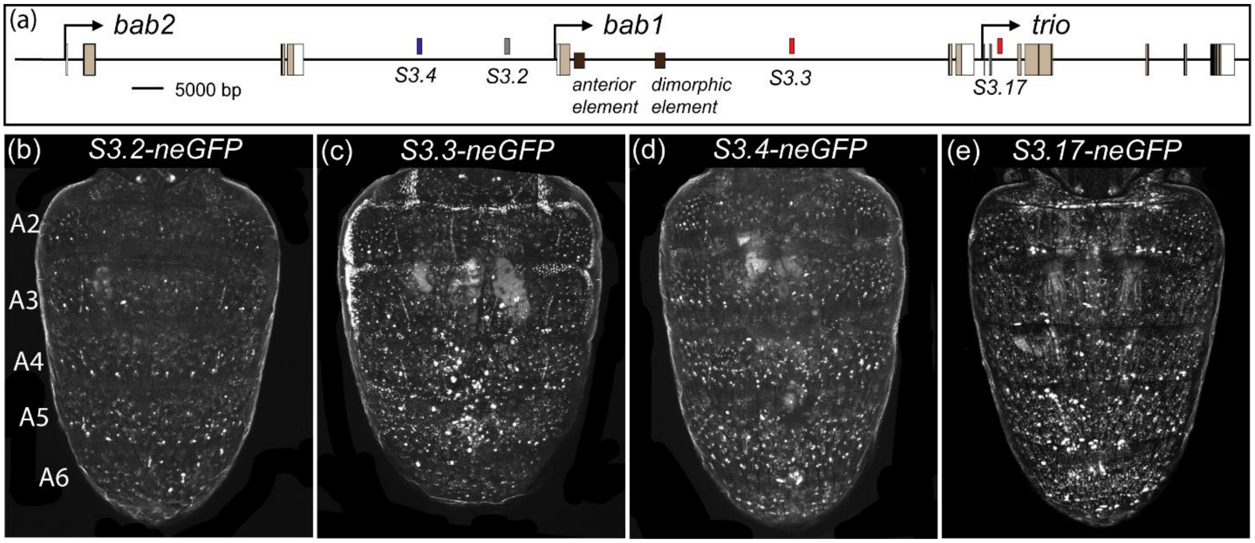

Supplementary Figure S5. neGFP expressions driven by predicted *cis*-regulatory elements (CREs) within the *bric-à-brac* and neighboring *trio* loci. a) *D. melanogaster bric-à-brac* (*bab*) and *trio* loci with the locations of the *S3.2*-*S3.4* and *S3.17* predicted CREs annotated. The *anterior element* and *dimorphic element* CREs are included here. These two CREs are known regulators of *bab* expression and they were included in the CRE training set used to identify the predicted CREs. CRE annotations colored in red, blue, and gray respectively indicate either abdomen epidermis, other cell type, or no noteworthy neGFP expression. b-e) *neGFP* reporter gene expression in the dorsal abdomens of *D. melanogaster* pupae between 80-95 hAPF.

Alt text: Subpanel a shares a to-scale representation of the *Drosophila melanogaster bab* gene locus with the positions of the studied *cis*-regulatory elements annotated. Subpanels b to e share Green Fluorescent Protein reporter transgene expressions in the pupal-stage abdomen that were activated by the four novel sequences that were tested for *cis*-regulatory element activity.

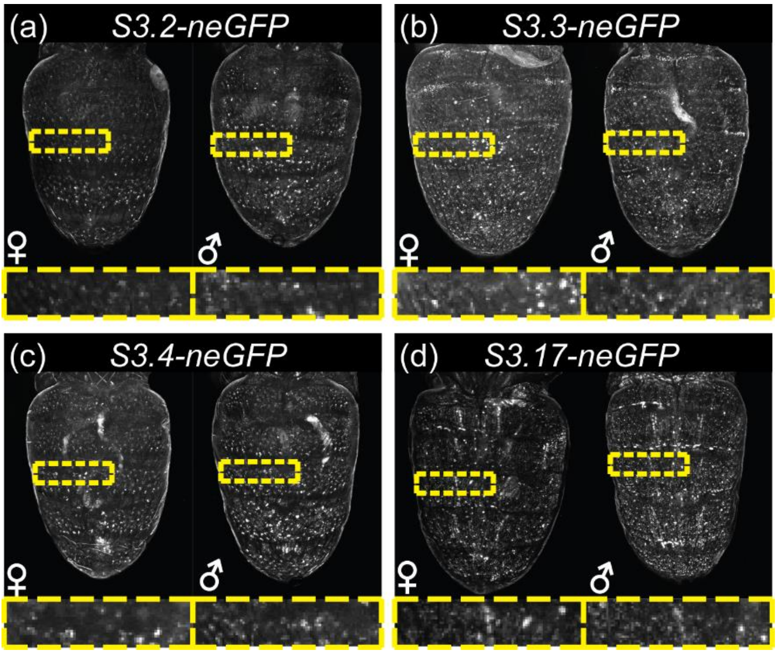

Supplementary Figure S6. neGFP expressions driven by *cis*-regulatory elements (CREs) within the *bric-à-brac* and *trio* locus. a-d) Representative *neGFP* reporter gene expressions in the dorsal abdomens of a female and male *D. melanogaster* pupae for the *S3.2-S3.4* and *S3.17* predicted CREs. Select (yellow dashed rectangles) abdomen regions were zoomed in on and provided below the full abdomen images to provide more detail on the neGFP expressions in epidermal cell nuclei. The developmental stage of specimens is between 80-95 hAPF. No noteworthy differences in neGFP expression were observed between female and male pupae.

Alt text: Subpanels a to d share Green Fluorescent Protein reporter expression patterns activated in male and female *Drosophila melanogaster* pupal abdomens by four sequences predicted to be *cis*-regulatory elements with abdomen activity. Each expression includes a regional zoom-in that provides a clearer visualization of the expressing cells.

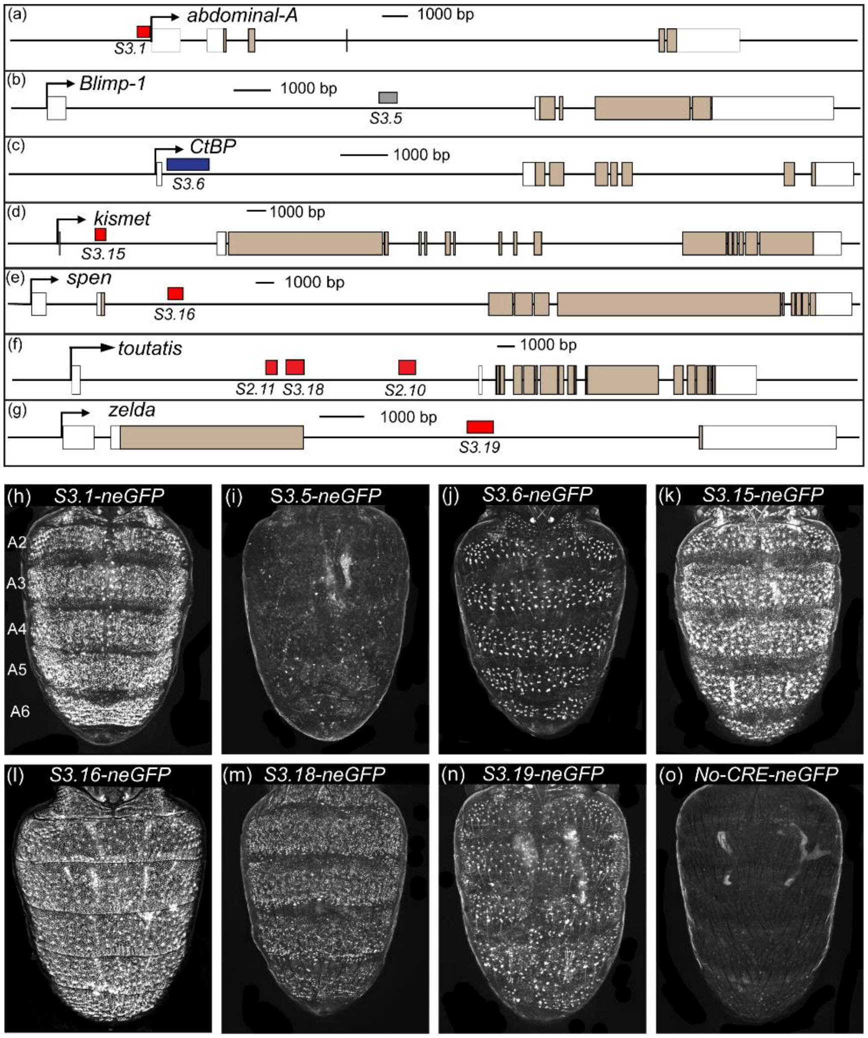

Supplementary Figure S7. neGFP expressions driven by predicted *cis*-regulatory elements (CREs) within or near to loci whose encoded proteins regulate gene expression. To scale models of a) *abdominal-A*, b) *Blimp-1*, c) *CtBP*, d) *kismet*, e) *split ends*, f) *toutatis*, and g) *zelda* loci. The *toutatis* locus *S2.10* and *S2.11* CREs are included here. These two CREs were previously shown to drive pupal abdomen epidermis expression of the *neGFP* reporter gene. CRE annotations colored in red, blue, and gray respectively indicate either abdomen epidermis, other cell type, or no noteworthy expression. h-o) neGFP expression in the dorsal abdomens of *D. melanogaster* pupae between 80-95 hAPF.

Alt text: Subpanels a to g share a to-scale representation of a *Drosophila melanogaster* gene with the positions annotated of the sequences that were predicted to be *cis*-regulatory elements. Subpanels h to n share Green Fluorescent Protein reporter transgene expressions in the pupal-stage abdomen that were activated by the suspected *cis*-regulatory elements, and subpanel o shows the limited reporter expression for a control specimen for which the reporter gene lacked a *cis*-regulatory element.

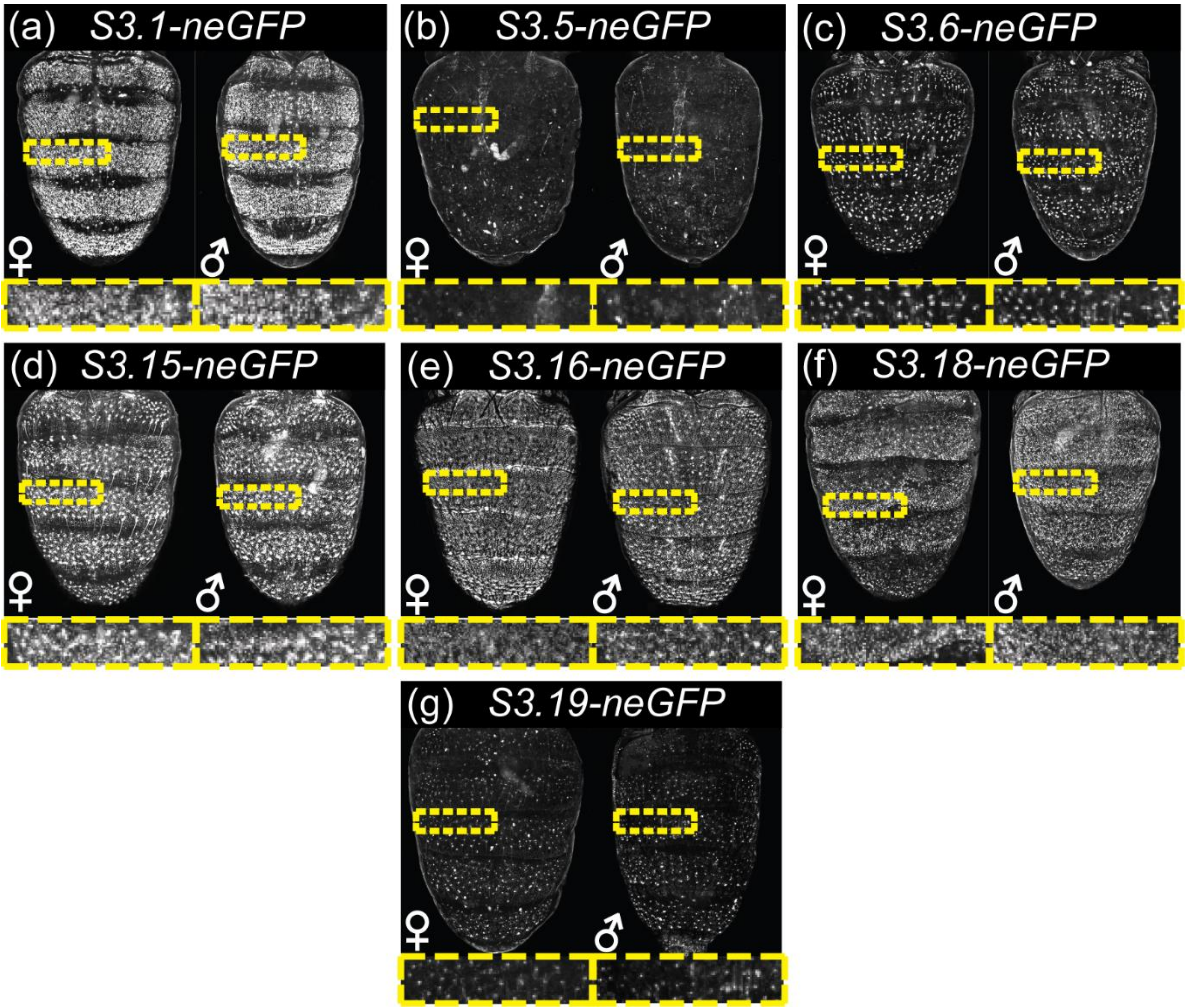

Supplementary Figure S8. neGFP expressions driven by *cis*-regulatory elements (CREs) within or near gene expression regulating loci. a-g) Representative *neGFP* reporter gene expressions in the dorsal abdomens of a female and male *D. melanogaster* pupae for several predicted CREs. Select (yellow dashed rectangles) abdomen regions were zoomed in on and provided below the full abdomen images to provide more detail on the neGFP expressions in epidermal cell nuclei. The developmental stage of specimens is between 80-95 hAPF. For all predicted CREs shown here, no noteworthy differences in expression were observed between female and male pupae.

Alt text: Subpanels a to g share Green Fluorescent Protein reporter expression patterns activated in male and female *Drosophila melanogaster* pupal abdomens by seven sequences predicted to be *cis*-regulatory elements with abdomen activity. Each expression includes a regional zoom-in that provides a clearer visualization of the expressing cells.

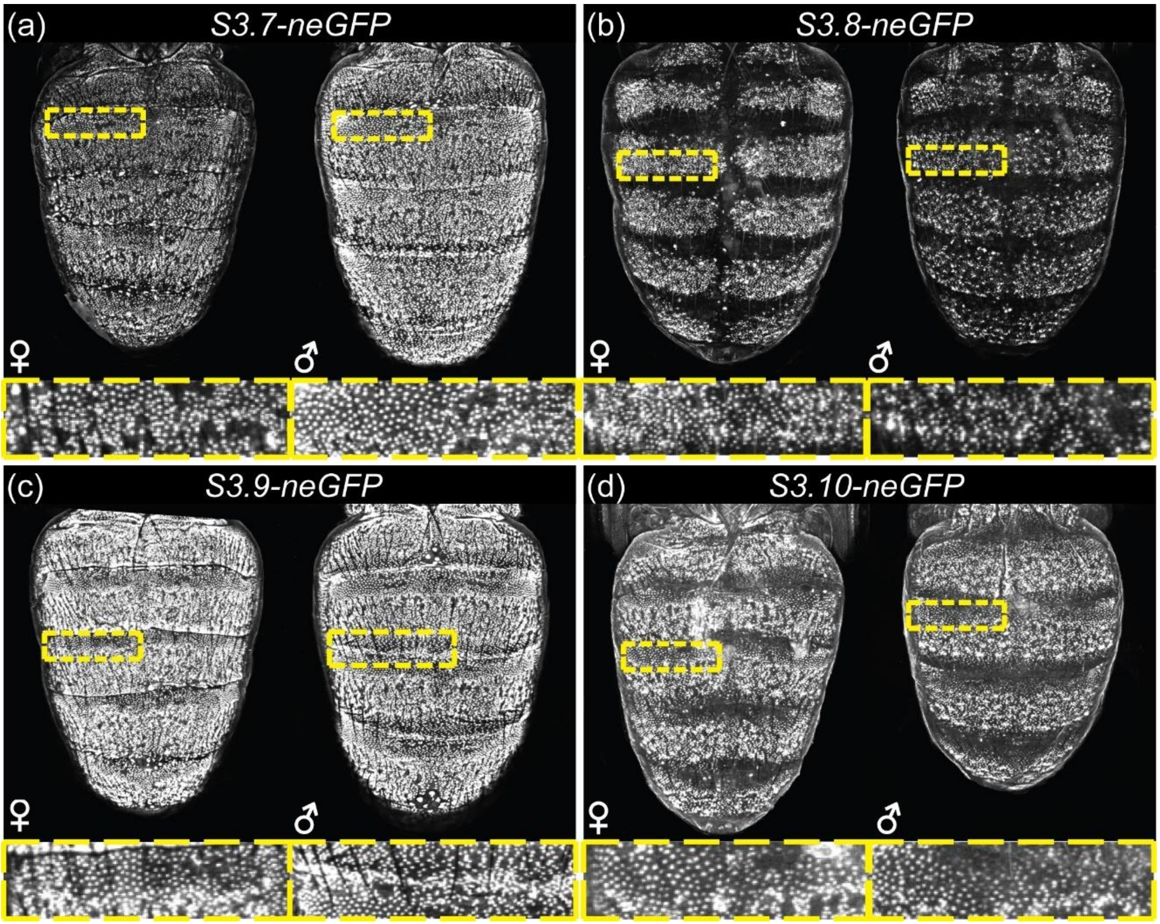

Supplementary Figure S9. Monomorphic neGFP expressions driven by *cis*-regulatory elements (CREs) within the *grainy head* locus. a-d) Representative *neGFP* reporter gene expressions in the dorsal abdomens of a female and male *D. melanogaster* pupae. The developmental stage of specimens is between 80-95 hAPF. Select (yellow dashed rectangles) abdomen regions were zoomed in on and provided below the full abdomen images to provide more detail on the neGFP expressions in epidermal cell nuclei.

Alt text: Subpanels a to d share Green Fluorescent Protein reporter expression patterns activated in male and female *Drosophila melanogaster* pupal abdomens by four sequences predicted to be *grh* gene *cis*-regulatory elements with abdomen activity. Each expression includes a regional zoom-in that provides a clearer visualization of the expressing cells.

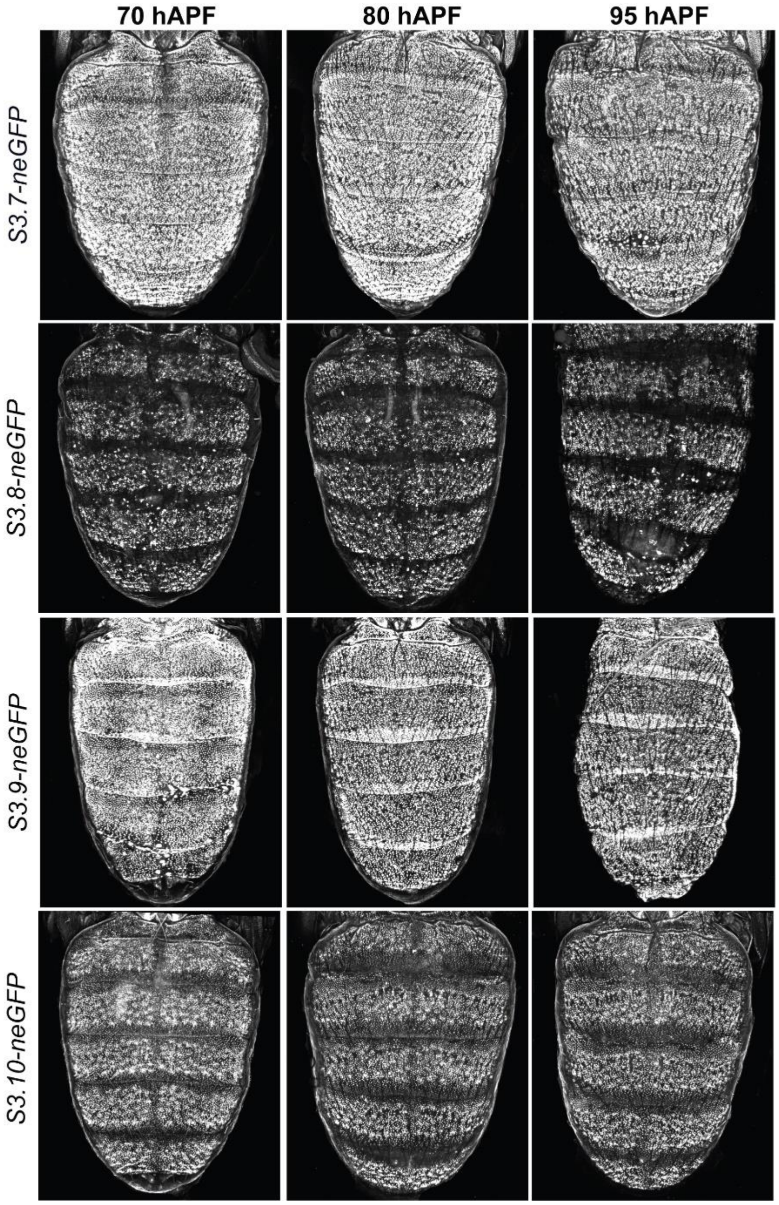

Supplementary Figure S10. The four *grh* locus CREs activate neGFP expression during the same critical time period when abdomen pigmentation is patterned. neGFP expressions in transgenic *D. melanogaster* driven by the *S3.7-S3.10* CREs were assessed at 70, 80, and 95 hAPF. For each CRE, similar patterns and levels of neGFP expression were observed at each time point.

Alt text: The images show Green Fluorescent Protein reporter expression patterns activated in transgenic *Drosophila melanogaster* abdomens at three different time points of pupal development. The four *grh* locus *cis*-regulatory elements activate expression at each timepoint.

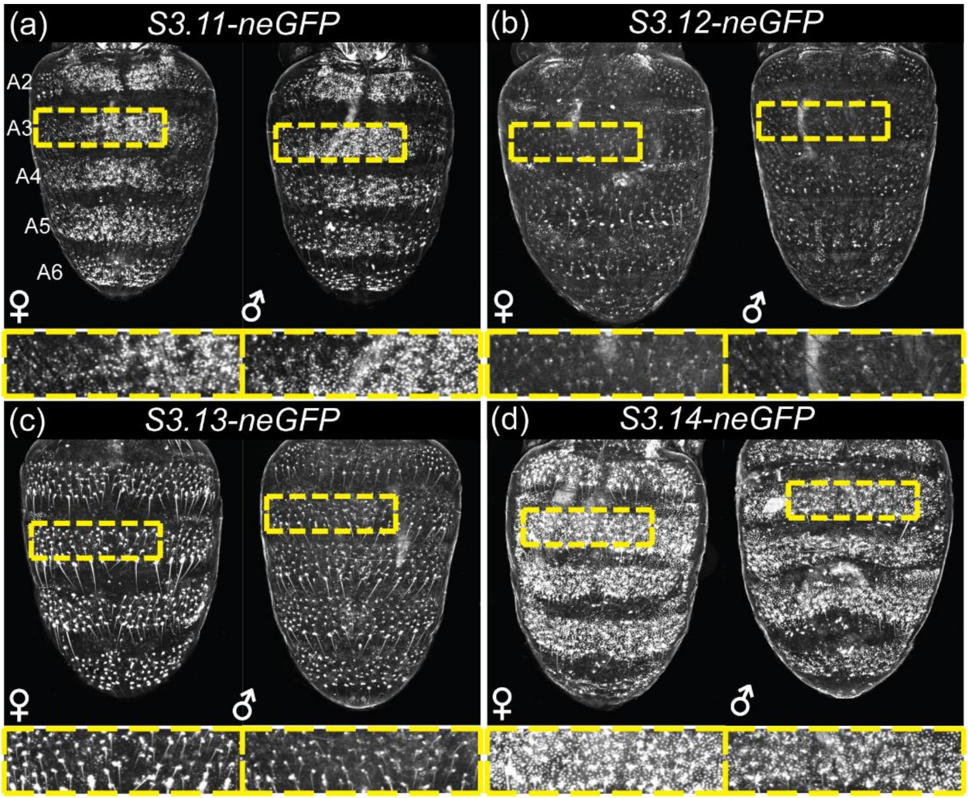

Supplementary Figure S11. Monomorphic neGFP expressions driven by the *S3.11* and *S3.14 hth* locus *cis*-regulatory elements (CREs). a-d) Representative neGFP reporter expressions in the dorsal abdomens of a female and male *D. melanogaster* pupae. The developmental stage of specimens is between 80-95 hAPF. Select (yellow dashed rectangles) abdomen regions were zoomed in on and provided below the full abdomen images to provide more detail on the neGFP expressions in epidermal cell nuclei.

Alt text: Subpanels a to d share Green Fluorescent Protein reporter expression patterns activated in male and female *Drosophila melanogaster* pupal abdomens by four sequences predicted to be *hth* gene *cis*-regulatory elements with abdomen activity. Each expression includes a regional zoom-in that provides a clearer visualization of the expressing cells.

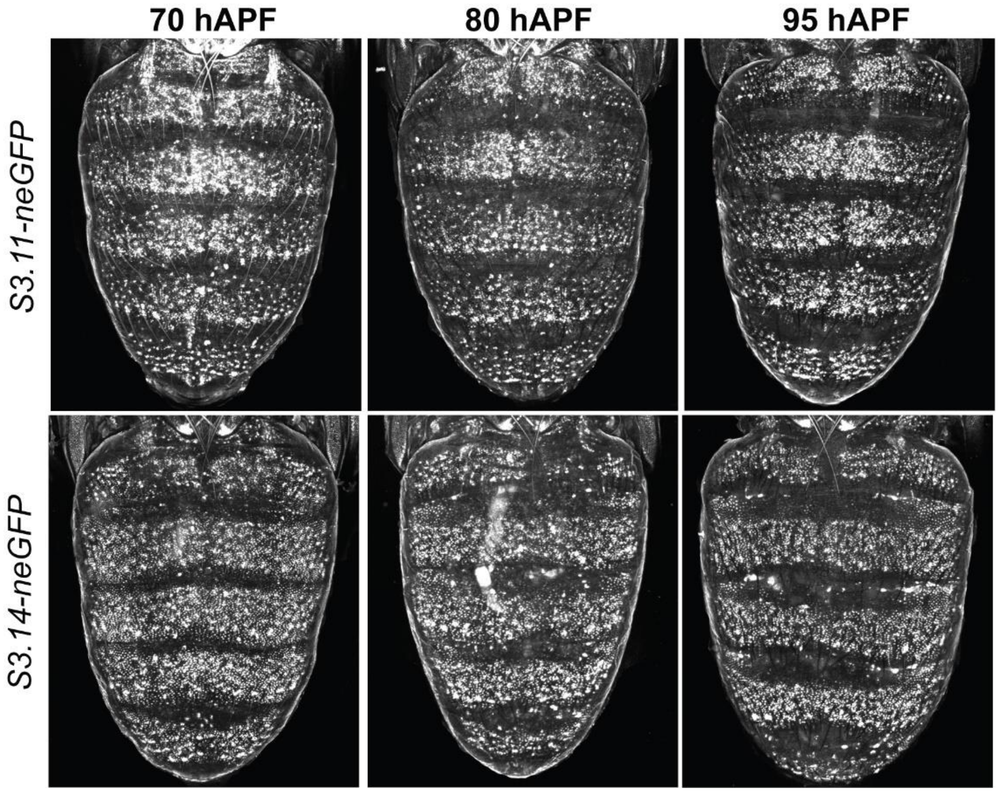

Supplementary Figure S12. The two *hth* locus CREs activate neGFP expression during the same critical time period when abdomen pigmentation is patterned. neGFP expressions in transgenic *D. melanogaster* driven by the *S3.11* and *S3.14* CREs were assessed at 70, 80, and 95 hAPF. For each CRE, similar patterns and levels of neGFP expression were observed at each time point.

Alt text: The images show Green Fluorescent Protein reporter expression patterns activated in transgenic *Drosophila melanogaster* abdomens at three different time points of pupal development. The two *hth* locus *cis*-regulatory elements activate expression at each timepoint.

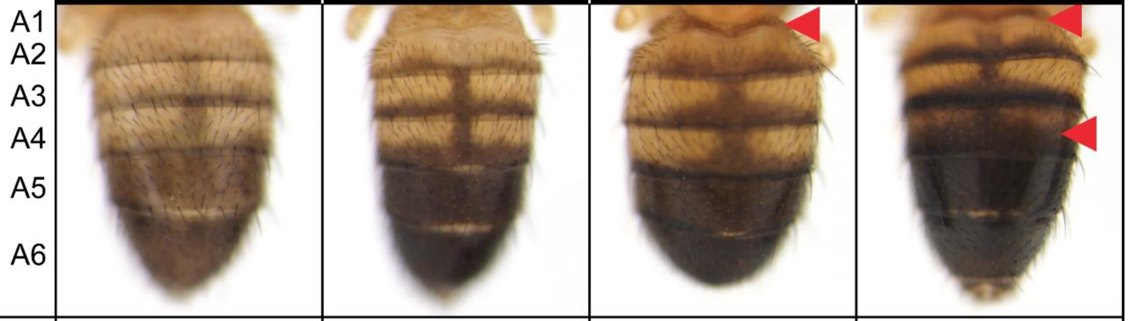

	Lower	Average	1 ectopic gain	2+ ectopic gains
+/+	7	17	4	1
ΔS3.14/ ΔS3.14	1	7	28	10
ΔS3.11/ ΔS3.14	0	18	10	17

Supplementary Figure S13. Incompletely penetrant and variably expressive pigmentation phenotypes. The abdomen tergite pigmentation phenotypes were scored for numerous adult flies with the wild type (+/+) *hth* genotype, homozygotes for $hth^{\Delta S3.14}$, and *trans*-heterozygous $hth^{\Delta S3.11/S3.14}$. We grouped pigmentation phenotypes into one of four categories. Pigmentation lower than typical, average, one ectopic aspect such as an A1 posterior stripe of pigmentation, and two or more aspects of ectopic pigmentation like an A1 stripe and excessive A4 tergite pigmentation. While the wild type individuals had phenotypes skewed towards lower and average, the *hth* CRE deletion mutants had phenotypes skewed towards ectopic pigmentation.

Alt text: The images and data show the distribution of melanic pigmentation phenotypes between flies with differing *hth* CRE genotypes. Notably, male flies with a homozygous deletion of the *S3.14* CRE or males with the *S3.11* CRE deleted from one *hth* allele and deletion of the *S3.14* CRE from the other allele frequently had gains in the extent of melanic pigmentation.

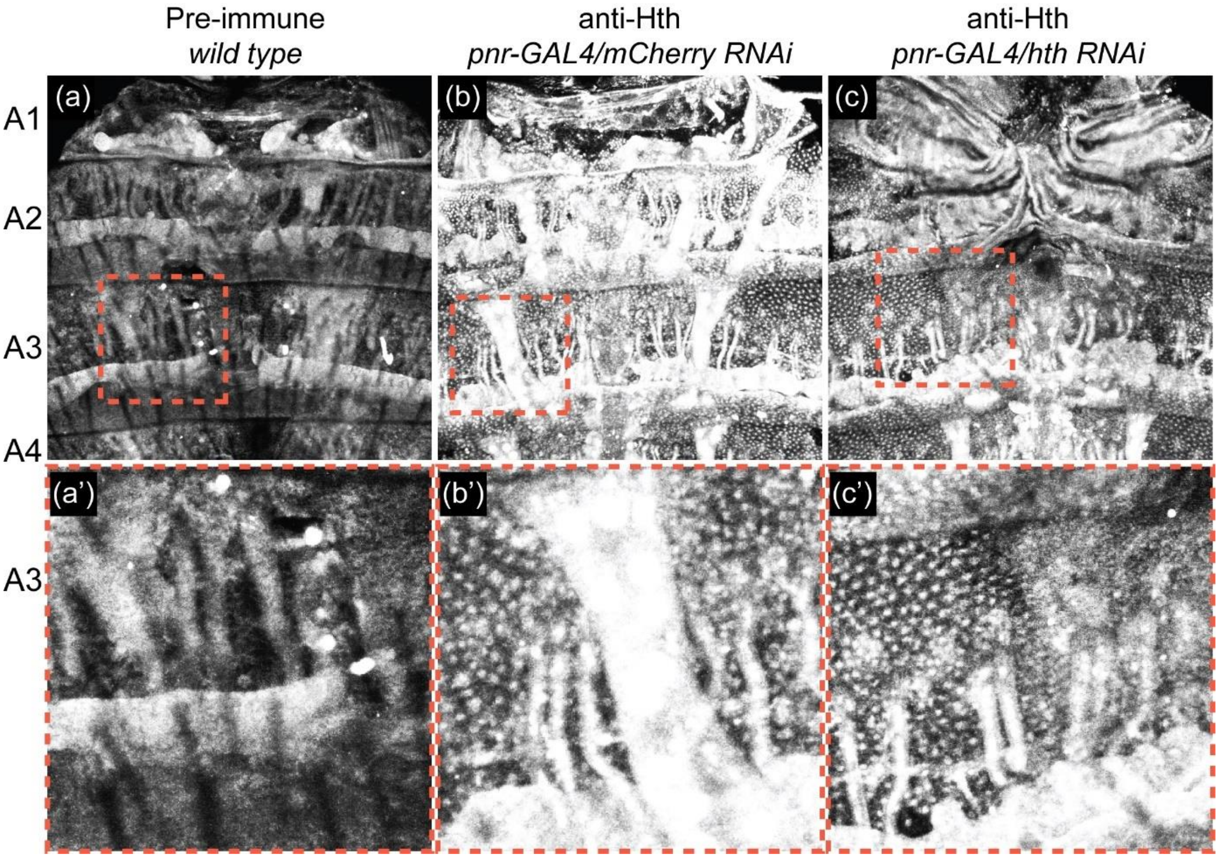

Supplementary Figure S14 Figure. Rabbit anti-Homothorax antibody detects nucleus-localized Hth in *Drosophila melanogaster* pupa. Dorsal abdomen tissue sections of *Drosophila melanogaster* pupae at the 88 hAPF stage. a and a') *w1118* genetic background treated with pre-immune IgG as the primary antibody. Genetic background expressing a UAS-RNA interference (*RNAi*) transgene targeting b and b') *mCherry* and c and c') *hth* in the dorsal midline region where GAL4 is expressed in the domain of the *pnr* gene. Red dashed boxes in A-C are zoomed in on in a'-c'. The RNAi expressing region is the right half of b' and c', and non-expressing on the left. b') While Hth is expressed evenly through the sample where *mCherry* was targeted by RNAi, c') Hth expression was notably reduced in the *hth* RNAi expressing region.

Alt text: The images show nucleus-localized Hth in control and wild type pupal abdomen epidermis sections. Notably, when *hth* was targeted by RNAi in the abdomen midline region, there was a visible reduction in detected Hth in this region compared to the lateral control tissue where RNAi did not occur.

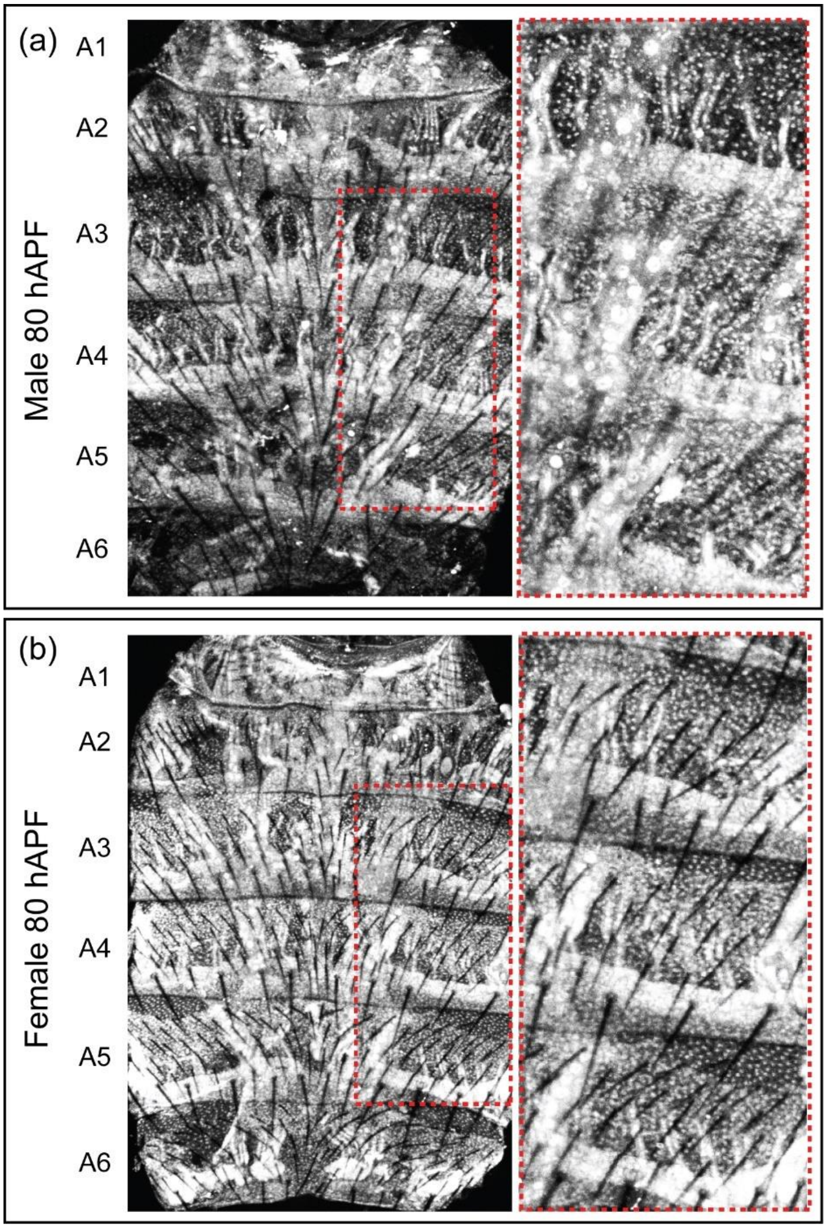

Supplementary Figure S15. Homothorax expression in the dorsal abdomen epidermis is monomorphic for *Drosophila melanogaster*. Hth expression as seen by immunohistochemistry in *D. melanogaster* a) male and b) female. The dashed red rectangle indicates a select region of the dorsal A3-A5 segments that is shown zoomed in on the right.

Alt text: The images are of immunohistochemistry data that shows Hth protein to be expressed similarly between *Drosophila melanogaster* males and females in the dorsal pupal abdomen epidermis.

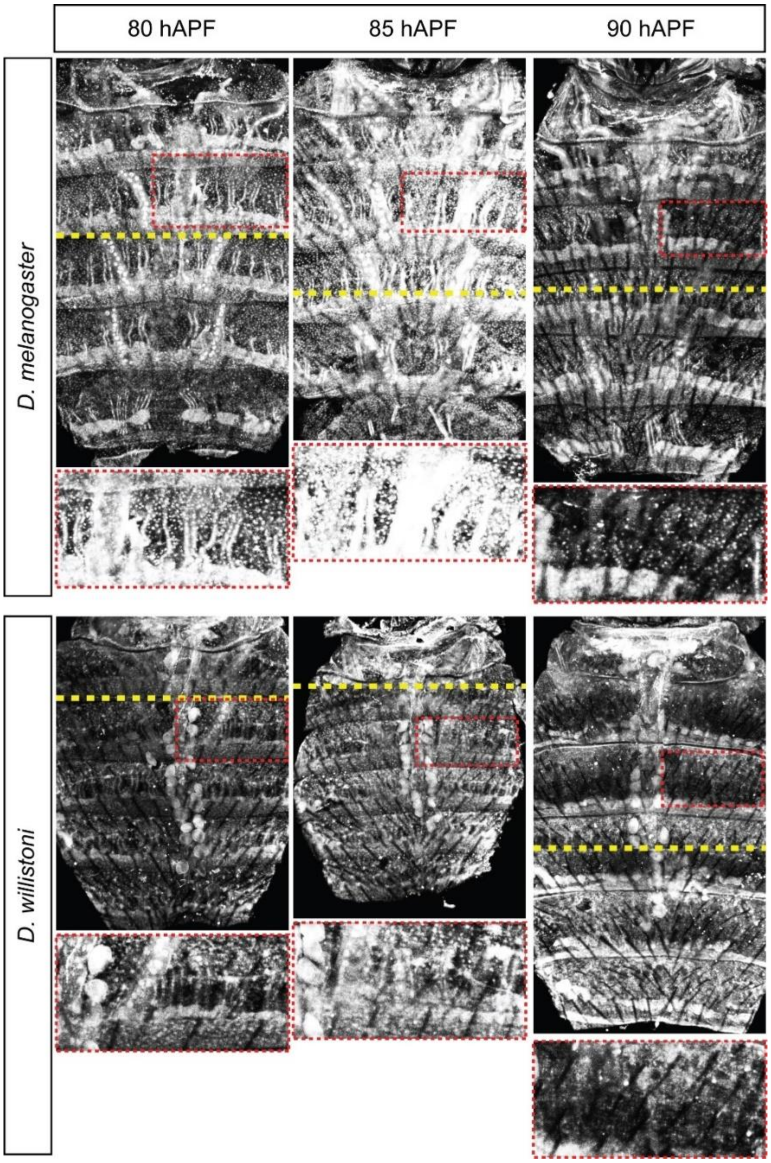

Supplementary Figure S16. Hth expression persists into later development stages in the abdomen epidermis of *D. melanogaster* but not *D. willistoni*. Hth expression in dorsal sections of specimens at the developmental stage equivalent to 80, 85, and 90 hAPF. Abdomens shown are composites from two separate images and the dashed yellow line specifies where the two images were merged. Representative sections in the A3 abdomen segment were selected from each specimen, outlined by dashed red boxes, and provided below as a zoomed-in image. Robust epidermis Hth expression was observed at the 80 and 85 hAPF stages of *D. melanogaster*, and modest expression remained at 90 hAPF. In contrast, Hth expression was notably reduced in *D. willistoni* at these same stages.

Alt text: The images are of immunohistochemistry data that shows Hth protein expression at three pupal timepoints for *Drosophila melanogaster* and *Drosophila willistoni*.

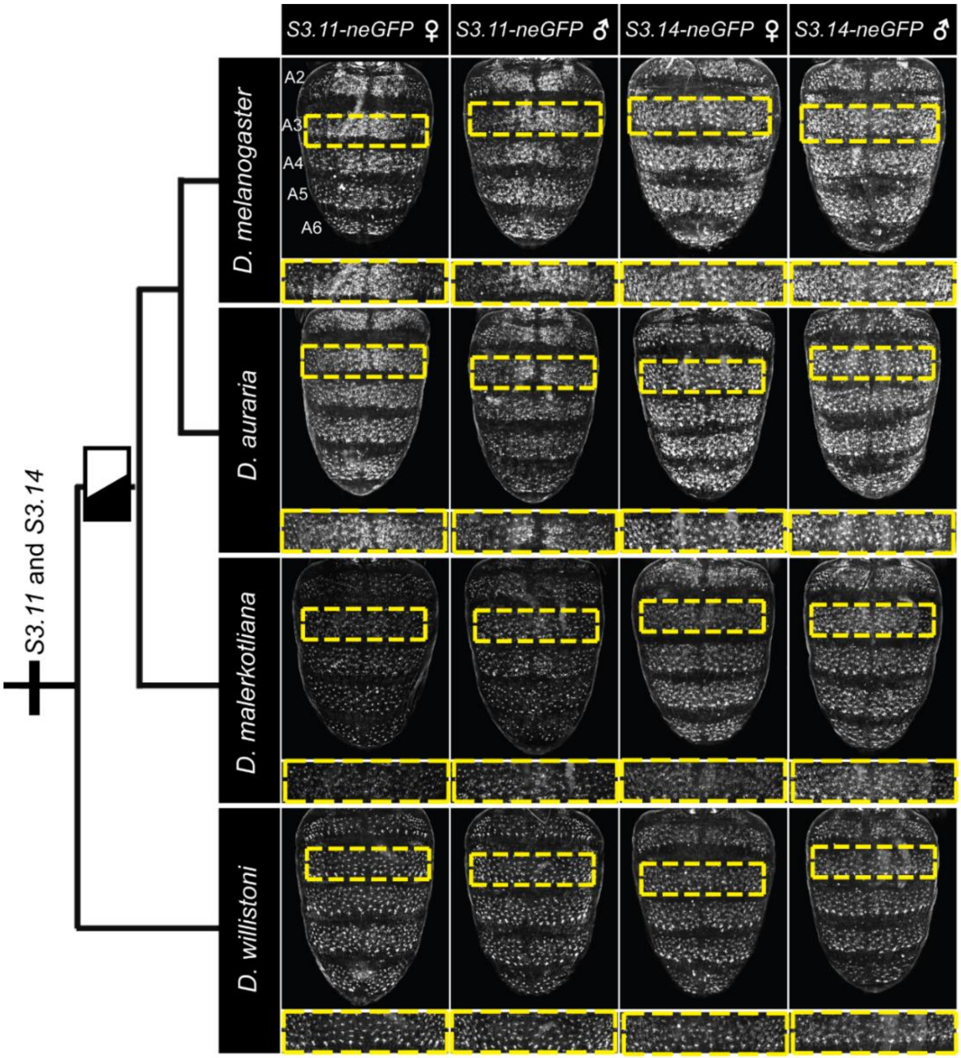

Supplementary Figure S17. The *S3.11* and *S3.14* CREs predate the origin of the derived dimorphic pigmentation trait. neGFP expressions in representative male and female pupae driven by the *D. melanogaster S3.11* and *S3.14* CREs and the orthologous sequences from *D. auraria*, *D. malerkotliana*, and *D. willistoni*. All transgenes are situated in the 51-D site of transgenic *D. melanogaster*, and specimens shown here are at the ~88 hAPF stage. The black/white bar on the phylogeny indicates the origin of the male-specific pattern of tergite pigmentation. Select (yellow dashed rectangles) abdomen regions were zoomed in on and provided below the full abdomen images to provide more detail on the neGFP expressions in epidermal cell nuclei.

Alt text: The figure shares Green Fluorescent Protein reporter expression patterns activated in female and male *Drosophila melanogaster* pupal abdomens driven by the comparable sequences from species with differing patterns of abdomen tergite pigmentation. A phylogeny is provided to show the evolutionary relatedness of the species whose *hth* CREs were evaluated here.

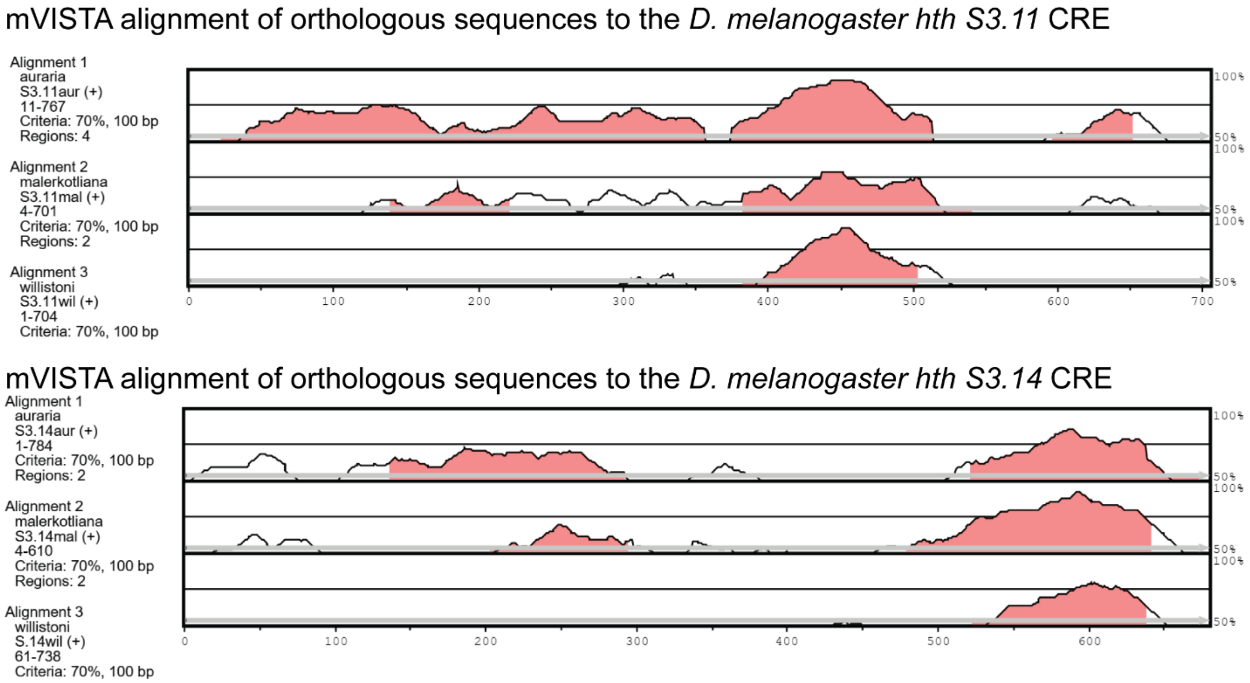

Supplementary Figure S18. Conservation and divergence of the sequences orthologous to the *D. melanogaster hth S3.11* and *S3.14* CREs. mVISTA output of sequence alignments of the orthologous sequences to the *homothorax S3.11* and *S3.14* of *D. melanogaster*. The orthologous sequences were obtained from the genome sequences available for *D. auraria*, *D. malerkotliana*, and *D. willistoni*. Sequence conservation is annotated as peaks exceeding 50% identity to the *melanogaster* sequence. Conserved non-coding sequences are annotated as salmon-colored peaks for which sequence identity is 70% or greater for 100 base pairs or more.

Alt text: mVISTA plot of the alignment of sequences orthologous to the *Drosophila melanogaster hth S3.11* and *S3.14* CREs. The peaks show regions where CRE sequences can be considered conserved.

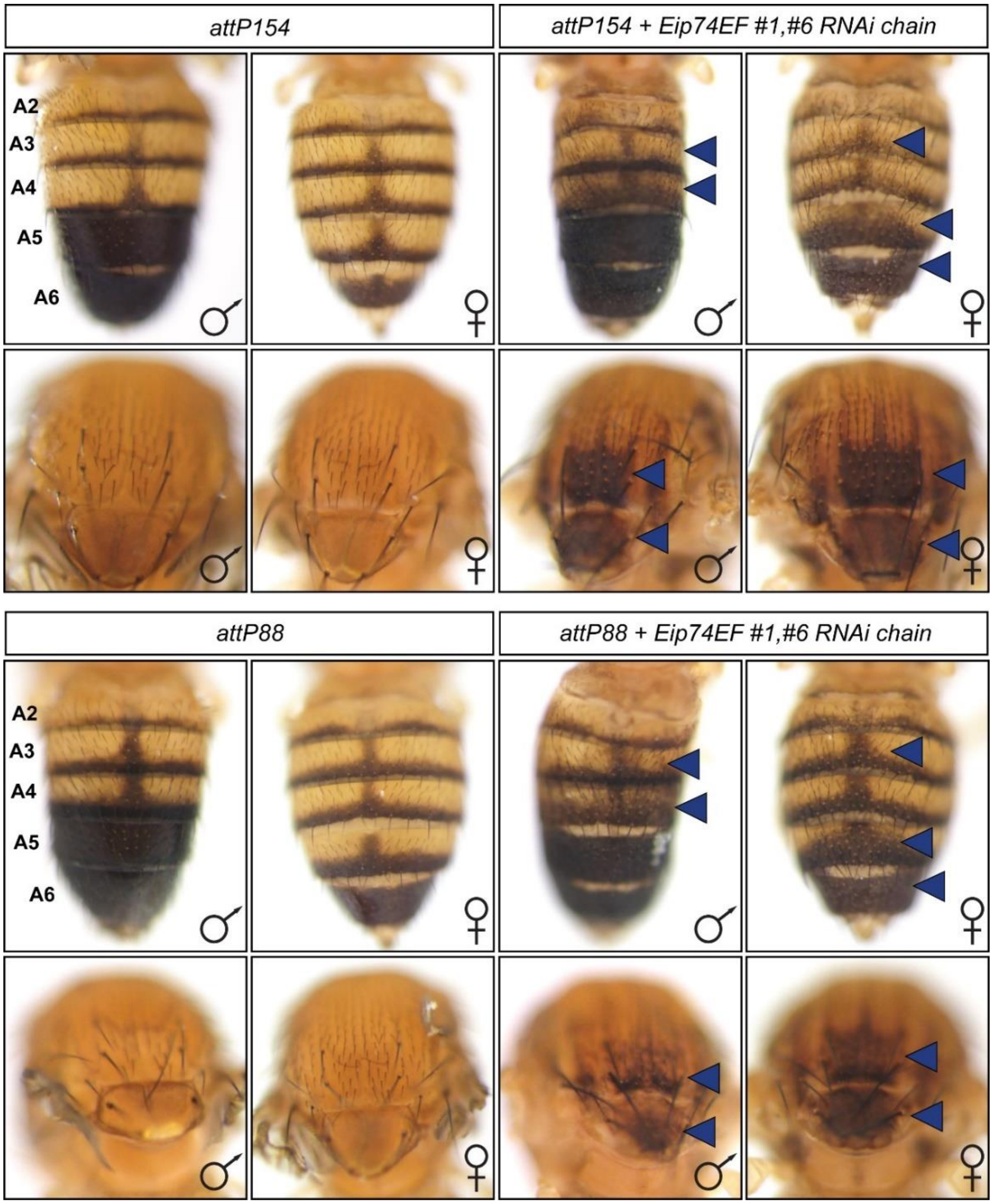

Supplementary Figure S19. Ectopic melanic pigmentation in specimens with *Eip74EF* RNA interference. The male and female pigmentation phenotypes are shown for the *att*P154 and *att*P88 *Drosophila melanogaster* stocks. Leaky expression of an RNAi transgene, called *Eip74EF #1,#6*, from these *att*P landing sites resulted in ectopic melanic pigmentation on several abdomen tergites and the notum. The samples shown here were at 4-5 days of adult age.

Alt text: RNA-interference for the *Eip74EF* gene resulted in ectopic melanic pigmentation in the abdomen and thorax of transgenic *Drosophila melanogaster*. Compared to the non-transgenic flies, left half images, leaky expression of an *Eip74EF* RNAi transgene caused gains in melanic pigmentation (right half images) in both males and females.

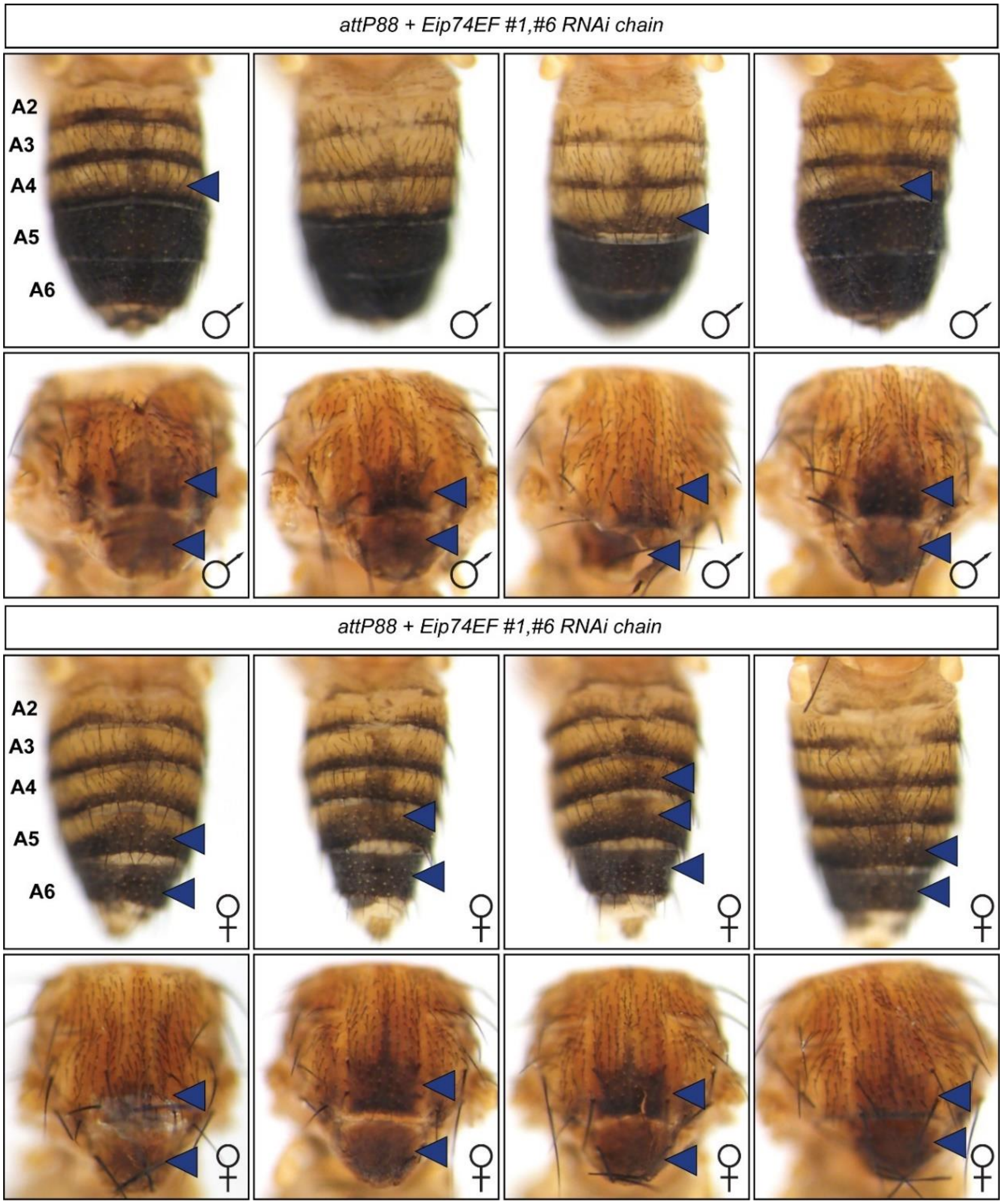

Supplementary Figure S20. Ectopic melanic pigmentation in replicate specimens with *Eip74EF* RNA interference. The male and female pigmentation phenotypes are shown for the *att*P88 *Drosophila melanogaster* stocks. Leaky expression of the *Eip74EF #1,#6* RNAi transgene from this *att*P landing sites resulted in consistent ectopic melanic pigmentation on several abdomen tergites and the notum. The samples shown here were at 4-5 days of adult age.

Alt text: RNA-interference for the *Eip74EF* gene resulted in consistent aberrations in abdomen and thorax pigmentation.

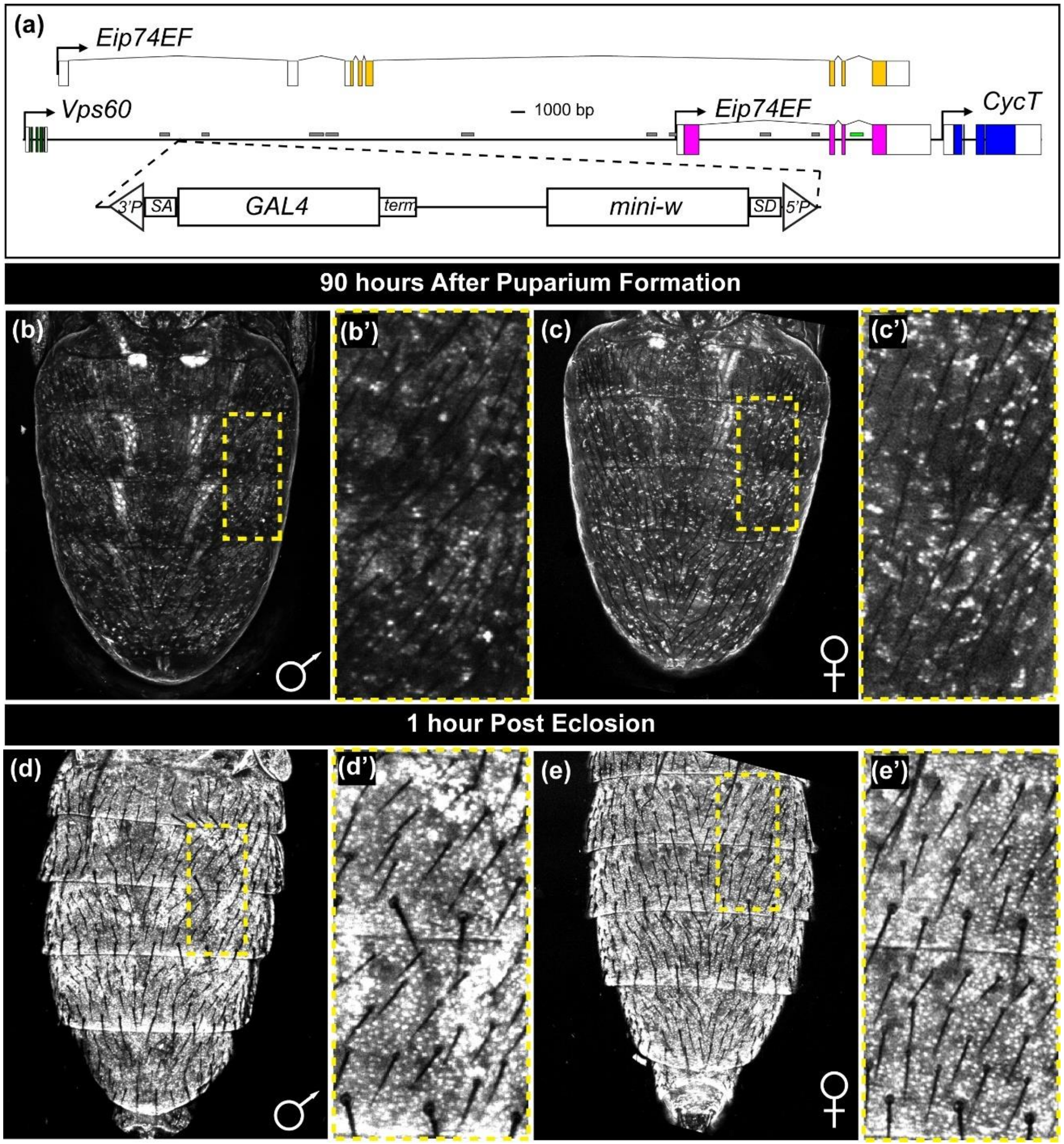

Supplementary Figure S21. *Eip74EF* expression is activated around the time point when pupae eclose from the pupal-stage puparium. a) To scale representation of the *Eip74EF* locus with its two promoters (rightward pointing arrows) and transcribed exon sequences that are spliced together into two alternate transcripts. The positions of the locus' 10 predicted CREs are indicated by the small and short colored rectangles. The position and contents of the inserted gene trap vector sequence is shown, which includes a splice acceptor (SA) adjacent to the coding sequence for *GAL4*, and the *mini-white* transgene sequence that is followed by a splice donor (SD) sequence. b-e) neGFP expression in the pattern of *Eip74EF* regulation in the dorsal abdomens and b'-e') zoomed in expression seen in select abdomen regions. At 90 hAPF expression can be seen in some abdomen muscles of b & b') male and c & c') female specimens. Just after eclosion, neGFP expression is robustly seen throughout the dorsal abdomen epidermis of d & d') male and e & e') female specimens.

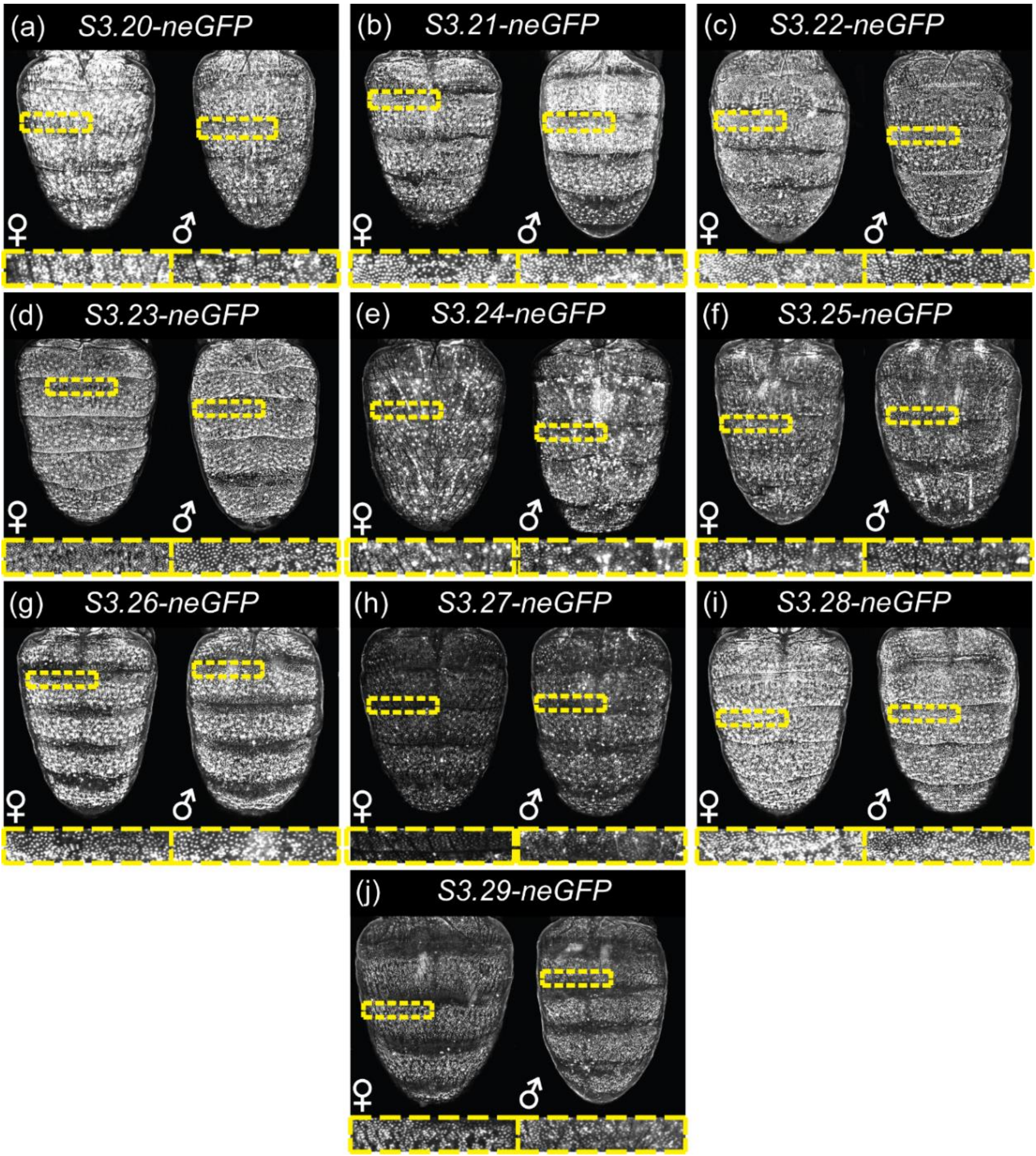

Supplementary Figure S22. Monomorphic neGFP expressions driven by the *S3.20-S3.29 Eip74EF* locus *cis*-regulatory elements (CREs). a-j) Representative neGFP reporter expressions in the dorsal abdomens of a female and male *D. melanogaster* pupae. The developmental stage of specimens is between 80-95 hAPF. Select (yellow dashed rectangles) abdomen regions were zoomed in on and provided below the full abdomen images to provide more detail on the neGFP expressions in epidermal cell nuclei.

Alt text: Subpanels a to j share Green Fluorescent Protein reporter expression patterns activated in male and female *Drosophila melanogaster* pupal abdomens by ten sequences predicted to be *Eip74EF* gene *cis*-regulatory elements with abdomen activity. Each expression includes a regional zoom-in that provides a clearer visualization of the expressing cells.

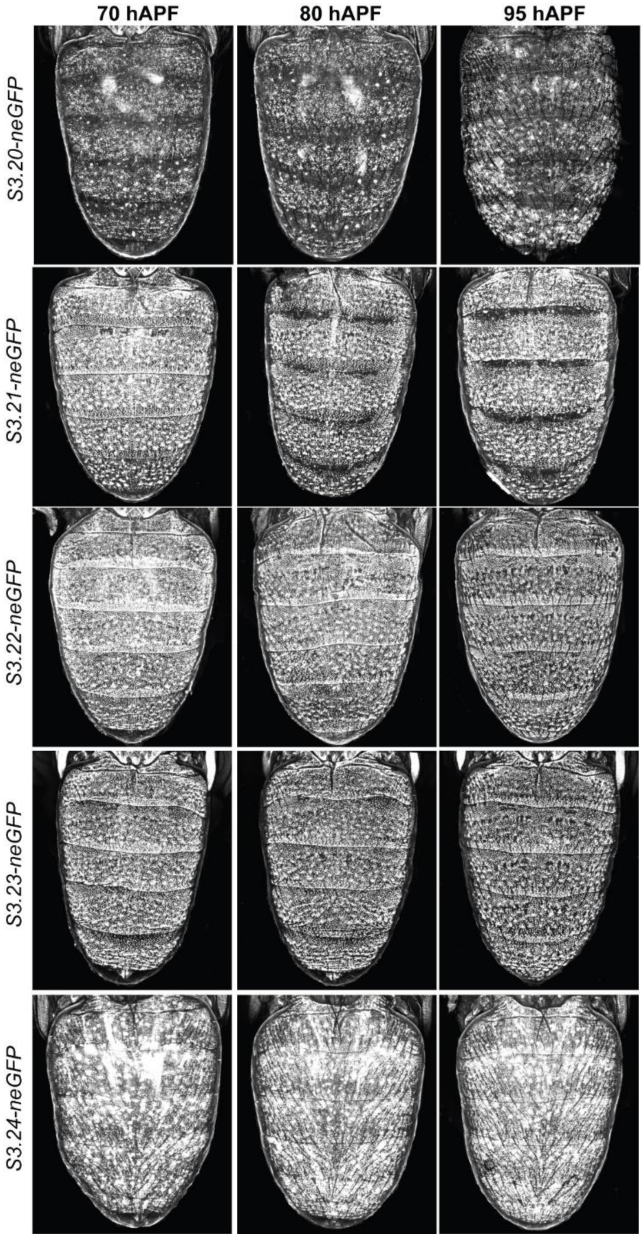

Supplementary Figure S23. The *Eip74EF* locus *S3.20-S3.24* CREs activate neGFP expression during the same critical time period when abdomen pigmentation is patterned. neGFP expressions in transgenic *D. melanogaster* driven by the *S3.20-S3.24* CREs were assessed at 70, 80, and 95 hAPF. For each CRE, similar patterns and levels of neGFP expression were observed at each time point.

Alt text: The images show Green Fluorescent Protein reporter expression patterns activated in transgenic *Drosophila melanogaster* abdomens at three different time points of pupal development. The five *Eip74EF* locus CREs shown activate expression at each timepoint.

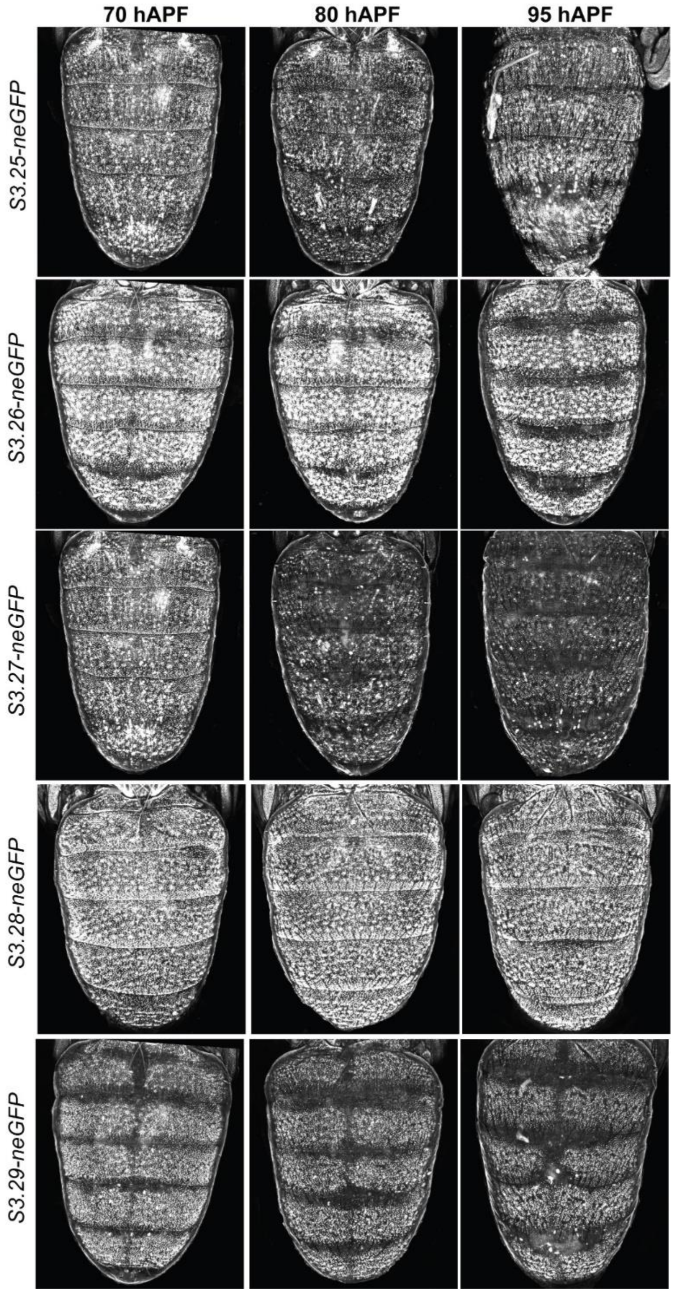

Supplementary Figure S24. The *Eip74EF* locus *S3.25-S3.29* CREs activate neGFP expression during the same critical time period when abdomen pigmentation is patterned. neGFP expressions in transgenic *D. melanogaster* driven by the *S3.25-S3.29* CREs were assessed at 70, 80, and 95 hAPF. For each CRE, similar patterns and levels of neGFP expression were observed at each time point.

Alt text: The images show Green Fluorescent Protein reporter expression patterns activated in transgenic *Drosophila melanogaster* abdomens at three different time points of pupal development. The five *Eip74EF* locus CREs shown activate expression at each timepoint.

(a)

	S3.20	*S3.21*	*S3.22*	*S3.23*	*S3.24*	*S3.25*	*S3.26*	*S3.27*	*S3.28*	*S3.29*
S3.20		<40	<40	<40	<40	NSS	<40	NSS	<40	<40
S3.21			NSS	<40	<40	<40	<40	<40	<40	<40
S3.22				50-80	<40	NSS	NSS	NSS	NSS	<40
S3.23					NSS	NSS	NSS	NSS	NSS	<40
S3.24						<40	<40	<40	<40	<40
S3.25							NSS	<40	NSS	<40
S3.26								<40	<40	<40
S3.27									<40	<40
S3.28										<40
S3.29										

BLAST Alignment Scores

(green) = 50-80

(blue) = 40-50

(black) = <40

NSS = No Significant Similarity

(b)

```
Score           Expect   Identities     Gaps       Strand
52.7 bits(57)   2e-10    40/47(85%)     3/47(6%)   Plus/Minus

Query  262  GCAATACCAACAGCAAAA---GCAACAACAACAACAACAGCAACAGC  305
            |||| ||||||||||  |    ||||||||||||||||||||| ||
Sbjct  400  GCAACACCAACAGCACCATTTGCAACAACAACAACAACAGCAACTGC  354
```

```
Score           Expect   Identities     Gaps       Strand
41.9 bits(45)   4e-07    42/55(76%)     0/55(0%)   Plus/Plus

Query  101  TTGTTGTTGTTGTTGTTGCGCAGCTCTTTCGAGTTTTCTTTCGCTTTTTCTTTTC  155
            ||||||||||||||||||    ||   ||||| ||||| ||    | ||| | |||||
Sbjct  190  TTGTTGTTGTTGTTGTTGGCGAGTGCTTTCAAGTTTGCTGCAGTTTTATTTTTTC  244
```

Supplementary Figure S25. BLAST comparison of *D. melanogaster Eip74EF* CRE sequences for compelling sequence similarity. (a) Graphical representation of the BLAST outcomes for pairwise comparisons of CRE sequences. Color coding was used to represent the highest alignment score observed for a pairwise comparison. (b) The alignments of the most compelling similarities seen between the non-homologous *S3.22* and *S3.23* CRE sequences. These alignments are between what can be considered low-complexity sequences.

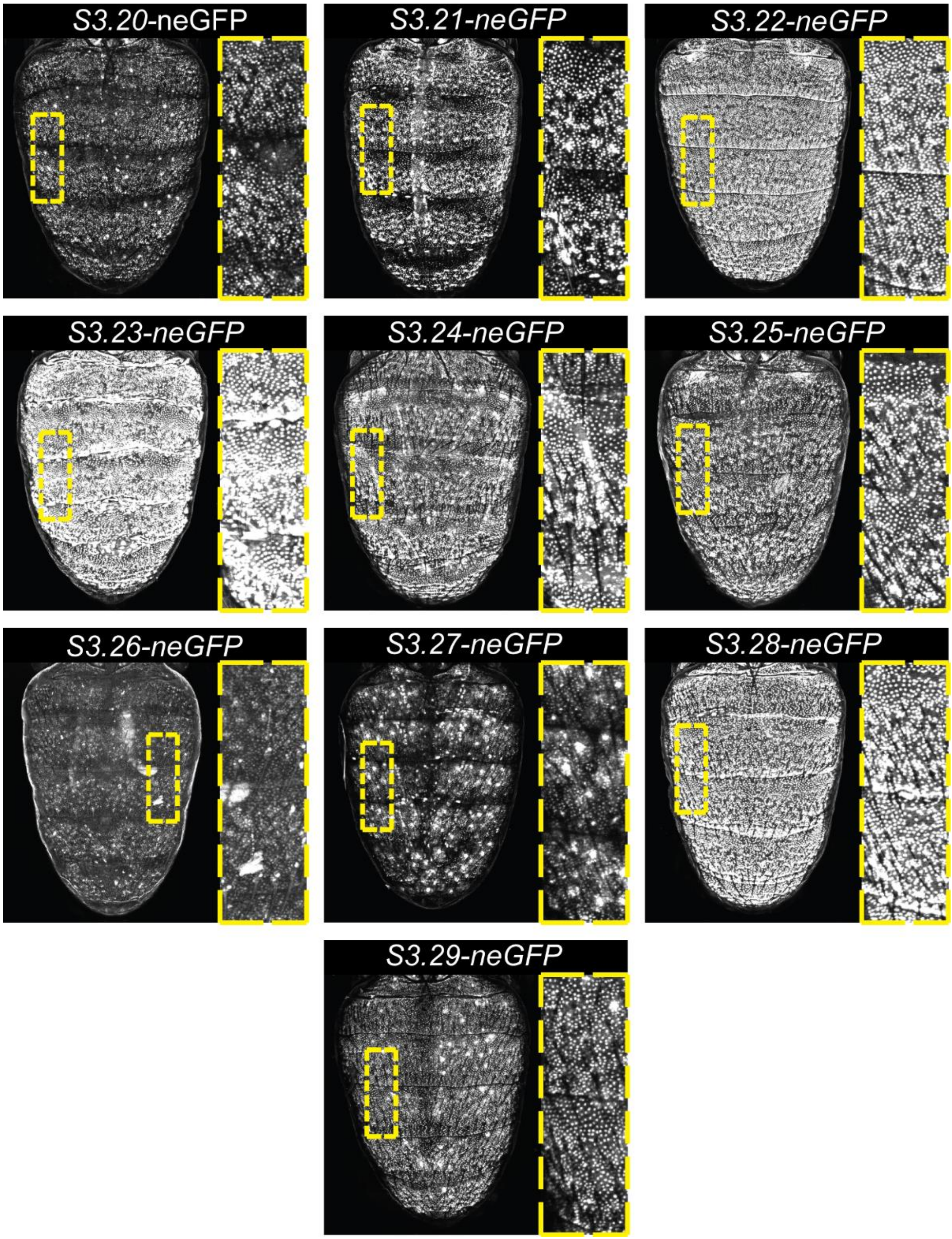

Supplementary Figure S26. Monomorphic neGFP expressions driven by the *S3.20-S3.29 Eip74EF* locus *cis*-regulatory elements (CREs) of *D. willistoni*. Representative neGFP reporter expressions in the dorsal abdomens of a *D. melanogaster* pupae. The developmental stage of specimens is between 80-95 hAPF. Select (yellow dashed rectangles) abdomen regions were zoomed in on and provided to the right of the full abdomen images to provide more detail on the neGFP expressions in epidermal cell nuclei.

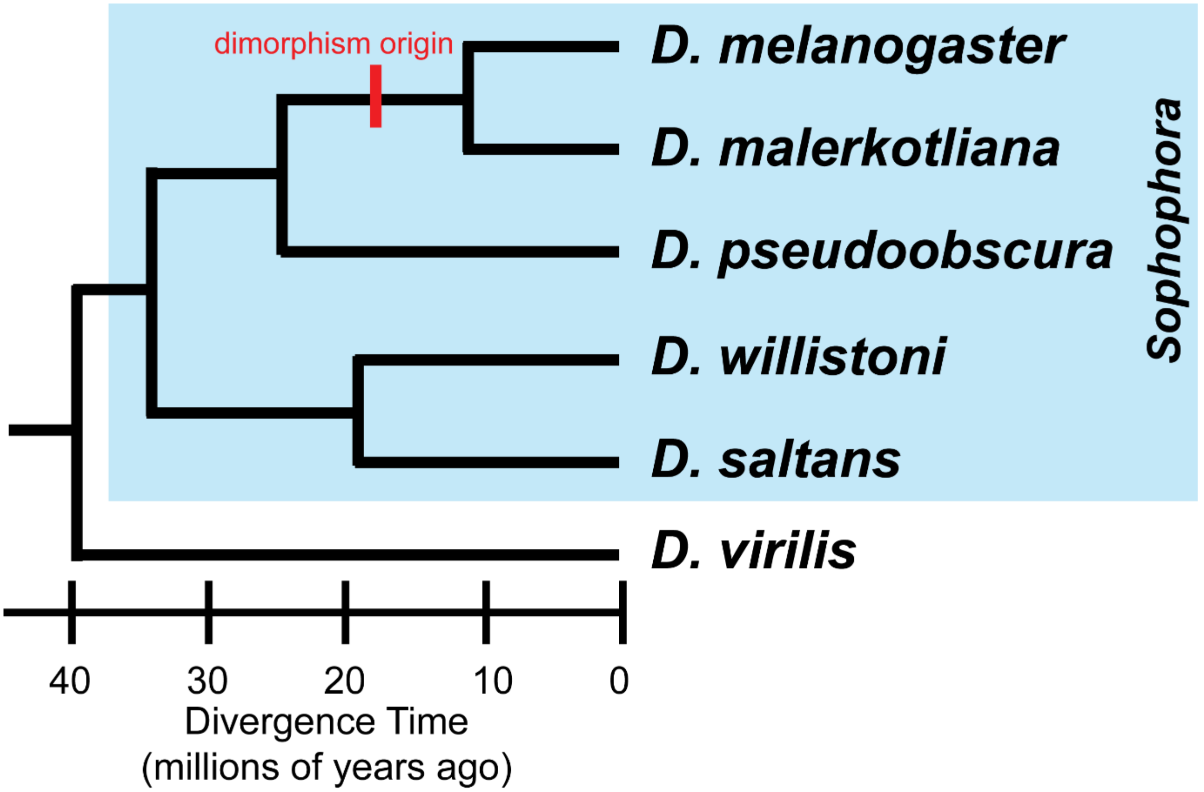

Supplementary Figure S27. Phylogeny of the species whose *grh*, *hth*, and *Eip74EF* loci were compared by mVISTA. The origin of the dimorphic abdomen pigmentation trait is annotated on the phylogeny as a red vertical bar. The blue background encloses the studied species that are considered part of the *Sophophora* subgenus of fruit flies.

Supplementary Table S1. Sequences targeted by gRNAs for CRISPR/Cas9 deletion of *homothorax* locus CREs.

Gene	CRE	target name	CRISPR target sequence (PAM)
homothorax	*S3.11*	S3.11 gRNA1	TTGAGAGTCATTGGAGTTTA (AGG)
homothorax	*S3.11*	S3.11 gRNA2	ATTAGTGAAACGCTTAAGTG (TGG)
homothorax	*S3.14*	S3.14 gRNA1	GAAGGAATACACTTAGCTAG (AGG)
homothorax	*S3.14*	S3.14 gRNA 2	GGGCGGGTGAATGTAAGGCT (GGG)

Supplementary Table S2. Appraisal of the sequence identity between the Conserved Non-coding Sequences within the orthologous sequences to the *D. melanogaster grh*, *hth*, and *Eip74EF* CREs.

D. melanogaster	***D. malerkotliana***		***D. pseudoobscura***		***D. willistoni***		***D. saltans***		***D. virilis***	
cis-regulatory element or CRE name (gene locus)	Total CNS length	CNS Identity %	Total CNS length	CNS Identity %	Total CNS length	CNS Identity %	Total CNS length	CNS Identity %	Total CNS length	CNS Identit y %
S3.7 (grh)	519 bp	85.9%	501 bp	78.8%	437 bp	80.3%	321 bp	86.3%	446 bp	79.1%
S3.8 (grh)	443 bp	76.1%	100 bp	83.0%	196 bp	80.1%	150 bp	83.3%	179 bp	71.5%
S3.9 (grh)	985 bp	74.7%	385 bp	84.7%	259 bp	84.9%	195 bp	84.1%	155 bp	73.5%
S3.10 (grh)	712 bp	75.0%	316 bp	73.4%	None	N/A	None	N/A	263 bp	71.1%
S3.11 (hth)	391 bp	73.9%	253 bp	77.5%	127 bp	86.6%	180 bp	87.8%	127 bp	85.8%
S3.14 (hth)	300 bp	76.3%	191 bp	77.5%	119 bp	74.8%	118 bp	78.8%	119	73.1%
S3.20 (Eip74EF)	459 bp	79.3%	310 bp	82.3%	307 bp	78.8%	304 bp	80.9%	245 bp	87.3%
S3.21 (Eip74EF)	521 bp	71.2%	310 bp	65.5%	229 bp	76.0%	116 bp	80.2%	107 bp	93.5%
S3.22 (Eip74EF)	252 bp	79.8%	168 bp	80.4%	100 bp	75.0%	None	N/A	124 bp	74.2%
S3.23 (Eip74EF)	599 bp	80.5%	634 bp	74.3%	269 bp	75.8%	233 bp	70.0%	None	N/A
S3.24 (Eip74EF)	203 bp	72.4%	182 bp	86.8%	249 bp	75.1%	232 bp	79.3%	192	77.1%
S3.25 (Eip74EF)	404 bp	79.0%	450 bp	70.9%	None	N/A	226 bp	73.5%	130 bp	76.2%
S3.26 (Eip74EF)	315 bp	73.7%	100 bp	82.0%	None	N/A	None	N/A	None	N/A
S3.27 (Eip74EF)	216 bp	90.3%	281 bp	74.4%	293 bp	70.3%	376 bp	72.3%	262 bp	72.1%
S3.28 (EiP74EF)	237 bp	75.5%	240 bp	77.1%	210 bp	77.6%	354 bp	78.2%	1292 bp	76.7%
S3.29 (Eip74EF)	457 bp	73.7%	185 bp	84.9%	254 bp	72.8%	383 bp	73.1%	346 bp	74.9%

Notes: Here, conserved Non-coding Sequences (CNS) are sequences of 100 base pairs or more with a sequence identity greater than or equal to 70%. Total CNS length is the combined length for all CNS within the orthologous sequence for a *D. melanogaster* CRE. CNS Identity% is the percent identity for all CNS within an orthologous CRE sequence. None means that no sequence rose to the level of 70% sequence identity over 100 base pairs or more. N/A stands for not applicable.

Supplementary Table S3. ***Eip74EF*** **small interfering RNA designs.**

Exon 7

siRNA_id	Position	SS Sequence	AS Sequence (Guide)	Score	Seed match	>15 bp off-target match
1	2	CCGAAAUUCCUAUUGUCAAGC	UUGACAAUAGGAAUUUCGGUG	99.6	no	no
2	57	GCAGCAGCACCACCUGCAACA	UUGCAGGUGGUGCUGCUGCUG	96.2	no	yes
3	177	GCUGCAGCAGCAGCAACAACA	UUGUUGCUGCUGCUGCAGCUG	95.1	no	ERROR
4	125	CCGCUCUGCUCCACAUAAAGA	UUUAUGUGGAGCAGAGCGGUG	91.5	no	no
5	10	CCUAUUGUCAAGCAAUCGACA	UCGAUUGCUUGACAAUAGGAA	91.5	no	no

Exon 8

siRNA_id	Position	SS Sequence	AS Sequence (Guide)	Score	Seed match	>15bp off-target match
6	191	CGAAUUUAUACCAGAACAAUG	UUGUUCUGGUAUAAAUUCGGA	97.3	no	no

Exon 9

siRNA_id	Position	SS Sequence	AS Sequence (Guide)	Score	Seed match	>15bp off-target match
7	291	GCAGCAGCAGCAACAGCAACA	UUGCUGUUGCUGCUGCUGCUG	98.8	yes	ERROR
8	535	GGCGGAUCCCAGAGUGUUAUC	UAACACUCUGGGAUCCGCCCG	97.8	no	no
9	137	AGCGAGGAACCACAAUCAAUG	UUGAUUGUGGUUCCUCGCUGG	96.5	no	no
10	578	GCUACGAUCUCUCCUACAUGC	AUGUAGGAGAGAUCGUAGCUG	96	no	no
11	953	AGGACAUCAUCGAGAUUGACU	UCAAUCUCGAUGAUGUCCUUG	94.3	no	no
12	138	GCGAGGAACCACAAUCAAUGG	AUUGAUUGUGGUUCCUCGCUG	94	no	yes
13	869	GGGCGCUGAGGUACUACUACC	UAGUAGUACCUCAGCGCCCUG	93.7	no	no
14	770	GGGAGAAGGGCGUCUUCAAGC	UUGAAGACGCCCUUCUCCCGG	93.4	no	yes
15	300	GCAACAGCAACAGCAGCAACA	UUGCUGCUGUUGCUGUUGCUG	93.4	yes	ERROR
16	258	GCAGGCGUUGCAUCAGCAACU	UUGCUGAUGCAACGCCUGCUG	92.5	no	no
17	833	AGAACAAGCCGGACAUGAACU	UUCAUGUCCGGCUUGUUCUUG	92.2	no	yes
18	959	UCAUCGAGAUUGACUGCAACG	UUGCAGUCAAUCUCGAUGAUG	91.4	no	no
19	1	CCCGACAACUACUACGGAAGC	UUCCGUAGUAGUUGUCGGGCA	91.3	no	yes
20	86	UGACAACGUCCUACUUCAACG	UUGAAGUAGGACGUUGUCAGG	91	no	no
21	189	GCAACAGCAGUCGCAGCAAUC	UUGCUGCGACUGCUGUUGCUG	90.7	yes	yes
22	127	GCGGCGUCCCAGCGAGGAACC	UUCCUCGCUGGGACGCCGCUG	90.6	no	yes
23	935	AGUUCGUGGAUGUGCCCAAGG	UUGGGCACAUCCACGAACUGG	90.6	no	no
24	174	CCAGCAGCAGCAGCAGCAACA	UUGCUGCUGCUGCUGCUGGUG	90.5	yes	ERROR
25	80	GCUACCUGACAACGUCCUACU	UAGGACGUUGUCAGGUAGCCA	90.4	no	no
26	285	CCAACAGCAGCAGCAGCAACA	UUGCUGCUGCUGCUGUUGGUG	90.2	yes	ERROR
27	201	GCAGCAAUCGCAGCAGCAACA	UUGCUGCUGCGAUUGCUGCGA	90.1	yes	ERROR
28	839	AGCCGGACAUGAACUACGAGA	UCGUAGUUCAUGUCCGGCUUG	90.1	no	yes
29	844	GACAUGAACUACGAGACGAUG	UCGUCUCGUAGUUCAUGUCCG	90.1	no	yes
30	702	GUGGGAGUUCCUCCUCAAACU	UUUGAGGAGGAACUCCCACAG	90	no	yes

ERROR indicates a failed BLAST search. These guide candidates were not further considered for use.

Supplementary Table S4. Primer pairs used to create CRE reporter transgenes to test 19 predicted CREs from the Weinstein et al. 2023 expanded dorsal pupal abdomen CRE training set

Forward primer with introduced restriction enzyme site (lower case)	Reverse primer with introduced restriction enzyme site (lower case)	pCRE name
TTCCGggcgcgccCATAACAGCAATTCCAGTGACAACAG	TTGCCcctgcaggTGCGAGCGTTCTTATGGGCAACTGG	S3.1
TTCCGggcgcgccGTGGCAACAAACAGTGAGTGAGTG	TTGCCcctgcaggAGCTTCTAATGCTTTAGTTTGCCTGC	S3.2
TTCCGggcgcgccGACGAGCGTAAGTAAGTAAGACG	TTGCCcctgcaggTACAACAGCCACTATTCAACCACC	S3.3
TTCCGggcgcgccGCTGAGATCGTTGCAGAAATCATGC	TTGCCcctgcaggATCTGAACTCTTGTGTTTGCTTTGG	S3.4
TTCCGggcgcgccACTTGCTTCGCACTTACAGATGC	TTGCCcctgcaggAATCACTTACACACACCCACTGTGC	S3.5
TTCCGggcgcgccAAGCTTTAGTTCGCGAAGAAGAAACG	TTGCCcctgcaggTGTGTGGCCGCGTTTTTGGCAC	S3.6
TTCCGggcgcgccAACCGAAAGATGCAAAGATACATACG	TTGCCcctgcaggTTGCTCTCTTAACTTACTGAAGCAC	S3.7
TTCCGggcgcgccATTGTCGCCCAGAGTTTGCTACTGC	TTGCCcctgcaggATTCACAATCATCATCAGCGGAGTGG	S3.8
TTCCGggcgcgccTGCACAAAATCGCAGCTAAAAACGAGG	TTGCCcctgcaggTCCAAAGGAGTGGTGGCAACAACG	S3.9
TTCCGggcgcgccAGTCTAATGATTACACTTTCCGGTGC	TTGCCcctgcaggCAACCATTTTGAACTGATATGAGAGACG	S3.10
TTCCGggcgcgccTGTTACATGCCTAGTCTTGCAATGG	TTGCCcctgcaggATCCCAGACGAGCGAAAGAGAGG	S3.11
TTCCGggcgcgccGCAAAAGGCCCATGAAGACTTGG	TTGCCcctgcaggTCGCAATGAGCCGTTCGAAAATGTC	S3.12
TTCCGggcgcgccTCTCTTTGATACCCTGCAATGCC	TTGCCcctgcaggACTATGTCTATACCTGGTTGGTTGG	S3.13
TTCCGggcgcgccGCCAACAAGACCAACAACATAGG	TTGCCcctgcaggGTTTTAGCACATTCTCAGAATCGAGAG	S3.14
TTCCGggcgcgccGGAGAAAACAGGCGTGTGACG	TTGCCcctgcaggTGTGAGTTAGTGAGTAAGTGAGATGG	S3.15
TTCCGggcgcgccCAGTTGAGAACCCTCCCATATGG	TTGCCcctgcaggGGTGCATACATAATAACACTGCTGG	S3.16
TTCCGggcgcgccTCCTCTCGTTTTACGGCTCATTTCG	TTGCCcctgcaggCAGCTGCGGTTTCGCGTTACTGG	S3.17
TTCCGggcgcgccGAACATTAAACTGTGGACCACTAGC	TTGCCcctgcaggACAGGAAAGTGACCAATTGGTTGG	S3.18
TTCCGggcgcgccGATCTACTGTCTTTCGAGGGTACG	TTGCCcctgcaggGTGGGCTCTTGGCAACTGTGG	S3.19

Note: Lower case letters indicate a sequence for an introduced restriction enzyme site. *Asc*I is ggcgcgcc and *Sbf*I is cctgcagg.

Supplementary Table S5. Primer pairs used to create reporter transgenes to test the *D. willistoni* orthologous *Eip74EF S3.20-S3.29* sequences for CRE activity

Forward primer with introduced restriction enzyme site (lower case)	Reverse primer with introduced restriction enzyme site (lower case)	pCRE name
TTCCGggcgcgccAGCCTTAGTTGAGGTCTTGCAATGC	TTGCCcctgcaggAGGTACCAGTTATCTTTCCAGTGG	S3.20
TTCCGggcgcgccTCGATGGCATTTTCTTTGATCAGTG	TTGCCcctgcaggTACACTTACAACAAACGACGAGCGC	S3.21
TTCCGggcgcgccTCCATCGGTGGGCAATGACGAC	TTGCCcctgcaggCTGCAAAAACTGCACAGGCAAAACTC	S3.22
TTCCGggcgcgccTTGAGTTTTGCCTGTGCAGTTTTTGC	TTGCCcctgcaggCACCATCATTATCCCTCACATCACC	S3.23
TTCCGggcgcgccGCATCAAAATTACGGAACCTCTTCC	TTGCCcctgcaggGCGCACGCTTTGCTTATACATTTGC	S3.24
TTCCGggcgcgccGCGCTTTAGATGGCAGTTGTCG	TTGCCcctgcaggAAATGGGTCAGGCTATATGAAAAAGGG	S3.25
TTCCGggcgcgccGCCCCATAAAAAAGCGTCTTAATCG	TTGCCcctgcaggTGAGGCATTGATGTTGTTGTTGC	S3.26
TTGCCcctgcaggACTGCCACCTGCAAAAATCGAAACG	TTCCGggcgcgccGCCAAAAGGAAGGAAGGAAGGTAGG	S3.27
TTGCCcctgcaggAGGCAATCAAAATGAGACGACGTCG	TTCCGggcgcgccCCAATTTGGTGAGCCATGAACTC	S3.28
TTGCCcctgcaggCAGACAGTTCATTTTGTGCGGTTTGC	TTCCGggcgcgccTTATGCTTCACCCCCTAAGTTATGC	S3.29

Note: Lower case letters indicate a sequence for an introduced restriction enzyme site. *Asc*I is ggcgcgcc and *Sbf*I is cctgcagg.
