# Supplementary material for: Redundant and Singular Regulatory Elements Underlie the Rapidly Evolving Pigmentation of Drosophila
Source: Mol Biol Evol. 2025 Sep 4;42(9):msaf213. doi: 10.1093/molbev/msaf213 (PMC12449766; doi:10.1093/molbev/msaf213)
Supplement: msaf213_Supplementary_Data [file msaf213_supplementary_data.zip › Supplementary Document 1 grh mVISTAs combined (08.05.25).pdf]

melanogaster S3.7grh:1-798

Alignment 1
malerkotliana
grh (-)
15869-16659
Criteria: 70%, 100 bp
Regions: 2

Alignment 2
pseudoobscura
grh (-)
16132-16896
Criteria: 70%, 100 bp
Regions: 2

Alignment 3
willistoni
grh (-)
18331-19146
Criteria: 70%, 100 bp
Regions: 3

Alignment 4
saltans
grh (-)
16920-17823
Criteria: 70%, 100 bp
Regions: 3

Alignment 5
virilis
grh (-)
16928-17623
Criteria: 70%, 100 bp
Regions: 2

X-axis: melanogaster
Resolution: 1
Window size: 100 bp

contig
gene
exon
UTR
CNS
mRNA

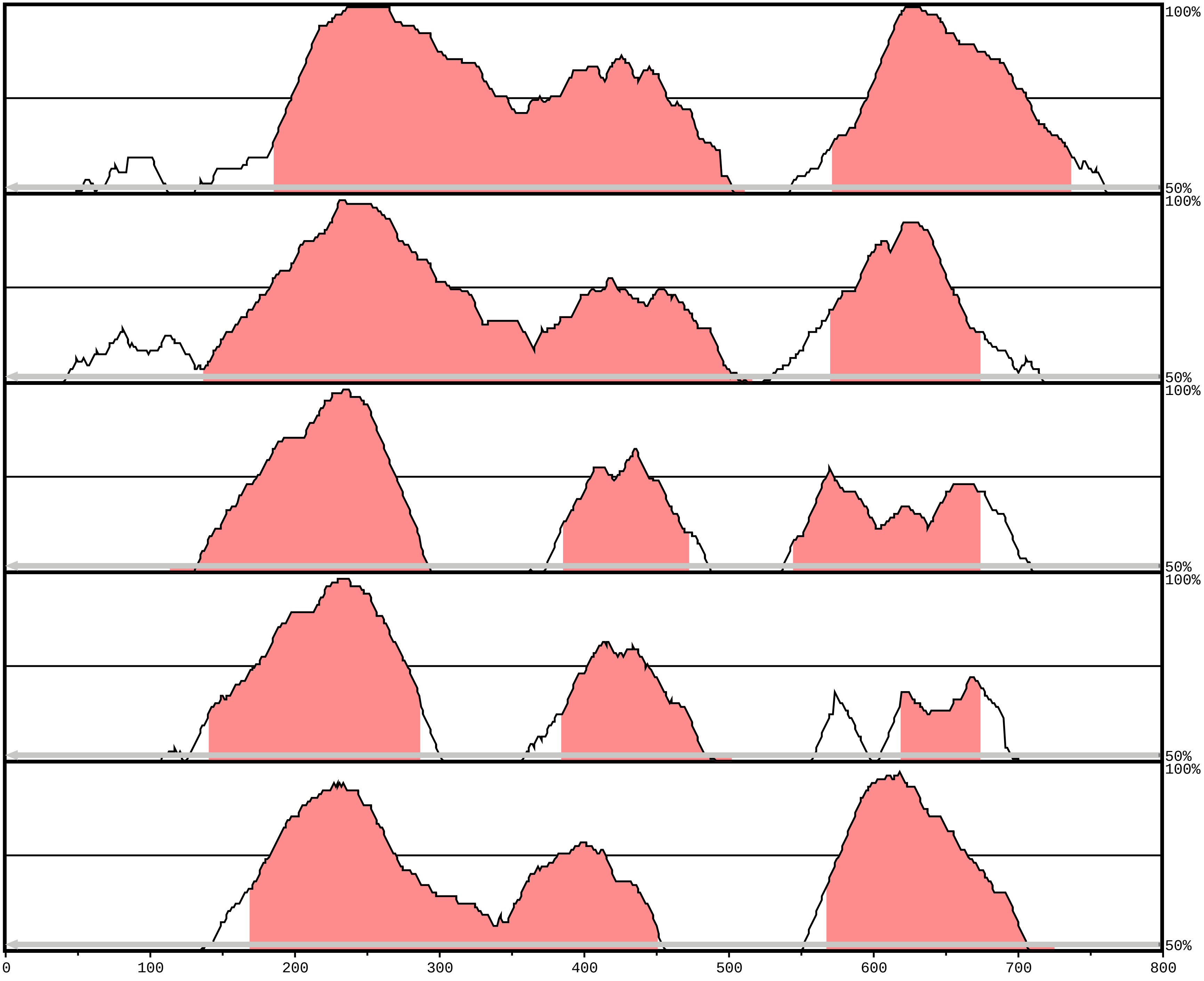

melanogaster S3.8grh:1-690

Alignment 1
malerkotliana
grh (-)
23567-24290
Criteria: 70%, 100 bp
Regions: 2

Alignment 2
pseudoobscura
grh (-)
23402-24109
Criteria: 70%, 100 bp
Regions: 1

Alignment 3
willistoni
grh
2 alignments
Criteria: 70%, 100 bp
Regions: 1

Alignment 4
saltans
grh
2 alignments
Criteria: 70%, 100 bp
Regions: 1

Alignment 5
virilis
grh
2 alignments
Criteria: 70%, 100 bp
Regions: 1

X-axis: melanogaster
Resolution: 1
Window size: 100 bp

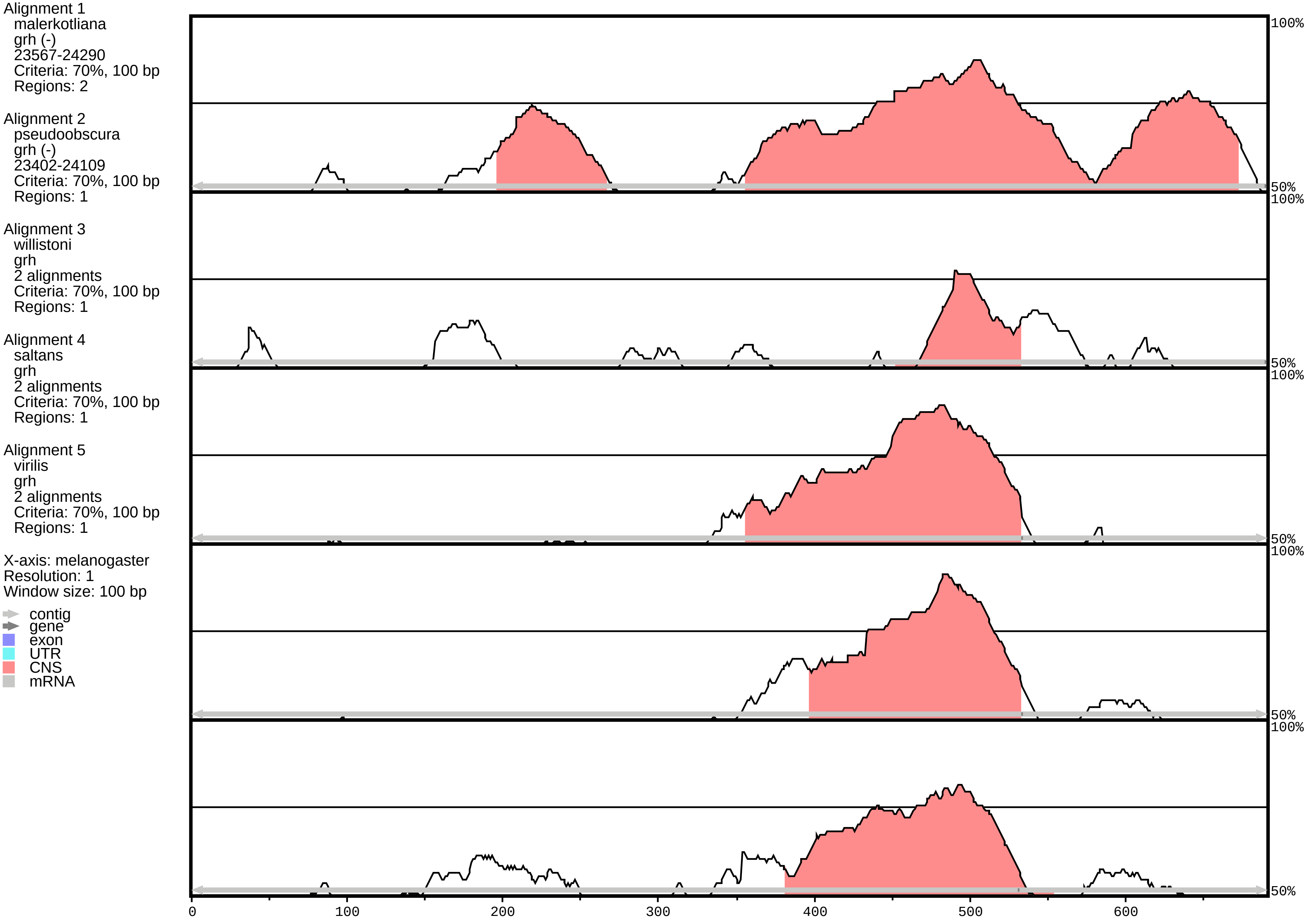

melanogaster S3.9grh:1-1152

Alignment 1
malerkotliana
grh (-)
38786-39966
Criteria: 70%, 100 bp
Regions: 2

Alignment 2
pseudoobscura
grh (-)
38970-40230
Criteria: 70%, 100 bp
Regions: 3

Alignment 3
willistoni
grh (-)
46319-47578
Criteria: 70%, 100 bp
Regions: 3

Alignment 4
saltans
grh (-)
46171-47394
Criteria: 70%, 100 bp
Regions: 2

Alignment 5
virilis
grh (-)
46699-47827
Criteria: 70%, 100 bp
Regions: 1

X-axis: melanogaster
Resolution: 1
Window size: 100 bp

contig
gene
exon
UTR
CNS
mRNA

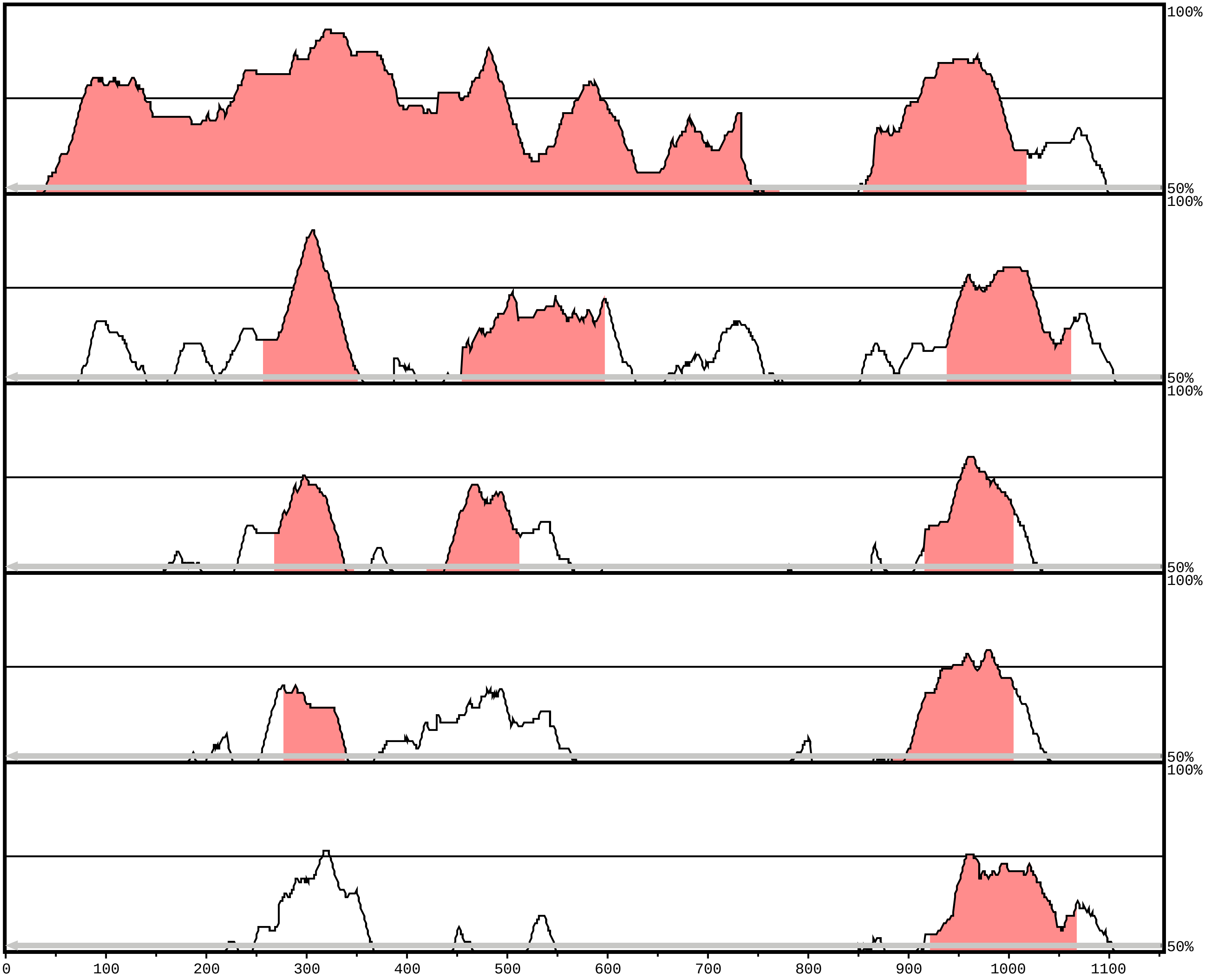

melanogaster S3.10grh:1-1077

Alignment 1
malerkotliana
grh (-)
24593-26321
Criteria: 70%, 100 bp
Regions: 3

Alignment 2
pseudoobscura
grh (-)
24396-25802
Criteria: 70%, 100 bp
Regions: 2

Alignment 3
willistoni
grh (-)
28521-29832
Criteria: 70%, 100 bp
Regions: 0

Alignment 4
saltans
grh (-)
27155-28454
Criteria: 70%, 100 bp
Regions: 0

Alignment 5
virilis
grh (-)
27382-28850
Criteria: 70%, 100 bp
Regions: 2

X-axis: melanogaster
Resolution: 1
Window size: 100 bp

contig
gene
exon
UTR
CNS
mRNA

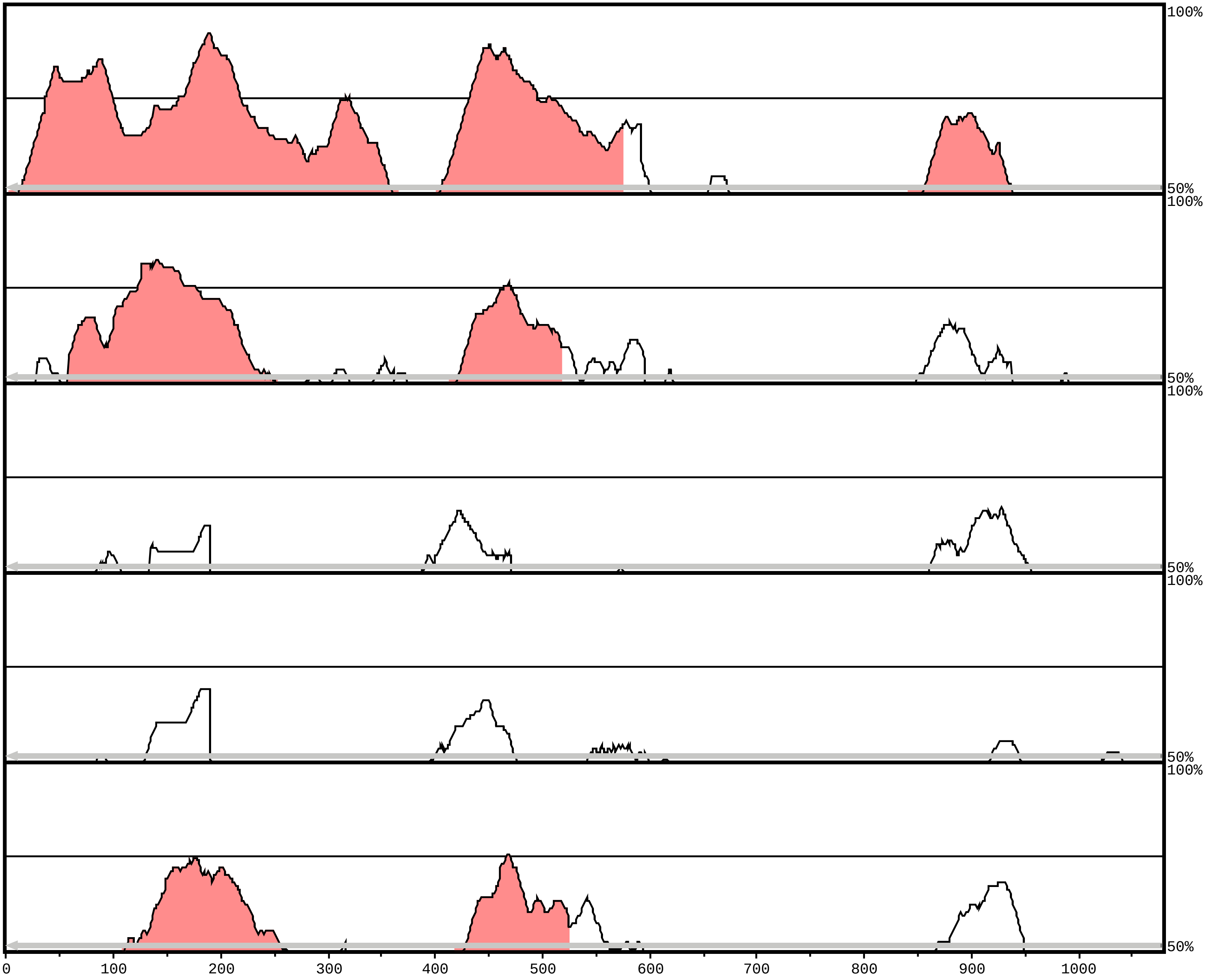

mel grh:1-42766

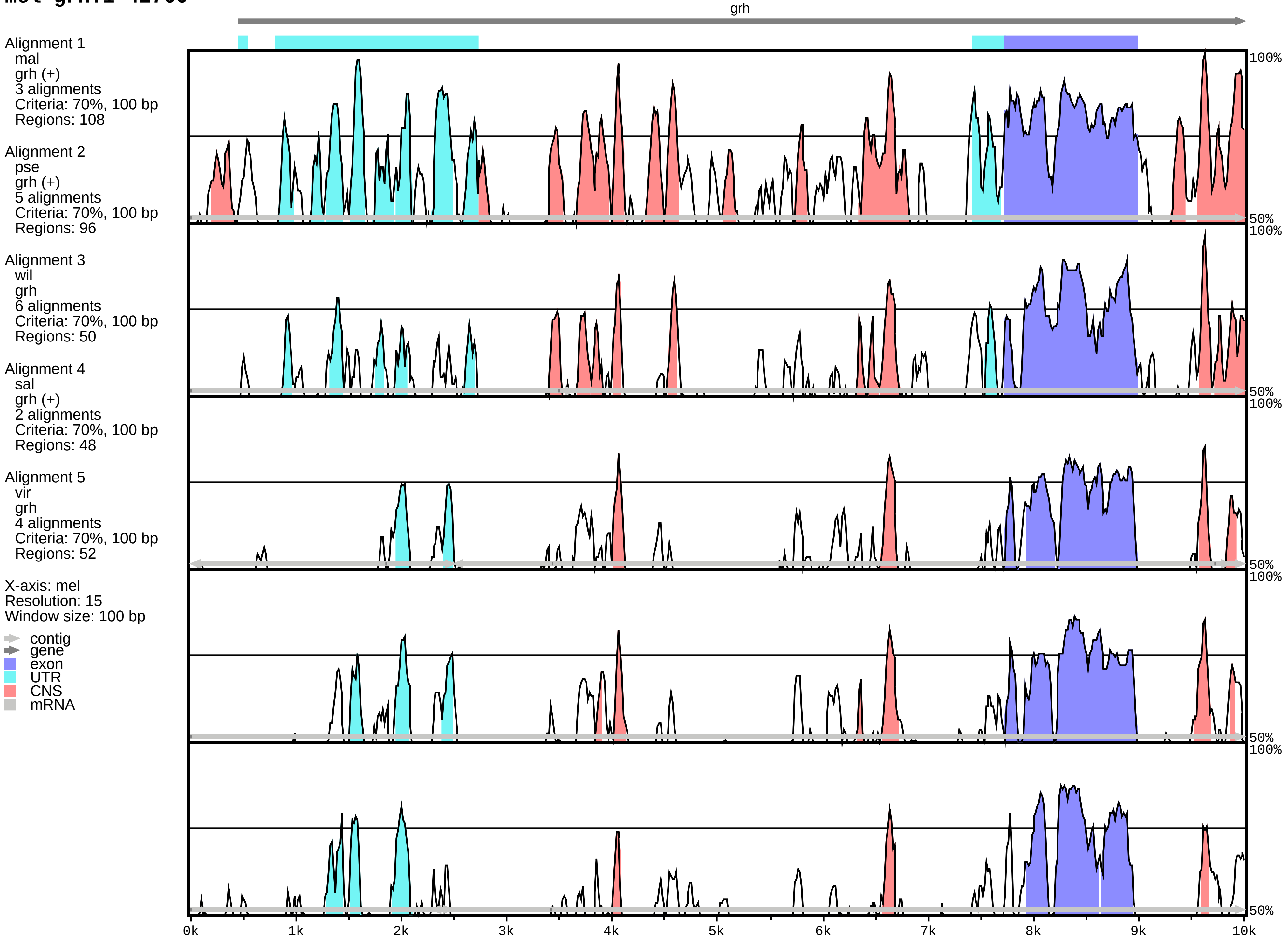

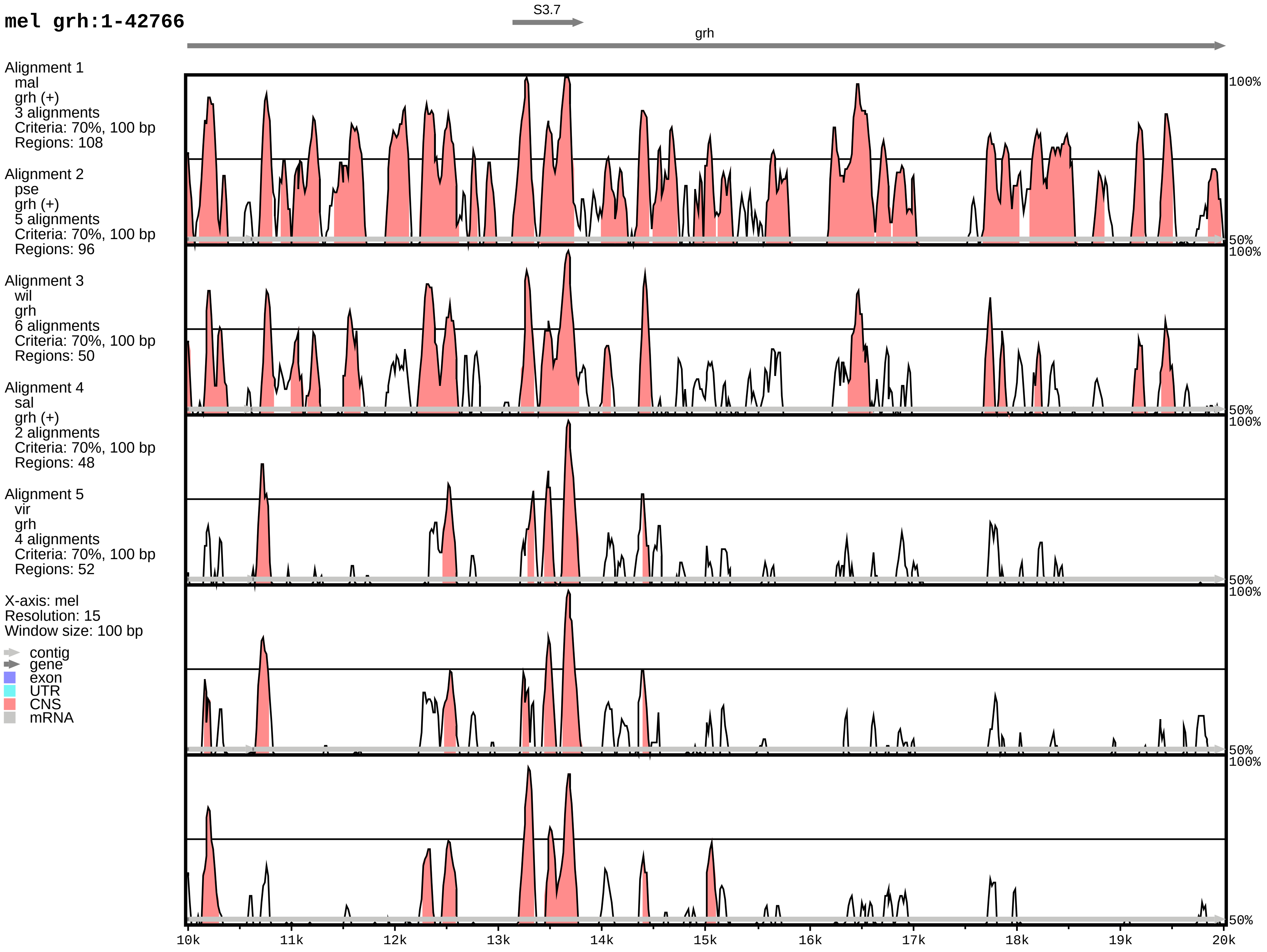

mel grh:1-42766
S3.7
grh
Alignment 1
mal
grh (+)
3 alignments
Criteria: 70%, 100 bp
Regions: 108
Alignment 2
pse
grh (+)
5 alignments
Criteria: 70%, 100 bp
Regions: 96
Alignment 3
wil
grh
6 alignments
Criteria: 70%, 100 bp
Regions: 50
Alignment 4
sal
grh (+)
2 alignments
Criteria: 70%, 100 bp
Regions: 48
Alignment 5
vir
grh
4 alignments
Criteria: 70%, 100 bp
Regions: 52
X-axis: mel
Resolution: 15
Window size: 100 bp
contig
gene
exon
UTR
CNS
mRNA
100%
50%
10k
11k
12k
13k
14k
15k
16k
17k
18k
19k
20k

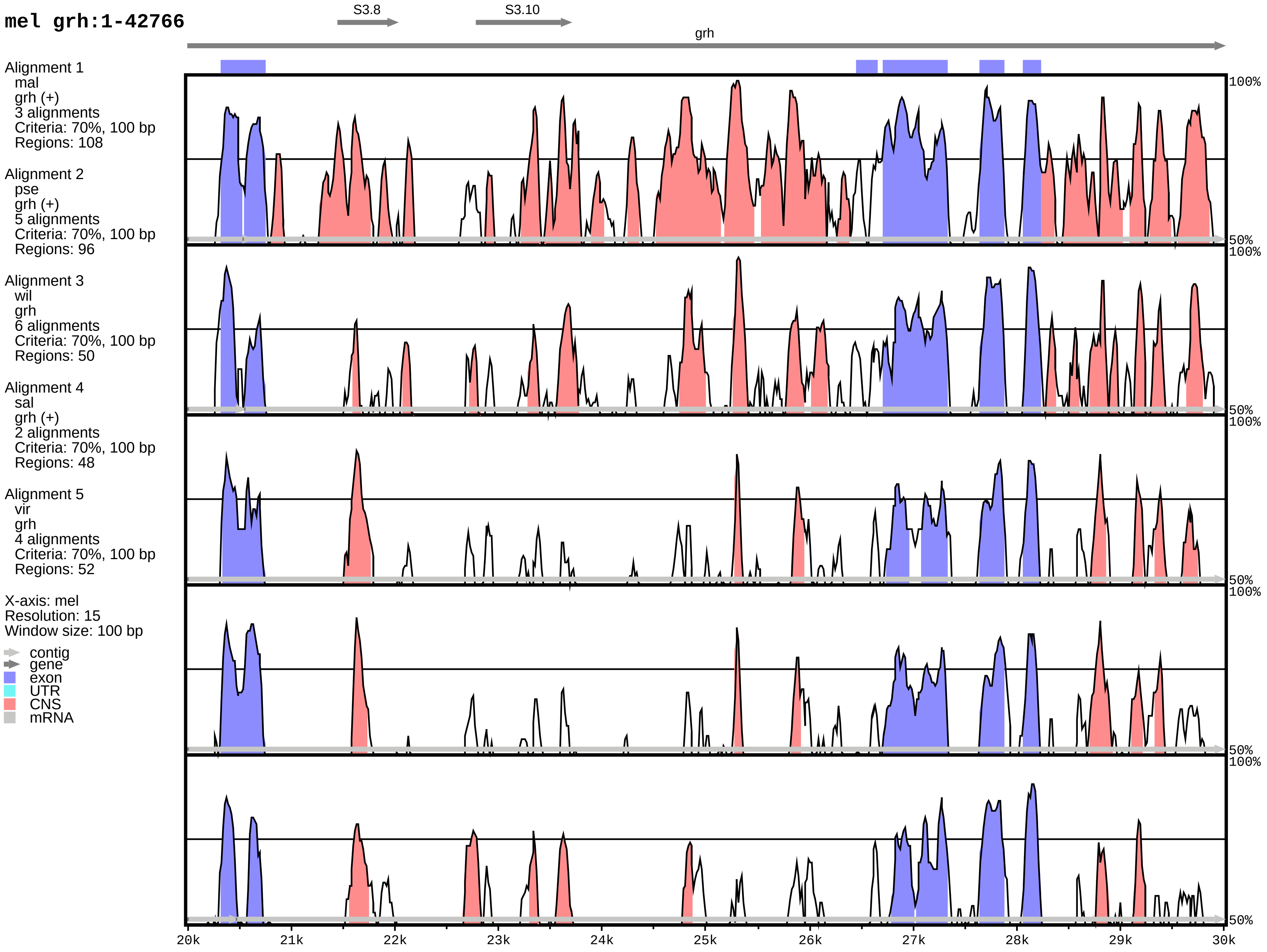
mel grh:1-42766
S3.8
S3.10
grh
Alignment 1
mal
grh (+)
3 alignments
Criteria: 70%, 100 bp
Regions: 108
Alignment 2
pse
grh (+)
5 alignments
Criteria: 70%, 100 bp
Regions: 96
Alignment 3
wil
grh
6 alignments
Criteria: 70%, 100 bp
Regions: 50
Alignment 4
sal
grh (+)
2 alignments
Criteria: 70%, 100 bp
Regions: 48
Alignment 5
vir
grh
4 alignments
Criteria: 70%, 100 bp
Regions: 52
X-axis: mel
Resolution: 15
Window size: 100 bp
contig
gene
exon
UTR
CNS
mRNA
100%
50%
20k
21k
22k
23k
24k
25k
26k
27k
28k
29k
30k

mel grh:1-42766

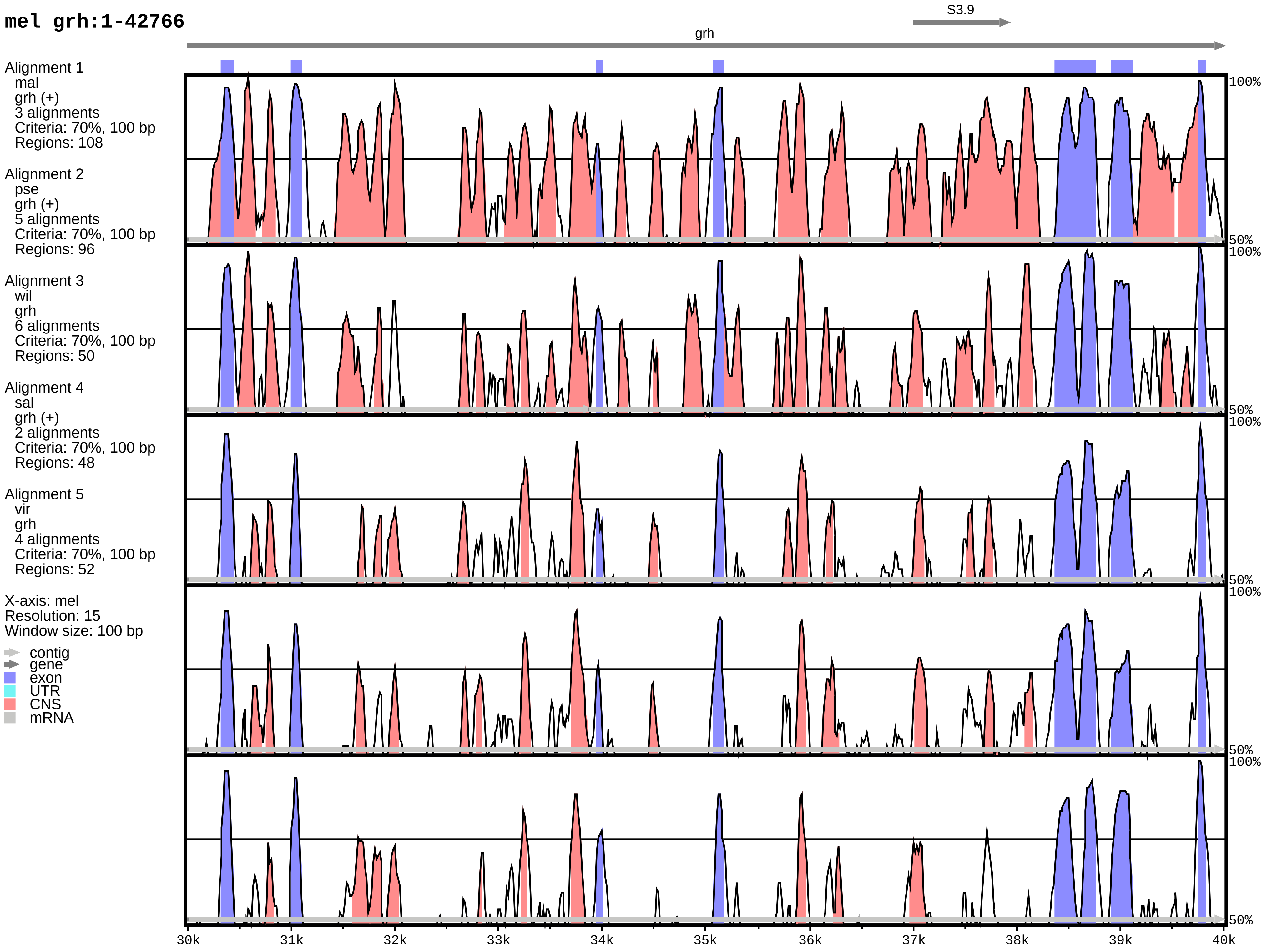

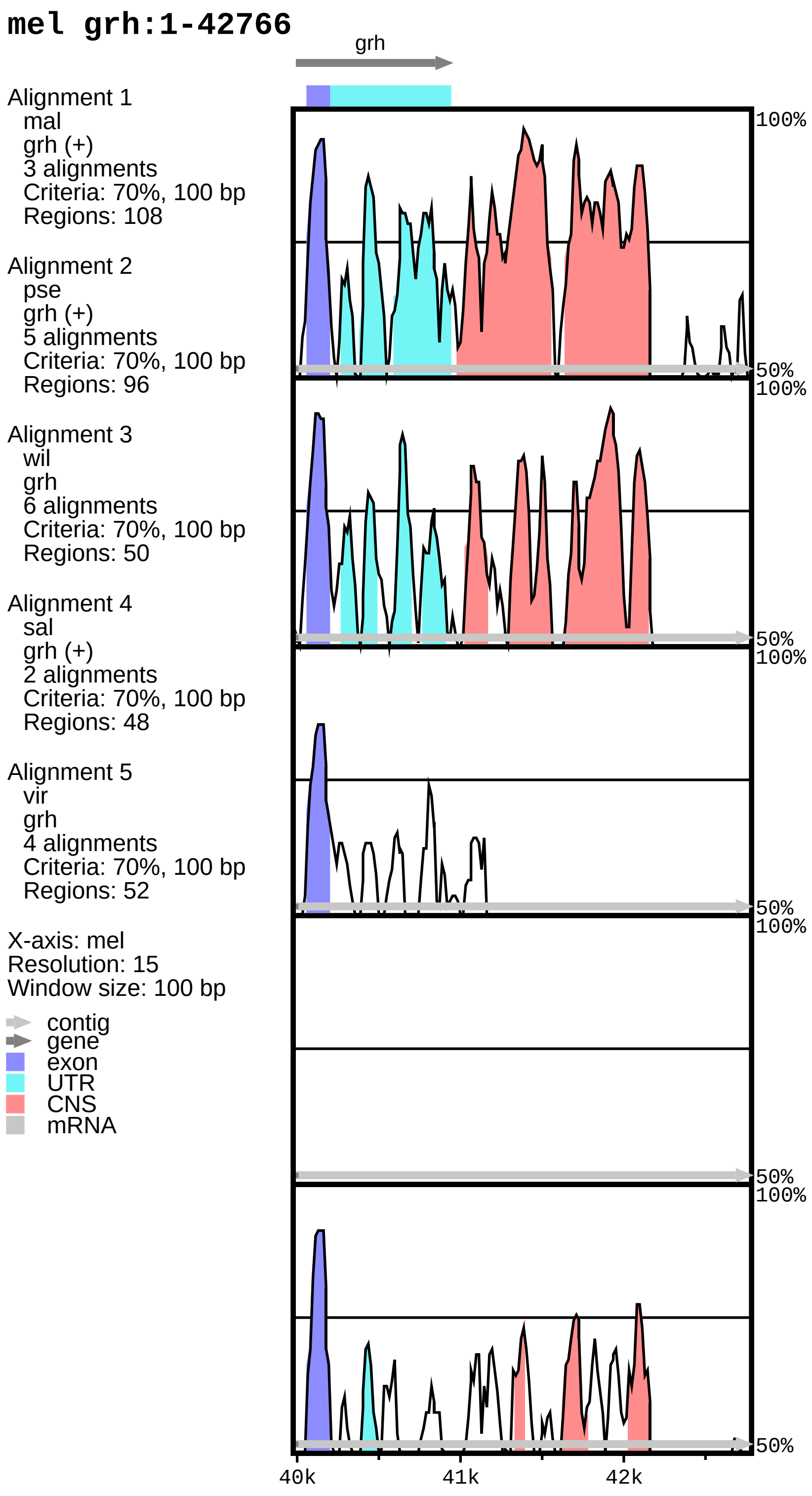
mel grh:1-42766
grh
Alignment 1
mal
grh (+)
3 alignments
Criteria: 70%, 100 bp
Regions: 108
Alignment 2
pse
grh (+)
5 alignments
Criteria: 70%, 100 bp
Regions: 96
Alignment 3
wil
grh
6 alignments
Criteria: 70%, 100 bp
Regions: 50
Alignment 4
sal
grh (+)
2 alignments
Criteria: 70%, 100 bp
Regions: 48
Alignment 5
vir
grh
4 alignments
Criteria: 70%, 100 bp
Regions: 52
X-axis: mel
Resolution: 15
Window size: 100 bp
contig
gene
exon
UTR
CNS
mRNA
100%
50%
40k
41k
42k
