# Supplementary material for: Redundant and Singular Regulatory Elements Underlie the Rapidly Evolving Pigmentation of Drosophila
Source: Mol Biol Evol. 2025 Sep 4;42(9):msaf213. doi: 10.1093/molbev/msaf213 (PMC12449766; doi:10.1093/molbev/msaf213)

melanogaster S3.11hth:1-1270

Alignment 1
malerkotliana
hth (+)
17021-18213
Criteria: 70%, 100 bp
Regions: 3

Alignment 2
pseudoobscura
hth (+)
62007-63189
Criteria: 70%, 100 bp
Regions: 2

Alignment 3
willistoni
hth (+)
77482-78952
Criteria: 70%, 100 bp
Regions: 1

Alignment 4
saltans
hth (+)
89275-90674
Criteria: 70%, 100 bp
Regions: 2

Alignment 5
virilis
hth (+)
72360-73769
Criteria: 70%, 100 bp
Regions: 1

X-axis: melanogaster
Resolution: 1
Window size: 100 bp

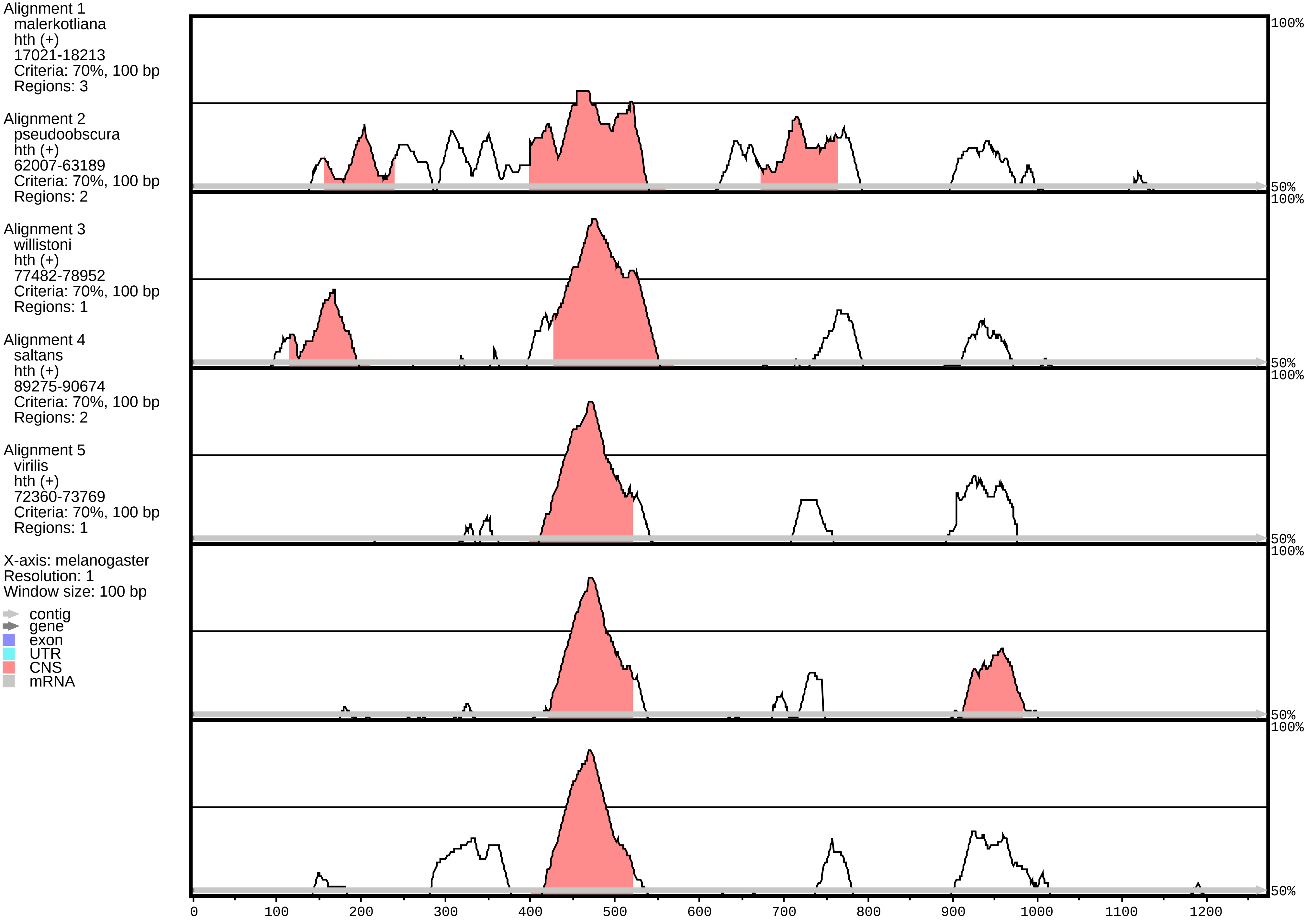

melanogaster S3.14hth:1-679

Alignment 1
malerkotliana
hth (+)
87048-87654
Criteria: 70%, 100 bp
Regions: 2

Alignment 2
pseudoobscura
hth (+)
145484-146325
Criteria: 70%, 100 bp
Regions: 1

Alignment 3
willistoni
hth (+)
167366-168043
Criteria: 70%, 100 bp
Regions: 1

Alignment 4
saltans
hth (+)
190675-191352
Criteria: 70%, 100 bp
Regions: 1

Alignment 5
virilis
hth (+)
168883-169560
Criteria: 70%, 100 bp
Regions: 1

X-axis: melanogaster
Resolution: 1
Window size: 100 bp

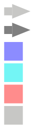

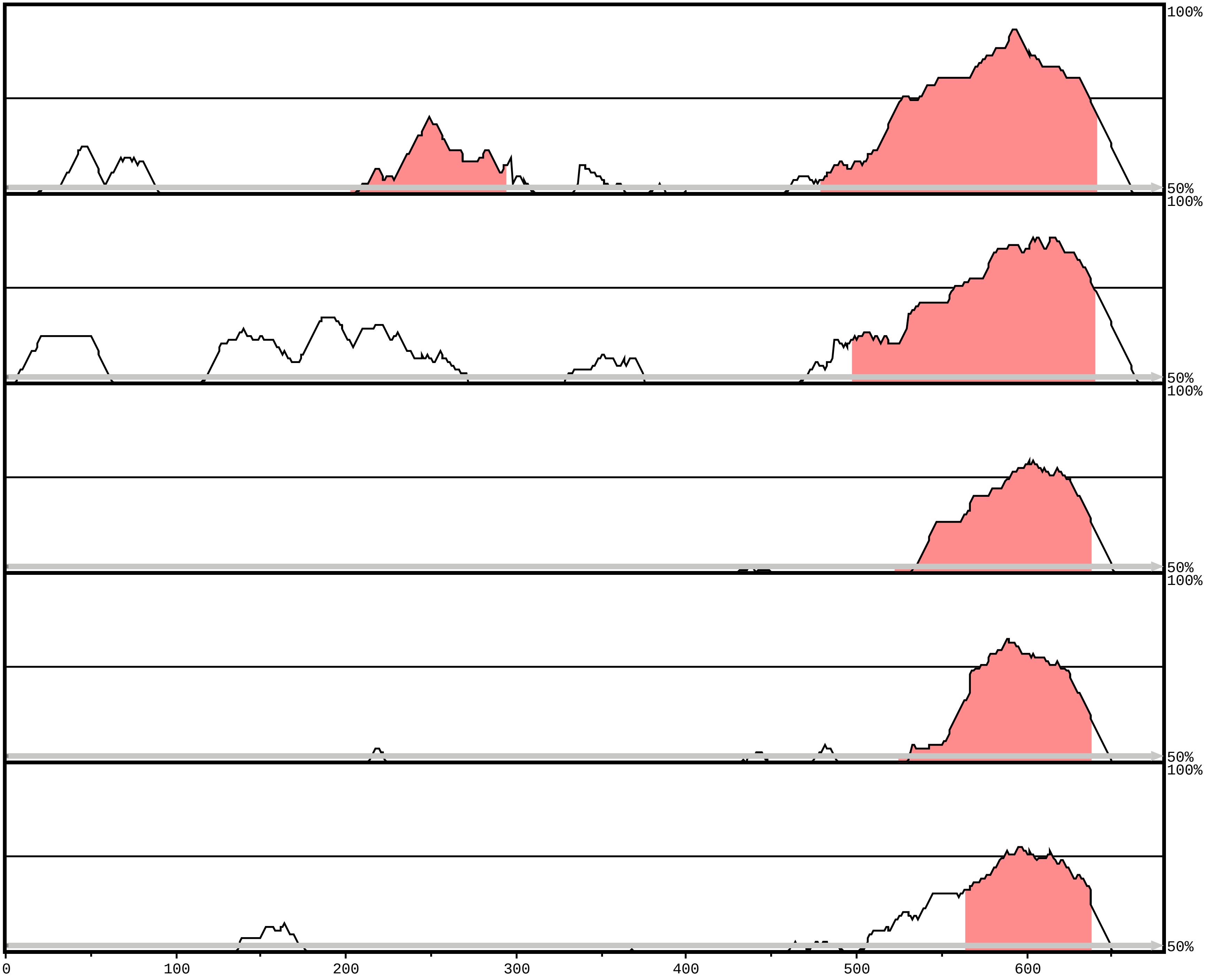

mel hth:1-238857

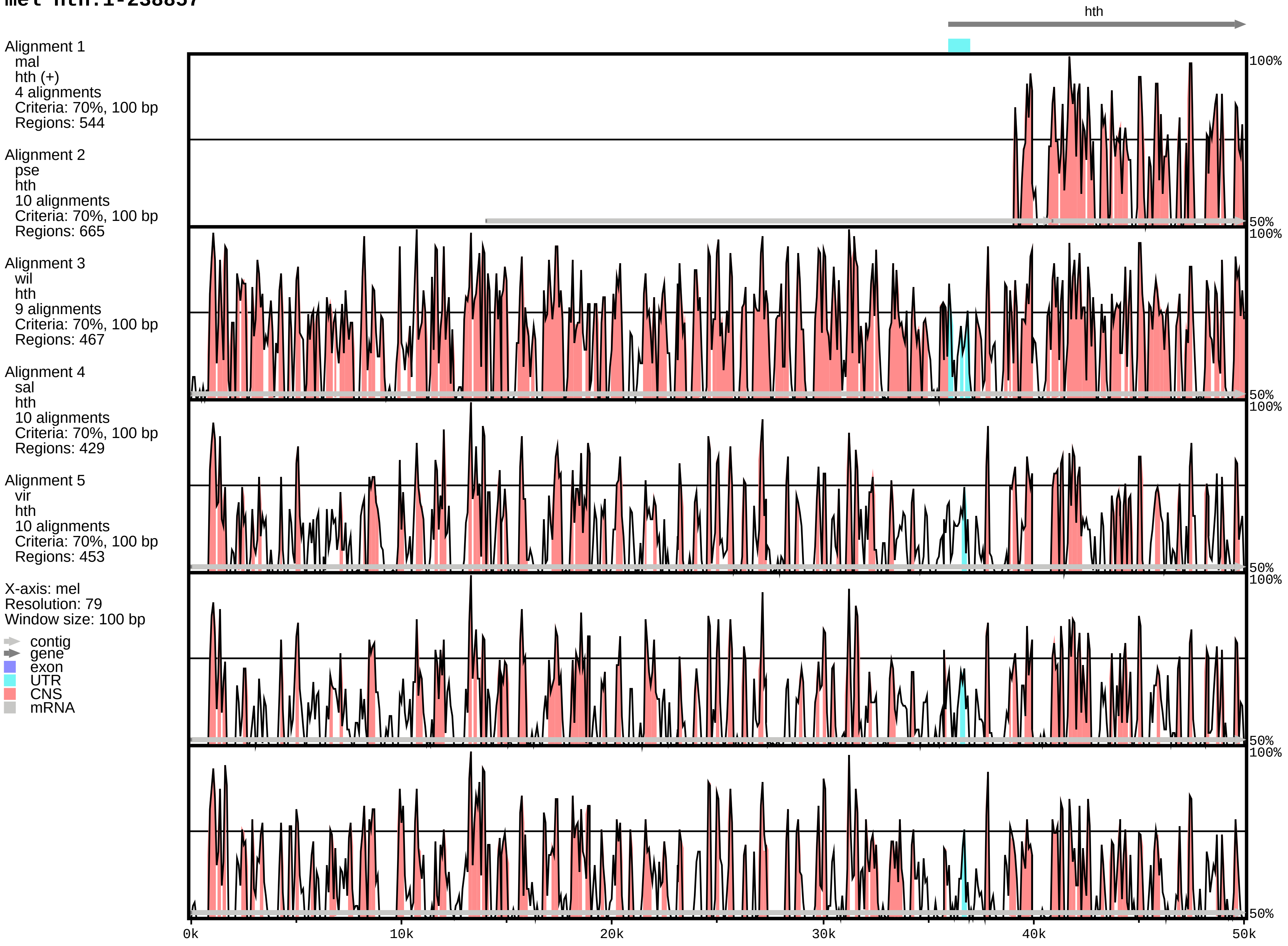

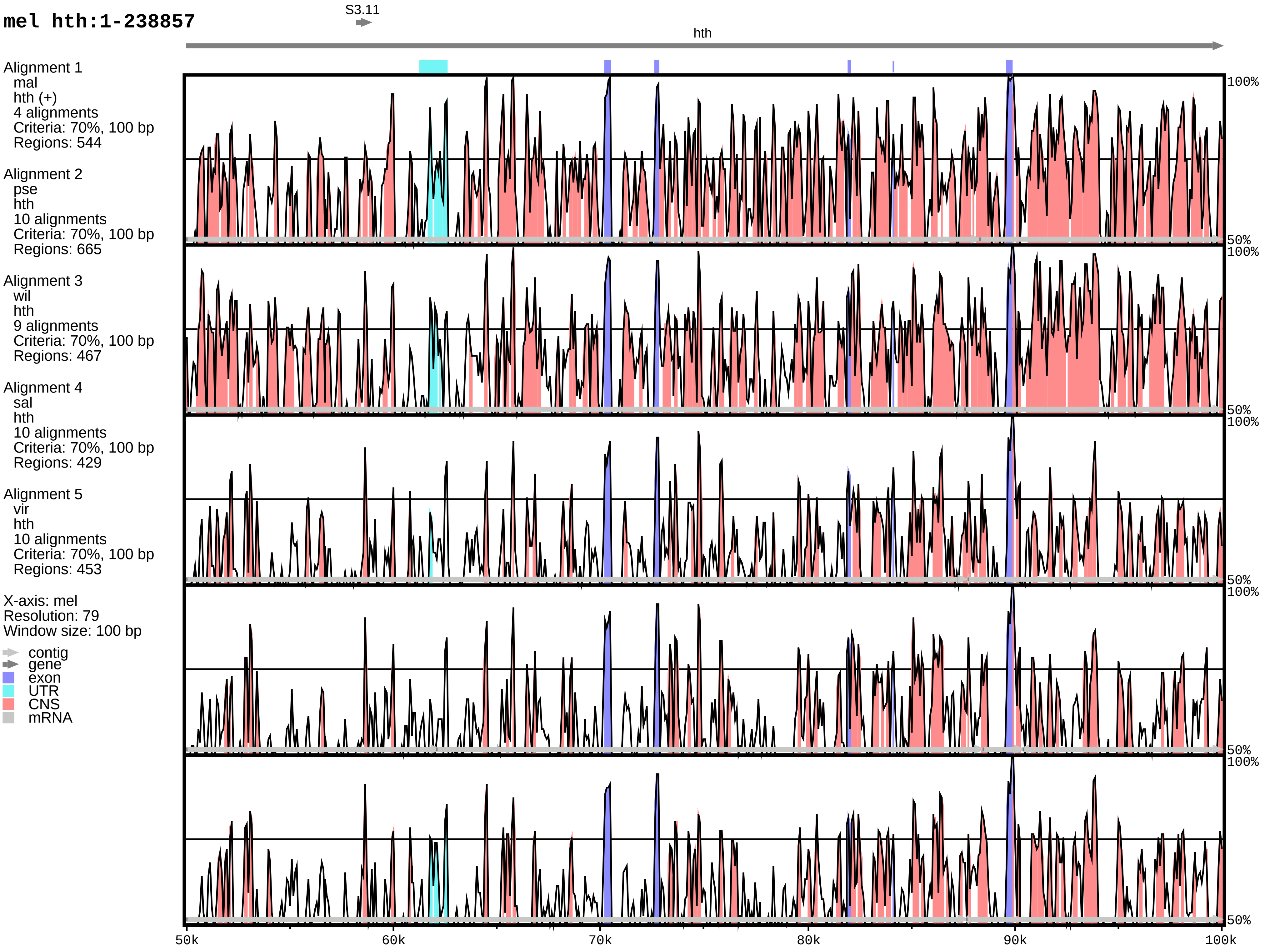

mel hth:1-238857
S3.11
hth
Alignment 1
mal
hth (+)
4 alignments
Criteria: 70%, 100 bp
Regions: 544
Alignment 2
pse
hth
10 alignments
Criteria: 70%, 100 bp
Regions: 665
Alignment 3
wil
hth
9 alignments
Criteria: 70%, 100 bp
Regions: 467
Alignment 4
sal
hth
10 alignments
Criteria: 70%, 100 bp
Regions: 429
Alignment 5
vir
hth
10 alignments
Criteria: 70%, 100 bp
Regions: 453
X-axis: mel
Resolution: 79
Window size: 100 bp
contig
gene
exon
UTR
CNS
mRNA
100%
50%
50k
60k
70k
80k
90k
100k

mel hth:1-238857

Alignment 1
mal
hth (+)
4 alignments
Criteria: 70%, 100 bp
Regions: 544

Alignment 2
pse
hth
10 alignments
Criteria: 70%, 100 bp
Regions: 665

Alignment 3
wil
hth
9 alignments
Criteria: 70%, 100 bp
Regions: 467

Alignment 4
sal
hth
10 alignments
Criteria: 70%, 100 bp
Regions: 429

Alignment 5
vir
hth
10 alignments
Criteria: 70%, 100 bp
Regions: 453

X-axis: mel
Resolution: 79
Window size: 100 bp

contig
gene
exon
UTR
CNS
mRNA

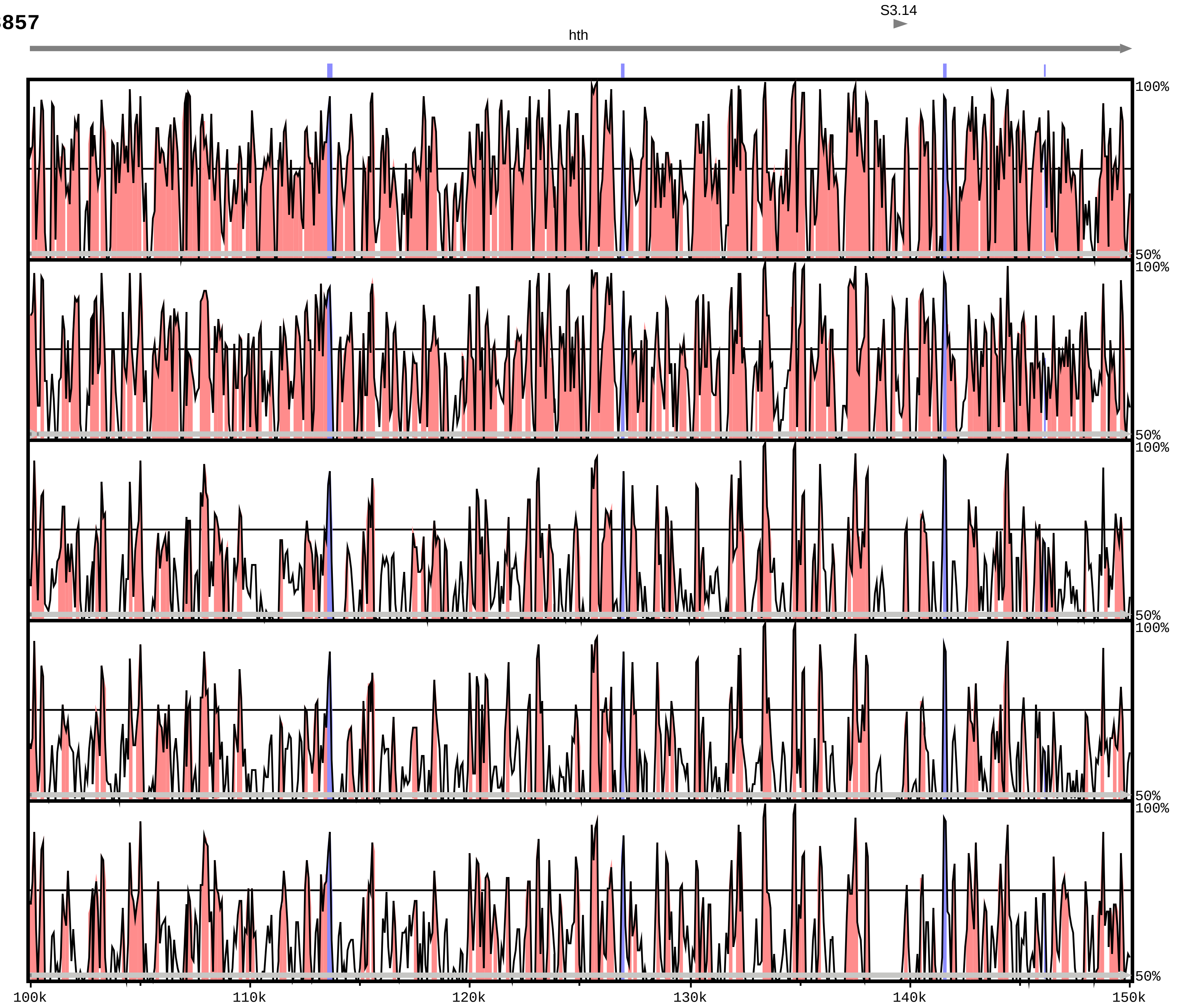

mel hth:1-238857

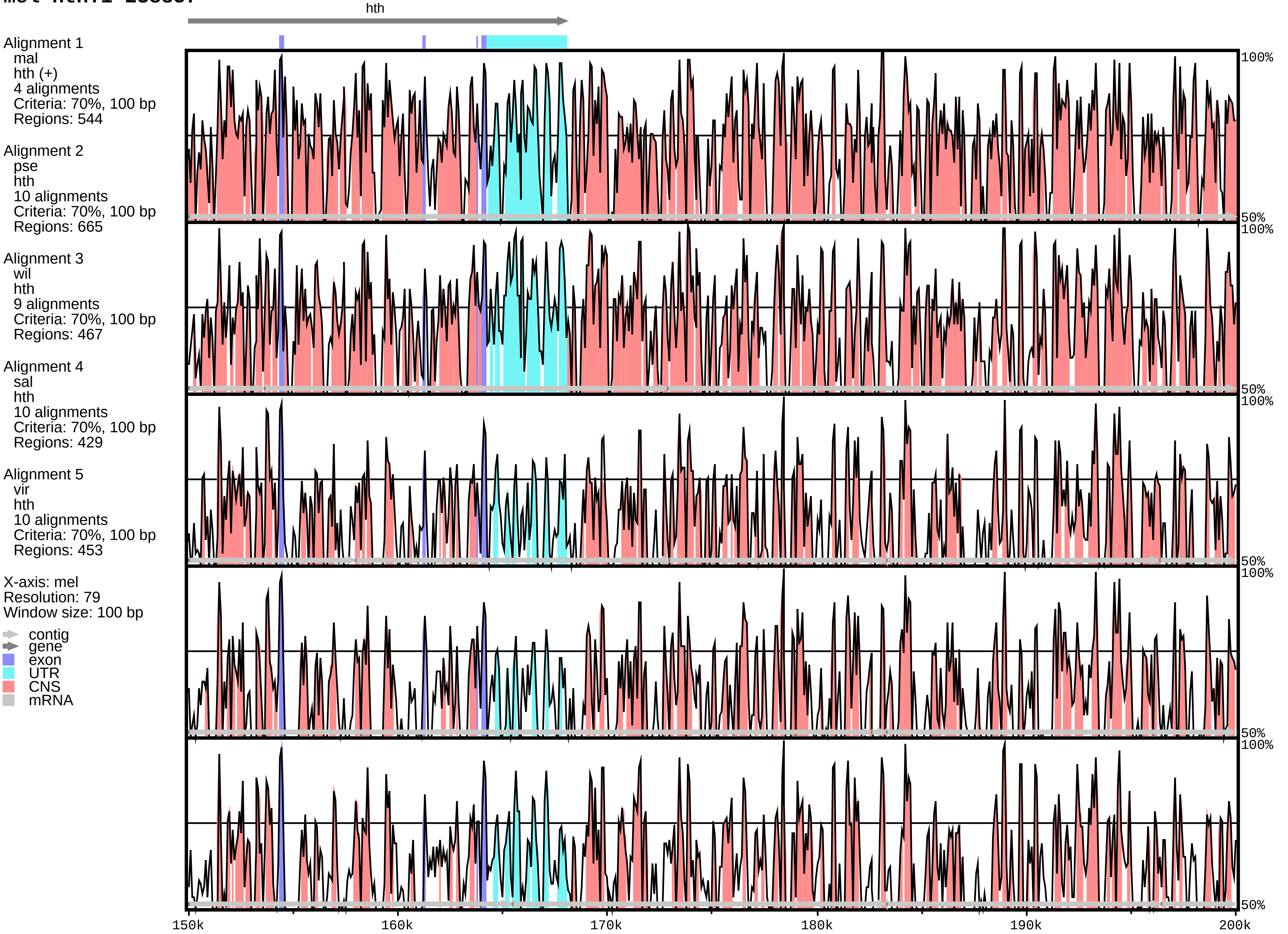

mel hth:1-238857

Alignment 1
mal
hth (+)
4 alignments
Criteria: 70%, 100 bp
Regions: 544

Alignment 2
pse
hth
10 alignments
Criteria: 70%, 100 bp
Regions: 665

Alignment 3
wil
hth
9 alignments
Criteria: 70%, 100 bp
Regions: 467

Alignment 4
sal
hth
10 alignments
Criteria: 70%, 100 bp
Regions: 429

Alignment 5
vir
hth
10 alignments
Criteria: 70%, 100 bp
Regions: 453

X-axis: mel
Resolution: 79
Window size: 100 bp

contig
gene
exon
UTR
CNS
mRNA

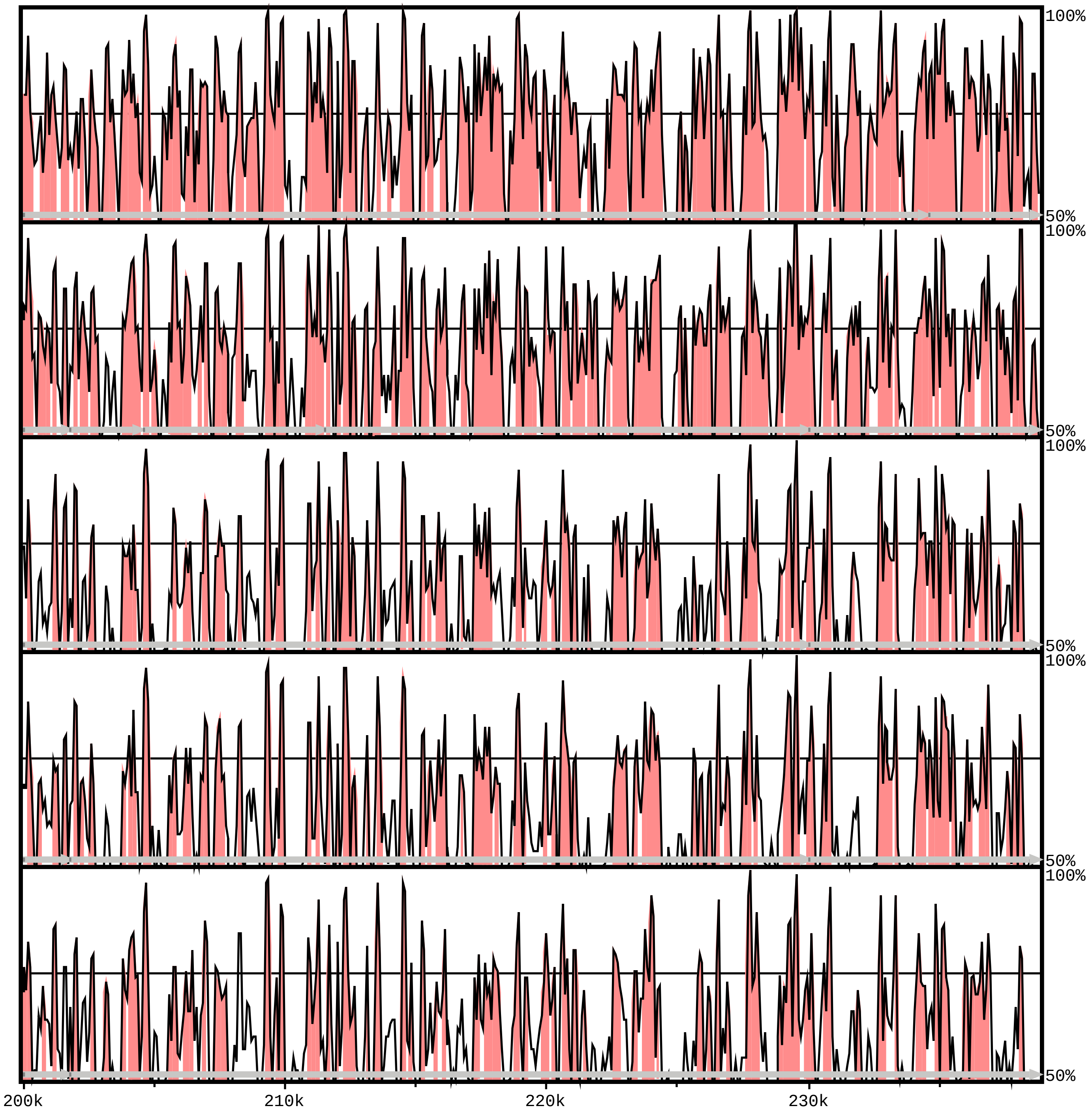

Supplement: msaf213_Supplementary_Data [file msaf213_supplementary_data.zip › Supplementary Document 2 hth mVISTAs combined (08.05.25).pdf]
