# Supplementary material for: Redundant and Singular Regulatory Elements Underlie the Rapidly Evolving Pigmentation of Drosophila
Source: Mol Biol Evol. 2025 Sep 4;42(9):msaf213. doi: 10.1093/molbev/msaf213 (PMC12449766; doi:10.1093/molbev/msaf213)

melanogaster S3.20mel:1-671

Alignment 1
malerkotliana
Eip74EF (+)
8951-9752
Criteria: 70%, 100 bp
Regions: 2

Alignment 2
pseudoobscura
Eip74EF
2 alignments
Criteria: 70%, 100 bp
Regions: 1

Alignment 3
willistoni
Eip74EF (+)
11547-12319
Criteria: 70%, 100 bp
Regions: 1

Alignment 4
saltans
Eip74EF (+)
11629-12405
Criteria: 70%, 100 bp
Regions: 1

Alignment 5
virilis
Eip74EF (+)
8684-9306
Criteria: 70%, 100 bp
Regions: 2

X-axis: melanogaster
Resolution: 1
Window size: 100 bp

contig
gene
exon
UTR
CNS
mRNA

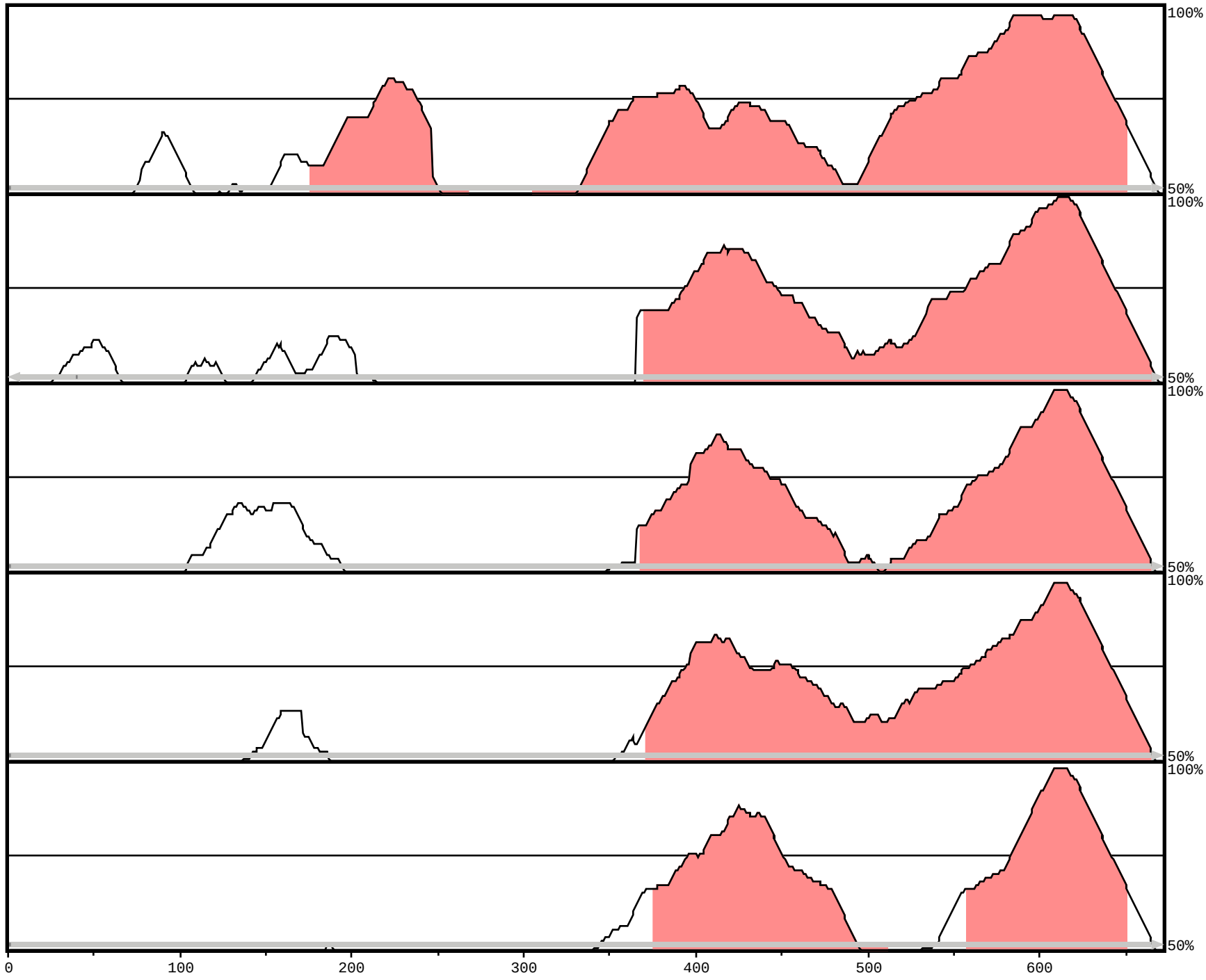

melanogaster S3.21mel:1-511

Alignment 1
malerkotliana
Eip74EF (+)
11898-12391
Criteria: 70%, 100 bp
Regions: 2

Alignment 2
pseudoobscura
Eip74EF (+)
12391-13189
Criteria: 70%, 100 bp
Regions: 1

Alignment 3
willistoni
Eip74EF (+)
15679-17490
Criteria: 70%, 100 bp
Regions: 2

Alignment 4
saltans
Eip74EF (+)
14877-16161
Criteria: 70%, 100 bp
Regions: 1

Alignment 5
virilis
Eip74EF (+)
11025-11668
Criteria: 70%, 100 bp
Regions: 2

X-axis: melanogaster
Resolution: 1
Window size: 100 bp

contig
gene
exon
UTR
CNS
mRNA

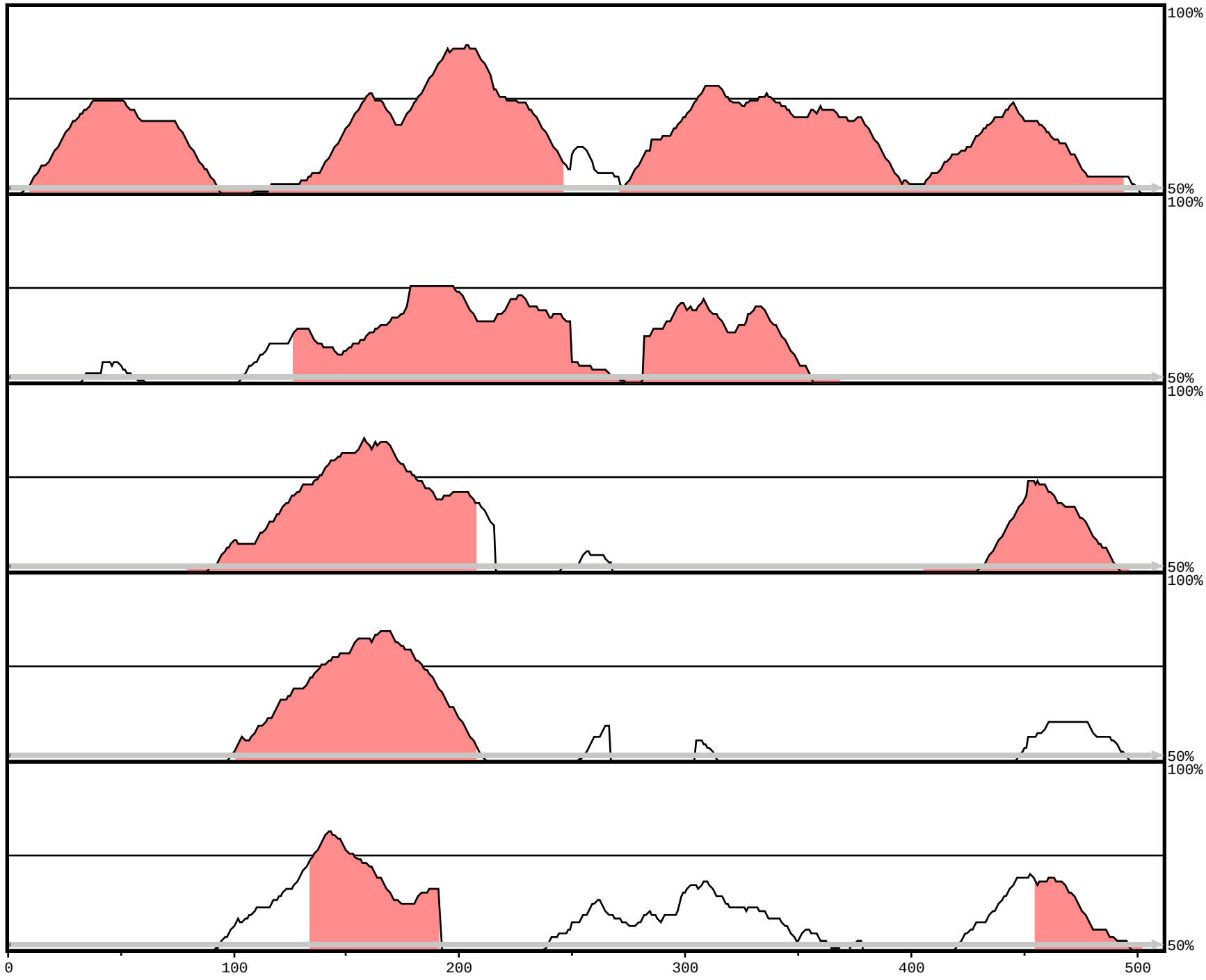

melanogaster S3.22mel:1-991

Alignment 1
malerkotliana
Eip74EF (+)
18916-60595
Criteria: 70%, 100 bp
Regions: 3

Alignment 2
pseudoobscura
Eip74EF (+)
14611-67401
Criteria: 70%, 100 bp
Regions: 1

Alignment 3
willistoni
Eip74EF (+)
1-72102
Criteria: 70%, 80 bp
Regions: 1

Alignment 4
saltans
Eip74EF (+)
774-66766
Criteria: 70%, 100 bp
Regions: 0

Alignment 5
virilis
Eip74EF (+)
6456-65236
Criteria: 70%, 100 bp
Regions: 1

X-axis: melanogaster
Resolution: 1
Window size: 100 bp

gene
exon
UTR
CNS
mRNA

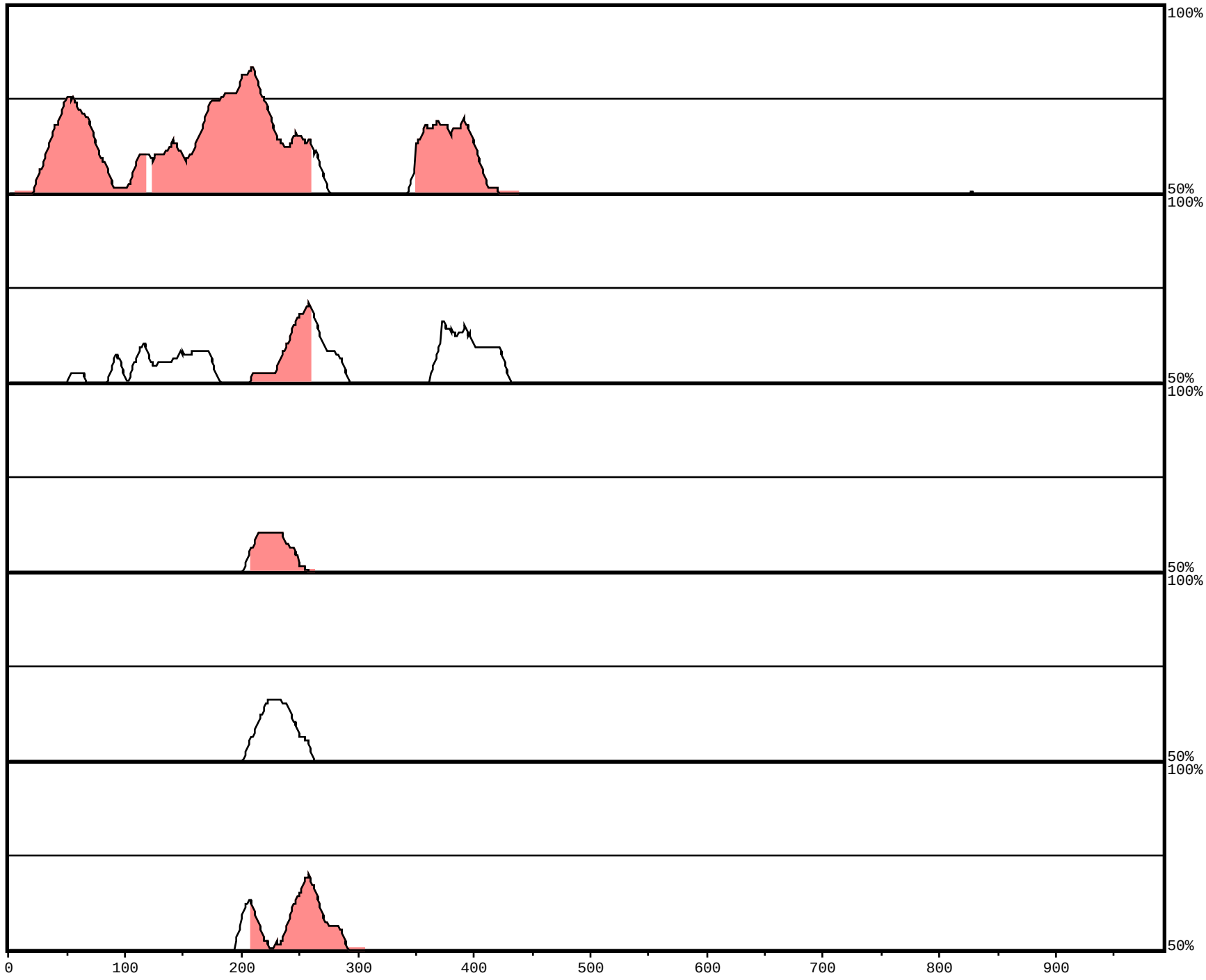

melanogaster S3.23mel:1-861

Alignment 1
malerkotliana
Eip74EF (+)
2 alignments
Criteria: 70%, 100 bp
Regions: 4

Alignment 2
pseudoobscura
Eip74EF (+)
21643-22605
Criteria: 70%, 100 bp
Regions: 3

Alignment 3
willistoni
Eip74EF (+)
28986-29884
Criteria: 70%, 100 bp
Regions: 2

Alignment 4
saltans
Eip74EF (+)
27140-28014
Criteria: 70%, 100 bp
Regions: 2

Alignment 5
virilis
Eip74EF (+)
18636-19487
Criteria: 70%, 100 bp
Regions: 0

X-axis: melanogaster
Resolution: 1
Window size: 100 bp

contig
gene
exon
UTR
CNS
mRNA

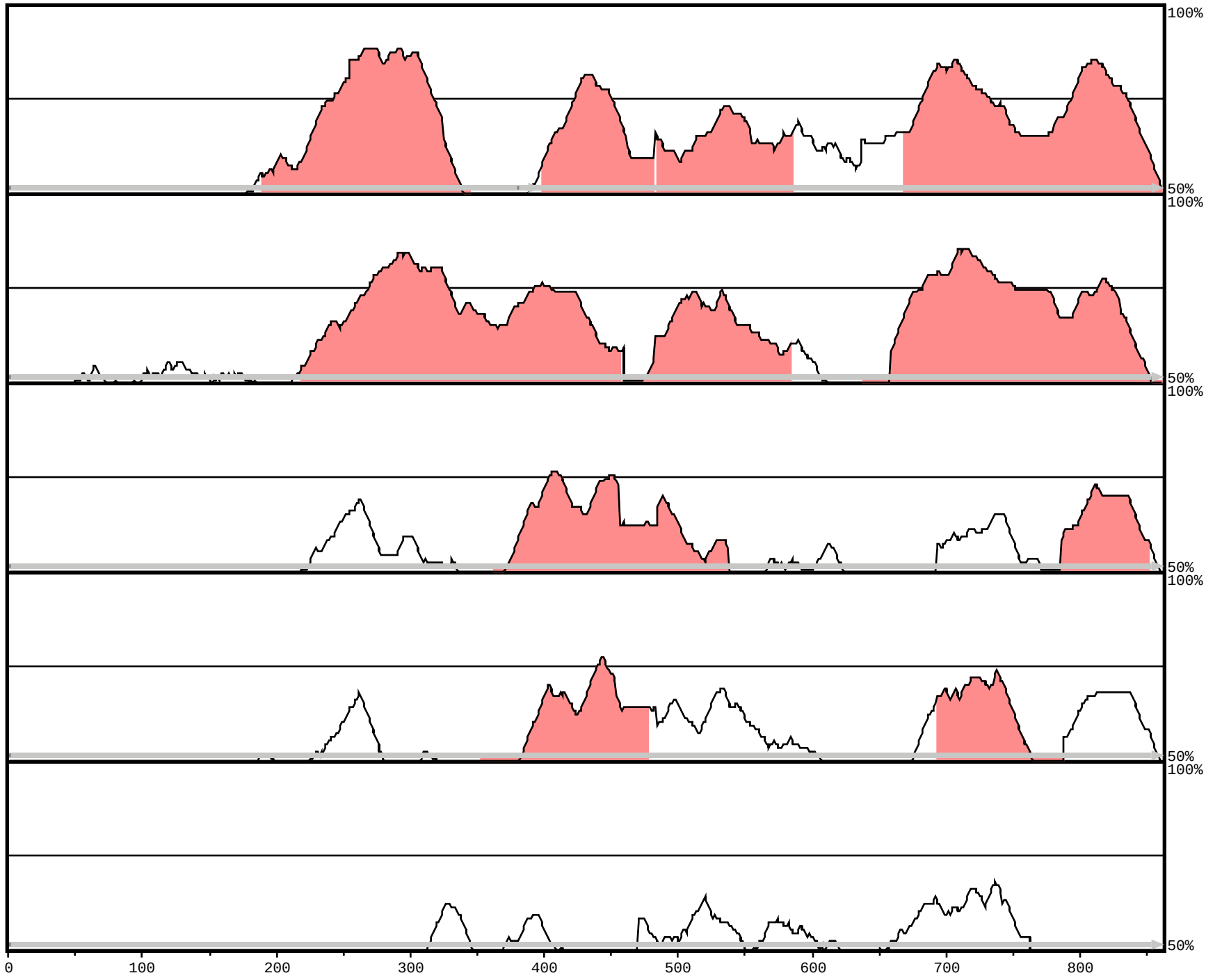

melanogaster S3.24:1-881

Alignment 1
malerkotliana
Eip74EF (+)
31372-32455
Criteria: 70%, 100 bp
Regions: 1

Alignment 2
pseudoobscura
Eip74EF (+)
31143-32081
Criteria: 70%, 100 bp
Regions: 2

Alignment 3
willistoni
Eip74EF (+)
41107-42048
Criteria: 70%, 100 bp
Regions: 2

Alignment 4
saltans
Eip74EF (+)
38470-39384
Criteria: 70%, 100 bp
Regions: 2

Alignment 5
virilis
Eip74EF
2 alignments
Criteria: 70%, 100 bp
Regions: 2

X-axis: melanogaster
Resolution: 1
Window size: 100 bp

contig
gene
exon
UTR
CNS
mRNA

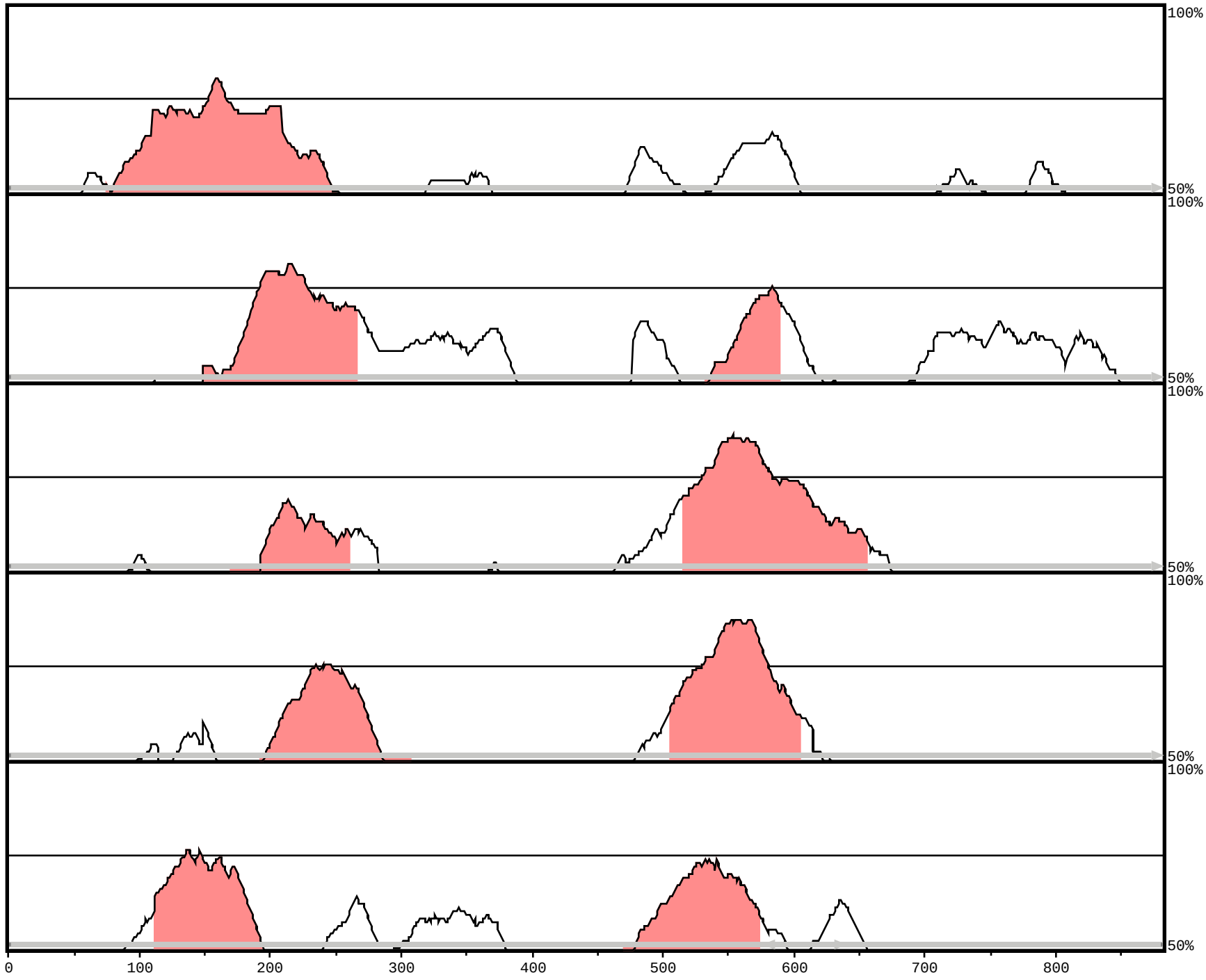

melanogaster S3.25mel:1-731

Alignment 1
malerkotliana
Eip74EF (+)
43822-44610
Criteria: 70%, 100 bp
Regions: 3

Alignment 2
pseudoobscura
Eip74EF (+)
44759-45558
Criteria: 70%, 100 bp
Regions: 3

Alignment 3
willistoni
Eip74EF (+)
57534-58229
Criteria: 70%, 100 bp
Regions: 0

Alignment 4
saltans
Eip74EF (+)
53109-53757
Criteria: 70%, 100 bp
Regions: 2

Alignment 5
virilis
Eip74EF (+)
42221-42897
Criteria: 70%, 100 bp
Regions: 1

X-axis: melanogaster
Resolution: 1
Window size: 100 bp

contig
gene
exon
UTR
CNS
mRNA

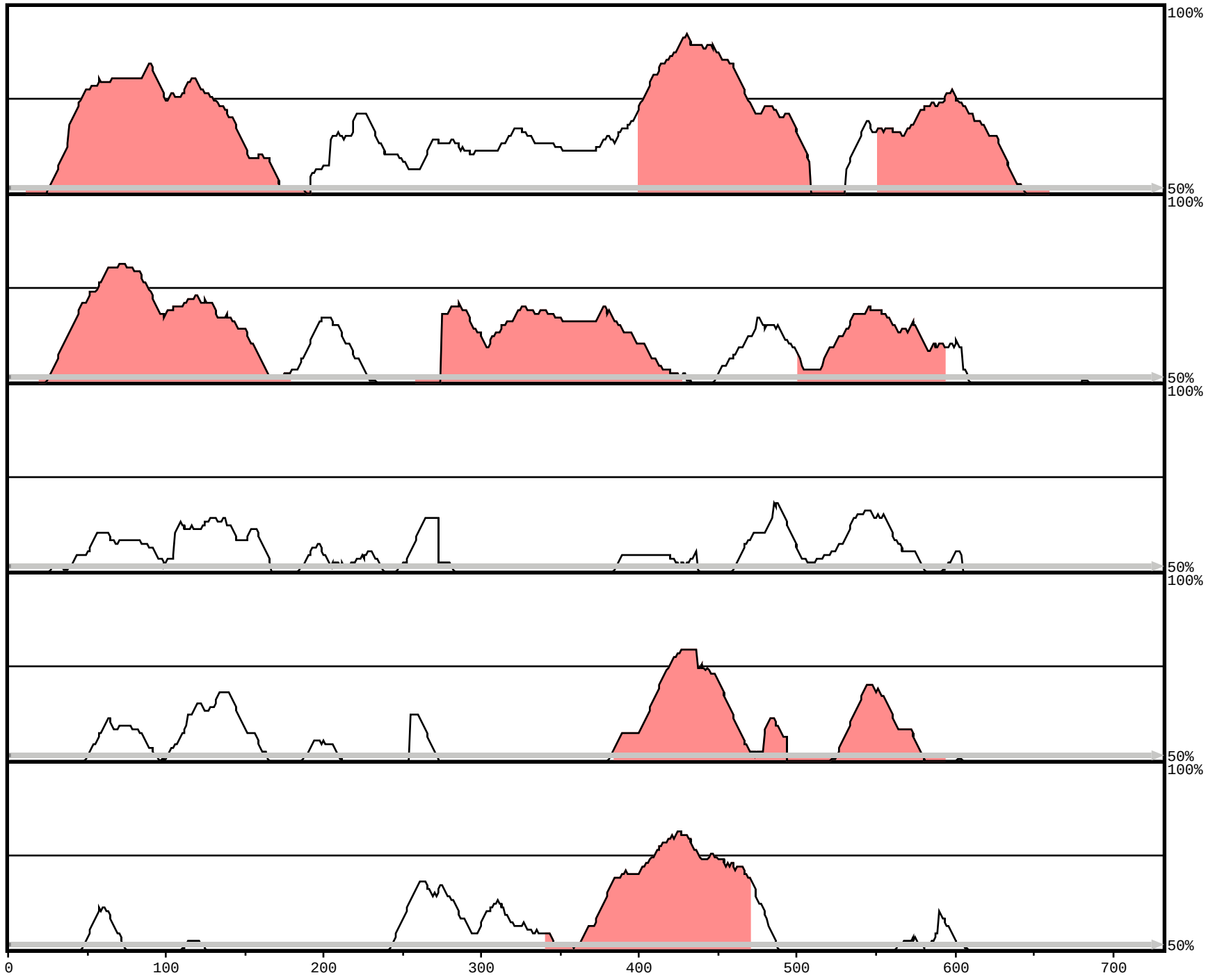

melanogaster S3.26mel:1-551

Alignment 1
malerkotliana
Eip74EF (+)
45119-45660
Criteria: 70%, 100 bp
Regions: 2

Alignment 2
pseudoobscura
Eip74EF (+)
45841-46657
Criteria: 70%, 100 bp
Regions: 1

Alignment 3
willistoni
Eip74EF (+)
59700-60687
Criteria: 70%, 100 bp
Regions: 0

Alignment 4
saltans
Eip74EF (+)
55371-56283
Criteria: 70%, 100 bp
Regions: 0

Alignment 5
virilis
Eip74EF (+)
43833-44503
Criteria: 70%, 100 bp
Regions: 0

X-axis: melanogaster
Resolution: 1
Window size: 100 bp

contig
gene
exon
UTR
CNS
mRNA

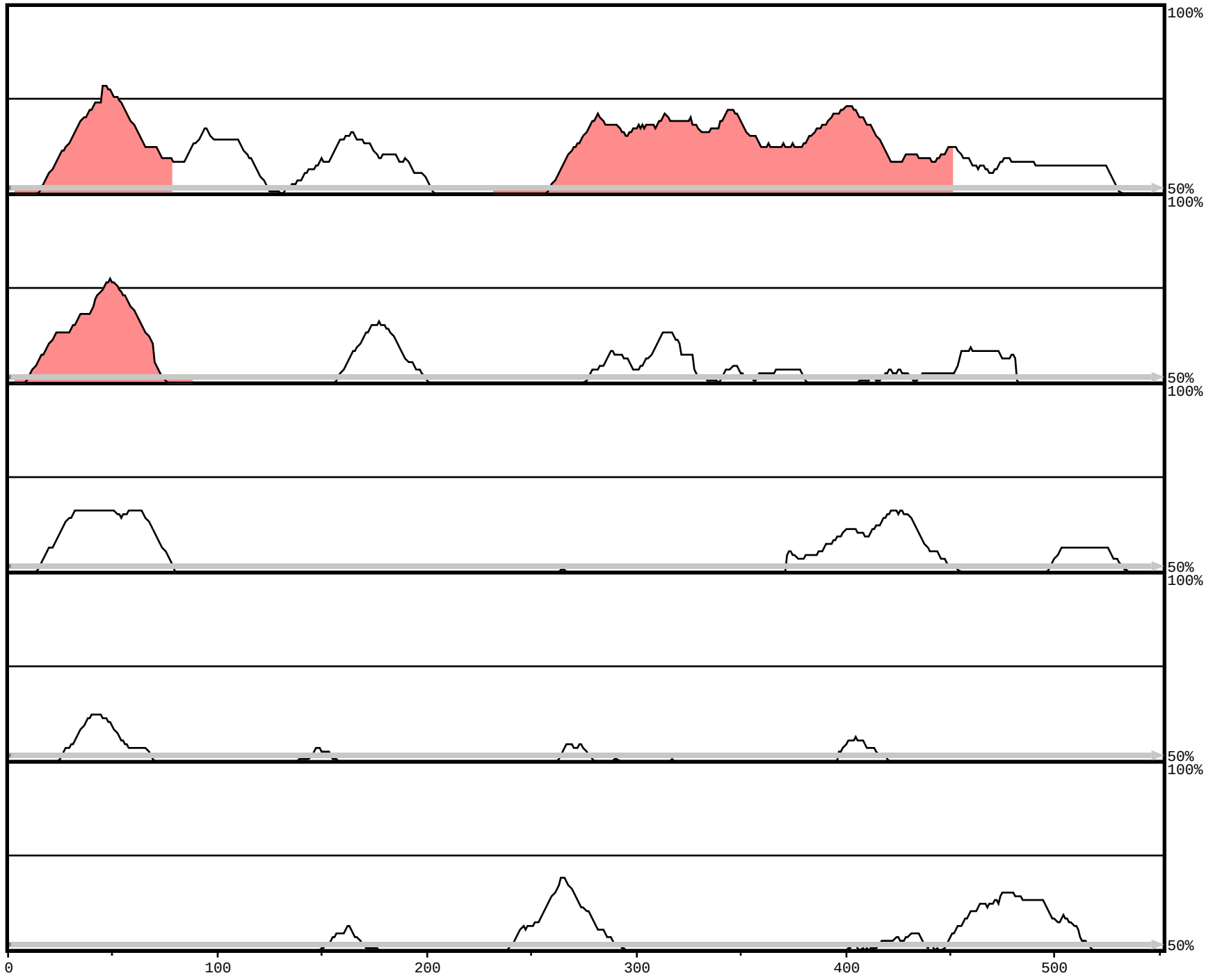

melanogaster S3.27mel:1-761

Alignment 1
malerkotliana
Eip74EF (-)
51464-52280
Criteria: 70%, 100 bp
Regions: 2

Alignment 2
pseudoobscura
Eip74EF
3 alignments
Criteria: 70%, 100 bp
Regions: 3

Alignment 3
willistoni
Eip74EF (-)
69661-70596
Criteria: 70%, 100 bp
Regions: 2

Alignment 4
saltans
Eip74EF (-)
64273-65372
Criteria: 70%, 100 bp
Regions: 3

Alignment 5
virilis
Eip74EF (-)
51944-53023
Criteria: 70%, 100 bp
Regions: 2

X-axis: melanogaster
Resolution: 1
Window size: 100 bp

contig
gene
exon
UTR
CNS
mRNA

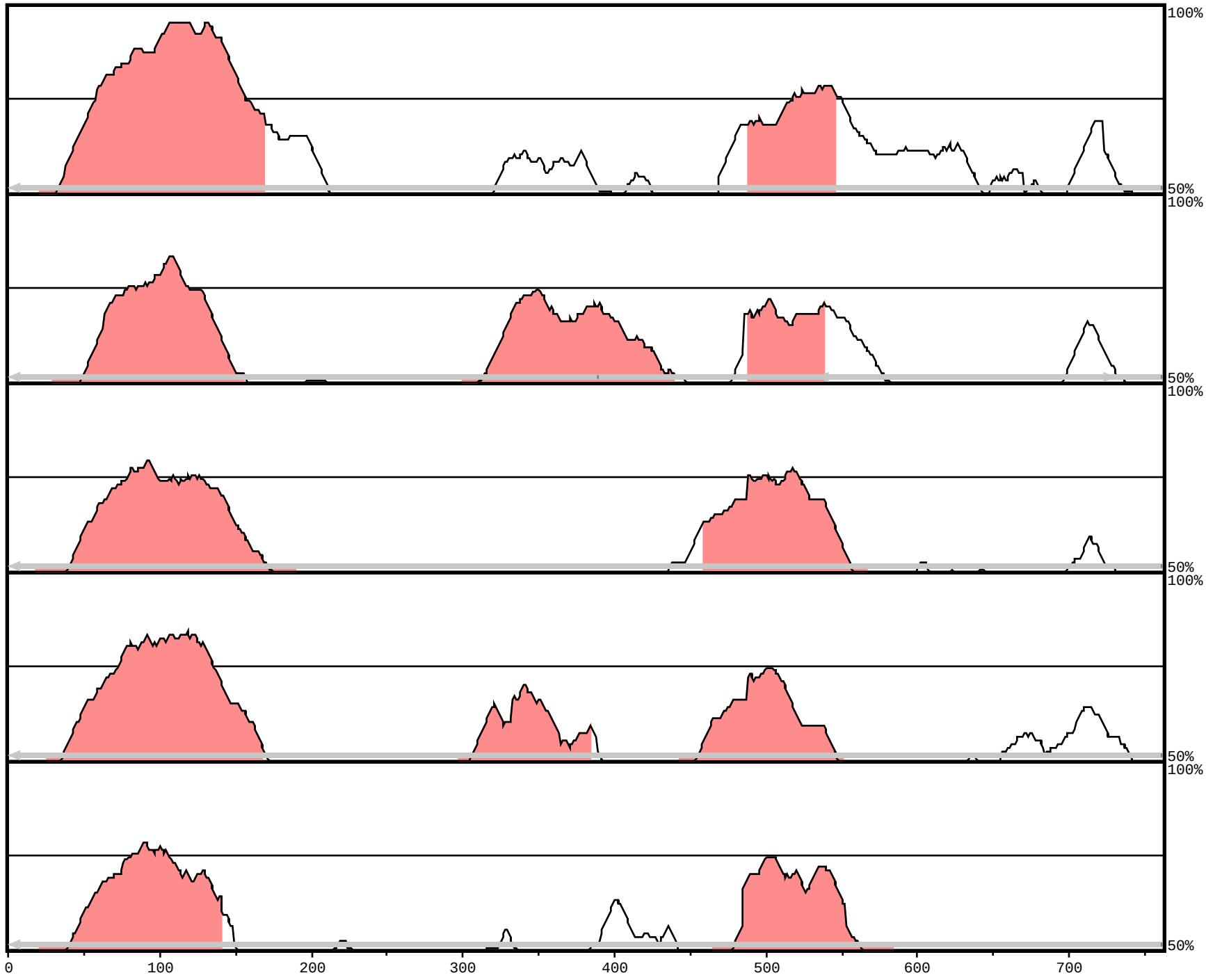

melanogaster S3.28mel:1-581

Alignment 1
malerkotliana
Eip74EF (-)
55387-55936
Criteria: 70%, 100 bp
Regions: 2

Alignment 2
pseudoobscura
Eip74EF (-)
57874-58571
Criteria: 70%, 100 bp
Regions: 2

Alignment 3
willistoni
Eip74EF (-)
75505-76211
Criteria: 70%, 100 bp
Regions: 2

Alignment 4
saltans
Eip74EF (-)
69627-70247
Criteria: 70%, 100 bp
Regions: 2

Alignment 5
virilis
Eip74EF (-)
55240-56166
Criteria: 70%, 100 bp
Regions: 3

X-axis: melanogaster
Resolution: 1
Window size: 100 bp

contig
gene
exon
UTR
CNS
mRNA

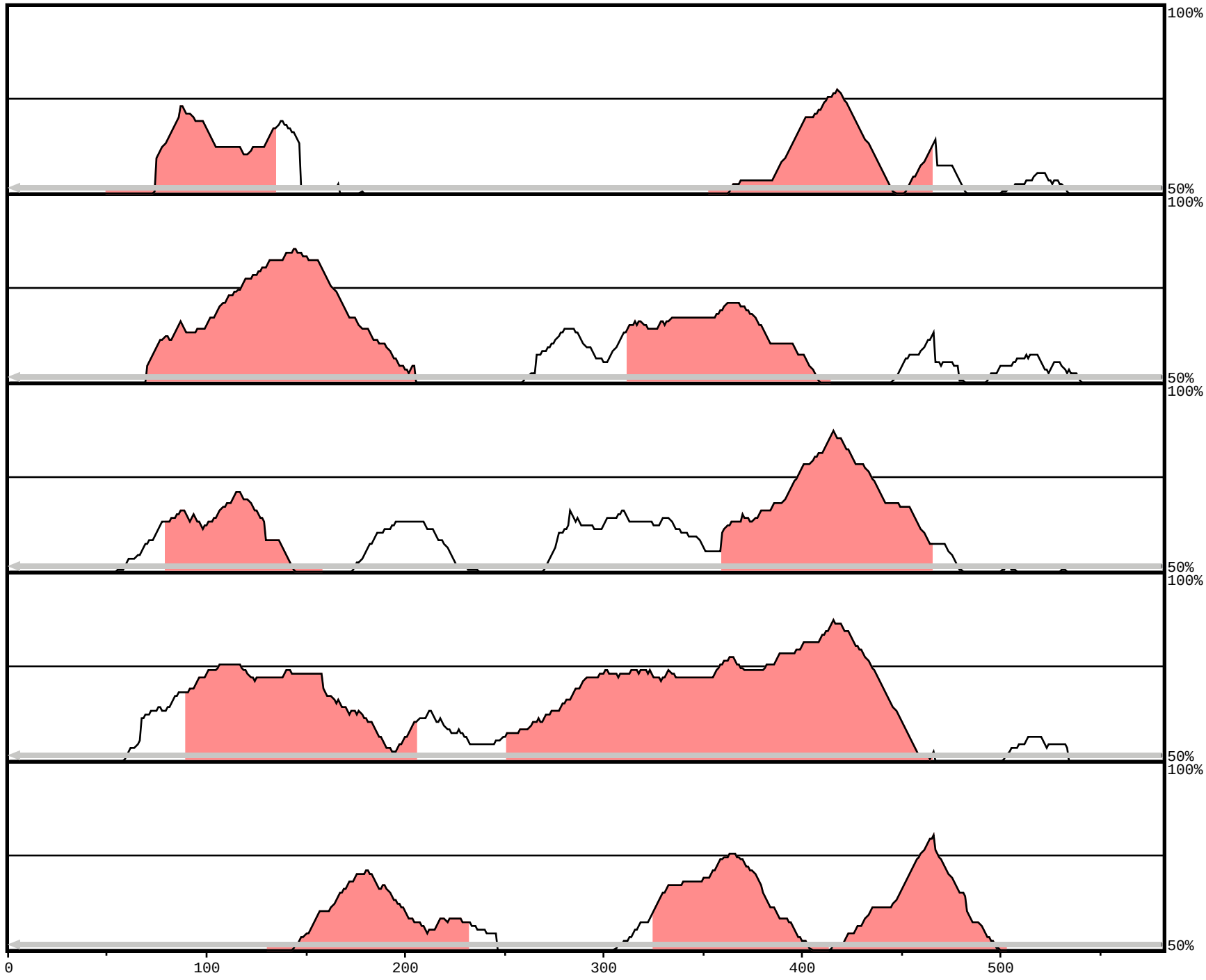

melanogaster S3.29mel:1-921

Alignment 1
malerkotliana
Eip74EF (-)
57256-58140
Criteria: 70%, 100 bp
Regions: 2

Alignment 2
pseudoobscura
Eip74EF (-)
60779-61729
Criteria: 70%, 100 bp
Regions: 1

Alignment 3
willistoni
Eip74EF (-)
78317-79401
Criteria: 70%, 100 bp
Regions: 1

Alignment 4
saltans
Eip74EF (-)
72364-73365
Criteria: 70%, 100 bp
Regions: 2

Alignment 5
virilis
Eip74EF (-)
57569-58445
Criteria: 70%, 100 bp
Regions: 2

X-axis: melanogaster
Resolution: 1
Window size: 100 bp

contig
gene
exon
UTR
CNS
mRNA

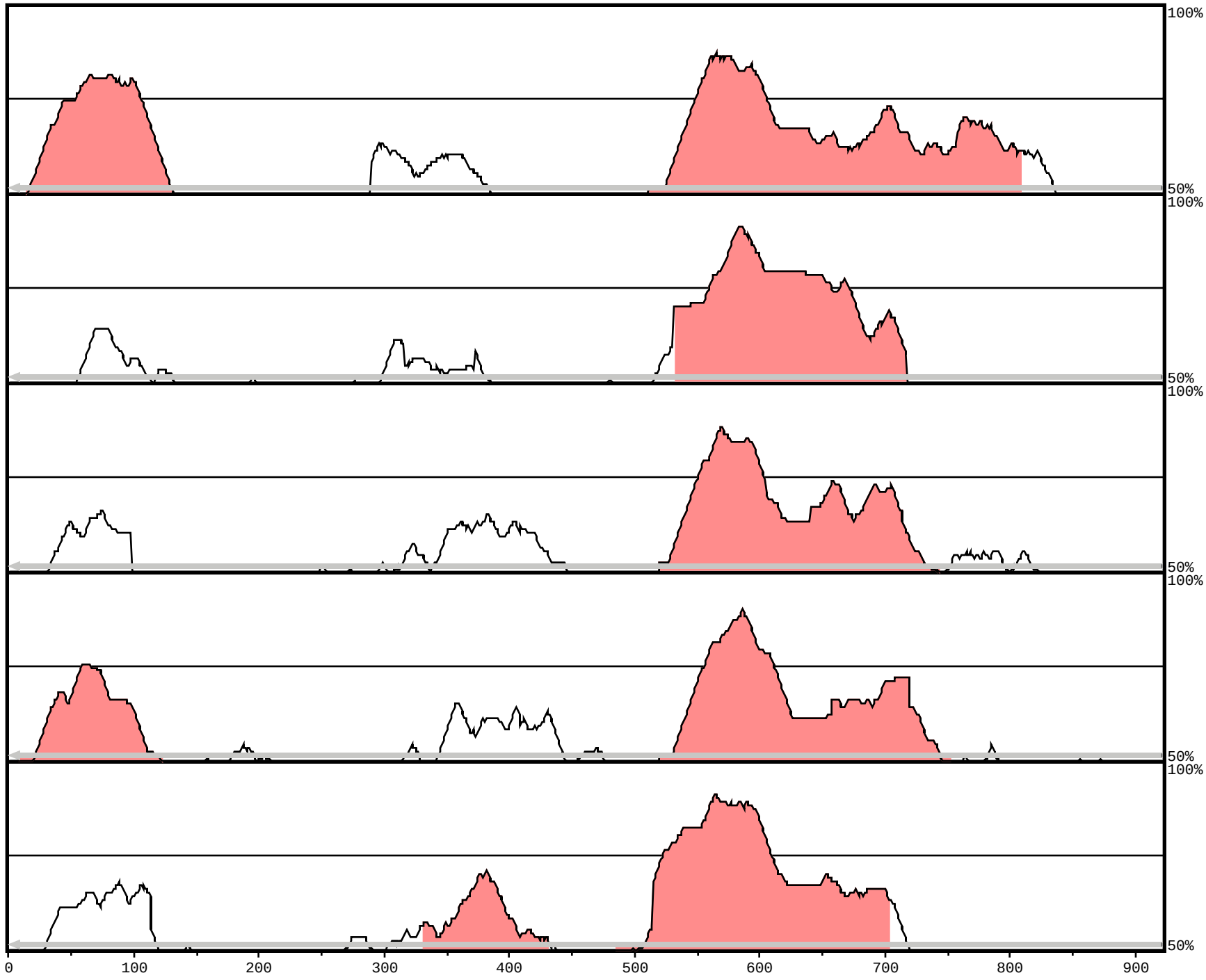

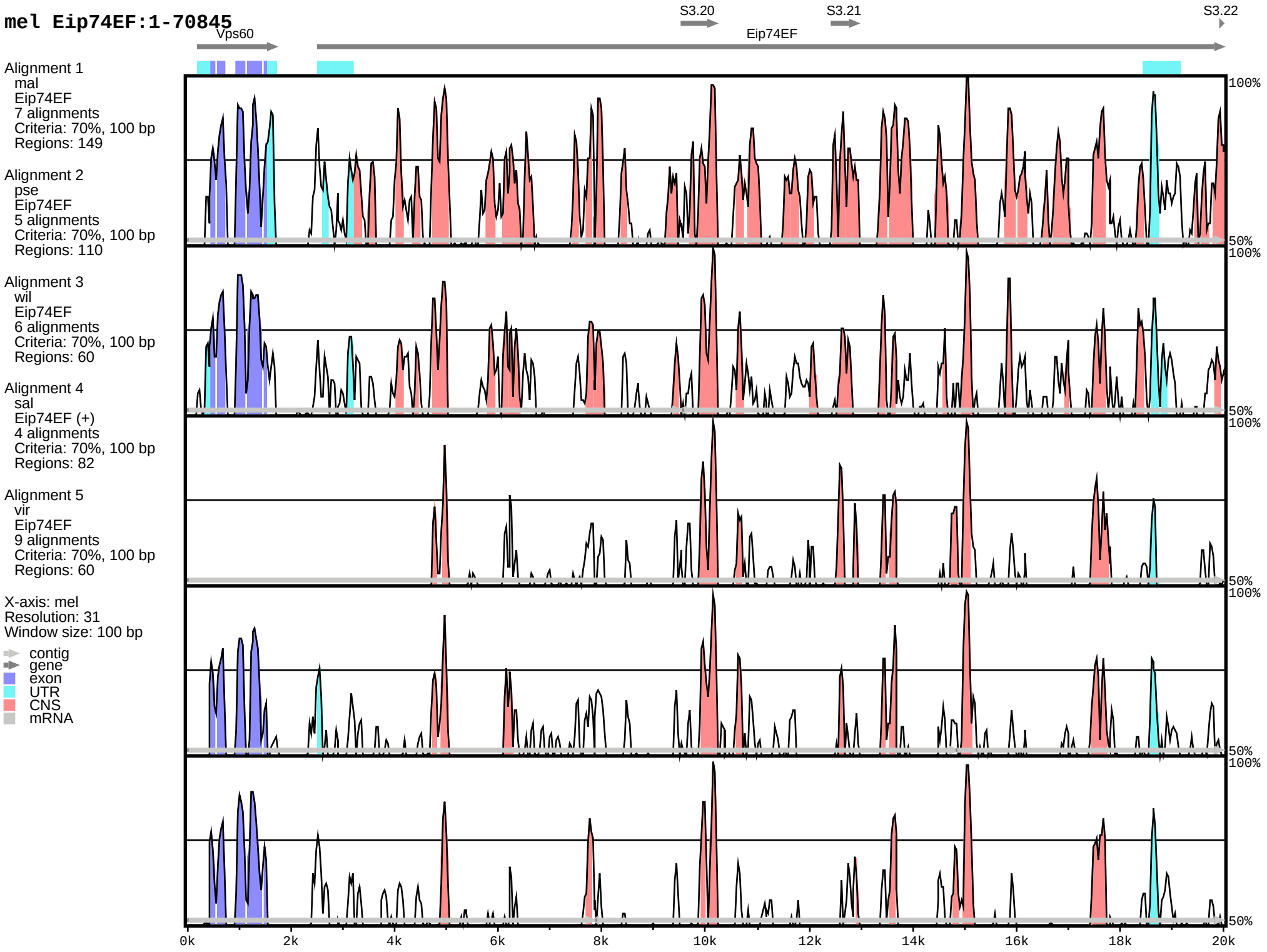
mel Eip74EF:1-70845
S3.20
S3.21
S3.22
Vps60
Eip74EF
Alignment 1
mal
Eip74EF
7 alignments
Criteria: 70%, 100 bp
Regions: 149
Alignment 2
pse
Eip74EF
5 alignments
Criteria: 70%, 100 bp
Regions: 110
Alignment 3
wil
Eip74EF
6 alignments
Criteria: 70%, 100 bp
Regions: 60
Alignment 4
sal
Eip74EF (+)
4 alignments
Criteria: 70%, 100 bp
Regions: 82
Alignment 5
vir
Eip74EF
9 alignments
Criteria: 70%, 100 bp
Regions: 60
X-axis: mel
Resolution: 31
Window size: 100 bp
contig
gene
exon
UTR
CNS
mRNA
100%
50%
0k
2k
4k
6k
8k
10k
12k
14k
16k
18k
20k

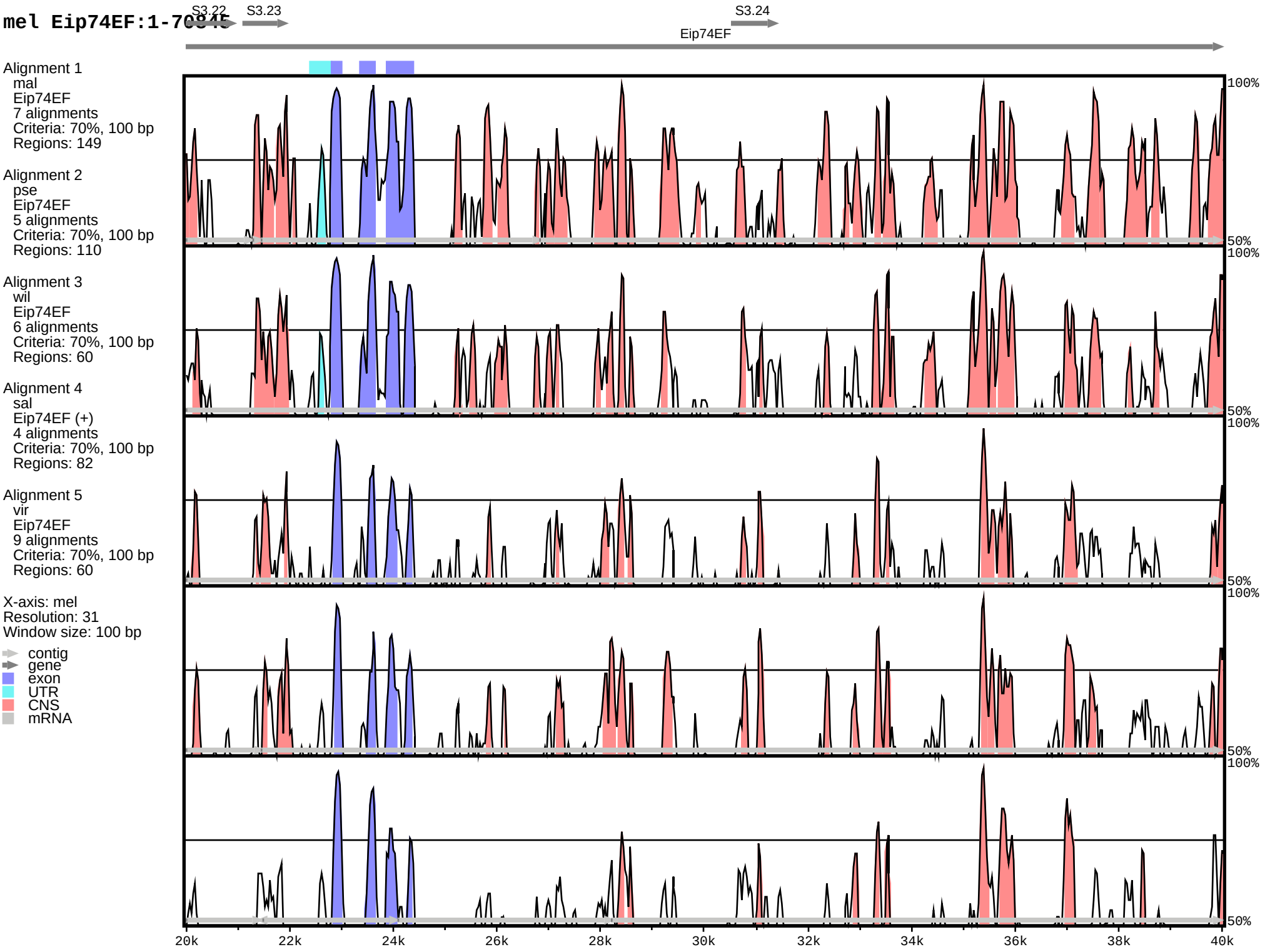
mel Eip74EF:1-70845
S3.22
S3.23
S3.24
Eip74EF
Alignment 1
mal
Eip74EF
7 alignments
Criteria: 70%, 100 bp
Regions: 149
Alignment 2
pse
Eip74EF
5 alignments
Criteria: 70%, 100 bp
Regions: 110
Alignment 3
wil
Eip74EF
6 alignments
Criteria: 70%, 100 bp
Regions: 60
Alignment 4
sal
Eip74EF (+)
4 alignments
Criteria: 70%, 100 bp
Regions: 82
Alignment 5
vir
Eip74EF
9 alignments
Criteria: 70%, 100 bp
Regions: 60
X-axis: mel
Resolution: 31
Window size: 100 bp
contig
gene
exon
UTR
CNS
mRNA
100%
50%
20k
22k
24k
26k
28k
30k
32k
34k
36k
38k
40k

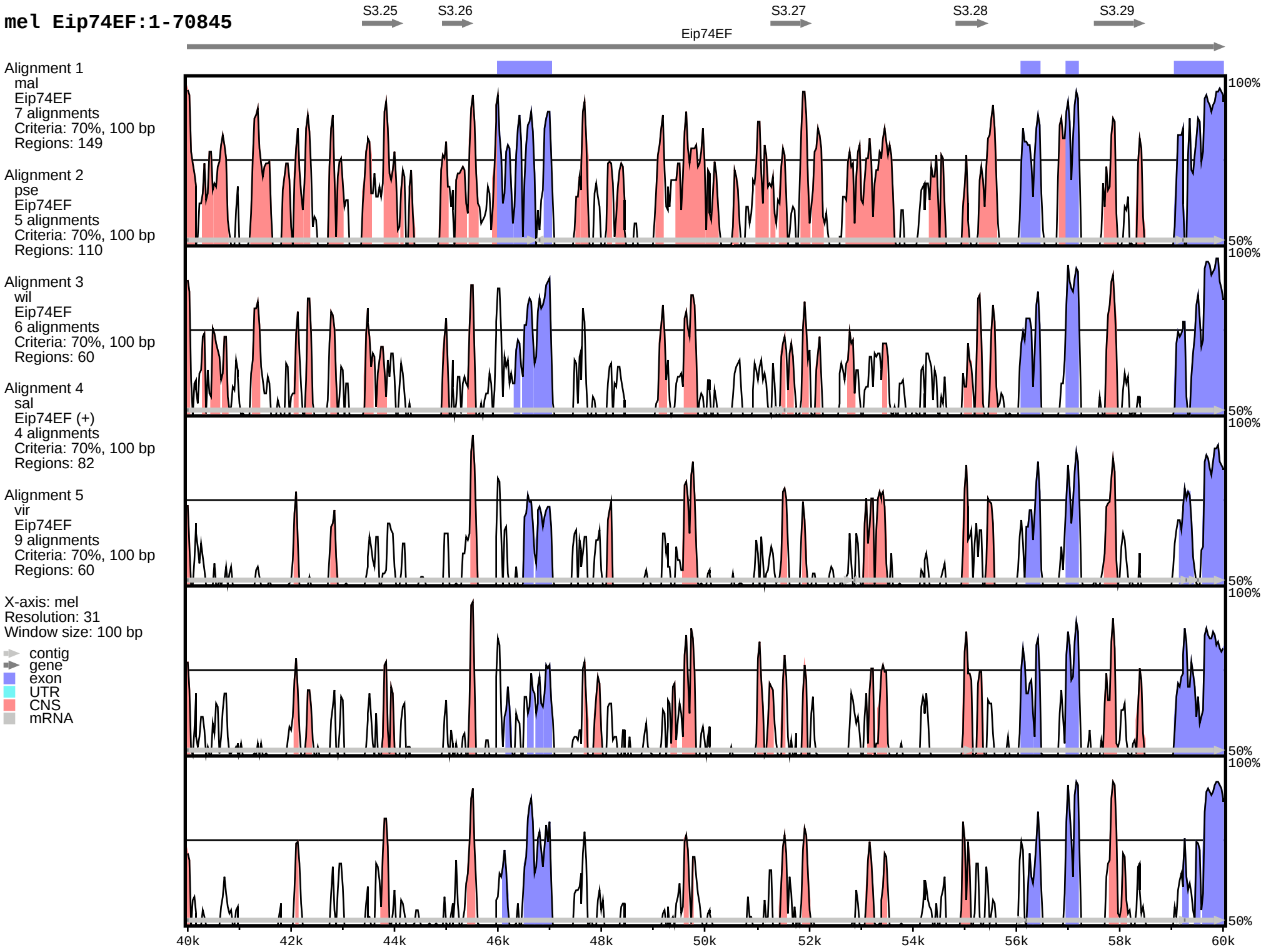
mel Eip74EF:1-70845
S3.25
S3.26
S3.27
S3.28
S3.29
Eip74EF
Alignment 1
mal
Eip74EF
7 alignments
Criteria: 70%, 100 bp
Regions: 149
Alignment 2
pse
Eip74EF
5 alignments
Criteria: 70%, 100 bp
Regions: 110
Alignment 3
wil
Eip74EF
6 alignments
Criteria: 70%, 100 bp
Regions: 60
Alignment 4
sal
Eip74EF (+)
4 alignments
Criteria: 70%, 100 bp
Regions: 82
Alignment 5
vir
Eip74EF
9 alignments
Criteria: 70%, 100 bp
Regions: 60
X-axis: mel
Resolution: 31
Window size: 100 bp
contig
gene
exon
UTR
CNS
mRNA
100%
50%
40k
42k
44k
46k
48k
50k
52k
54k
56k
58k
60k

mel Eip74EF:1-70845

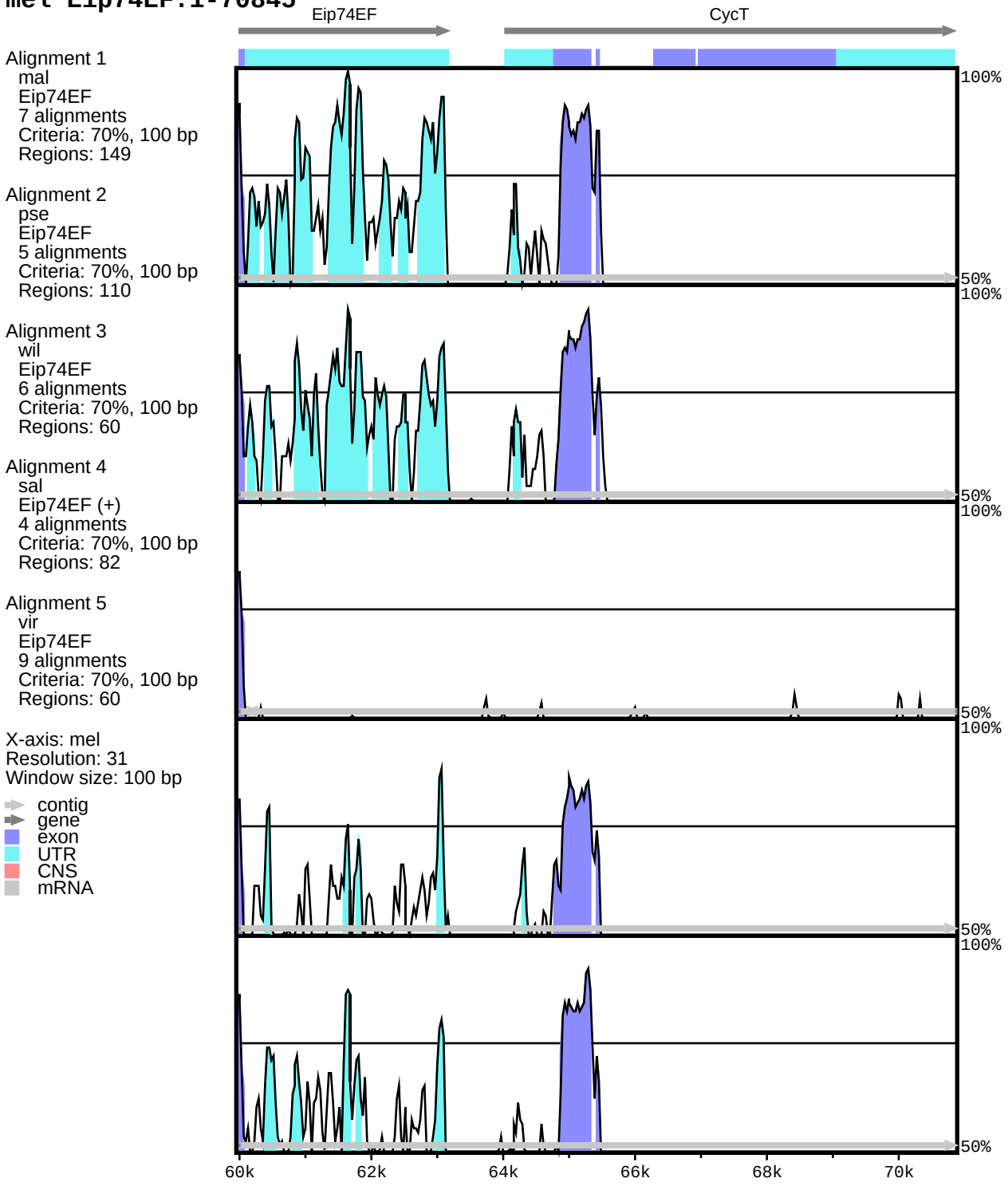

Supplement: msaf213_Supplementary_Data [file msaf213_supplementary_data.zip › Supplementary Document 3 Eip74EF mVISTAs combined (08.05.25).pdf]
