# Supplementary material for: Redundant and Singular Regulatory Elements Underlie the Rapidly Evolving Pigmentation of Drosophila
Source: Mol Biol Evol. 2025 Sep 4;42(9):msaf213. doi: 10.1093/molbev/msaf213 (PMC12449766; doi:10.1093/molbev/msaf213)
Supplement: msaf213_Supplementary_Data [file msaf213_supplementary_data.zip › Supplementary Document S10 Metadata for loci mVISTA alignments v1.docx]

**Supplementary Document S10**

Species: Drosophila melanogaster

GenBank GCA_000001215.4

Genome Assembly Release 6 plus 1501MT

*Eip74EF* Sequence Coordinates: 3L:17551004..17621625

*grh* Sequence Coordinates: 2R:17804227..17843442

*hth* sequence Coordinates: 3R:10675560..10436704

Species: Drosophila malerkotliana

Genome assembly ASM1815323v1

Submitted GenBank Assembly: GCA_018153235.1

*Eip74EF* Sequence ID: JAECXV010000067.1

*Eip74EF* Sequence Coordinates: 6500815-6564924

*grh* Sequence ID: JAECXV010000075.1

*grh* Sequence Coordinates: 15295796-15250755

*hth* Sequence ID: JAECXV010000008.1

*hth* Sequence Coordinates: 662197- 845928

Species: Drosophila pseudoobscura

Genome assembly UCI_Dpse_MV25NCBI

Submitted GenBank assembly: GCA_009870125.2

*Eip74EF* Sequence ID: NC_046683.1

*Eip74EF* Sequence Coordinates: 44804888-54385373

*grh* Sequence ID: NC_046680.1

*grh* Sequence Coordinates: 20241588- 20288299

*hth* Sequence ID: NC_046679.1

*hth* Sequence Coordinates: 7354017- 7642559

Species: Drosophila willistoni

Genome assembly UCI_dwil_1.1

Submitted GenBank assembly: GCA_018902025.2

*Eip74EF* Sequence ID: NW_025814056.1

*Eip74EF* Sequence Coordinates: 4352184-4282074

*grh* Sequence ID: NW_025814047.1

*grh* Sequence Coordinates: 733156- 679437

*hth* Sequence ID: NC_061086.1

*hth* Sequence Coordinates: 29452790- 30192471

Species: Drosophila saltans

Genome assembly: ASM1890357v1

Submitted GenBank assembly: GCA_018903575.1

Sequence ID: JAEIGH010000001.1

*Eip74EF* Sequence ID: JAEIGH010000001.1

*Eip74EF* Sequence Coordinates: 1531694-1612019

*grh* Sequence ID: JAEIGH010000002.1

*grh* Sequence Coordinates: 9355512- 9303931

*hth* Sequence ID: JAEIGH010000009.1

*hth* Sequence Coordinates: 1752413-8864483

Species: Drosophila virilis

Genome assembly: Dvir_AGI_RSII-ME

GenBank Assembly: GCA_030788295.1

*Eip74EF* Sequence ID: NC_091545.1

*Eip74EF* Sequence Coordinates: 8727422-8792657

*grh* Sequence ID: NC_091547.1

*grh* Sequence Coordinates: 9197930-22290270

*hth* Sequence ID: NC_091544.1

*hth* Sequence Coordinates: 22179298- 22455721
