# Supplementary material for: Redundant and Singular Regulatory Elements Underlie the Rapidly Evolving Pigmentation of Drosophila
Source: Mol Biol Evol. 2025 Sep 4;42(9):msaf213. doi: 10.1093/molbev/msaf213 (PMC12449766; doi:10.1093/molbev/msaf213)
Supplement: msaf213_Supplementary_Data [file msaf213_supplementary_data.zip › Supplementary Document S4 synthesized melanogaster Eip74EF sequences v2.docx]

**Synthesized and subcloned *D. melanogaster* *S3.20* *Eip74EF* CRE. Sequence is flanked by *Asc*I and *Sbf*I restriction sites that were used to subclone in the same sites of the S3aG reporter transgene vector.**

***Asc*I**

**ggcgcgcc**CACGCACACACATGTGTGTTTGCATGTGTGTGCGTATGTGAGCGCTGGCGTAATGAAAACAACTCCCGCGCTTTTTCGCGCCCGCAAGAGAGCGACAGTGAAAGAGAGATGCTAAATTTAGTTCAATGAATTCGAATTTCACCCAGCTCTTAGCAGGCTCTCACTCTCCATCTTGCTCTCTCGCGCTTTGTTTTTGTTTTGTTGTTATTGTTGGGGCACAGTGTATTGACTACCGCAGAGAGCAAAGCACGTTACTCCACTC

GGCACCACTCGGCACCACCCACCACCCCCTCTGGACGCATCGCACTCTGCGATGATGATAATGACGTTGCCAGCGGCTGAGTTTCTGCAACTGCAACAACGACACGATGATGATGATGATGACGATGGTTATCGAAGGTCTTTCACCCGAACTCGGTCATTGGCGTACATTTGTGTGCTGCTGCTGCTTCAGCTGCGACGTCGCTGCCACCGATGATGTTGCTGTTGTATCTGCTGCTGGGCTTCTGCTTGTGCTGCCGCCGCCGCAGCAACTGCAGCTCTGCCTTACTGCCGCCGCTCGCAAATCCAGAGCTTTTCAAAGTGTACATGTCGCAATTTCTTAAGAAAGTTCAATTACCCCAAACAATCAGATTTACTGTCTGTTTTTTTGATGCATACACTCGCTGGCC**cctgcagg**

***Sbf*I**

**Synthesized and subcloned *D. melanogaster* *S3.21* *Eip74EF* CRE. Sequence is flanked by *Asc*I and *Sbf*I restriction sites that were used to subclone in the same sites of the S3aG reporter transgene vector.**

***Asc*I**

**ggcgcgcc**TTTAAAATCAAAAGTGAAATTCCCCATAAAAGTTACTCAATAAAACGAAATTATTTTGCGCTGCGCTGCATTTCTTGTGGCAGTTTGCACGAGTTCAGCGTGTTGTTGCTGCTCTTGATTTTTGCGTTATTTTTTTTTTTTTTTTGTTTTTTGGGTGCATTAAACGGCAAATTGTTTCGTGGGATCAACACAAAGTGCGTTGATCTCTCGGCTGAAAAAGTGAATGAATGCAAACGGTGACGTCGCGACGCGACTGCGCTGCGCTGCTCTGCTGGCGTGGGCGTCGCTGCCTGGTTACGTTTGGCGGCGGCGTGAAAATAGCTCGAATGTGACTAACAATTAATAAATTATTAATTGCGTTGAATCATGAGTCTGTGCGCTGTGTACTTTTTACTTTTTGGAGCAACTCCCGATCGCCATCTCACTCACTCTCTCTCTCTCTCTTTCACTCACTCACCCATTTCACTCCACTCGATGCGAAAGCAAAAGCAAAACAAAAATTCATCGGCCT**cctgcagg**

***Sbf*I**

**Synthesized and subcloned *D. melanogaster* *S3.22* *Eip74EF* CRE. Sequence is flanked by *Asc*I and *Sbf*I restriction sites that were used to subclone in the same sites of the S3aG reporter transgene vector.**

***Asc*I**

**ggcgcgcc**GAACGGCGAACAGTAAACGGCAAATGGCCAACTTGGCGCCAGCTGAAGCTAAAAACAAGGCTGGACGTTTTCCATTGGCTTTTCCTTTCGCGCTTCTTGTTTGTTGTTGTTGTTGTTGCGCAGCTCTTTCGAGTTTTCTTTCGCTTTTTCTTTTCGCCTGAACTTGCAGTTTGGCTGGAAAGTGAAATAGTTGCAGCCGCCCGAGTCGCTTGTATTGAATAATGAAAAACGAGTTTTCCGCCGCGCATGCGCATCATAATGG

CAATACCAACAGCAAAAGCAACAACAACAACAACAGCAACAGCTAAGCTGTGGAAAACCCGAAGAGGCCGAAAGAAAAGTGGCGATAGGTAGGGAGAAAGAGAAATGATGATGACGCGGGTGCCAGGCAGCGCAATGTTGTAATGTCTGTGCTGCAAGTTGAATGTGTTGCACTGCGGCCATTGATGTTGCTGCCGTTTCTAATGCTCATAATGCTGATGCTGCTGCATGTTGCATGTTGCGTGTGTTTGTTGCTCGTGTGCCGCACACAAAGTTGCTATTGTTTTTGTTCTTTCGACTTCTAATGCTAATATCTACATGATATACTGCTTCTGCAAAGATGTTTAATGCTTTCCTTTATTTATTGCTTGTATAGCAGATGTAATTTCTTTTATAAAATGCTATGGATTCAGATGAATTTATTAGAGTTTTGTAATTGGGTTGTTATTATTTCTTTCGGCTCTTTACTCCTCCTATTTAGTAAGTCTCGTATCTCTTAGCTTCTTTCAGTTCGGATAGTTTATTTTCACTTAGTATAGACTAAAGAATTTGTTTAAGGTAACATTTATAATAATGTTATTCTCATTTTGCTTAGTTATATGTAATATGTAATAACAGTCATATTTAACAGCTGAGATAATAAACGAAAGATCTGTTTTTTTGTTATTGATAATAGTAGATATCTTATTTGTTCCCACTTTTTGTGCTTTATTTAGTCGTAAACTAATGA**cctgcagg**

***Sbf*I**

**Synthesized and subcloned *D. melanogaster* *S3.23 Eip74EF* CRE. Sequence is flanked by *Asc*I and *Sbf*I restriction sites that were used to subclone in the same sites of the S3aG reporter transgene vector.**

***Asc*I**

**ggcgcgcc**TAGCTGAAGGTCTTTAAAACTAATTGCCGATACTGCTCCTTGTCTGAAGTTATTAAAATAAGATTGCAAGTCTATTGTTACTTTTTTACGGCTGCATGTTTAAGTATAGCAGATGTTGTTGTTTCTCCAGTCTCCTATTTTATAATATGTTTTTTTTTCAGCTAACGCTTGCACCTAAAGCTGAAAATGTTGTTGTTGTTGTTGTTGGCGAGTGCTTTCAAGTTTGCTGCAGTTTTATTTTTTCCTATGCGCATTTTTCTCTTCGTATTCCGCCTCGTTTGCTTCGTAGTTCAATGTTAACGAACTTTTTGCGCCGTGAATGCAGATTGTTGTTGCAATCTGCAGTTGCAGTTGCAGTTGCTGTTGTTGTTGTTGTTGCAAATGGTGCTGTTGGTGTTGCTGTTGTTTTTGGACTTTCACTTGTAATGAATTTGGCGAGTGCAAAACTTGTTGTTGCTCCATGCTGTTGTTGTTGTTGTTGCCGCAGTTCAGTAACATGTTATGTAAAAAGCGTTTTTCCAGCTTTGCTTCTATTTTCGTTTTTCTCCTGCTTTTTTGCACACTTTTTTTTTTTTTTGATTATTTGTATGCAGTTTTCAGTTTTAGCTTGCCACAAAATTTGTGCCGCACGTCTTGCCCCGCCGCCGTGGATGCAACTTGCCCCGATCGCCGAGTTTTCTTTCCGCTCTTGCTTTTATGTTGTAATTCGCAATTTCTAATGTTATTGAACTTTAAACACAAACACAATCGCAAAACCGAAAAAAAAGTTGCGGCGCGCGGAGGAAAACTTTGATTCAGAGGAAAATTAGTTATAACCGGAAGCCACAAGGCAAAAATTGAAACAATGCAACGAGACGACTT**cctgcagg**

***Sbf*I**

**Synthesized and subcloned *D. melanogaster* *S3.24 Eip74EF* CRE. Sequence is flanked by *Asc*I and *Sbf*I restriction sites that were used to subclone in the same sites of the S3aG reporter transgene vector.**

***Asc*I**

**ggcgcgcc**CTTTAAAATTAAAAAAAATGCATATAGTATTTATAGACTTATGGCCCGGCTTTAGGCCCTTTGGGTCTTTAAGACCGTCCCTGAACTAAACCGGGCTTCGGATCCAGTTTGCGCTGGCCCTTTGATTGCATTAGAGCGTGTGCCGCCCGTCGCATCTCGTTAAAGGCAGAAATGAAAACAAAAGCAGCGGCAGCAGCAGCAGCAACATCAGCAATGATGATGATACAACCTTACAAAATTCTCAGTCGCAGACTGCGGTGCG

ATCCAAACCCCCAGCCGCACCACCCCCTCCAGCCACACCACCCGCTCTTGCCCATCGCAGCGACTTTGAATGTTTAGCGCTGAACTTGTCTTGCACACGACCACAGATGCAGTTCGCGCGAGAGAGCGAGAGCGCGCCGCAGAGAGATAGACGGCGAGATAGTGATAGAGAGTGGTGGGGGCTCAAGCAAAGGCAATAGCAAACACACGGAGAGGCGCAGCGAGAGAGAGAGAGAGAGCGCGACAAACACAGTGATAGCTTTATTTTTGCCGTAGCGTGTGTGGTATGCAGCCTTGAATTAGTTCGCTGCACCCGCCCGTACATACACACAAACAAACAAACGCACACACGGCGTATATAAATACACGCGAACCCCACACACACACACACGCACACATGGGAGCAGTGCCATATAAGCGTTTCGCAGTGTGCGAAAGCTCTGTGTTGCTCTGCTCTGCTCTGCTCGTTGCTGCTTTGCTCCCCTTTTGCAATGCTCGTCGATCCCCTCGCCAACCCCAAAGCCCCTCCCCCTGTCACGCCAAGCCACCCAACACCCCATGTCATCCCAATCGAAATCGTAGAAGGAAAGAATCTACTGCGGCGCTATAAAGTACATATA**cctgcagg**

***Sbf*I**

**Synthesized and subcloned *D. melanogaster* *S3.25 Eip74EF* CRE. Sequence is flanked by *Asc*I and *Sbf*I restriction sites that were used to subclone in the same sites of the S3aG reporter transgene vector.**

***Asc*I**

**ggcgcgcc**GAGTGCACCCCTAGTTGTGGGTACATTGGACTCATTTCACACACACACTTTTGCACTTAGACGGGCGCACACAGACAAAGAGCTATTGCAAAAGTATGTCTGTGTCGACGTCGCCTCATTTAGCTTCTCCTTCTTCTGCCTGCTGCCTCCTTTTCCGCCCCCTGCCCCATGCCCCCTAAAATCCGCCAACCCACACCCCTTGCGTCTACGCCCTCGCTGTTTCTAAAAACAAAACTCTTCCTTCTGCCGCTCCTCCGTCTTCTTCTTCTTCTTCGCCGTCTACTTGCTCTGCTTCCTTTATTTGCAACATACATTATATGCCAACGGAGGGAAGAAGAGAGAGAGAGAGGGAGAGAAAAGCGGCAAGCGGAGCTTCCTTCCCACTCGCACACTTGAATTGCAATCGGCAAAGTAAATGGAAAGGCCCCCAACAACACTAAGCAAACGGAAAGTGAAAATTTGCTCCACTGACAGACAGCTAGAAGAGAGTGAGAGCGAGTGAGTGAGAGAGAGTGCTGCTGGCACTCTCACAAAGACAGAGAAAAGTGCTTACATACACTTGCATTGAGTCATAATCGTGTCGATTGCAGCAGCGACGTCCGCTCTCCTGCACCTCTTCCCCTTCCTCATGCTCTGCAGCCACCCAATCTGCTCCCTCTTGTCCTTCGTCCCCTTTCGCTCATCCCCCGTTTCAGTTCCTCTTCGCTGTGCAAAAACACAGACCAGATGAT**cctgcagg**

***Sbf*I**

**Synthesized and subcloned *D. melanogaster* *S3.26 Eip74EF* CRE. Sequence is flanked by *Asc*I and *Sbf*I restriction sites that were used to subclone in the same sites of the S3aG reporter transgene vector.**

***Asc*I**

**ggcgcgcc**AATCCCCTCGAGAATTGCATGCAAATGCGCATGTGGCACACAAATCCCCCAAACATGCACACACACACACACACATACCGGTGCAACAACGAGGTGGTGGGGGTGAAGAGGAAGACGCAGCAGAGAGAGCTCCAAACATTCGCGTGCTCTCTCGCTCTCCCCCTGTCTTTGCCCCGCGATTACGTTTGTTTGCCATTTTTGCGCTCTCTCGCAACGTTTTTCGCAACACCAGCGGCAGCGGCAGCAACGTCGTGCCGTCGATTCAGTCGTTCAGTCGGCGTAAGCGAGTCGAGCGAGCGAGCCCCATTCAGTCCGGCGATTTGGCTCTTTTGCACCTGTGCAAGTGTGTGTTTCTCCCGCGGTGCGTGCGTCCGTGTAAGTGTGTGTGCGTGAGCGGTGGAACTGGTGCACTGGTATTGGGGCATCGGTGAGTGGGGCGCGCGCGCGCTTTTGCAGCCGCAGCATCGCAGTCGGCGTCTCTGCTGGCGCTGACGTCGAAGCCGACGTTTGTTGTTCGGTGTTTGCGCGTAACAAACACTCGCAGCGGTTTT**cctgcagg**

***Sbf*I**

**Synthesized and subcloned *D. melanogaster* *S3.27 Eip74EF* CRE. Sequence is flanked by *Asc*I and *Sbf*I restriction sites that were used to subclone in the same sites of the S3aG reporter transgene vector.**

***Asc*I**

**ggcgcgcc**CAAGTTAATTCGAATGCAGCAGCGACAGACGAAAGTAAAATGCACAAAGAGTCGAAAGCAAAATCAACAGGAACGGGCGACAACAACGCAAAAGTTCTATATTTAAATATTATAATTTATGTGTTTGTTTGGCAATTGTAGTATTTGTTGTTGGTGTGAATGTCTCTTCCCCAATTTTTTATGTGTACGTACTACAAATATATTTTTTTTGGGTTCCGTCTGCCTCGCTTTTGCACGATCCCCATTATTCCCAGTCCCATTCCCGTTCCCGTTCCCATTCCCATTCCCATTCCCCTTTTTTCTCTTCCTCTCTCTCTCTTTCTTGGCGGCTCTCTGCTGCTCTCTAAATTCCTGTTCTGCCGCCTTATTTTGGCTTTTATGCCGCTGCCAACTTCGCTGCTTTCACGACTGCCTGTGTATTGGTGGCCAGAATAAAAGCCAGTTGTTGTTCTCTCGCTTCTTCGGTGCGCGCGTATGTGTGTGTGTGCGCTTTTTGGAAATTCAGTCTCAAGTTCAAGGTCTCGCGGATAACGCACACCAGCAAAGACACACTGCTCACACGGACACGGGATTCTCATTGTTTACGCACACAGGCGCAGGCAGCGAGACATATGCATATCAGGCAATTTCTCATTGTCGTACGAAAATGTGTGTTTATTAGGGCTACATATGCAGTTAATACAAGTCCACACACAAACGCACATTATTCGTATTTATATAGACAACAGAAATTCTTCTTCAATATTTCCGCATTAAGAAAT**cctgcagg**

***Sbf*I**

**Synthesized and subcloned *D. melanogaster* *S3.28 Eip74EF* CRE. Sequence is flanked by *Asc*I and *Sbf*I restriction sites that were used to subclone in the same sites of the S3aG reporter transgene vector.**

***Asc*I**

**ggcgcgcc**GAGCCATGAACTCGCAACCGTTAAAAACAACGCCAGAGGAAAGGCAACAAAAATTAGCTTCTCACTTTCCTTTGGCCGTTGTTGTTGCAGCTGAAAAGTAAAAGTGCATTTTCACTTATTTCATGCAGCGGCAGCACTGGCAGCAGCGGCATGAAAATAAGCAGAGGCAAAAAAAATTGCATTGAATGTCAAGCGGTACGCACACACACATACACACACACACATTCACACGAATCAAATCCGCGGCAACTGCAGTGTGCAGGAAAACGTCAAAGCCAGCGGAGCAATGAAGCTTGCAAGCACACTCACTCTCACAGATACGCGCACACACACAGCCACGCCAAGCACTCACACACACAAGCCCAGACGAGTGGCAGCAGAGAGGAAGAGAAATCAGGTCAAGGCTTGTCGGCAACGGCGACGAGTTTACAATTTATGCTTTGCTGGCTGCATTATTGCATGTTGCCGCTGTGTGCGAGTGCTTTTTACAGCTTTTTAAATACACGGCAGCCCGGCCGACTTCGCACACTGGCCGATAATGCCTCGTAAAGCGGTGTAAAACTAACCATTTTCCATATATT**cctgcagg**

***Sbf*I**

**Synthesized and subcloned *D. melanogaster* *S3.29 Eip74EF* CRE. Sequence is flanked by *Asc*I and *Sbf*I restriction sites that were used to subclone in the same sites of the S3aG reporter transgene vector.**

***Asc*I**

**ggcgcgcc**CGCAGATCGCTTGCGACTATTTCACACTGTTTCTGTTTCTGTGTGGTGGCACAGGTATAATTTTGTATGCTATTTAGTAAGTTTTTTTTTTTTCTTTTTTTTACATCCGGCGTTGGCGTTGCCCAATTTCGCGGCTCAGCCAGTTGGCAGTCAGTGGATTTGGATTCGCAGTCGTCTTTGGATTCGGTGGACCATTTTAGACGGGGGGTGTGGCTGGGGGGCACAGGGGCATGGGACAGGATGAGAGTGGGGAAAGACTGGGCTGAATCTGAATAAAAACAAAAGTAAGCAGCGCCACTCTCTCACCAACTCTCTCACAGACACATCGCACAGAGAGCAACGCACGCTGTGTTGTTCGTGTGTTGCTCATGTGTCACGTTCGTGTTGCTGTTGCATTTGCGTTGGTGTTTGGCACTCTTTCACTCTCTCTCTCTCTCTCTCTTTTCCGCCTCTCCGGGAGAAGGAGGAAAAGAGAGTGGAGCACTCTCGCGAGCAGCATTATTGCCACATTGGCGCTGCTGCTGCTGCTGTTGCTGTCGTTGTCGTTGTCGTTGTGACCTTCACAATTTGCGCAAAGGCAATGTGTCGATGACCACAGATTTTATACAGCAACACGGACTCACACACACACACACTCACGCACTCAGTTGGCAGAGAGCATAAAAGCAGCGAGTGCAACACATTCGCATTGCAATTGATTGTGCTGTACGTACATGGGTATGTGTGCGTGCGCCTTGCTTGCCTGACATTTTGTAGGAAAGAGTACGCTGGATTAGGAGTGCATTATGTATGCAACAGATTATACCAACTTCAAATTGATTGCAATATGCAAAGTTCCCCATTAGGTCATATGATTAATTGGTTGAGATAAAACCGTTCTCAGCTTAGATCGAATCCCCGAAATAATTGTGCGATTTACTT**cctgcagg**

***Sbf*I**
