# Supplementary material for: Redundant and Singular Regulatory Elements Underlie the Rapidly Evolving Pigmentation of Drosophila
Source: Mol Biol Evol. 2025 Sep 4;42(9):msaf213. doi: 10.1093/molbev/msaf213 (PMC12449766; doi:10.1093/molbev/msaf213)
Supplement: msaf213_Supplementary_Data [file msaf213_supplementary_data.zip › Supplementary Document S5 synthesized ortho hth sequences v2.docx]

**Supplement Document S5**

**Synthesized and subcloned sequence from *D. auraria* and orthologous to the *D. melanogaster* *S3.11* *hth* CRE. Sequence is flanked by *Asc*I and *Sbf*I restriction sites that were used to subclone in the same sites of the S3aG reporter transgene vector.**

***Asc*I**

**ggcgcgcc**CTGTGACAGCATAAATATCCGCTTAAAAGTGACCCTCAAACTACAAGAACAGCTGTTCAAAACGCGTTGCCATAAAACATAGCCTTGCCTCTCGCTCTCAGGCAAGCACCGTACAAGCCATCTCGCTCTGGCTTACTGCTCTCCGGCGAATTCGAAACAAAAACAAACCATTAAAAGCGCGGCGAAGAGAGTGTGTCCGTGTGTGTGTGTGTGTGTGTGTTGAGAGAGTGCACTTGTCTGTCAAGATTATGGCGGTTTCAGTCACTTTGTTGTGTTGTTGTTTTTGCTGCTTTTGGGGCAGCGGCGGCGACGGCGGCGAAGAAGCAGAAGAAGAAGAAGCAGAGCCGACGGCAACTCCTATTTTTCTTCATGTGCCATTTTTTCCGTTCTTTCTGTTGTTGTTGTTGCCTTGGTTTTTTGCTTGAGCGGCGTGCGAGCGAGCAGGCGCTAGCGGCGTGAGCGCGAGCGAGCGAGGGAGATGGCGCCACATCAAGTTGATATCGTGTCGTCGTCTTCATCTTTTGCTGTCGCTCGCTCGTCGTTTGCCATTTGATAAATTGCGTTTATAAGAAAGAGATGAATTGTCATGCACACAACGCACACACACACACACACAGACAGGAGGGCTCCCCTCTGCCGACGTCGCTGCCTTGGCGTGCGTGTGTGAGTGTGAGTGTTTAAATCATATTGGTTGCCCCCCAATTGGTGTGAGTGAGAGCCCGACCCGAGCTTTCCCCCTTCGCTCACTCTCCACTCCTTGGTCGCCCTCTCTCGCTCGCTCGTCTGGGAT**cctgcagg**

***Sbf*I**

**Synthesized and subcloned sequence from *D. malerkotliana* and orthologous to the *D. melanogaster* *S3.11* *hth* CRE. Sequence is flanked by *Asc*I and *Sbf*I restriction sites that were used to subclone in the same sites of the S3aG reporter transgene vector.**

***Asc*I**

**ggcgcgcc**TTTCAGATACAAACTACAATCCTACGCCCGCTATAAAATACACTAAAATCGGTGAATAAAGAAATCCAAAATATTAAAATGTAAATACAGTGTTTGAAATTTATGGCAAAACAGCTGTTGCCATAAAACACACTCCACACGCGGCGAATTCGGACAAAAACAAACCATTAAAAGTGCGGCGAAGAGCGCAGAGAGAGCCGGACAACGAGAGAGAGTGCACTTGTCTGTCAAGATTATGGCGGTTTCACTTTGCTTTTATGTTTTTGTTTCTGTTTTCGTTTTGGGGAGCATGGGGGCCAGAAGACGATGTAGAAGAAGAAGAAGAAGCAACTCCTATTTCCTTCATGTGCCATTTTTTTGCTCCAACGTTTTGAGTGAGTGAGTGAGCGCAAGCGAGAGCGAGAGAGGAGTGAGTGAAATGGCGCCACATCAAGTTGATATCGTGTCGTCGTCTTCTTCATCTTTTGATTTGCTGTCCCCCCATTTGATAAATTGCGTTTATAAGAAAGAGATGAATTGTCATGCCACGACACTCGCACACACACACTCGCACTCTGGTGACGTCGACGGCGCTGCCCCAAAAGTGTCAGTGTGTGTGTGAGGGGGAGCGTTAAATCATATTGGCTGCCCCAAAGGTGTCAGTGTGTTTGTGAGCTTTCTCGCAACCCCCCCCTCGGCCCGACCACCCAGACTCTCGTCTCCAAT**cctgcagg**

***Sbf*I**

**Synthesized and subcloned sequence from *D. willistoni* and orthologous to the *D. melanogaster* *S3.11* *hth* CRE. Sequence is flanked by *Asc*I and *Sbf*I restriction sites that were used to subclone in the same sites of the S3aG reporter transgene vector.**

***Asc*I**

**ggcgcgcc**GAATTCGGTACGCGCGCGAAACAAAAACAAACCATTAAGAAGACATGCTCTCTCTCCGGCGTGTGTGTGTGTGTGTGGTGAGAGAGTGCGCTTGTGTGTGTGCGTGTGAGTACGGTGCACTTGTCTGTCAAGATTATGGCCGTTGAGTTTTTGGGGGGCTGGCGGCCAAATAAGAAAAGCAACGCGCCATTGCCCCATCTCAAAACGCCGACGGCAACTCCTATTTTTCTTTTTCTTTTGCATGCATGTGCCATTTGCCATTTTCTTTTGCATAGAAATAGAAATGAGTGTCTTTTTGAGTGAGTCAAATGAGTGAGTGAGTGAGTACGACTTGCTCTCTCGAAGGTCCTTACGCAGCTTCTGCTTCTGCTGCTGCGAGGAGGAAGCAAAAGGCTGGCCATCAACTTGATATCGTGTCGTCGTCTTCATCTTTGCTGTCGCTCGCTCGTCGTTTTCATTTGATAAATTTGCGTTTATAAGAAAGAGATGAATTGTCATGCACAACAGCAGCAGCAGCAGCAGCAAGCAGCAGCGGCAGCGGCAGCCAAAAAGCAGCAACAACTGAAGGGGGGCGTGTGTACATATATATATGTTATGGCTCTCTCTCTTTGCATCTGTATGTGTGTGTGAGCGAACAACGGTTGGCGCTCAGTTGGGGCCGCTTTAAATCATATTGGCTGCCCCTCTGTGTATTGGTGTGCTGCTCGAGTGACTGTATGTGTGTGTGTGAGTGTGGAGCTTTCTCATCGCCACATTTTTCGCTCGCCGACGACCATTCAATGTCGCTCAACGCCAGCTCGCTCGCTCGTCTGGGAT**cctgcagg**

***Sbf*I**

**Synthesized and subcloned sequence from *D. auraria* and orthologous to the *D. melanogaster* *S3.14* *hth* CRE. Sequence is flanked by *Asc*I and *Sbf*I restriction sites that were used to subclone in the same sites of the S3aG reporter transgene vector.**

***Asc*I**

**ggcgcgcc**TGCCAACAAGAACAACAACACGCAGAGCGACAACCGAAAAAAAACACAAAATTGTTCGCACATAAACCTTTGCACTAAAAAATTTTATTGTCGTTTTTATTGTAAAGAAATAAATAAAAAGAGAAAAAGCCAAAGCAAAGCAAAACAAAAACAAAGCAAAACACAAAACAAAAACAAAGGGCAAAAGAGACAGAGAGACAATCAGTCGGCTGTCAGAGAGCGAAATTCGCTCTCCCTCTTGCTCGCGGGCGGTTTTATTTCCCCTTGGCGAGATTATCGTATCGTAAGTTAGCTGCTTAAATATCGTAAGAAGGCAGCGCCATTTGCCATCTTACCTCCTCCTCGCACCGCTTATCGTCGCTGGGAGTGCGAGTGGGACCAAGCTAGTGAAGACACTCAAGTGCAAGGCGAATTTGGTGGGGGCTATGGGGCGAATGGGGCGAATTGAGTTGCCGCCAGTCAATGGGCGCTCGCTCGTATTTATAATATTTTTTTTTTCGTCTTTTCAACCAAGTTAATGATTTTCGATTTTTGCTCAGACTTGACTTATTTGATTTTGGACTCCCTCGTTTTTGTCCCACTATTTTTTGTACCTTTTCTTATGCAATGTTAAATCAATAAAGCGAACAAAGCAAATTCGCTTCGCTTTAACGCTTGAAAAATGCCTTGAATAATACCGAGTATTAATACAAAAATGCTTTAATAAAAATAAAACACACGACTCTTTCGAATTCTTTGTTTATGGGTCTTTTAAATGTCACAATTCTCAATTTTGAGAATGTGCTAAAAC**cctgcagg**

***Sbf*I**

**Synthesized and subcloned sequence from *D. malerkotliana* and orthologous to the *D. melanogaster* *S3.14* *hth* CRE. Sequence is flanked by *Asc*I and *Sbf*I restriction sites that were used to subclone in the same sites of the S3aG reporter transgene vector.**

***Asc*I**

**ggcgcgcc**TGCCAACAGCAACAACAACAACAAACAAGTCTACAAGCAAAAAAAATTGTTCGCACATAAATCTTTGCACTAAAAAATTTTATTGTCGTTTTTATTGTAAAGAAATAAAATAAAAAAGCCAAAGCCAAAGCAAAACAAAACAAACACAAAAGCCCGGAGACAGAGCCAAACAGTCTGCGCTGAACTCGCTCTCCCAGAGTGAGACACGGGCAGAGTCTTTCTCTTTTGCGTACATTTCCCCTGTCAGATTACCGTATCGTAAGTTAGCTGCTTAAATATCGTATCGCATCTTCCATCCTGCTCCCGCTTTAATGGTCTAAGCGAGAGCAAGAGGGATAAAGCTAGTGAAGAGCTGCGGCGAATTGAGTTGCCATTCAATGGGCGAATTATTTTTAATTTTTATTTTGTTTGTGCCCCAAAAGTTAATGATTTTCGTTCAGACTTGACTTTGATTTTTGAGTCACGTCTTTTTCTCTACAAAAAAAAATAAAAATAACATTGACAAATATTTAAAATAAAATTGTGTATATTTTTTTGGTTTTTTTAAGATTCCTTGTTTATGGCGCTTTTAAATGTCACAATTCCCATTCCCACTCGCAATGTGCTAAAAC**cctgcagg**

***Sbf*I**

**Synthesized and subcloned sequence from *D. willistoni* and orthologous to the *D. melanogaster* *S3.14* *hth* CRE. Sequence is flanked by *Asc*I and *Sbf*I restriction sites that were used to subclone in the same sites of the S3aG reporter transgene vector.**

***Asc*I**

**ggcgcgcc**TGGTAAAGTGCGGGGCAAAAGGGGGAAAGACAACGGGCAACTAAACCAAATTGTTCGCACATAAATCTTTGCACTAAAAATTTTATTGTCGTTTTTATTGTAAAGAAATAGAATAACAACAATAACAGCAACAGCAACAGCAACAACAACTGAAAAGGCAGCAGCCAAACAGTCTGCGCTGCCAAAGAGAGTGAAGCAGCAGCGCCACAAGAACACTCACTCAGTCTCCCACTCTCTCACACACACACACGCACGCACATAA

TACTCATTCACTCATTTTCTCATTTATGTTGTGTATTTCCCCTCCACAATACATTTTGGGCTTATCGTATCGTAAGTCTTCGCTTGCTTAATATCGTAAGAAGGCAGCGCCATTGCTGGCCAAGCATGAGCTTCTCTCTGCGTGTGTGTGTCGATGAGTGCGAGCGAGATGAAGCTACTCTAGTGAGTGAGGGCAAAGAGAAATGTACCAACACAACTGCAAATCAAGCGAATTGAAGGCGAATTCAAAGCGAATTCATTTGCCGCCTGC

GAGTCAATAATAATAATAATAATAATATGTAATAAGCAGCTATTTTTCTTTCTCTCTAGTTAATGATTTTTGTTAGTCGATTTAACCAAGTTATGCAATGTTAAATCAATAAAATTGCCTAAGACAACTTTAATAAGAACAAAAGTATTCCAATTTTCCCGTGTTTTTCCCGCATGTTTTAAAGCATAAAGAGAAAAATTGTGCTTTGTCT**cctgcagg**

***Sbf*I**
