# Supplementary material for: Redundant and Singular Regulatory Elements Underlie the Rapidly Evolving Pigmentation of Drosophila
Source: Mol Biol Evol. 2025 Sep 4;42(9):msaf213. doi: 10.1093/molbev/msaf213 (PMC12449766; doi:10.1093/molbev/msaf213)
Supplement: msaf213_Supplementary_Data [file msaf213_supplementary_data.zip › Supplementary Document S6 ortho Eip74EF sequences revised v1.docx]

**Supplement Document S6**

**PCR-amplified and subcloned sequence from *D. willistoni* and orthologous to the *D. melanogaster* *S3.20 Eip74EF* CRE. Sequence is flanked by *Asc*I and *Sbf*I restriction sites that were used to subclone in the same sites of the S3aG reporter transgene vector.**

***Asc*I**

**ggcgcgcc**AGCCTTAGTTGAGGTCTTGCAATGCACCTCGAAATGCATAAAAACAACAATAACAACAACAAAATGGCAAAATGCATTACAAATAAAAATAAAACTAAAGCACAAAAAAAAAATCAACTCCCGCGCTTTTCAGAGAGAGAGTGAGTCCGAGAGCAACGGGAGAGAATATGGATAAGCATTCAAGAAAAGAGAGAGAGAGAAAGAGAGGGGCTAAATTTAGTTCAATGAATTTGCACATTTTTAGCGGTTCTCTTAAAATTTCACTCTCTCGCTCTTTCTCTTAAGCTGTCAGACTTTGTTTGTGTTTTGTTGTTATTGTTGTGCCGAGGAGTAGATGTTGTGGGGGTGGCGGAACTGGTGCGGGGGGCTTGGGGAGTGTGGTCGCGGTGGAAGGCGGCCTAGTGCATTGACTACTAGCCAGACGCAACACACTGCTAGCAGCGACAGCGACAGAAGCAGAAGTACACGCCAGAGCACGTTACTCGACTTGCTGCTGCTTGCTTCGATGGTGATAATGATGTTGCGGACTCTGCAACAACTGCTGCAATGCTGCCGATGATGATGACGTTGTGTTGTGGATGATGATGATGATGATGATGATGATGACGATGGTTATCGTACGAAGGTCTTTCACCCGAACTCGGTCATTGGCGTACATTTTGTGCTGCTGCTGCGAGTTTGCTGCTGTCGCTGCGCTGCCTTTGCTGGTGTTGTTTCTGGTGTTGTTGATGTTGCTGCTGCTGCTGTTGGCTCTGCTCTGCTCGCAAATCCAGAGCTTTTCAAAGTGTACATGTCGCAATTTCTTAAGAAAGTTCAATTACCCCAAACAATCAGATTTACTGTCTGTTTTTTGATGCATACACTCGACGTCGATGCCGGCGCAGCACACTGGGCCTCAACCCAAAAAGACAAATAACACATCTAACATTTTTTGTGTTAATTCTTTATTAATTCCACTGGAAAGATAACTGGTACCT**cctgcagg**

***Sbf*I**

**PCR-amplified and subcloned sequence from *D. willistoni* and orthologous to the *D. melanogaster* *S3.21 Eip74EF* CRE. Sequence is flanked by *Asc*I and *Sbf*I restriction sites that were used to subclone in the same sites of the S3aG reporter transgene vector.**

***Asc*I**

**ggcgcgcc**TCGATGGCATTTTCTTTGATCAGTGAAAAGTGAAATTACTCCACCCCCCGCCCAAAAAAAAGAAGAAAAAAACAAGAAAAGCTTAAAAATAAAAAAAAAATACGTTACTCAATGAAATAAATTTTTGGTGTGTGTGTGTGTTTTTTTTTCATTTTATTATTTTGTTTGACTCAATTTTCCGCTGCTGACTGCATTTGAATTGCATTTTGTTGTGTTTCTATACGTTTTTTTGTGTGTGTTGTTGCTTTTTGTTTTTTTTTTGGGTGCATTAAACGGCAAATTGTTTCGTGGGATCAACACAAAGTGCGTTGATCTCGGCGCAGCTTACAAACACAAAAAAAAAAAACAAAAGTGAATGAATATGAATGCGCGAATGCGAATGGGGCGAAACACAGCGGCGAACGTTGCGTCAACGTCAGCGTGGCGTAGGCGTCGCGAGCGCTGCCTTGTTACGCATGGGCTTTTTTTTTTTCTTTACATGAAACAAAACAAAACAACGGCAATAAAAGCAACAATAACAACAAAATAGCTTAAATGTGACTAACAATTAATAAATTATTAATTGCTCTCGAAAGGCAAAAGCCAGCACCCGCACCCGCACCCGTGTCCCATAAGTGTTAAATGAATCATAATACACATTTCGAGGCATTCCAAGGCAAGCTGCGGCAGCCGCTTCTCATCTTTCTTCTTCTTTATCGTCATCGTGGTTGGGTTTCTCTCGGTTGGAAGGGATATCATCGTATGAGACAGTCGTCAATTGAGTTCCGGATTTCTTTTTCAAGTCTTAGAATTTCTATAGCATAGTTTTAGGTCTAATTAGAGAAGAAAGTGCTTACAACTTGCTACAAGTTAATACTAGAGAATGGAAAAAGGCAAGTTGCTTCTGTTTGTTTTTTGTTTTTATGATCAATCGGAGAGAAATCGTAATGAAGATTTGACAAAGTCGATCATACTGAATAAAATTCTAAAGGATATTTTCTAGAGTTTAGAAGCAAAGTTGAATTCTATTTTCATTTCATTTAAATTGGAGACTTTTAACAAGAATAATTTAAAGAGTAATAGTCGTAGAGACTTGGCATAAGAATGTATGAGACAAAAAACACTTTGTAGGTTGTACAATTTTATTTTCAATACTTTGGAGACTTTTTGAAAAAATCATTCGTGTAATAATTTGCTTTAATTGAATTAAAGTTATTCAAAAGTTTAGAAAACAATGAAGAGTTTGCTCATTATTTTTAGTAACTTGGAGACTTTTTCAAATAGGCGTTGCCAGTTACTAAATTAATAAAATAAAAGTTAACCTTTTATAATATAAGTTATTACATATTACAATAACTAATTGTAGAGTTTGGTAAATAAGTTAAATAATATTTGAAGACTTTCGATTGAAAAATAACTAGAATTTTGTTCTCATATAATATAACAGAGTTTTATTATTTCATTTTATTCTAATTAAGAGTTATTCGTATTTCACTTGATAATTTTGTGTAAAGTTTTTAAGAATTAAGTTAAAGAATAACTTTATTGTAGAGTTTTTGAAAATTATACTTTAAAAAAGTAAAAAGAGAATTGCCATAAGGTTATGGGGGGAGTAACCTTTGGGTATAAGCAGTCTCTCTCTCTCTTTCTCTCCCCCAAATTGCGGTAGAAATTTTGGATACTCAACATTTTTCTCCTTTCTTTTTAATCATTTTTGTTTTTTTTGTTTTTGGGGAATTTGCCTTATATTTTCCCATCTGATCGTATCGTATCTGATCGGTTTATGGATCGGAACAGCAGCAGGCCAATTGTGATGATCTCCCTGCAAACGCTCACTCTCCACTTACAACACACTCTCTCCCGCTCTTTCTCTCTCTCCATTCGCTCATTTCACTTCACTCGACGCGAAAGCAAAAGCAAAACACAAATTTTTTTTTTATTTCGCCAGCCCGTTGGATATTTTTTGCGCTGCTGCTGCTCTGCTTGCTGCGCTGCGCTCGTCGTTTGTTGTAAGTGTA**cctgcagg**

***Sbf*I**

**PCR-amplified and subcloned sequence from *D. willistoni* and orthologous to the *D. melanogaster* *S3.22 Eip74EF* CRE. Sequence is flanked by *Asc*I and *Sbf*I restriction sites that were used to subclone in the same sites of the S3aG reporter transgene vector.**

***Asc*I**

**ggcgcgcc**TCCATCGGTGGGCAATGACGACGACGTGTACCCAATATTTTGCATAAACGTTGATCTTCCGACAGAGAGTGAGAGAGAGAGAGAGAGAGGGAAGTGAGTGGCGGGGGGGGGCCAGAAATATCTAATTATAATTTTACCACACAACTGTGGCGCCAAGCAAAAAATAAAACCAAAAACAACAACTAGAAAATGGTGCTGAAGGTTTTTCCTCTGCAGCATTTTCTTTTCAAGTTGAACTTGCTTTTGCTTGTTGTTGACCAGGCAGAAAAGTGAAATAGACGCTGCTGCTGCTTGTATTGAATAATGAAAAACAAGTTTTCCGCGGTGCATTGCGCATCAACATGGAAAAACCAAAGCGCAAATGCAACAGGCCAAACTAAACCGATTGAGAGATGATGATGATGGCAATGATGATGATGATGATGACGTGCCCCGTACAGTGGCTTAGAATTTGCCATAGACTACAAAAAAAATATTTATCAATAGTGAAGGGTGACTAACTAAATTCATTCCAGCTAAAGCTGACCCATTATTAAATTAAAGCAAGAGCAAAAGATTGGAAACCTTCTTAATATCGAGTTTAAGCAATTTTATAATTGATCAGTTGGAGTTTCTGTTTTTACTTTAATACAGGTTCCCCTAGAAATTTTGAATGAGTGGCTTAGTATTAGGTTATATATAAAAAGATATAGGAATATTGATAATCAACTCGCCGACTAGAAAGATACCGAAACATTGAATCTGCCGCATCAAACGAGATGACACTTAGCACCCTCTACTCTCTTCTACCCTAAGCTAGGTGGTAACCTACTGACTGACCCTCCCATCTAAATGTTATGAAGGAAATACCGTTGAGAACAAGCTCATTTTTATCAGATTGTGATACAAAAAATCTCAATCGATGAAGAATATGATTCATTCGTCTTTTCTAGTTTAAAGCAAAAATACTTTTTCATAAATCAATCAAAAGCGAAAGCCCCAAATATTATATACTGCCTAAATTGGAGCTAAATTGAAGATTTGTGGTATACAGTTTCTTTTAGTTTGACTAGATCTACAACCTCTTTAAAGAGTTGAATCTGTATTTTTTAAGAATTTAGAACAACTAAAACATAGATTTTAATGATTTTAGAGTTGTAATGATTATCATACTATTTCTTCAACAATCGTTACTGTTTATCGTCTTAAAACAATCAATATCCAAACAATTATTGCAATATTTGATTAAGCTTAATTGCTATAACTTTTTTCTCTAAACTCTTGTCTTCAATATCTTTCTTTGACGTCAAGCAAAATATCTTTCAAACACAGAAAAATGTTATTTAAAATTATTTTTAGAATTTATTTATTTTTTATTTTACTAATAGAAGTTAGCAGGCTAGGTTAATTAGTTTACTGAGGGCCAAATTAGAGCTACTTTTTCAGTTTAATAA

GCGTTTTGAAACCACTGTGCAGTGGCAACGCGCGGGTGCCAGGCAGCAGCGGCAAAATGTTGTAATGTCGCAGACTCTCTCCCCTTCTCTCTTTTTCTCTCTCTCTCTCTCTCTCTCCATGTGTGTGTGTGTGTGTGTGTGTTTATCTCTGTCTCTAACTCTCTAGTTTTGAGTTTTGCCTGTGCAGTTTTTGCAGCCTGCAGGAAGCGATCGCAACCGCGGAAGCTAGCGGATC**cctgcagg**

***Sbf*I**

**PCR-amplified and subcloned sequence from *D. willistoni* and orthologous to the *D. melanogaster* *S3.23 Eip74EF* CRE. Sequence is flanked by *Asc*I and *Sbf*I restriction sites that were used to subclone in the same sites of the S3aG reporter transgene vector.**

***Asc*I**

**ggcgcgcc**TTGAGTTTTGCCTGTGCAGTTTTTGCAGTTTGCTTTCAAGTTTGCAATTTTTTTTTTCGTATTCCGCCTCGTTTCGTTTCGTAGTTCAATGTTAACGAACTTTTTCTGCAAGTTGTTGTTGTTGCTGTTGCATTTGTTGTAGTTGTTGTTTTTAGACTTTCACTTATAATGAATTTGGCGAGTGGAAAACTTGTTGCTCTTGTTGCAGCAGCAGCACCAGTTGTTGTTGTTGTTGTTGTTGCTGGTATTGCTTTGCATGCAGTTCAATAACATGTTATGTAAAAAGCATTTTTCCAAGTTTCTTTTGGTTTTTCCTTTGTTCAAATCATTCGTTCGTCTTTTCTTTTGCTGCTGCTGCTGCTGCAGCTGTTGCTTTTCTTTTCTTTTAATTTTTTTTTTGCAGTTTTTCCTCCCCACAAAAATTTTCTTACTGCAGGGCTCCATTTATTTTTGTTGTTGTTGTATTTTATACCATATTGTTTATACATATATATATATATATATATACGTTTTATGTTGTAATTCGTAATTTCTAATGTTATTGAACTTTAAAAAACAACAACAACAACACAATAAAAAAGAAGACAAAAAGAAACACCAACAACAACAACAACCAACTTTGATTCAACTGAAAATTAGTTATAACCGGAAGCCACAAAACAAAAATTGAAACAATGCAACAAGACGACTTTCTACATTTATTTATCTTCTTTTTTTCGTTGTTTTTGTTGTTGTTGGCTTTTTTGGTTGGCTATATTAATTGCCCGATCATCATCATCATCATGGTGATGTGAGGGATAATGATGGTG**cctgcagg**

***Sbf*I**

**PCR-amplified and subcloned sequence from *D. willistoni* and orthologous to the *D. melanogaster* *S3.24 Eip74EF* CRE. Sequence is flanked by *Asc*I and *Sbf*I restriction sites that were used to subclone in the same sites of the S3aG reporter transgene vector.**

***Asc*I**

**ggcgcgcc**GCATCAAAATTACGGAACCTCTTCCTAGCCCCACTTCCTCTTTTCGACCTGTGCCTCCCACCAACTTCCCTTATTAGTTTTGGCCCTGTTCATTATTTTGTTTTTGTCGATCTTAGATCTTTTTTTGTTTGTTCCGGCCCTGTCGGAAAGTTCAGTTTGCTCAGTTCCCCAAGTTCCAGTTTATGCGATGTTCGTTTAGCTTTTGATTGCATTAGAGCGTGTGCCGCCGCTGCCGTCGCCACCGACCAACTGACTACACGACGTCGACGTCGCCGTCGTCGCATCTCGTTTAAAATAAGCAGCAGCAGCAGCGGCAGCAGCAAAATGAAAACAAAAGGCAAAGCAACAGAGCGACAGCAGCGCTGTTGACGACGTTGACGATGATACAACCTTACAAAATTCTCAGTCACTGTCTGCCTCCCCTTCACCTCCCTCTCCGCGCTCCATTGTCTCTCTCCTTCTCTGGCTCCTACTCCTCTTGCTCCTCCTCCTCCACTCTTCATTTCACATAAACCCAATAGCAGCAATACCCGACTTTGGGTGGCTTTGAATGTTTAGCGCTGCCTGAACTTGTCTTGCACACACACACACACATGCATATACACTGACGGGCAGGCAAATGGTAGTGAGAGCGAGCGGCGACGTCAGCAGAGCTCCACACACACACACACTCTGAGTGGCCGGAGAGCAACAAACACATTGATATAGCTTTATTTTTGCCATAGCGTGTGGTATGCAGCCTTGAATTAGTTCGCTGCACCCGCCTGTTATATACATACATACAAACAGACAAACACACAAACATAGACAGCACACACACACACACACACACAGACACACAAGCAAATGTATAAGCAAAGCGTGCGC**cctgcagg**

***Sbf*I**

**PCR-amplified and subcloned sequence from *D. willistoni* and orthologous to the *D. melanogaster* *S3.25 Eip74EF* CRE. Sequence is flanked by *Asc*I and *Sbf*I restriction sites that were used to subclone in the same sites of the S3aG reporter transgene vector.**

***Asc*I**

**Ggcgcgcc**GCGCTTTAGATGGCAGTTGTCGGTACATTGGACTCATTTCACACACACACACACACACACAGTAAAACTATGTCTGTGTTGCTGTTGTCGTCGTCGCCTCATTTAGGTTCTTCTTTAACTAAGCAACCCACCCCCTTATCCCCTCCCTTCCGTTCGACCCTCTCAAAAACTCGCCTCCTTGTTCTAAAAACAAAACTCTTCCTTCTGATGTGCCCTGCTACTGCTGCTGCTGCTGCTTCTCAAAGTCGTCGTCGCTGTCGCTGTCGTTGTTGCTCTGCTTCCTTTATTTGCAACAACTTGCTCTCTCGCTTCCACACTTGCAATCGCAAATCGTCAAAGTAAATGGAAAGGCCCCCAACAGCAGCGGCAGCAGCAGCAGCAACAACAACATCAGCAGCAATCAAACAAACGGAAAGTGAAAATTTGCTCTACTGCCAGAGAGAGAGCTAAATAAAAAGTGTGATAGAAAGAGAGAGAGAGCGCGCGCTCTCACATATACGCACACACTTGCATTGAGTCATAATCATGTCGATTGCAGCAGCAGTAGCAGCTACAGCAGCAGCGACGCTAGCGACAGCGTCAGCATTGCCTTCTCTTGCCAAACATGTTCCTGCTTGCTCTCTCTCCCGTTCGCTCTCGCTCTCCTCCTGTCCACCCCCCATGATCGTGTTTAGCCCTTTTTCATATAGCCTGACCCATTT**cctgcagg**

***Sbf*I**

**PCR-amplified and subcloned sequence from *D. willistoni* and orthologous to the *D. melanogaster* *S3.26 Eip74EF* CRE. Sequence is flanked by *Asc*I and *Sbf*I restriction sites that were used to subclone in the same sites of the S3aG reporter transgene vector.**

***Asc*I**

**Ggcgcgcc**GCCCCATAAAAAAGCGTCTTAATCGTTTCATACCAAGGCGCGATATGTTTGCCCCACTCAACAAGAATTGCATGCAAATGCGCATGTGGCACACAAAAGTTCCCCCAAACACAAACACACACTCACACACACACACATACAAATACATACAATTAAGCAGCTGCAACAAGAAGAAGCAACATCAGCAGCAGAGGCACCAGGCGGTAGCAGCATTGTTAAATAGACAGAGAGAGAGAGCAAAGAATATATATATGTGTGTGTCTGTGTGTGTGTGGGAGAGACAGCGAGAGCGCCAAACATTTTTGCTCGCTCGCCAAACTGTTACGTTTTGTTTGCCATTTCGTCGTCGTCGTCGCCGTCGCCGTCGTCGTCGCGAGTGGAGCTCCGTCGTTTGGCTGGGTGGTTTGCTTGACTCTCCGGCAAAGTTTTCGCAATCGCAGCGACGCCAACATCGCAGCCAACTGCGACTGCGGGCAGAGAGAGCAATTGGCCAAACGAACGAACGAACAAACGGGCGTTCAGTCAGCGCGAGTCAAGCAAAGCAAGCGAGCCCCATTCAAAATTTTTGCCGTTGCTGCTTGCTCACTTGGCGTTTTATATTATTATTATCATTTTTTTTTTGGTTATTTATTTCTGATTTGTTTTTTTTTTGTTTAATTTTGTTGTTATTTTGGTTGGTTGGCTATTTTTCTTGATATCTCGTTGTCAACATCACCGCCGCCAACAACAACAACAACAACACCATCGAAGTCATCGTCAAAGTTGTTGCATTTGTATTGTGCATGTGTGCGTGCTGCTGCATGCGTGCGTGAGTGAGTGAGTGCGGCGTGCGTGTGTGTGCTGCTGTTTGCCTTTTACACCTGCGTGTGTGTTGGTGAGTGGGGCGCGCGCGCGCTTTTGCGAGAAACATCGTCACTGTTTGCTGTCAACGACGCTGCCTACTCTTGACGTCGACGTTGACGTCGCGACGTCGTTGCCGTTGCCGTCGTCGCTGCAACATTTTTGTGTTGTTTGCGCCTAACAAACACTCGCAGCGGTTTTTGTGCTGACTTTAGATTTTAGTTTTGCTGATACCGTAAGAGATAAATGACGTGCCGCGGCGGCACCACTTCACATCAAAAAAAAGAAAGAAAATAAAAAAATAAAACCATCAACAACACAGAGCAAAAAATTAATATAGAAACAAAATATAAAAAAAACAAATATTGCCAAAAACTAAATTCAACAAAAAGTTCTAACAAGAAACAGGAGAAAGAAACTTACTCAGCACATCAAAACGTCAAATAAACAAATTTTTTTTTGTCATCGTCTCAATTGCAACAACAACATCAATGCCTCA**cctgcagg**

***Sbf*I**

**PCR-amplified and subcloned sequence from *D. willistoni* and orthologous to the *D. melanogaster* *S3.27 Eip74EF* CRE. Sequence is flanked by *Asc*I and *Sbf*I restriction sites that were used to subclone in the same sites of the S3aG reporter transgene vector.**

***Asc*I**

**ggcgcgcc**GCCAAAAGGAAGGAAGGAAGGTAGGGAGGATGGTGAATACGGGGTGGCTTTCGGGAGGGGATGTCTAAAGAAAGCAAAATTCAGCTTAATTACGAATATCAAAAAGAGAGAGATAGAGTGAGAGAGAGAGAGAGTGAGAAAGAGGGGGAGCAGCAAACAAAGACCGATTAAACGAAAAGCTAAAGCACCAAAAAGAACCAACAAGAAATGCTTCCAAAAATTACACCAATCCACAAATACCCTAACACATGAATGGCAAATCTTCATCCGTATCTCTACTTCCCCTTCTCCCCTCCCGTCTCTTCGAAGACTTCTCAAAATAGATTTGATTCTAATCTTGACTTTCTTCTTCCTCTTGGCAATCGAAACCCTTTTGTTTCTTCACAAGTGAAGCGAGCGGTGGTGGCGGTGGTGGTGGGTTGTAGGTGGAGGGGGCAAAGACTTGTCAAAAAGAGCATCGCCAACAAAAAAAAAACAAAACATAGAAGAAGAAAGGCAGCGAAAAAACGGCGACAACAACGCAAAAGTTCTATATTTAAATATTATAATTTATGTGTTTGTTTGCCTATTGTATGTATGTGCGTTTCTTTCTCTCTCTCTGTGTGTGTGTGTGTGTGTGTGATGGCTGGGATGTGGTTGGTAGATGTCTCTTCCCCATTCCCATTCGATGATCCCCCCACAACTCACACACACACATACACACACGCATACCATTCCCAGTTTTATTCCCAGTTTGTAACGTCAGCAGCGCGGCATATTTTGACACAGTGTCTTCCCCTGTGTCCCCGTCACCACAGTCACCCACTCCTTCTTCCTCGCTGTTTTATTTTTTTGTGCTGTTCTGTTACCGCACGTCCCTTTACCATCCCTCTCACACACACTCGCTCACTTACTCACTCTCTCTTTCTGGCTTGCTTTGTCACTCTTATAGTCTTCCGCTCTCTCTCTTTCTCCCTATCTCTGTCTAAATTCCTGTTCTGCCTCTGCTGACTTATTTTGGCTTTCATGCCGACACATTTACTCTTGTCTGTATCCGCAACTGTCTCTCTATGTCTGTGTGTGTGTGTGTGTGTATGGGTGAGGAGAGTGTCTGTCTCTGTATGGGTAGAAAGAATAAAAACTGACTTGCTGCTTGTCGTTTTGGGTGTGTCTGTGTGTGTGTTTGTGTGAGTTGTGCGCTTTTTGGAAATTCAGTCTCAAGTTCAAGGTCTCGCGGATAACGCACACACATACAAACAAGCGGACACACGGACACAGACATATGGCAAGTGAAAGAAGAAGCAGCAGCAGCAGCACACATACCAGCACGATTCTTATTGTTTATTAAAGGCAGACACACACACACACACACACATACGAATAAACAAACATTTCTACATGCATGTGGCAAAAGCAATTTCTCATTGTTGTTAATACATACTAATACAAACGTATATATCTATCTATATACTTTATATGCCTACTATATAATACTTATACGGAGCTACAAAAAAAAAAAAATATATCTTCTTTAATATTTCCGCATTAAGAACAAAAATTTACGACCAACAAATTTGTTGTTGATTTTTTTTGGTTGGTTTTTGTTTTTTGTTGCTATTTGTTGCTTTCTATACTAAATACTTTAAAAAATGAAAAAAACAGCAACAACAACAAAAAAACAAAGTATAAATAAATAATATAAAGGAAATATAATGTAATTTTTGTTTGGTGTTGTTGCTAATTTTGTTTTAATTTGTCATTAAGTACTTTTATATATATATATATACATATATATATATATATATATATGTATGTATGTGTGAGTGTGTTTTATTGTATGTGGTATAATATGCATATAGATTGACGCATTTGCCTGAAACGCAATTTGTATAATATTTAAATTGACCAACAAAATTTTGTAATAATTTAAATAAAGGCAAAAACTCTCTAGAGTGCATATATACCATACATATGCCCCTAACATACATATATATATGTATGTACATATATACATACAATACAATCCATTTGTATATATATCGTGTCTTTATTTATTCAAGAATTTCGTTTCGATTTTTGCAGGTGGCAGT**cctgcagg**

***Sbf*I**

**PCR-amplified and subcloned sequence from *D. willistoni* and orthologous to the *D. melanogaster* *S3.28 Eip74EF* CRE. Sequence is flanked by *Asc*I and *Sbf*I restriction sites that were used to subclone in the same sites of the S3aG reporter transgene vector.**

***Asc*I**

**ggcgcgcc**CCAATTTGGTGAGCCATGAACTCAAAGCAACTCAACGGCAACGGCAACAACTGCAACAGCAACAGCAGCAGCAACAACAACCGTTAAAAGCAATGACAAAAGAAATGCAAAACGAGAAGAAAAGGGAAAACCAACAAACGAAAAAAGAAAGAAAAGAAAAGAAAAATGGCATCTCACTTTCTTTTGCAATGCGAAAAGAAAAATGCACAAGTTGTTGTTGTTGCCTGATAGCAATTGCTGCTGCTGCTGCTGCTGTGAAAAGTAAAAGTGCATTTTCACTTATTTCATGCAGCCAGCAGCAGCAGCCACAACGGCAGCGGCATAAAAATGAAACAAAAATAAAACTAAAGAGGAACAACAAAAAATAAAATGAGAAAAAAAAAATTGCATTGAATGTCAAGCTGTTCACATACACACACACACATCCACACAAATAGCACACACAGTGGGCAAGGCAAGCATGTTGTCTCGCTCTGGCAAATGCAATATGCAGAGGAAAAGCCAAGCATCAGCAGCAGCAGCAAACAAGCAATGAAGCTTGCAAGCACACTCACTCACACACACACACACTTGTCGTCGTCGGCACTCACATGCACACAATTGGCAGCAGCAGAAACAGCGACAGCGGCGGCAGTAAGAGAGGAAGAGAAATCAGGTCAAGGCTTGTCGGCGACGAGTTTACAATTTATGCTTTGCTGGCTGCATTATTGCATGTTGTACGTTTGTCTCTCTATGCGTGTTTGTGTGTGTGAGTGAGTGTGAATGTGTGTGCTTTTTACAGCTTTTTAAATACACAAGCTTACAGACAAACAAACAAACAAGCAAGCAGCAAACCGTCGACGTCGTCTCATTTTGATTGCCT**cctgcagg**

***Sbf*I**

**PCR-amplified and subcloned sequence from *D. willistoni* and orthologous to the *D. melanogaster* *S3.29 Eip74EF* CRE. Sequence is flanked by *Asc*I and *Sbf*I restriction sites that were used to subclone in the same sites of the S3aG reporter transgene vector.**

***Asc*I**

**ggcgcgcc**TTATGCTTCACCCCCTAAGTTATGCTACACATATTCAAATTCTTACAGTTTCATTCACTCGTCATGACTAGTTTACACTAATAAAATAAAAGGCACAGGTATAATTTCGTTTGCTATTTAGTAAGTTTTTGGTTTTGCTTCTTTTTTGTTTTATTTTACATCCGGCGTCAGCGTAGCGGTTGTCATCATCATCATCATCATCATCATCAACATCATCATCAGAGCAACAACAACAACATTGTTTTTATTGTTGTTGTCGTTGCCCAATTCAACAGTCAGTTGGTGGTTTTTTTTTTTGGTGGACCAACAGCAGCAGCAGCAGACCCAAGGGTGCTCAGAGAGGAGGGCAATGAAGATGACGACGGGTGTATAGGCCAAGCAAGAGAGAGAGGGCTGGAGGAGCGGCAGCGGGAGTTGGCGTTGAATTAAAAACAAAAGTGCTTCTCACTAATACAATGACAATTTGGCTGAGAGCAACGCACGCTTTCGTTAGCTGCTGCTGCTGTTCGTTGTCTCATTGTCGTTGCATTTGCGTTGGTGTCGTCGTCGTCGCTGCTGCTCTCTCCGTCCTTGCTCTCTCTTGCGCGAGCTCTCTCGTTTACACTCTCGCCGTTTACTCTCTCTCTCTCTCTCTGAGCAGAGGCCCCCATCATCATTGCCACATTGGCGC

TGCCTTCTCCTGCTGCTGCTGCCGCTGCTGTTAACGTCAACTTCGTTGTCGTTGCTGTTGCTGTTGCTGTCGTTGTCGTTGTGACCTTCACAATTTGCGCAAAGGCAATGTGTCGATGACCACAGATTTTAATACACAGCAACTCACACACACACACTTGCGTTGAGAGCGAAACAAACACACAGACAAAGAGCATAAAAGCAGCGAGTGCAACACATTCGCATTGCAATTGATTGCGTTGTATGTATGTATTCAATTTATATATGTGTTTGTGTGCTGTTTATTCGCCCCCTGACTGACATTTTTAAATGTATTCAAATACCCATTAGAAGGGTATTTTTCATTAAAGATTTAATTAATCAACTTAACTGTATTGAGAGTGTTTATAGGGTTAATCAAAGAGATATTTTTGATCTTTTATTTTCATAAGCTTCTAATTTCTAATATATGCATCTAGAATCTAGACTTTGATGTTTGAAATTTTACTAATATTTATATTTAATAATTGACCATAGGATATCATTTAGTCGTTGCCCCACTTGTTTTTTTT

TTTTGTTCTCTCATCTGATTATCCTCTTTTCGCTTTTGCAAACCGCACAAAATGAACTGTCTG**cctgcagg**

***Sbf*I**
