# Supplementary material for: Redundant and Singular Regulatory Elements Underlie the Rapidly Evolving Pigmentation of Drosophila
Source: Mol Biol Evol. 2025 Sep 4;42(9):msaf213. doi: 10.1093/molbev/msaf213 (PMC12449766; doi:10.1093/molbev/msaf213)
Supplement: msaf213_Supplementary_Data [file msaf213_supplementary_data.zip › Supplementary Document S7 deleted hth sequences v1.docx]

**Supplement Document S7**

**The sequence deleted from the *homothorax* locus in the *hthΔS3.11* allele.**

CCAATGACTCTCAAATCATGTTACATGCCTAGTCTTGCAATGGCTGCACAACTGTGAAACAGCTGTAAAACGCGTTGCCATAAAACACTTAGGGCCTCTCGCTCCAACGCAACTGCAACGCATGCCATCTCGCTCGGCGCTCTCTCCCGCGCTGTTCGGCGAATTCAGACAAAAACAAACCATTAAAAGCGCGGCGAAAAGAGCGCGTGCACTTGTCTGTCAAGATTATGGCGGTTTCAGTCACTTTGTTGCTGTTTCTGTGTGTTCGATGTTTTTTTTTCGTATTTCGCAGCGACGGCGAAGAAGAAGAAGAGCGCCGACGGCAACTCCTATTTTTCTTCATGTGCCATTTTTTTGTTTTTGGCTTTCTGTTTCGGTTCTTGCTTGAGTGAGTGAGCGGGAGCAAGCGAGATGGCGCCACATCAAGTTGATATCGTGTCGTCGTCTTCATCTTTTGCTGTCGCTCGTCGTTTGCGTTTGATAAATTGCGTTTATAAGAAAGAGATGAATTGTCATGCACACAACACACACTTCTCTGCCGGCGTCGACGGCGACGCCGACTGCACTGCCGGCGTTAACATCACTGTAGGTGTTCGCAAGTGTGTGCCTGTGTGTGTGCGTGCGTGAGTGTTTAAATCATATTGGCTGGCCCCCCAATTGGTGTGAGTGCACGTGTATGGGTAAGCTTTCCCTCTCTGCCCTCTCTTTCGCTCGTCTGGGATTGGAAGTTTCCAAATGCAATTTTCCTTGAGGTTTTCCCGAGTCGGTTCCTTTTCGGCTTTCTTCTTTCATTTTTTTTTCGCTCACTGGGTGGAGGCCCAGCCCCCTAAATTCGCTTCCTGCCCCTCCTTTTGTACACCCCCCCCCCCACCGTTCGCTCTTCTCGCTTCTTGATTATTTTCCTTTTTCGGTGATTTAAATTTCATGGTCCCTTCAATTTGGCAAAGTTCTAAATATTAAATTAAATTCCTGCTTCATTTGCAAAAATTCTTGAGCTTTTCTGGAGCTGTCCGCTTTATTTGCATTCTTCGAAATTTAAGTTATCTGTTCTTATTAAATTTCATCTGAAAACATTTTTTCACTCAGTATTATTCTACGAACTAAGTATATTCTATTCCTCAATTAGCGCTTCTCAGAAAACCTCGAGTTTTCTTAAACAAATAAACTTCATTTGTCTAACATTTCGTTGGGATCCTTGAACTTTGCTACCGCGTCGATCGATCTATTCGAATTTAACTTGGTCACCGATAAGTCTGAAAAGTTCCCACAC

**The sequence deleted from the *homothorax* locus in the *hthΔS3.14* allele.**

CTGAAAAGCAAATGGGGTCGCCACCGAAATAGTGCCACTAAAAATAGAAAATCGTTCGATGCGTTTCACCGACAAAAGGCCGAAAAGCAAATCAAGCAAAGCAACAAACGAAATTAAAAATAAATATATAAAAACGGGAATATAAAAAAAGTCGCGTCTAACTTAAGGCAATTTAAAATTCAACTAAAAGAAAGCGATATATTCAAAGGTCAAAAAAGAACAAACCAAAAATAGGTTACTAAAAATACAACAAAATTCGCACAAGAGCAAACATAATTAACAAAACCAAAATTAATTTCAAAAACATTTCGAAACATTAAAGTGGCAAAACATGCAAAAATAAAATAGAAGAAACGAAAAGCCGATCAAGATAATTTAAAATACATTTTTGAAGCAAATTGAACAACACAAAAAGCTAGAATAATTTAAAATACATTTTGAGACAAGCTAACCATAAATGTTTTAGCACATTCTCAGAATCGAGAGTTGTGACATTTAAAAGCCCCATAAACAAAGGAATCTGAAAAAGCATTTTGGTTTAAAATAATATATAACTTGTGGCATTTTCAAGTTTTTGTTTTTTGCTTGTGGCCAGTTAAAGCGAAGCGAATTAGCTTTGTTGGCCTTATTGATTTTGCATTGCATAAGGCAATAGACGTGAAGAAGAAGAGCTGAGGGACTCAAAAATCGAATTAGTCAAGTCACAAAAATTGAAAATCATTAACTTGGCAGAAACCAAAAAAAAAAAAAAAAACGCTCAATTCGCCGCAGTTTTATGTCTTCACTAGCTTCGTCTCGCTCTGGCGCACCAAGAGAGAGCAGAGCCGCTGCGAGGCGGGGAAAATATACGCGGCCATCAAAGAGCGAAAGAGAGAGGGAGAATCTGTGGATGCACGAGTGTTTGTGCTTGTGTTTTTTTTCCATGTGTGTTTGTGTTTGGGAGAGCAAAAATCAGCAGACTGTTTGGCTCTGTCTCTTTGTGTTGTTGTTATTGTTGTGACTTTGTTTTGTTTTGCTGCGGCTTTTTTATTTTATTTCCTTACAATAAAAACGACAATAAAATTTTTTAGTGCAAAGATTTATGTGCGAACAATTTTTTTCGGCCACTCATTTTACCTATGTTGTTGGTCTTGTTGGCCCCAAAAATCTCTATTAATTTTGATATTGATTTCGGTTGAGTGCATAGAAACAACACGTTGTCAGGTCAATTTTTATTAGCTGAAAAATGTTGGGTAACTATTTGGGCTAACAATTTAATAACACAAAAATAATAACATTTAAAAAATCCGAAATTATTTAAGCCATATAAAATGCCACAAAATAAAGAATATTTATGCAGCAATCGAGCAGAAAAATAGCTAAAATAGTTTAACATTTTATTATTTTAGTTTTATAGTTTTATTATTATAGTTTAACATATTATTATTATTATTATCACGTAAAAACATTTGGAATTGATAACCAATTAAATAGATATTTTTCCACTGTTTGTTATTTTTGCCGATTTTATTTTTGGCCCTATTACGTTACATTTTAATTGGGCAGGTGCAGTTTCGCTGGCAATTTAATATTCCATTTAAGTTAAAAAAAAAAAAAAAAACAGTAGACACTTGCATTCGCTCTTGCCAAAAGGGGCGTGGCATTCGGGGGTGGCTCTTCGAGGGCGGGTGAATGTAAGGCTGGG
