# Supplementary material for: Redundant and Singular Regulatory Elements Underlie the Rapidly Evolving Pigmentation of Drosophila
Source: Mol Biol Evol. 2025 Sep 4;42(9):msaf213. doi: 10.1093/molbev/msaf213 (PMC12449766; doi:10.1093/molbev/msaf213)
Supplement: msaf213_Supplementary_Data [file msaf213_supplementary_data.zip › Supplementary Document S8 Eip74EF siR cassettes v1.docx]

**Supplement Document S8**

***Eip74EF* #1 & #6 siR chain cassette**

*Kpn*I

**GGTACC**CCGCCGGGATTCCGGTCTAGAGGAAGATCTTCCCATCCCATATTCAGCCAAGCTTAGT**CCGAAATTCCTATTGTCAAGC**tagttatattcaagcata**GCTTGACAATAGGAATTTCGG**GCGGATCCAGGCGAGACATCGGAGTTGAAACTAAAACTGAAATTTACTAGAAAACATCCCATAAAACATCCCATATTCAGCCGCTAGCAGT**CGAATTTATACCAGAACAATG**tagttatattcaagcata**CATTGTTCTGGTATAAATTCG**GC**GAATTC**

*Eco*RI

***Eip74EF* #4 & #9 siR chain cassette**

*Kpn*I

**GGTACC**CCGCCGGGATTCCGGTCTAGAGGAAGATCTTCCCATCCCATATTCAGCCAAGCTTAGT**CCGCTCTGCTCCACATAAAGA**tagttatattcaagcata**TCTTTATGTGGAGCAGAGCGG**GCGGATCCAGGCGAGACATCGGAGTTGAAACTAAAACTGAAATTTACTAGAAAACATCCCATAAAACATCCCATATTCAGCCGCTAGCAGT**AGCGAGGAACCACAATCAATG**tagttatattcaagcata**CATTGATTGTGGTTCCTCGCT**GC**GAATTC**

*Eco*RI
