# Supplementary material for: Redundant and Singular Regulatory Elements Underlie the Rapidly Evolving Pigmentation of Drosophila
Source: Mol Biol Evol. 2025 Sep 4;42(9):msaf213. doi: 10.1093/molbev/msaf213 (PMC12449766; doi:10.1093/molbev/msaf213)
Supplement: msaf213_Supplementary_Data [file msaf213_supplementary_data.zip › Supplementary Document S9 pRedEyePig Eip74EF siR chain sequence v1.docx]

**Supplement Document S9**

***pRedEyePig Eip74EF* #1 & #6 siR chain**

AAAAAAAAACAAAAACTCAAAATTTCTTCTATAAAGTAACAAAACTTTTA

3XP3 promoter region

GGATCTAATTCAATTAGAGACTAATTCAATTAGAGCTAATTCAATTAGGA

TCCAAGCTTATCGATTTCGAACCCTCGACCGCCGGAGTATAAATAGAGGC

GCTTCGTCTACGGAGCGACAATTCAATTCAAACAAGCAAAGTGAACACGT

CGCTAAGCGAAAGCTAAGCAAATAAACAAGCGCAGCTGAACAAGCTAAAC

DsRed Open Reading Frame

AATCGGCTCGAAGCCGGTCGCCACCATGGCCTCCTCCGAGGACGTCATCA

AGGAGTTCATGCGCTTCAAGGTGCGCATGGAGGGCTCCGTGAACGGCCAC

GAGTTCGAGATCGAGGGCGAGGGCGAGGGCCGCCCCTACGAGGGCACCCA

GACCGCCAAGCTGAAGGTGACCAAGGGCGGCCCCCTGCCCTTCGCCTGGG

ACATCCTGTCCCCCCAGTTCCAGTACGGCTCCAAGGTGTACGTGAAGCAC

CCCGCCGACATCCCCGACTACAAGAAGCTGTCCTTCCCCGAGGGCTTCAA

GTGGGAGCGCGTGATGAACTTCGAGGACGGCGGCGTGGTGACCGTGACCC

AGGACTCCTCCCTCCAGGACGGCTCCTTCATCTACAAGGTGAAGTTCATC

GGCGTGAACTTCCCCTCCGACGGCCCCGTAATGCAGAAGAAGACTATGGG

CTGGGAGGCGTCCACCGAGCGCCTGTACCCCCGCGACGGCGTGCTGAAGG

GCGAGATCCACAAGGCCCTGAAGCTGAAGGACGGCGGCCACTACCTGGTG

GAGTTCAAGTCCATCTACATGGCCAAGAAGCCCGTGCAGCTGCCCGGCTA

CTACTACGTGGACTCCAAGCTGGACATCACCTCCCACAACGAGGACTACA

CCATCGTGGAGCAGTACGAGCGCGCCGAGGGCCGCCACCACCTGTTCCTG

Tubulin 3’ Untranslated Region

TAGTAAACTAAGCGTCGCGCCACTTCAACGCTCGATGGGAGCGTCATTGG

TGGGCGGGGTAACCGTCGAAATCAGTGTTTACGCTTCCAATCGCAACAAA

AAATTCACTGCAACACTGAAAAGCATACGAAAACGATGAAGATTGTACGA

GAAACCATAAAGTATTTTATCCACAAAGACACGTATAGCAGAAAAGCCAA

GTTAACTCGGCGATAAGTTGTGTACACAAGAATAAAATCGGCCAGATTCA

GTGTTGTCAGAAATAAGAAAACCCCACTATGTTTTTCTTTGCCTTTTCTT

TCTCCCAGCGATCATTCATTTCGTGGTGAAAGAACGGGGTCATTGCACGG

AGTTTCGACTGCGGGAAAGCAGAGCTGCCGTTCACTTCGTCTATAATTAG

CGCTTTCTATTTTCCCCGATTCGGGCCGCTGCTGCGCTTTTCCGCCTGCT

GTTTGTGGCAAGTGTAGCAGCAGGCTGTGCACGCAGTGTGGCATGCACTT

GGCTTTCCACCGTTGGTATCGATTCTCTGGGACGATGAGTCATTCCTTTC

GGGGCCACAGCATAATCGTTGCCAGCTCACCGAAATGGTGACTTCATTTC

TTAACTGCCGTCAAGCATGCGATTGTACATACATACATATTTATATATGT

ACATATTTATGTGACTATGGTAGGTCGATATAATAGCAATCAACGCAAGC

AAATGTGTCAGTCCTGCTTACAGGAACGATTCTATTTAGTAATTTTCGTT

GTATAAAGTAATTATGTATGTATGTAAGCCCCATAAATCTGAAACAATTA

***Asc*I *Sbf*I**

GGCAAAACCATGCGAAGCTTCCTTCTG**GGCGCGCC**AAATTTAAA**CCTGCA**

UAS 1 UAS 2 UAS 3

**GG**TCGGAGTACTGTCCTCCGAGCGGAGTACTGTCCTCCGAGCGGAGTACT

UAS 4 UAS 5

GTCCTCCGAGCGGAGTACTGTCCTCCGAGCGGAGTACTGTCCTCCGAGCG

Heat inducible hsp70 promoter

GAGACTCTAGCGAGCGCTAGAATCCCAAAACAAACTGGTTATTGTGGTAG

GTCATTTGTTTGGCAGAAAGAAAACTCGAGAAATTTCTCTGGCCGTTATT

CGTTATTCTCTCTTTTCTTTTTGGGTCGCTCCCTCTCTGCACTAATGCTC

TCTCACTCTGTCACACAGTAAACGGCATACTGCTCTCGTTGGTTCGAGAG

AGCGCGCCTCGAATGTTCGCGAAAAGAGCGCCGGAGTATAAATAGAGGCG

CTTCGTCTACGGAGCGACAATTCAATTCAAACAAGCAAAGTGAACACGTC

GCTAAGCGAAAGCTAAGCAAATAAACAAGCGCAGCTGAACAAGCTAAACA

ATCTGCAGTAAAGTGCAAGTTAAAGTGAATCAATTAAAAGTAACCAGCAA

CCAAGTAAATCAACTGCAACTACTGAAATCTGCCAAGAAGTAATTATTGA

ATACAAGAAGAGAACTCTCAAAATGCGAGACCACTCATCGCCCAATCACT

AGTGAATTACCGGTAATAGGGAATTGGGAATTAATTCGTTAACAGATCTT

***Kpn*I** *Eip74EF* #1 & #6 siR chain cassette

CCTCTAGT**GGTACC**CCGCCGGGATTCCGGTCTAGAGGAAGATCTTCCCAT

CCCATATTCAGCCAAGCTTAGTCCGAAATTCCTATTGTCAAGCTAGTTAT

ATTCAAGCATAGCTTGACAATAGGAATTTCGGGCGGATCCAGGCGAGACA

TCGGAGTTGAAACTAAAACTGAAATTTACTAGAAAACATCCCATAAAACA

TCCCATATTCAGCCGCTAGCAGTCGAATTTATACCAGAACAATGTAGTTA

***Eco*RI**

TATTCAAGCATACATTGTTCTGGTATAAATTCGGC**GAATTC**AGGCGAGAC

ATCGGAGTTGAAACTAAAACTGAATTACTAGTCTCGAGGCTAGAGGATCT

TTGTGAAGGAACCTTACTTCTGTGGTGTGACATAATTGGACAAACTACCT

Small t intron

ACAGAGATTTAAAGCTCTAAGGTAAATATAAAATTTTTAAGTGTATAATG

TGTTAAACTACTGATTCTAATTGTTTGTGTATTTTAGATTCCAACCTATG

GAACTGATGAATGGGAGCAGTGGTGGAATGCCTTTAATGAGGAAAACCTG

TTTTGCTCAGAAGAAATGCCATCTAGTGATGATGAGGCTACTGCTGACTC

TCAACATTCTACTCCTCCAAAAAAGAAGAGAAAGGTAGAAGACCCCAAGG

ACTTTCCTTCAGAATTGCTAAGTTTTTTGAGTCATGCTGTGTTTAGTAAT

AGAACTCTTGCTTGCTTTGCTATTTACACCACAAAGGAAAAAGCTGCACT

GCTATACAAGAAAATTATGGAAAAATATTTGATGTATAGTGCCTTGACTA

GAGATCATAATCAGCCATACCACATTTGTAGAGGTTTTACTTGCTTTAAA

AAACCTCCCACACCTCCCCCTGAACCTGAAACATAAAATGAATGCAATTG

SV40 poly-A signal containing sequence

TTGTTGTTAACTTGTTTATTGCAGCTTATAATGGTTACAAATAAAGCAAT

AGCATCACAAATTTCACAAATAAAGCATTTTTTTCACTGCATTCTAGTTG

TGGTTTGTCCAAACTCATCAATGTATCTTATCATGTCTGGATCCATGCGT

piggyBac 5’ inverted repeat *attB*

CAATTTTACGCAGACTATCTTTCTAGGGGATGGGTGAGGTGGAGTACGCG

CCCGGGGAGCCCAAGGGCACGCCCTGGCACCCGCACCGCGGCTTCGAGAC

CGTGGCGGCCGCATCGGATGCCGGGACCGACGAGTGCAGAGGCGTGCAAG

CGAGCTTGGCGTAATCATGGTCATAGCTGTTTCCTGTGTGAAATTGTTAT

CCGCTCACAATTCCACACAACATACGAGCCGGAAGCATAAAGTGTAAAGC

CTGGGGTGCCTAATGAGTGAGCTAACTCACATTAATTGCGTTGCGCTCAC

TGCCCGCTTTCCAGTCGGGAAACCTGTCGTGCCAGCTGCATTAATGAATC

GGCCAACGCGCGGGGAGAGGCGGTTTGCGTATTGGGCGCTCTTCCGCTTC

CTCGCTCACTGACTCGCTGCGCTCGGTCGTTCGGCTGCGGCGAGCGGTAT

CAGCTCACTCAAAGGCGGTAATACGGTTATCCACAGAATCAGGGGATAAC

GCAGGAAAGAACATGTGAGCAAAAGGCCAGCAAAAGGCCAGGAACCGTAA

AAAGGCCGCGTTGCTGGCGTTTTTCCATAGGCTCCGCCCCCCTGACGAGC

ATCACAAAAATCGACGCTCAAGTCAGAGGTGGCGAAACCCGACAGGACTA

TAAAGATACCAGGCGTTTCCCCCTGGAAGCTCCCTCGTGCGCTCTCCTGT

TCCGACCCTGCCGCTTACCGGATACCTGTCCGCCTTTCTCCCTTCGGGAA

GCGTGGCGCTTTCTCATAGCTCACGCTGTAGGTATCTCAGTTCGGTGTAG

GTCGTTCGCTCCAAGCTGGGCTGTGTGCACGAACCCCCCGTTCAGCCCGA

CCGCTGCGCCTTATCCGGTAACTATCGTCTTGAGTCCAACCCGGTAAGAC

ACGACTTATCGCCACTGGCAGCAGCCACTGGTAACAGGATTAGCAGAGCG

AGGTATGTAGGCGGTGCTACAGAGTTCTTGAAGTGGTGGCCTAACTACGG

CTACACTAGAAGAACAGTATTTGGTATCTGCGCTCTGCTGAAGCCAGTTA

CCTTCGGAAAAAGAGTTGGTAGCTCTTGATCCGGCAAACAAACCACCGCT

GGTAGCGGTGGTTTTTTTGTTTGCAAGCAGCAGATTACGCGCAGAAAAAA

AGGATCTCAAGAAGATCCTTTGATCTTTTCTACGGGGTCTGACGCTCAGT

GGAACGAAAACTCACGTTAAGGGATTTTGGTCATGAGATTATCAAAAAGG

ATCTTCACCTAGATCCTTTTAAATTAAAAATGAAGTTTTAAATCAATCTA

AAGTATATATGAGTAAACTTGGTCTGACAGTTACCAATGCTTAATCAGTG

AGGCACCTATCTCAGCGATCTGTCTATTTCGTTCATCCATAGTTGCCTGA

CTCCCCGTCGTGTAGATAACTACGATACGGGAGGGCTTACCATCTGGCCC

CAGTGCTGCAATGATACCGCGAGACCCACGCTCACCGGCTCCAGATTTAT

CAGCAATAAACCAGCCAGCCGGAAGGGCCGAGCGCAGAAGTGGTCCTGCA

ACTTTATCCGCCTCCATCCAGTCTATTAATTGTTGCCGGGAAGCTAGAGT

AAGTAGTTCGCCAGTTAATAGTTTGCGCAACGTTGTTGCCATTGCTACAG

GCATCGTGGTGTCACGCTCGTCGTTTGGTATGGCTTCATTCAGCTCCGGT

TCCCAACGATCAAGGCGAGTTACATGATCCCCCATGTTGTGCAAAAAAGC

GGTTAGCTCCTTCGGTCCTCCGATCGTTGTCAGAAGTAAGTTGGCCGCAG

TGTTATCACTCATGGTTATGGCAGCACTGCATAATTCTCTTACTGTCATG

CCATCCGTAAGATGCTTTTCTGTGACTGGTGAGTACTCAACCAAGTCATT

CTGAGAATAGTGTATGCGGCGACCGAGTTGCTCTTGCCCGGCGTCAATAC

GGGATAATACCGCGCCACATAGCAGAACTTTAAAAGTGCTCATCATTGGA

AAACGTTCTTCGGGGCGAAAACTCTCAAGGATCTTACCGCTGTTGAGATC

CAGTTCGATGTAACCCACTCGTGCACCCAACTGATCTTCAGCATCTTTTA

CTTTCACCAGCGTTTCTGGGTGAGCAAAAACAGGAAGGCAAAATGCCGCA

AAAAAGGGAATAAGGGCGACACGGAAATGTTGAATACTCATACTCTTCCT

TTTTCAATATTATTGAAGCATTTATCAGGGTTATTGTCTCATGAGCGGAT

ACATATTTGAATGTATTTAGAAAAATAAACAAATAGGGGTTCCGCGCACA

TTTCCCCGAAAAGTGCCACCTGACGTCTAAGAAACCATTATTATCATGAC

ATTAACCTATAAAAATAGGCGTATCACGAGGCCCTTTCGTCTCGCGCGTT

TCGGTGATGACGGTGAAAACCTCTGACACATGCAGCTCCCGGAGACGGTC

ACAGCTTGTCTGTAAGCGGATGCCGGGAGCAGACAAGCCCGTCAGGGCGC

GTCAGCGGGTGTTGGCGGGTGTCGGGGCTGGCTTAACTATGCGGCATCAG

AGCAGATTGTACTGAGAGTGCACCATATGCGGTGTGAAATACCGCACAGA

TGCGTAAGGAGAAAATACCGCATCAGGCGCCATTCGCCATTCAGGCTGCG

CAACTGTTGGGAAGGGCGATCGGTGCGGGCCTCTTCGCTATTACGCCAGC

TGGCGAAAGGGGGATGTGCTGCAAGGCGATTAAGTTGGGTAACGCCAGGG

TTTTCCCAGTCACGACGTTGTAAAACGACGGCCAGTGAATTGGAGATCGG

TACTTCGCGAATGCGTCGAGATGGCCGGCCCAGGTGGCAGCACCTGCGAT

piggyBac 3’ inverted repeat

CTTAACCCTAGAAAGATAATCATATTGTGACGTACGTTAAAGATAATCAT

GCGTAAAATTGACGCATGTGTTTTATCGGTCTGTATATCGAGGTTTATTT

ATTAATTTGAATAGATATTAAGTTTTATTATATTTACACTTACATACTAA

TAATAAATTCAACAAACAATTTATTTATGTTTATTTATTTATT
